# High-Calorie Diets Exacerbate Lipopolysaccharide-Induced Pneumonia by Promoting Propionate-Mediated Neutrophil Extracellular Traps

**DOI:** 10.3390/nu17132242

**Published:** 2025-07-07

**Authors:** Yingqiu Sun, Hui Liu, Jiyu Jiang, Leyan Hu, Qingpu Ma, Shuxuan Li, Tiegang Liu, Xiaohong Gu

**Affiliations:** 1 Qi-Huang Chinese Medicine School, Beijing University of Chinese Medicine, Beijing 100029, China; 18951053150@163.com (Y.S.); huleyan1130@163.com (L.H.); 19809509189@163.com (Q.M.); 2 Institute of Chinese Medicine Epidemic Disease, Beijing University of Chinese Medicine, Beijing 102488, China; 13671081286@163.com; 3 School of Traditional Chinese Medicine, Beijing University of Chinese Medicine, Beijing 100029, China; 17667506961@163.com (J.J.); bzylishuxuan1998@163.com (S.L.)

**Keywords:** high-calorie diet, pneumonia, propionate, neutrophil extracellular traps, histone deacetylases

## Abstract

**Objectives**: High-calorie diets are linked to increased risks of chronic inflammation and immune dysfunction, yet their role in modulating pneumonia severity remains unclear. Focusing on the interactions among gut-originating short-chain fatty acids (SCFAs), neutrophil function, and histone deacetylases (HDACs), this research examined the exacerbating effects of a high-calorie diet on pneumonia in rats. **Methods**: Male Sprague-Dawley rats (3 weeks old, 110 ± 10 g) were allocated among four groups: normal diet (N), high-calorie diet (G), LPS-induced pneumonia (P), and high-calorie diet combined with lipopolysaccharide (LPS)-induced pneumonia (GP). LPS was administered via aerosolization for three days. Fecal, serum, and lung SCFA levels were quantified via GC-MS. Neutrophil extracellular traps (NETs) formation, neutrophil apoptosis, and HDAC activity were assessed using immunofluorescence, TUNEL assays, and qRT-PCR. Propionate supplementation and HDAC inhibitor (trichostatin A) interventions were applied to validate mechanistic pathways. **Results**: The group GP exhibited exacerbated lung inflammation, increased NETs release, and reduced neutrophil apoptosis compared to the group P. Propionate levels in feces, serum, and lung tissues decreased sharply in GP rats, correlating with elevated HDAC1/2/3/6 activity and reduced histone acetylation. Propionate supplementation or HDAC inhibition significantly attenuated lung injury, suppressed NETs, and restored neutrophil apoptosis. **Conclusions**: High-calorie diets exacerbate pneumonia by depleting gut-derived propionate, which drives HDAC-mediated NETs overproduction and impairs neutrophil apoptosis. Restoring propionate levels or targeting HDACs may offer therapeutic strategies for diet-aggravated respiratory diseases. Mechanistically, propionate-mediated HDAC inhibition demonstrates proof-of-concept efficacy in modulating H4 acetylation, warranting further investigation in disease-specific pneumonia models.

## 1. Introduction

Pneumonia, a widespread inflammatory lung condition triggered by pathogens such as bacteria and viruses [1,2,3], remains a leading cause of infant mortality in high-risk regions and the second most frequent cause of death among children under five globally [4,5,6], particularly affecting those over one year old [7,8]. Emerging evidence suggests that dietary habits significantly influence pneumonia severity [9,10]. Children frequently demonstrate a pronounced preference for diets with high caloric density, which is associated with an increased risk of chronic diseases in later life [11]. Such dietary choices have been correlated with adverse health outcomes, including the potential to impair optimal growth and developmental trajectories [12,13]. Recent research has demonstrated that high-calorie diets promote a low-grade inflammatory response, which may exacerbate immune dysfunction [14,15]. A proposed mechanism involves the compromised intestinal barrier integrity permitting enhanced LPS translocation into the bloodstream, subsequently triggering an inflammatory cascade within immune cells [16,17,18]. Previous studies have demonstrated that high-calorie diets induce dysbiosis within the gut microbiota of juvenile rats with pneumonia, consequently reducing the production of SCFAs. SCFAs, metabolites produced by gut bacteria, play critical roles in immune regulation and are essential for maintaining lung health [19,20,21]. These studies indicate that high-calorie diets may intensify pneumonia severity in children, underscoring significant implications for children’s health. Nevertheless, these underlying mechanisms remain incompletely understood.

SCFAs, principally acetic, propionic, and butyric acids produced via intestinal microbiota-mediated dietary fiber fermentation, critically bridge metabolic balance and immune function [22,23]. In human colons, acetic, propionic, and butyric acids, respectively, reach concentrations of 50–150 mM [24]. SCFAs, synthesized in the gut, enter the bloodstream and influence immune responses in distant organs, including the lungs. This highlights their importance in the ‘lung–gut axis’ [25], where they help maintain lung health by balancing immune activity and reducing the risk of severe inflammation [26,27]. HDACs and G protein-coupled receptors (GPCRs) serve as key targets for SCFAs, enabling their anti-inflammatory actions [28]. HDACs are enzymes found in the cytoplasm and nucleus that regulate gene expression by modifying chromatin structure. They control histone acetylation, which affects how DNA is packed into chromosomes [29,30]. SCFAs can activate histone acetylation through suppressing HDACs, reducing inflammation in the gut [31,32]. However, their role in lung inflammation via HDACs is still unclear and requires further study.

Neutrophils play vital roles in defense against pathogens. These cells combat infections through multiple mechanisms, including secreting inflammatory signals, engulfing microbes, and releasing NETs [33]. These NETs comprise DNA–protein complexes with antimicrobial properties that immobilize and neutralize invaders [34]. While essential for immunity, excessive NETs release has been linked to tissue damage and inflammation in conditions such as pneumonia and adult respiratory distress syndrome [35,36,37]. Clinical studies show that severe lung inflammation often coincides with heightened neutrophil activity and NET formation. This unchecked inflammation may lead to secondary respiratory diseases, like asthma and chronic obstructive pulmonary disease [38,39,40,41,42,43]. Emerging evidence suggests that SCFAs, metabolites produced by gut microbes, influence neutrophil behavior [44]. SCFAs attenuate pro-inflammatory cytokine release, neutrophil migration, and NETs formation, and enhance pathogen phagocytosis [45,46], potentially by inhibiting HDACs [47]. These enzymes control gene expression patterns that govern immune responses [48,49]. Understanding how SCFAs affect neutrophil activity and NETosis is critical for clarifying their role in lung inflammation. This study explores whether dietary factors that alter SCFA levels—such as high-calorie diets—impact LPS-induced lung injury, with implications for managing diet-related respiratory diseases.

Our recent research has shown that high-calorie nutritional regimens disturb gut microbial equilibrium in juvenile rats, consequently suppressing SCFA generation. This reduction appears to worsen the severity of pneumonia in these animals [19,20,21]. However, the exact mechanisms by which SCFAs reduce the release of NETs remain unclear. The current study contributes to evaluate the effects of a high-calorie diet on NETs release, SCFAs, and HDACs in juvenile rats with LPS-induced pneumonia. Additionally, we seek to uncover the mechanisms through which high-calorie diets exacerbate pneumonia by modulating SCFAs-mediated NETs release. The findings reveal the therapeutic potential of gut-microbiota-metabolite-targeted dietary strategies for pneumonia intervention.

## 2. Materials and Methods

### 2.1. Chemicals and Reagents

LPS, acetate, propionate, and butyrate were supplied by Sigma–Aldrich (St. Louis, MO, USA). Rat interleukin-1 beta (IL-1β), interleukin-6 (IL-6), tumor necrosis factor-alpha (TNF-α), and macrophage inflammatory protein-2 (MIP-2) enzyme-linked immunosorbent assay (ELISA) kits were obtained from BioLegend (San Diego, CA, USA). Antibodies against AcH4 were obtained from Cell Signaling Technology (Chicago, IL, USA). Antibodies against Ly6G and Histone H3 were obtained from Abcam (Cambridge, MA, USA).

### 2.2. Animal Experiments

Male Sprague-Dawley rats (3 weeks old, 110 ± 10 g) were supplied by Beijing SPF Biotechnology Co., Ltd., Beijing, China, experimental animal quality certificate no.: 110324241105092638 and accommodated in the animal laboratory of Beijing University of Chinese Medicine. Under Ethics Approval BUCM-2023121107-4245 from Beijing University of Chinese Medicine’s Animal Care Committee, studies used SPF-housed rats provided ad libitum food/water following approved care protocols.

High-calorie fodder was supplied by Beijing SPF Biotechnology Co., Ltd., and shared the same shape with rat standard maintenance fodder. The high-calorie fodder exhibits nutritionally engineered hypercaloric properties, delivering 5.4× greater energy density (1828 kJ/100 g) than standard maintenance fodder (340 kJ/100 g). This caloric amplification stems primarily from elevated lipid (16.10% vs. 4.62%) and carbohydrate (58.80% vs. 5.25%) content, coupled with reduced protein (13.73% vs. 16.26%). Our prior studies have delineated the comprehensive composition of high-calorie fodder and clarified the sample size [19]. Following 3-day acclimatization period, the forty rats were randomly assigned to four distinct groups based on their initial body weights: normal diet (N), high-calorie diet (G), LPS-induced pneumonia (P), and high-calorie diet combined with LPS-induced pneumonia (GP). Randomization was performed using a computer-generated random sequence via the random number generator function in Microsoft Excel (2019) to ensure unbiased allocation of rats into different groups.

As depicted in Table 1, Groups G and GP received high-calorie fodder during the whole experiments, while Groups N and P consumed rat standard maintenance feed, with 5 rats in each cage. During the last 3 days of the experimental protocol, Groups P and GP underwent daily 30-min aerosol exposures to 0.5 mg/mL LPS solution. Concurrently, Groups N and G were subjected to an equivalent volume and frequency of physiologic saline aerosolization as a control measure. The validation of the pneumonia model’s efficacy will be established through systematic histopathological examination of pulmonary tissues, wherein successful induction is defined by characteristic lesions, including the following: (1) pronounced peribronchial and alveolar infiltration of neutrophils, lymphocytes, and macrophages; (2) disruption of alveolar septal architecture; (3) interstitial edema. Rats exhibiting mortality or significant behavioral deviations from baseline were excluded from the study. The rats were monitored for their general health status and body weights were recorded daily. On the seventh day of the experiment, all rats were subjected to a 12 h fasting period, followed by anesthesia induction via intraperitoneal administration of 2% sodium pentobarbital at a dosage of 1 mL per 100 g of body weight. Blood samples were collected from the abdominal aorta for peripheral serum. The whole lung tissues were rinsed with physiological saline to remove any residual blood, then blotted dry with absorbent paper to remove surface moisture, and the weights were recorded. Portions of the lung samples were fixed in 4% paraformaldehyde solution for histopathological examination. For subsequent biochemical assays, pulmonary tissue sections and fecal specimens underwent rapid cryopreservation via immersion in liquid nitrogen followed by long-term storage at −80 °C in ultralow temperature freezers.

### 2.3. Blinding Procedures

To minimize bias, rats received unique identifiers from researcher XHG and underwent stratified randomization by initial body weight into four coded groups (A, B, C, D). These codes concealed the true group assignments (N, G, P, GP). Only researchers QPM and SXL maintained group-diet-nebulization mappings; all others remained blinded to group identities until final analysis. Biological specimens were collected in coded tubes (A–D) by personnel without group allocation knowledge. Independent pathologists analyzed histopathological and immunofluorescence results using unique identifiers, devoid of experimental metadata. The researcher YQS conducted statistical evaluations without group assignment information. Due to overt phenotypic differences from diet-induced obesity, daily monitoring caregivers (QPM and SXL) could not be blinded; endpoint analyses strictly adhered to blinding protocols.

### 2.4. Lung Histopathology

Harvested pulmonary tissues underwent immersion in 4% paraformaldehyde solution for a duration of 24 h for fixation, followed by standard ethanol-xylene dehydration and then impregnated with paraffin for embedding. Embedded specimens were sagittally sectioned at 5 μm thickness. Resultant slices were hematoxylin and eosin (H&E)-stained (hematoxylin: 5 min; eosin: 3 min) to facilitate histological examination by the optical microscope. For each experimental group, a random selection of 8 to 10 fields of view at a magnification of 200× was made, and the photograph was taken for further analysis.

### 2.5. ELISA

Pulmonary concentrations of IL-1β, IL-6, TNF-α, and MIP-2 were quantified using manufacturer-specified ELISA assays. Optical density measurements taken at 450 nm were acquired in duplicate via a SpectraMax^®^ i3x microplate reader (Molecular Devices, Silicon Valley, CA, USA) with data normalized against standard curves.

### 2.6. Drug Administration

First, 10 mg/mL trichostatin A (TSA) (MedChemExpress, Monmouth, NJ, USA) stock solution was prepared by dissolving 5 mg of TSA in 500 μL of DMSO. For in vivo administration, 80 μL stock was diluted in 3.92 mL physiological saline (final concentration: 0.2 mg/mL). Based on receiving the same diet and aerosolization solution as the GP group, GP + TSA group was administered a daily intraperitoneal injection of the aforementioned solution at 0.5 mg/kg, while the solvent control group received an equivalent volume of the solution consisting of 2% DMSO and 98% physiological saline via intraperitoneal injection.

Building upon the same dietary and aerosolization solution as the GP group, GP + propionate group was supplied with 67 mM sodium propionate in drinking water, consistent with our preceding study [19].

### 2.7. Real-Time PCR

Total RNA extraction from pulmonary samples was conducted utilizing TRIzol reagent (Invitrogen, Austin, TX, USA). The integrity and purity of the isolated RNA were assessed via spectrophotometric analysis. For the synthesis of complementary DNA (cDNA), the cDNA Reverse Transcription Kit was employed, utilizing 1 mg of total RNA per reaction, in strict accordance with the manufacturer’s guidelines. Post-cDNA synthesis, the quantification of relative mRNA expression levels was carried out on the CFX96 Real-Time PCR Detection System (Bio-Rad Laboratories, Hercules, CA, USA). Each PCR reaction was executed in triplicate to ensure experimental rigor and reproducibility. Quantitative data analysis was performed employing the comparative CT method, wherein cycle threshold values were normalized against the endogenous reference gene glyceraldehyde 3-phosphate dehydrogenase (GAPDH) prior to relative fold-change calculations, to account for variations in sample input and amplification efficiency. The primer sequences for amplification were meticulously designed based on the genomic clone sequences, and a comprehensive list of these primers is presented in Table 2.

### 2.8. Fecal/Serum/Lung Tissue SCFAs Analysis

SCFA levels in fecal, serum, and lung tissues were quantified employing gas chromatography-mass spectrometry (GC-MS). Standard curves were constructed using gradient concentrations of acetate, propionate, and butyrate. For sample preparation, 30 mg of fecal, serum, and lung materials was suspended in phosphoric acid (900 μL, 0.5%), vigorously mixed (2 min), and then centrifuged (14,000× *g*, 10 min). The supernatant (800 μL) was extracted with an equal volume of ethyl acetate, mixed again (2 min), and centrifuged (14,000× *g*, 10 min). To the upper organic phase (600 μL), 4-methylvaleric acid was introduced as an internal standard (500 μM final concentration), and subsequently, 1 μL of this supernatant was injected into the GC-MS system (Agilent DB-WAX capillary GC column, 10:1 split). The GC oven temperature program initiated at 90 °C, ramped to 120 °C (10 °C per minute), and held for 2 min. Subsequently, the temperature was increased to 150 °C (5 °C per minute) and maintained for 2 min. Finally, the temperature was ramped to 250 °C (25 °C per minute) and held for 2 min. Nitrogen serves as the carrier gas. Mass spectrometry detection was performed, then integrating the chromatographic peak areas and determining retention times. Based on the standard curve, the levels of SCFAs in the fecal, serum, and lung tissues were quantified.

### 2.9. Immunofluorescence

Lung tissues harvested immediately post-anesthesia were ice-embedded in optimal cutting temperature (OCT) matrix and cryosectioned at 20 μm thickness using a Leica CM1950 cryostat (Leica Biosystems, Shanghai, China). Resultant sections were transferred to pre-chilled glass slides and fixed with a 1% paraformaldehyde solution at room temperature for 10 min to preserve cellular morphology. Following fixation, the sections were permeabilized with 0.1% Triton X-100 for 10 min at room temperature to enhance antibody penetration. After permeabilization, the sections were blocked with 10% bovine serum in phosphate-buffered saline for half an hour to reduce non-specific antibody binding. Slides were treated with primary antibodies: anti-Ly6G, anti-histone H3, and anti-AcH4, followed by species-matched fluorescent secondary antibodies (1 h, RT). The nuclei were counterstained with 4′,6-diamidino-2-phenylindole (DAPI). To visualize the stained sections and to prevent channel crosstalk and saturation, a confocal laser-scanning microscope (Olympus FV3000, Olympus, Tokyo, Japan) was employed.

Additionally, Cellular DNA fragmentation was evaluated using the TUNEL assay (In Situ Cell Death Detection Kit, #11684795910, Roche, Basel, Switzerland), employing terminal deoxynucleotidyl transferase-mediated dUTP end-labeling, a hallmark of apoptosis, following the instructions. This assay helps to identify apoptotic cells within the tissue sections.

### 2.10. Statistical Analysis

Statistical analyses and graphical visualization were conducted using R software (version 4.3.2) [50]. The following R packages were utilized for data processing and statistical analysis: ggpubr, ggplot2, tidyverse, readr, forcats, broom, readxl, rstatix, and patchwork. These packages were installed using the install.packages() function in R. Outliers were detected and removed using the interquartile range method during data preprocessing to ensure the robustness of the analysis. Normality and homogeneity of variance were evaluated via the Shapiro–Wilk test and Levene’s test, respectively. Experimental results conforming to a normal distribution will be expressed as mean ± standard deviation and analyzed using two-tailed unpaired Student’s *t*-tests. Non-normal datasets will be analyzed using non-parametric tests to assess significant differences. For data with a normal distribution, statistical comparisons were conducted using the two-tailed unpaired Student’s *t*-test. Statistical significance was determined at the threshold of *p* < 0.05, and exact *p*-values and confidence intervals are provided in Supplementary Table.

## 3. Results

### 3.1. High-Calorie Diets Exacerbate Pneumonia-Associated Lung Injury in Juvenile Rats

**Figure 1:**
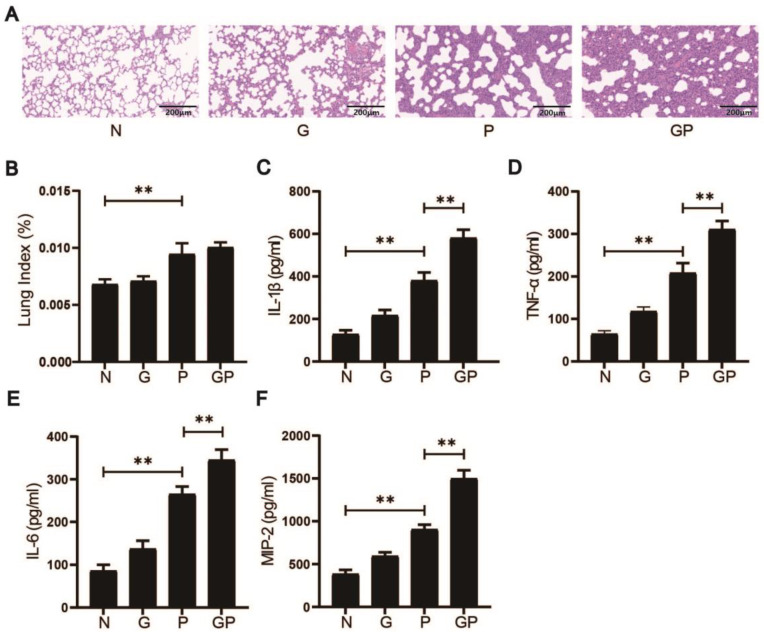
High-calorie diets exacerbate pneumonia-associated lung injury in juvenile rats; (**A**) H&E staining results (200×); (**B**) Lung index; (**C**–**F**) Determination of pro-inflammatory cytokines including IL-1β, IL-6, TNF-α, and MIP-2 in pulmonary samples in each group by ELISA. Results quantified as arithmetic mean ± SD with *n* = 6 independent observations. “**” *p* < 0.01.

To assess the effects of a high-calorie diet on lung inflammation in juvenile rats with LPS-induced pneumonia, we conducted histological analyses of lung tissue using H&E staining and measured the lung-to-body weight ratio as an indicator of inflammation. Histological examination revealed that after LPS exposure, rats in the P group showed thickened alveolar septa with neutrophil and lymphocyte infiltration around pulmonary veins and bronchial vessels. In contrast, rats in the GP group exhibited more severe lung damage, including extensive neutrophil infiltration, alveolar collapse, and pronounced inflammation (Figure 1A).

Quantitative analysis of the lung index showed a significant increase in the P group compared to the N group and the GP group had a further elevated lung index compared to the P group, consistent with worsened lung pathology (*p* < 0.01 P vs. N; Figure 1B). We also measured inflammatory cytokine levels in lung tissue using ELISA. Results showed significant increases in pro-inflammatory mediators—IL-1β, IL-6, TNF-α, and MIP-2—in LPS-exposed rats (*p* < 0.01 P vs. N; Figure 1C–F). Cytokine levels were further augmented in the GP cohort relative to the P group (*p* < 0.01), reflecting an amplified inflammatory response. The data demonstrate that high-calorie diets exacerbate pneumonia-associated lung injury in juvenile rats.

### 3.2. High-Calorie Diets Promote Neutrophil Recruitment and NETosis While Inhibiting Apoptosis

**Figure 2:**
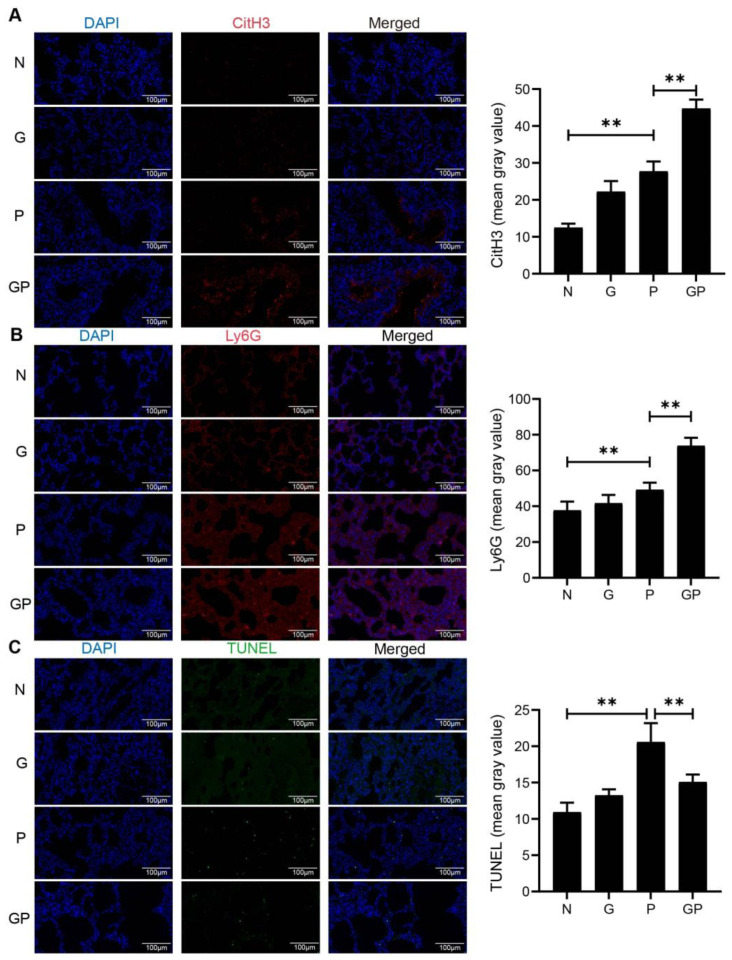
High-calorie diets promote neutrophil recruitment and NETosis while inhibiting apoptosis; (**A**–**C**) Immunofluorescence staining of rat lung tissue for CitH3, Ly6G, and TUNEL. Results quantified as arithmetic mean ± SD with *n* = 6 independent observations. “**” *p* < 0.01.

Given neutrophils’ critical involvement in pulmonary infections and the significance of NETs release in exacerbating lung inflammation, this study investigates the influence of high-calorie diets on NETs release from neutrophils in the lungs of rats with pneumonia. To investigate the impact of high-calorie diets on neutrophil recruitment and inflammation in the lungs of juvenile rats with pneumonia, we employed immunofluorescence staining using Ly6G as a neutrophil marker [51]. Our results revealed a significant increase in the mean fluorescence intensity of Ly6G in the pulmonary tissues of juvenile rats with pneumonia (*p* < 0.01 vs. N; Figure 2B), and then further elevated after the administration of high-calorie diets (*p* < 0.01 vs. P; Figure 2B). Similarly, assessed by CitH3 staining [52], the release of NETs was significantly increased in both pneumonia-affected rats and those fed high-calorie diets (*p* < 0.01 P vs. N; *p* < 0.01 P vs. GP; Figure 2A). In contrast, apoptosis levels, measured by the TUNEL assay [53], were elevated in the pulmonary tissues of juvenile rats with pneumonia but were significantly reduced after the introduction of high-calorie diets (*p* < 0.01 P vs. N; *p* < 0.01 P vs. GP; Figure 2C). This experiment revealed that rats fed high-calorie diets exhibited heightened neutrophil recruitment in lung tissue, elevated levels of NETs release, and reduced neutrophil apoptosis compared to those on a normal diet. Following LPS nebulization, these effects were further pronounced in high-calorie diet-fed rats with pneumonia (group GP) relative to normal diet-fed rats with pneumonia (group P). Our findings indicate that high-calorie diets may enhance neutrophil recruitment, promote the release of NETs, and suppress neutrophil apoptosis in juvenile rats with pneumonia.

### 3.3. High-Calorie Diets Reduce SCFAs Levels, Particularly Propionic Acid

**Figure 3:**
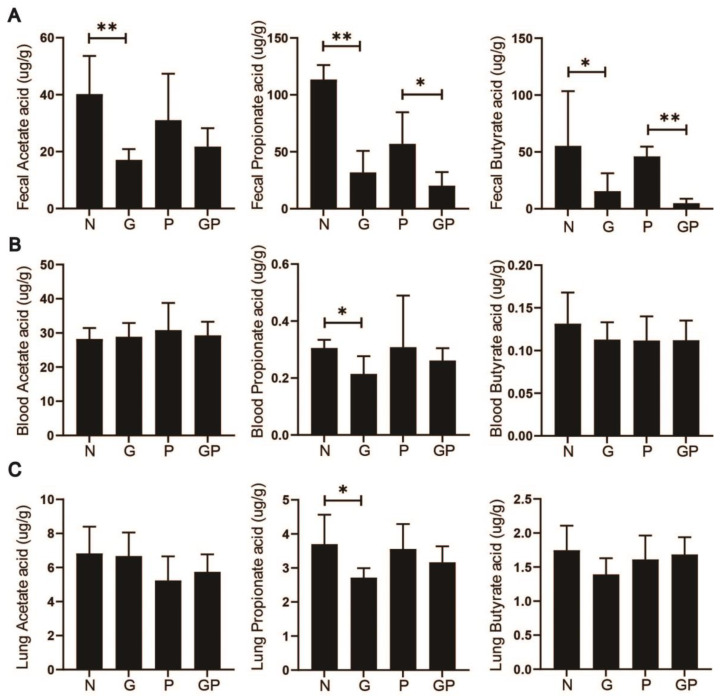
High-calorie diets reduce SCFAs levels, particularly propionic acid. (**A**) Acetic, propionic, and butyric concentrations in feces of juvenile rats; (**B**) Acetic, propionic, and butyric concentrations in serum of juvenile rats; (**C**) Acetic, propionic, and butyric concentrations in lung tissue of juvenile rats. Results quantified as arithmetic mean ± SD with *n* = 6 independent observations. “*” *p* < 0.05, “**” *p* < 0.01.

SCFAs are essential for modulating immune defenses within the alveoli, and their levels in the lungs demonstrate inverse relationships with pathological injury grading. Fecal metabolomic profiling via mass spectrometry in juvenile rats indicated a significant reduction in SCFA levels in both the G and GP groups, with acetic and propionic acid showing the most pronounced decreases. Given the systemic circulation of SCFAs and their potential to influence immune responses in distant organs, we further assessed SCFA levels in serum and lung tissues. Our findings indicate a differential decrease in serum SCFA levels in both groups, with propionic acid exhibiting the most substantial reduction. Similarly, lung tissue SCFA levels were predominantly characterized by a decline in propionic acid (Figure 3A–C). In summary, this study reveals that high-calorie diets reduce SCFA content in the intestines, serum, and pulmonary tissues of juvenile rats with pneumonia, especially with a particularly notable impact on propionic acid levels in the lungs.

### 3.4. Propionate Restoration and HDAC Inhibition Reduce Lung Injury Induced by Pneumonia and High-Calorie Diets

**Figure 4:**
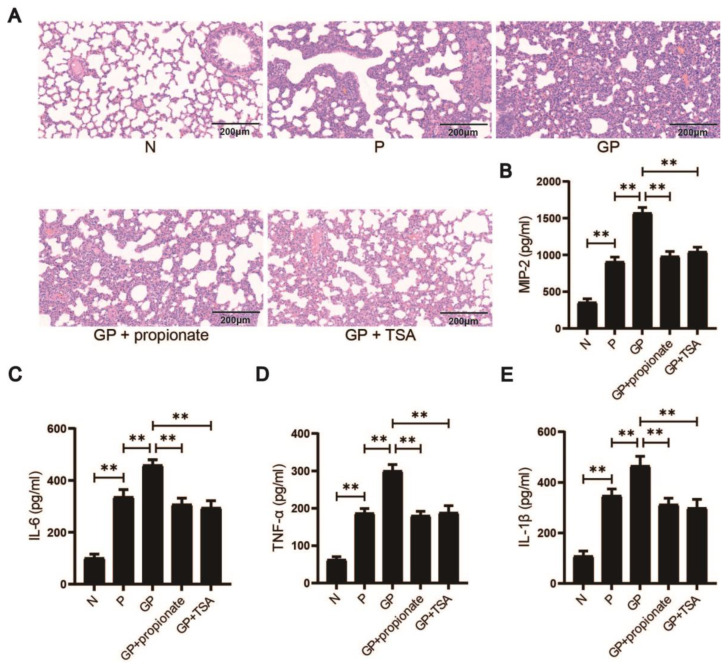
Propionate restoration and HDAC inhibition reduce lung injury induced by pneumonia and high-calorie diets; (**A**) H&E staining results (200×); (**B**–**E**) Determination of pro-inflammatory cytokines including IL-1β, IL-6, TNF-α, and MIP-2 in the pulmonary tissues in each group by ELISA. Results quantified as arithmetic mean ± SD with *n* = 6 independent observations. “**” *p* < 0.01.

To investigate the contribution of propionate to high-calorie diet-driven pneumonia severity, we supplemented rats in the group GP with propionate via drinking water and administered intraperitoneal injections of TSA, a broad-spectrum HDAC inhibitor. Histological analysis of lung tissue via H&E staining revealed that rats receiving propionate (GP + propionate group) or TSA (GP + TSA group) exhibited reduced alveolar damage, thinner alveolar septa, decreased lymphocyte infiltration, and lower neutrophil counts compared to untreated GP rats (Figure 4A). Inflammatory cytokine levels in lung tissue were also assessed. Both the GP + propionate and GP + TSA groups showed significant reductions in pro-inflammatory cytokines, including IL-1β, IL-6, TNF-α, and MIP-2, (*p* < 0.01 GP vs. GP + propionate; *p* < 0.01 GP vs. GP + TSA; Figure 4B–E). The data suggests that high-calorie diets exacerbate lung injury by reducing propionate levels, which diminishes HDAC inhibition. Supplementing with propionate or TSA mitigated lung injury, emphasizing the critical contribution of propionate in inhibiting HDAC to reduce immune responses. This indicates that propionate’s anti-inflammatory effects during caloric excess are mediated, at least in part, through HDAC-dependent pathways.

### 3.5. High-Calorie Diets May Reduce Propionate Levels, Potentially Activating HDAC Receptors 1, 2, 3, and 6

**Figure 5:**
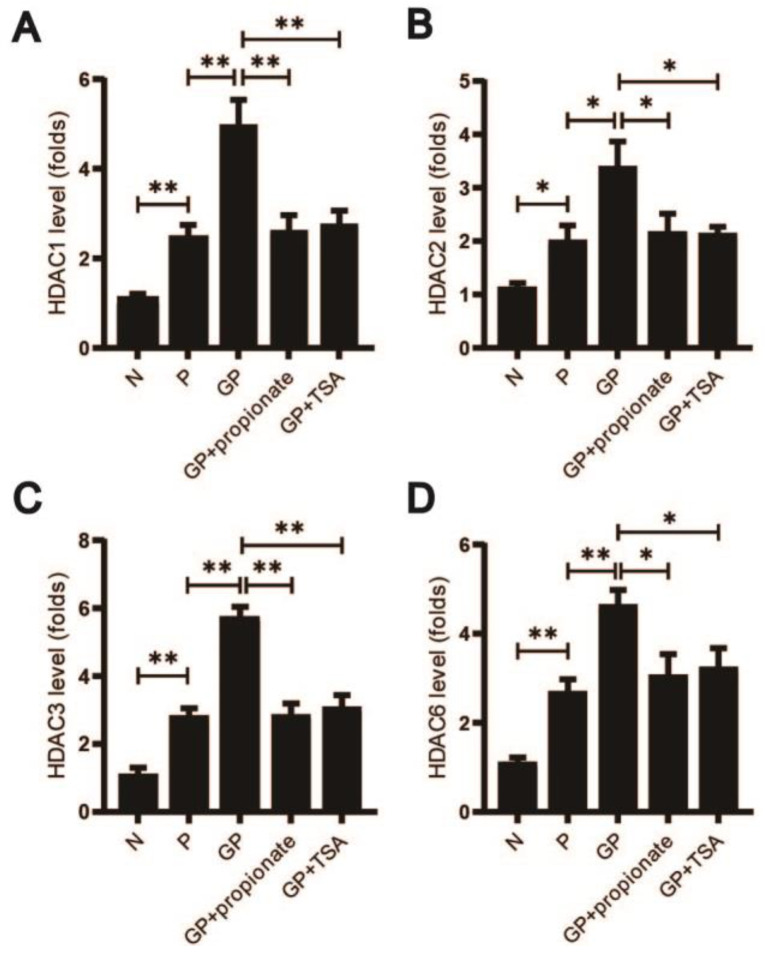
High-calorie diets may reduce propionate levels, potentially inhibiting the activity of HDAC receptors 1, 2, 3, and 6. (**A**–**D**) Transcript levels of HDAC isoforms 1, 2, 3, and 6 in pulmonary tissue were evaluated using RT-PCR. Results quantified as arithmetic mean ± SD with *n* = 3 independent observations. “*” *p* < 0.05, “**” *p* < 0.01.

Propionate is known to suppress HDAC receptors, particularly HDAC1, 2, 3, and 6 [54,55]. To determine whether the high-calorie diet-induced reduction in propionate affects these receptors, we measured the expression of HDAC 1, 2, 3, and 6 in lung tissue. Rats fed a high-calorie diet and infected with pneumonia exhibited significantly higher levels of HDAC 1, 2, 3, and 6 compared to those on a normal diet with similar infections. Treatment with concanavalin or TSA, which inhibit HDAC activity, significantly reduced these HDAC levels (*p* < 0.05 N vs. P; *p* < 0.05 GP vs. P; *p* < 0.05 GP vs. GP + propionate; *p* < 0.05 GP vs. GP + TSA; Figure 5A–D). The data reveal that high-calorie diets may reduce propionate levels, thereby decreasing the inhibition of HDAC 1, 2, 3, and 6 in lung tissue, potentially exacerbating pneumonia.

### 3.6. Propionate Has Been Observed to Inhibit the Release of NETs from Lung Tissue, an Effect That May Be Associated with the Enhancement of AcH4 Acetylation

**Figure 6:**
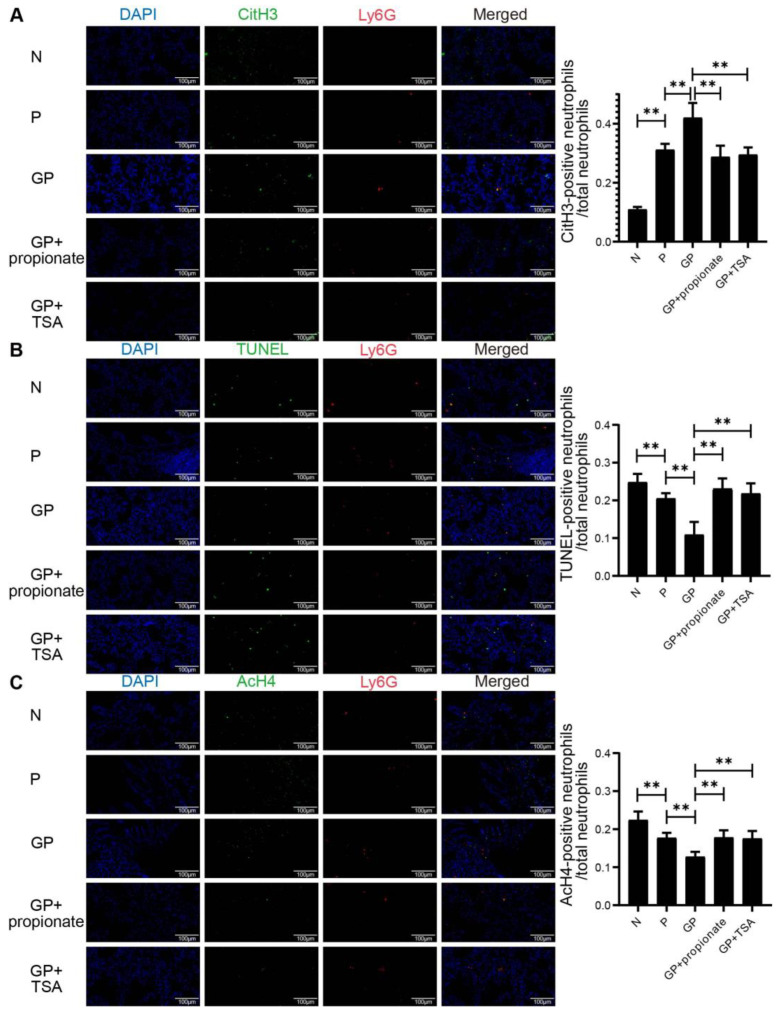
Propionate has been observed to inhibit the release of NETs from lung tissue, an effect that may be associated with the enhancement of AcH4 acetylation. (**A**) Results of immunofluorescence co-localization of CitH3 and Ly6G in pulmonary tissues. (**B**) Results of immunofluorescence co-localization of TUNEL and Ly6G in pulmonary tissues. (**C**) Results of immunofluorescence co-localization of AcH4 and Ly6G in pulmonary tissues. Results quantified as arithmetic mean ± SD with *n* = 6 independent observations. “**” *p* < 0.01.

While HDACs 1, 2, and 3 exhibit predominant nuclear localization [56], HDAC 6 is likewise detected within the nucleus [57]. Our study found that increased HDAC activity inhibited histone acetylation, leading to chromatin decondensation and enhanced NETs formation, while simultaneously suppressing neutrophil apoptosis. SCFAs, which inhibit HDAC activity, counteract these effects. Using immunofluorescence co-localization, we observed that the proportion of neutrophils releasing NETs from lung tissues was significantly higher in group P compared to group N, and this increase was exacerbated by high-calorie diets (*p* < 0.01 N vs. P; *p* < 0.01 GP vs. P; Figure 6A). Conversely, treatment with TSA or propionic acid supplementation significantly reduced NET release (*p* < 0.01 GP vs. GP + propionate; *p* < 0.01 GP vs. GP + TSA; Figure 6A). Additionally, neutrophil apoptosis was significantly decreased in rats with pneumonia and further reduced by high-calorie diets (*p* < 0.01 N vs. P; *p* < 0.01 GP vs. P; Figure 6B). However, in rats treated with TSA or propionic acid, neutrophil apoptosis rates significantly increased (*p* < 0.01 GP vs. GP + propionate; *p* < 0.01 GP vs. GP + TSA; Figure 6B). Immunofluorescent visualization revealed parallel AcH4 expression patterns within pulmonary tissue neutrophils with apoptosis, assessed by TUNEL staining (*p* < 0.01 N vs. P; *p* < 0.01 GP vs. P; *p* < 0.01 GP vs. GP + propionate; *p* < 0.01 GP vs. GP + TSA; Figure 6C).

Our findings indicate that a high-calorie diet significantly increases NET release from lung tissue in a pneumonia model, suppresses neutrophil apoptosis, and is associated with reduced histone acetylation in neutrophils. In contrast, propionate treatment effectively reverses these effects, suggesting that propionate may inhibit NET release by enhancing AcH4.

## 4. Discussion

Emerging evidence underscores the crucial role of gut microbiota in regulating pulmonary immune defenses [58]. SCFAs, key players in the gut–lung axis, exert broad effects on the function of inflammatory cells, including neutrophils and macrophages [59], and the inflammatory mediators they produce [60]. These effects impact lung health, affecting conditions like lung injury [61] and respiratory infections [62]. However, the precise mechanisms by which SCFAs inhibit pneumonia development in children through changes in lung immunity remain unclear. Our study demonstrates that a high-calorie diet reduces SCFA levels, particularly propionate, a gut microbiota metabolite, while simultaneously promoting NETs release. This process worsens pneumonia severity. Mechanistically, the decrease in propionate depletion from caloric excess affects the balance between NETosis and neutrophil apoptosis through nuclear HDAC 1/2/3/6 receptors, leading to increased histone acetylation. Our study emphasizes the significant influence of the gut microbiota metabolite propionate in pneumonia pathogenesis. They suggest propionate could be critical in pneumonia development and a viable candidate for clinical intervention against inflammatory diseases. Further study is needed to explore the clinical implications of modulating propionate levels in the context of dietary interventions for pneumonia.

Given that LPS, a key component of Gram-negative bacterial outer membranes, triggers potent inflammatory responses, we employed aerosolized LPS exposure to induce pneumonia in rats, modeling clinical pneumonia pathogenesis [63]. To be specific, LPS induces acute pneumonia through multiple signaling pathways, including Toll-like receptor4 engagement [64], neutrophil extracellular trap formation [65], and potentiation of neutrophil phagocytic and migratory capacities [66]. SD rats at early developmental stages are commonly employed to model pediatric disorders [67], and therefore, we used 3-week-old juvenile SD rats to study childhood pneumonia pathogenesis. Similarly, Bahader et al. established a pediatric traumatic brain injury model in 4-week-old SD rats, an age approximating human childhood physiology, and induced post-traumatic brain injury Streptococcus pneumoniae infection [68]. Consistent with our prior pediatric pneumonia model established in 3-week-old SD rats, we maintained this age-matched experimental paradigm [19]. Our model demonstrates that high-calorie diets exacerbate pediatric pneumonia severity. This aligns with clinical evidence from a 12-month-follow-up prospective cohort study (*n* = 334), where excessive caloric intake positively correlated with recurrent respiratory infection rates in children and adolescents [69]. Recently, Guzman et al. identified obesity (prevalence: 23.3%) as an established risk factor for severe COVID-19 in adolescents through their analysis of BMI in pediatric patients [70].

High-calorie diets alter gut microbiota composition, impairing the intestinal immune barrier and gut microbiota metabolites, SCFAs, which can affect the immune cells of distant organs, including the lungs [71]. Our prior research showed that such diets modulate gut microbiota and reduce bacteria-producing SCFAs, worsening pneumonia severity [19]. To be specific, the 16S rRNA sequencing revealed decreased gut microbiota α-diversity in high-calorie diet rats versus normal diet rats, which was independent of pneumonia status [19]. Key alterations included depletion of Lactobacillus and Muribaculaceae taxa associated with propionate biosynthesis pathways [72,73]. Gurav et al. demonstrated that butyrate modulates immune responses, protecting against colitis and colorectal cancer [74]. Sumbria et al. revealed that propionate supplementation could alleviate chronic corneal damage induced by herpes simplex virus by regulating inflammatory factors [75]. Shi et al. showed that propionate alleviates herpes-induced eye damage and allergic diseases by inhibiting HDAC receptors on basophils [76]. This study showed how caloric excess reduced SCFA levels in juvenile rats’ intestines, blood, and lungs, regardless of pneumonia infection. Notably, the extent of the decrease varied among different SCFAs, while propionate levels declined sharply in feces, serum, and lung tissue. This variation may likely be attributed to dietary factors, intestinal pH, peptide availability, and cellular receptor affinity [77]. For instance, intestinal pH and nutrient supply influence SCFA production, while tissue-specific SCFA absorption affects their distribution. Walker et al. demonstrated that controlling intestinal pH and peptide supply can modulate the human gut microbiota, thereby modulating the production of acetic, propionic, and butyric acid [78]. Cummings et al. observed through autopsy that colon epithelium and hepatocytes differentially absorb SCFAs, leading to varying concentrations in the portal, hepatic, and peripheral veins [79]. This suggests that different tissues and organs have distinct requirements for SCFAs. Propionate, absorbed into the bloodstream, travels to the lungs to modulate immunity. Previous studies demonstrated that altering gut propionate via fecal microbiota transplantation mitigated lung injury in mice with bacterial pneumonia [80]. Similarly, our propionate supplementation in high-calorie diet-fed rats with LPS-induced pneumonia reduced lung inflammation, confirming that diet-induced propionate loss exacerbates pneumonia. These findings reveal the critical influence of gut-derived propionate in lung health. By reducing propionate, high-calorie diets disrupt the immune balance, promoting LPS-reduced inflammation and lung injury. Targeting SCFA metabolism, particularly propionate, may offer therapeutic strategies to counteract diet-related respiratory complications.

Propionate influences the progression of pneumonia, potentially through its modulation of neutrophils, macrophages, and their cytokine functions [81]. Studies have shown that propionate mitigates pneumonia severity and lung tissue remodeling by reducing the infiltration of neutrophils, eosinophils, and lymphocytes, and by interfering with the release of chemokine C-X-C Motif Chemokine Ligand 1(CXCL1) and inflammatory factor TNF-α [82]. Our findings align with these observations: a high-calorie diet lowered propionate levels in the gut, blood, and lungs, resulting in heightened neutrophil recruitment, increased NETs release, and reduced neutrophil apoptosis. Conversely, propionate supplementation curbed NET release and promoted neutrophil apoptosis, indicating its role in reducing lung NET formation. Propionate and other SCFAs act on neutrophils via GPCRs and by inhibiting HDACs. Propionate’s effects are primarily HDAC-mediated; it acts as a non-competitive HDAC inhibitor, achieving ~60% maximum efficiency [83]. Substantial evidence indicates SCFAs reduce NET formation primarily through HDAC receptors [84,85]. Specifically, propionate and butyrate exclusively induce neutrophil death modalities, via HDAC-dependent mechanisms rather than GPCR pathways, as demonstrated across multiple experimental systems [86,87]. Our findings confirm this paradigm: propionate regulates neutrophil death through inhibiting HDAC receptors, including reducing NETosis and improving apoptosis. Vinolo et al. showed that propionate inhibits LPS-stimulated neutrophil release of inflammatory factors, a process potentially mediated by the inhibition of HDACs activity and activation of nuclear factor-κB (NF- κB) [88]. Propionate selectively inhibits HDAC 1, HDAC 2, HDAC 3, and HDAC 6 receptors [44,54,55], as evidenced in our study, where we observed elevated HDAC 1, HDAC 2, HDAC 3, and HDAC 6 levels in the pulmonary tissues of rats with pneumonia. These levels were further increased following caloric excess but significantly decreased with propionate supplementation. Additionally, intraperitoneal administration of TSA in rats with caloric excess combined with pneumonia resulted in decreased HDACs levels and alleviated pneumonia severity, further supporting the role of propionate in mitigating pneumonia through HDACs inhibition.

While HDAC inhibitors are clinically deployed primarily in hematological malignancies, their potential in viral and inflammatory diseases remains preclinical [80]. Our findings reveal HDAC-modulated pathways in diet-exacerbated pneumonia, suggesting novel therapeutic avenues. However, clinical translation requires caution: systematic reviews report significantly increased adverse events with HDAC inhibitors in breast cancer [89]. Given this safety profile and limited clinical data on microbiota-derived propionate, dietary modulation represents a more immediate intervention. As Moore et al. demonstrate, caloric restriction reduces pneumonia severity in obese COVID-19 patients [90], supporting nutritional approaches as novel strategies.

Various modes of neutrophil death, including pyroptosis, apoptosis, and NETosis, can impact the severity of both acute and chronic infections [91]. Excessive NET release from neutrophils can exacerbate pneumonia, while promoting neutrophil apoptosis can reduce inflammation [92]. Alsabani et al. studied neutrophil NETs in septic mice and found that inhibiting NETosis reduced lung tissue damage without impairing bacterial clearance [93]. Conus et al. revealed that modulating the normal apoptosis program of neutrophils can affect the duration of innate immunity, thereby affecting the timing of inflammatory remission [94]. Our findings indicate that a high-calorie diet exacerbates pneumonia by inhibiting neutrophil apoptosis and promoting NETs production, but this effect can be reversed by treatment with TSA and propionate. Previous studies have shown that HDACs inhibitors can promote neutrophil apoptosis by enhancing histone acetylation and inhibiting NETs production [48]. Hamam et al. found that HDACs inhibitors dose-dependently shift the mode of neutrophil death from NETs release to apoptosis, potentially due to increased nuclear condensation facilitated by elevated levels of AcH4 in neutrophils [49]. This is supported by our in vivo study, which showed that propionate supplementation increased AcH4 levels, improved neutrophil apoptosis, and decreased NETs levels in neutrophils from the lung tissue of rats with caloric excess. These results suggest that the promotion of histone acetylation may be the mechanism by which propionate reduces neutrophil NETs levels.

In summary, our study demonstrates that a high-calorie diet reduces levels of propionate, a metabolite produced by gut microbiota. This decrease in propionate weakens the inhibition of HDAC 1, 2, 3, and 6, leading to reduced histone acetylation in neutrophils within the lung tissue of juvenile rats with pneumonia. This process promotes the release of NETs and suppresses neutrophil apoptosis, thereby intensifying lung inflammation. Our findings suggest that enhancing the production of SCFAs, particularly propionate, through gut microbiota modulation could be a potential approach for managing pneumonia and other immune-related inflammatory diseases.

## 5. Conclusions

A high-calorie diet exacerbates pneumonia by depleting gut-derived propionate, which drives HDAC-mediated NETs overproduction and impairs neutrophil apoptosis. Re-storing propionate levels or targeting HDACs may offer therapeutic strategies for di-et-aggravated respiratory diseases. Mechanistically, propionate-mediated HDAC inhibition demonstrates proof-of-concept efficacy in modulating H4 acetylation, warranting further investigation in disease-specific pneumonia models.

## Figures and Tables

**Table 1:** Experiment protocol.

Group	*n*	Feed (1–6 Days)	Atomization (4–6 Days)
N	10	Rat standard maintenance fodder	Physiologic saline
G	10	High-calorie fodder	Physiologic saline
P	10	Rat standard maintenance fodder	LPS solution
GP	10	High-calorie fodder	LPS solution

**Table 2:** Primer sequences.

Gene	Primer	Sequence
HDAC1	Forward Reverse	5′-TTCCAACATGACCAACCAGA-3′ 5′- ACCACCTTCTCCCTCCTCAT-3′
HDAC2	Forward Reverse	5′-ACCCGGACAAAAGAATTTCC-3′ 5′-TTGGGGTCTGTTTTCTCACC-3′
HDAC3	Forward Reverse	5′-AATGTGCCCTTACGAGATGG-3′ 5′-GTAGCCACCACCTCCCAGTA-3′
HDAC6	Forward Reverse	5′-CTGGCTAAGGGAGTCAGTGC-3′ 5′-TAGCACGGCTTCTTCCACTT-3′

## Data Availability

The data are available from Mr. Tiegang Liu and Ms. Xiaohong Gu.

## Supplementary Materials

The following supporting information can be downloaded at https://www.mdpi.com/article/10.3390/nu17132242/s1.

## Abbreviations

NETs	neutrophil extracellular traps
SCFAs	short-chain fatty acids
LPS	Lipopolysaccharide
HDAC	histone deacetylase
GPCR	G protein-coupled receptors
H&E	hematoxylin and eosin
IL-1β	interleukin-1 beta
IL-6	interleukin-6
TNF-α	tumor necrosis factor-alpha
TSA	trichostatin A
ELISA	enzyme-linked immunosorbent assay
cDNA	complementary DNA
GAPDH	glyceraldehyde 3-phosphate dehydrogenase
GC-MS	gas chromatography-mass spectrometry
OCT	optimal cutting temperature
DAPI	4′,6-diamidino-2-phenylindole
TUNEL	terminal deoxynucleotidyl transferase dUTP nick-end labeling
CXCL1	C-X-C Motif Chemokine Ligand 1
NF-κB	nuclear factor-κB
