# Supplementary figures and images for: High-Calorie Diets Exacerbate Lipopolysaccharide-Induced Pneumonia by Promoting Propionate-Mediated Neutrophil Extracellular Traps (part 2 of 2)
Source: Nutrients. 2025 Jul 7;17(13):2242. doi: 10.3390/nu17132242 (PMC12251575; doi:10.3390/nu17132242)

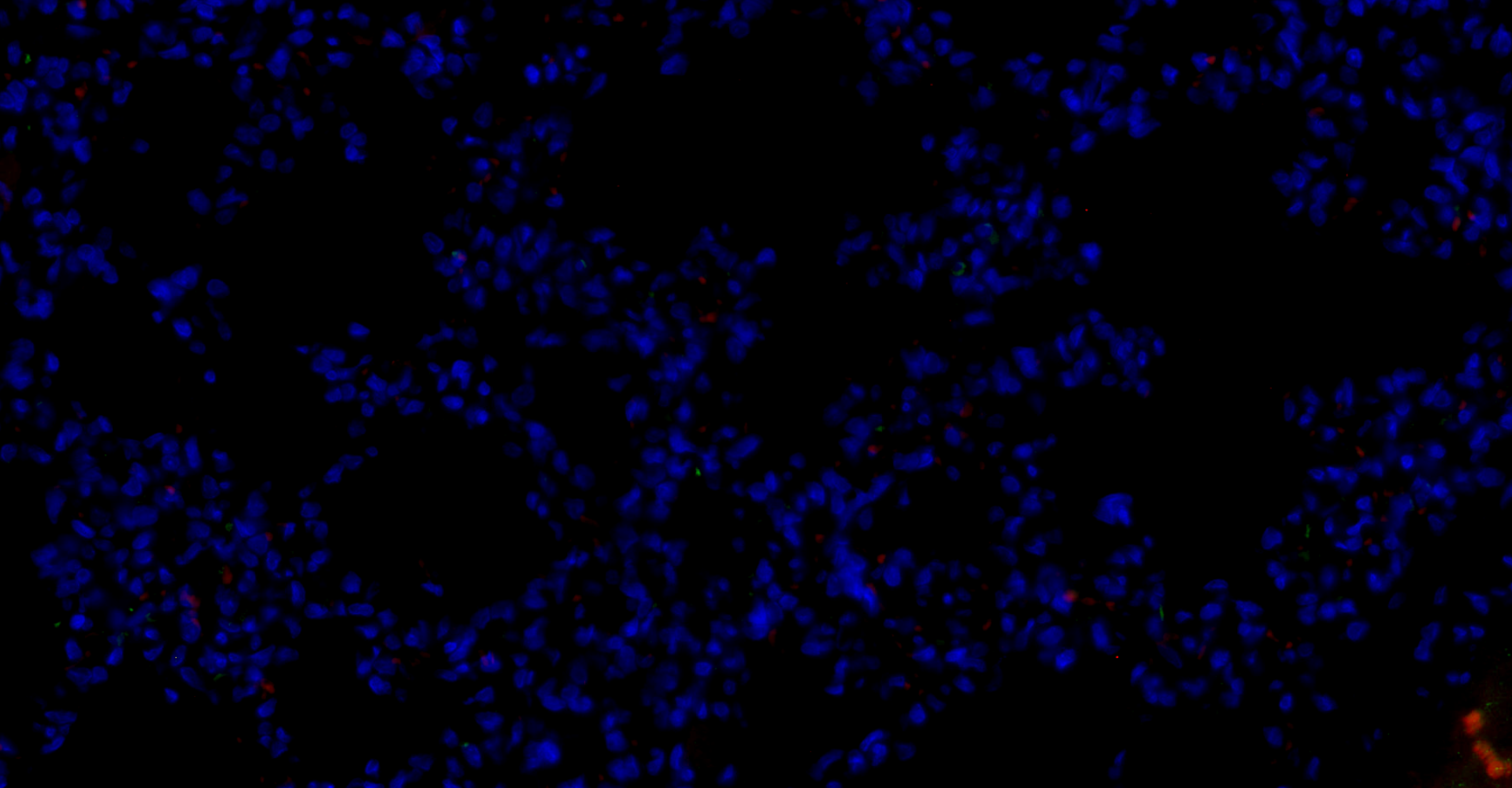

Supplement: Supplementary file 1 [file nutrients-17-02242-s001.zip › Figure S2 Original images/figure6-N-4 LY6G-CITH3_40.0x.tif]

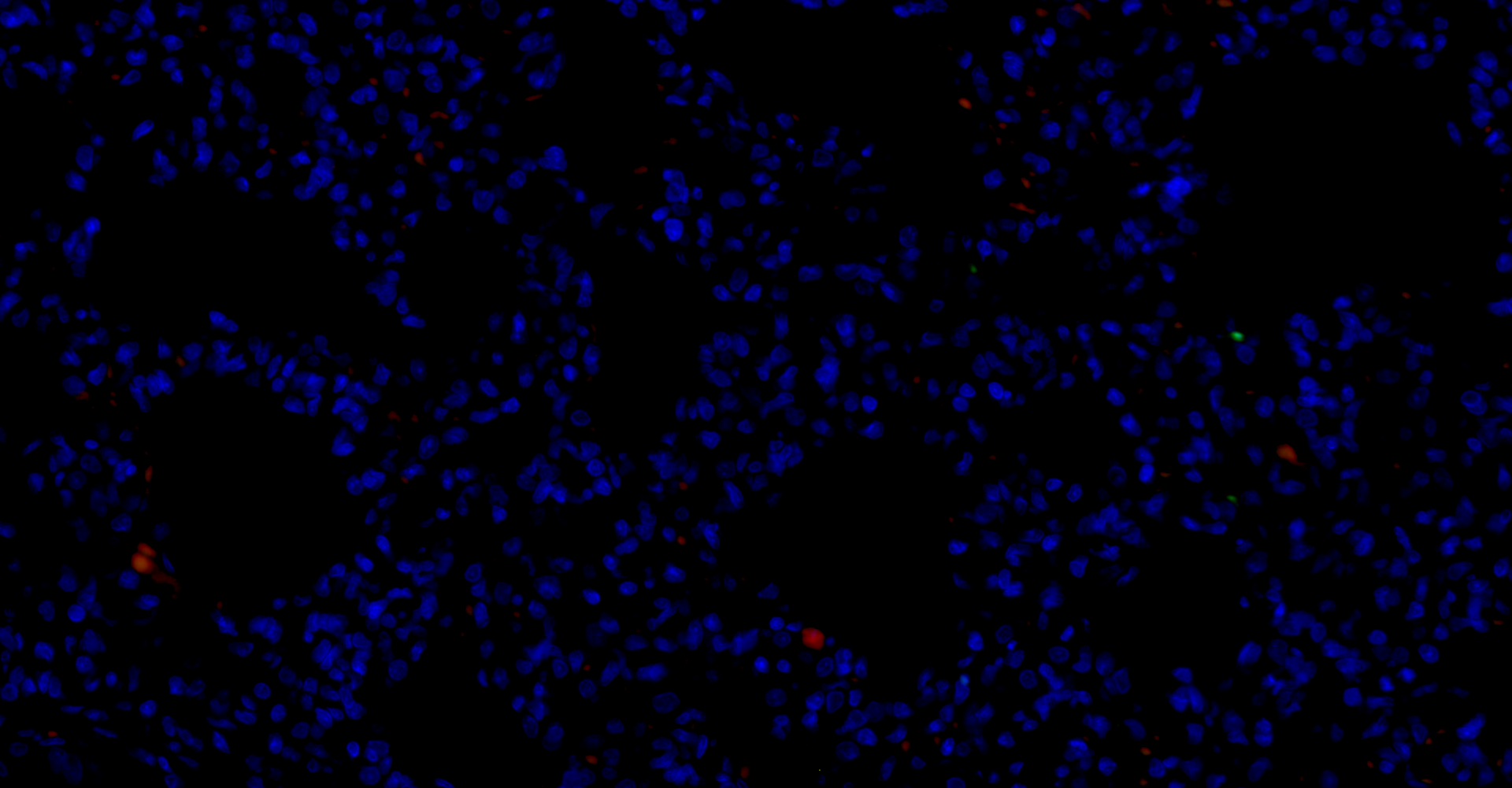

Supplement: Supplementary file 1 [file nutrients-17-02242-s001.zip › Figure S2 Original images/figure6-N-4 TUNEL-LY6G_40.0x.tif]

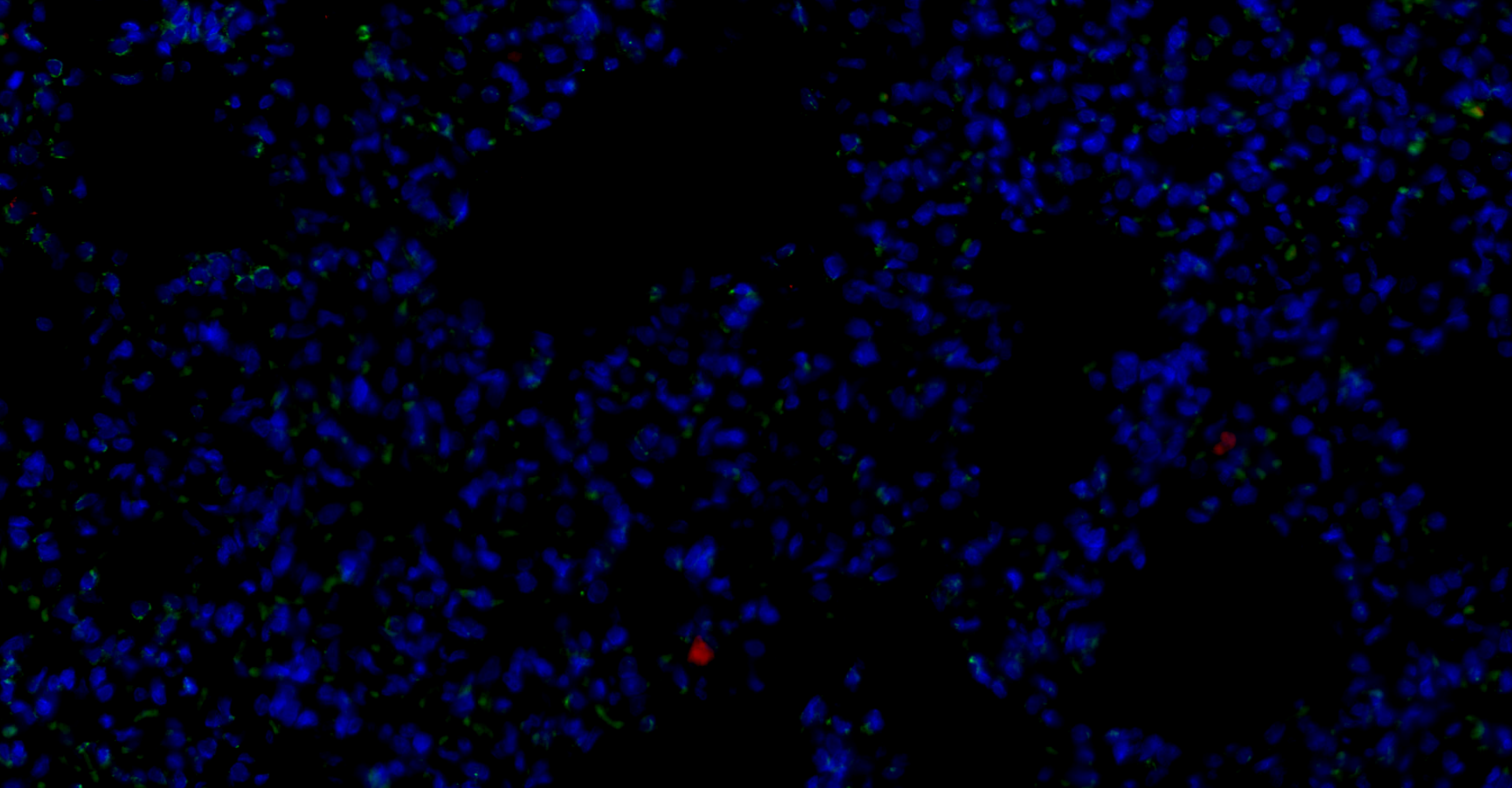

Supplement: Supplementary file 1 [file nutrients-17-02242-s001.zip › Figure S2 Original images/figure6-N-5 LY6G-ACH4_40.0x.tif]

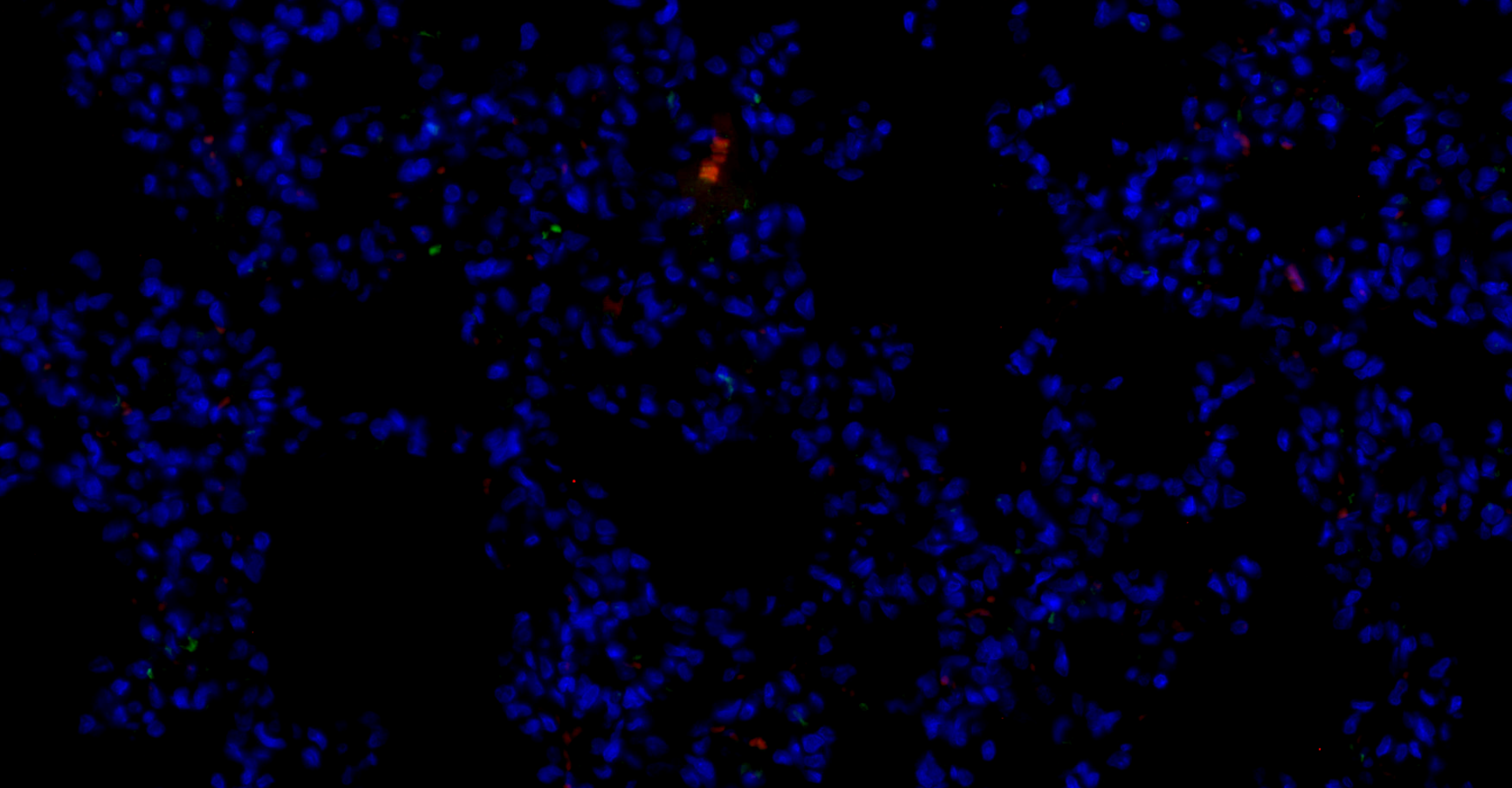

Supplement: Supplementary file 1 [file nutrients-17-02242-s001.zip › Figure S2 Original images/figure6-N-5 LY6G-CITH3_40.0x.tif]

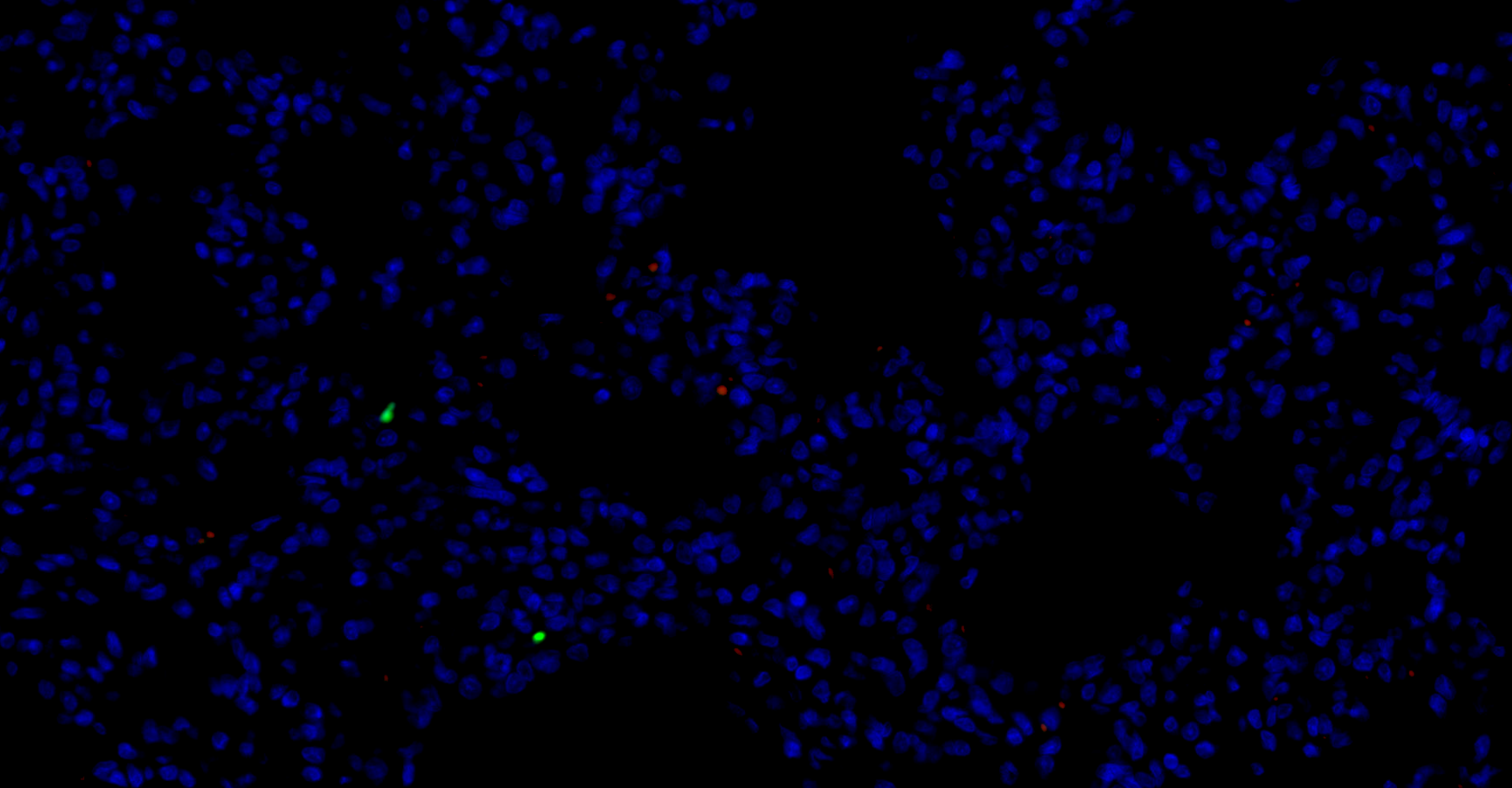

Supplement: Supplementary file 1 [file nutrients-17-02242-s001.zip › Figure S2 Original images/figure6-N-5 TUNEL-LY6G_40.0x.tif]

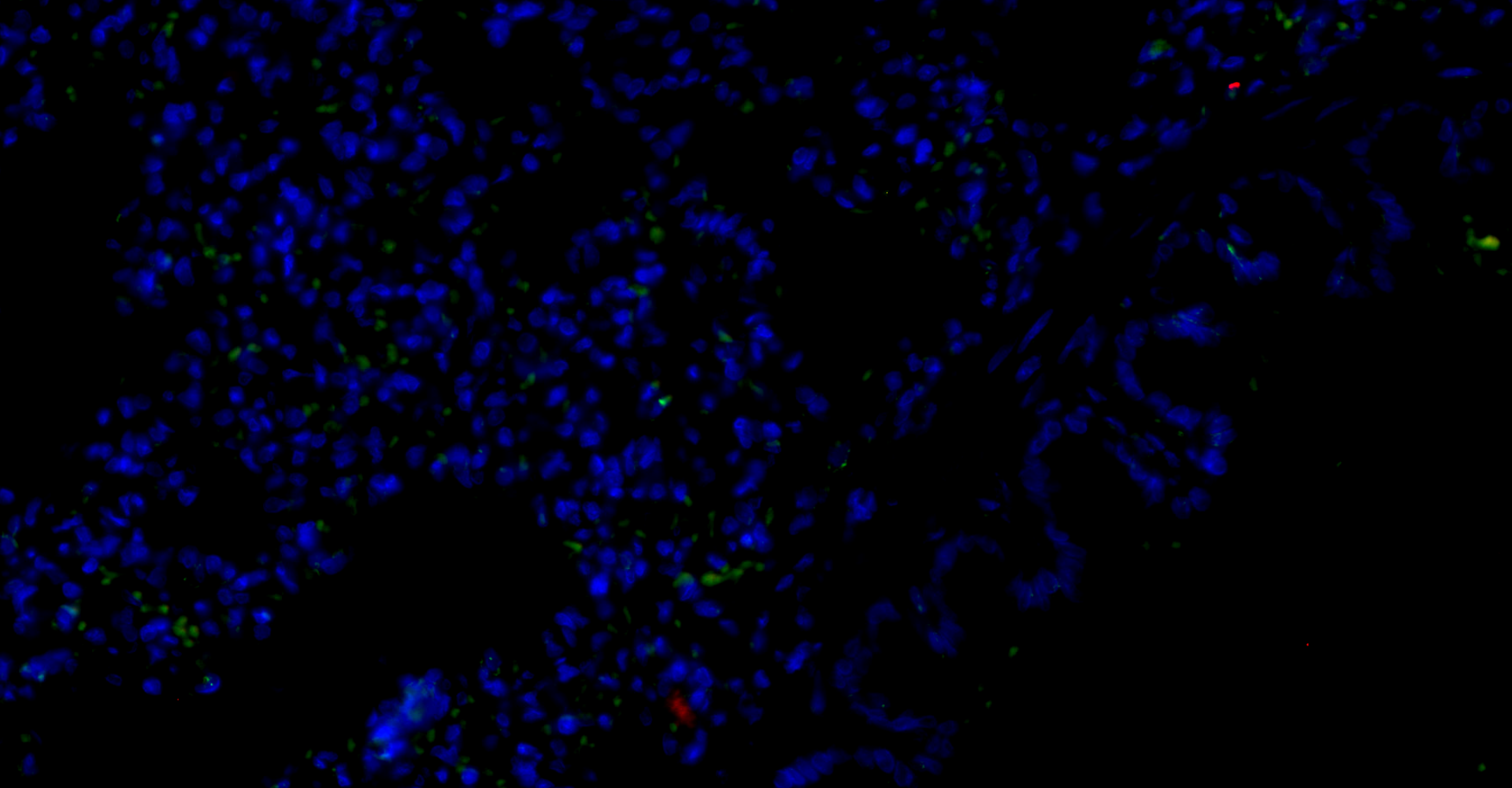

Supplement: Supplementary file 1 [file nutrients-17-02242-s001.zip › Figure S2 Original images/figure6-N-6 LY6G-ACH4_40.0x.tif]

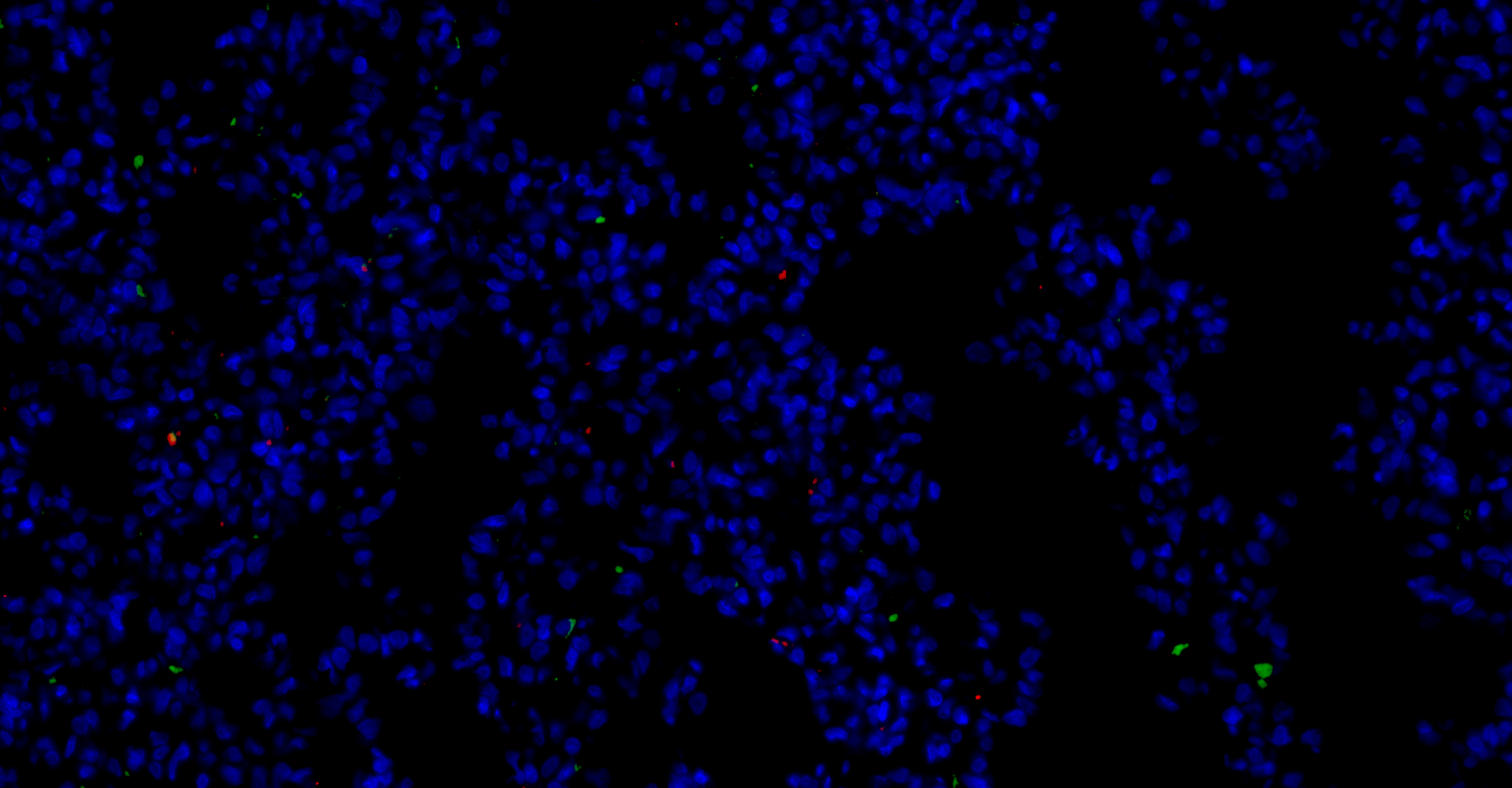

Supplement: Supplementary file 1 [file nutrients-17-02242-s001.zip › Figure S2 Original images/figure6-N-6 LY6G-CITH3_40.0x.tif]

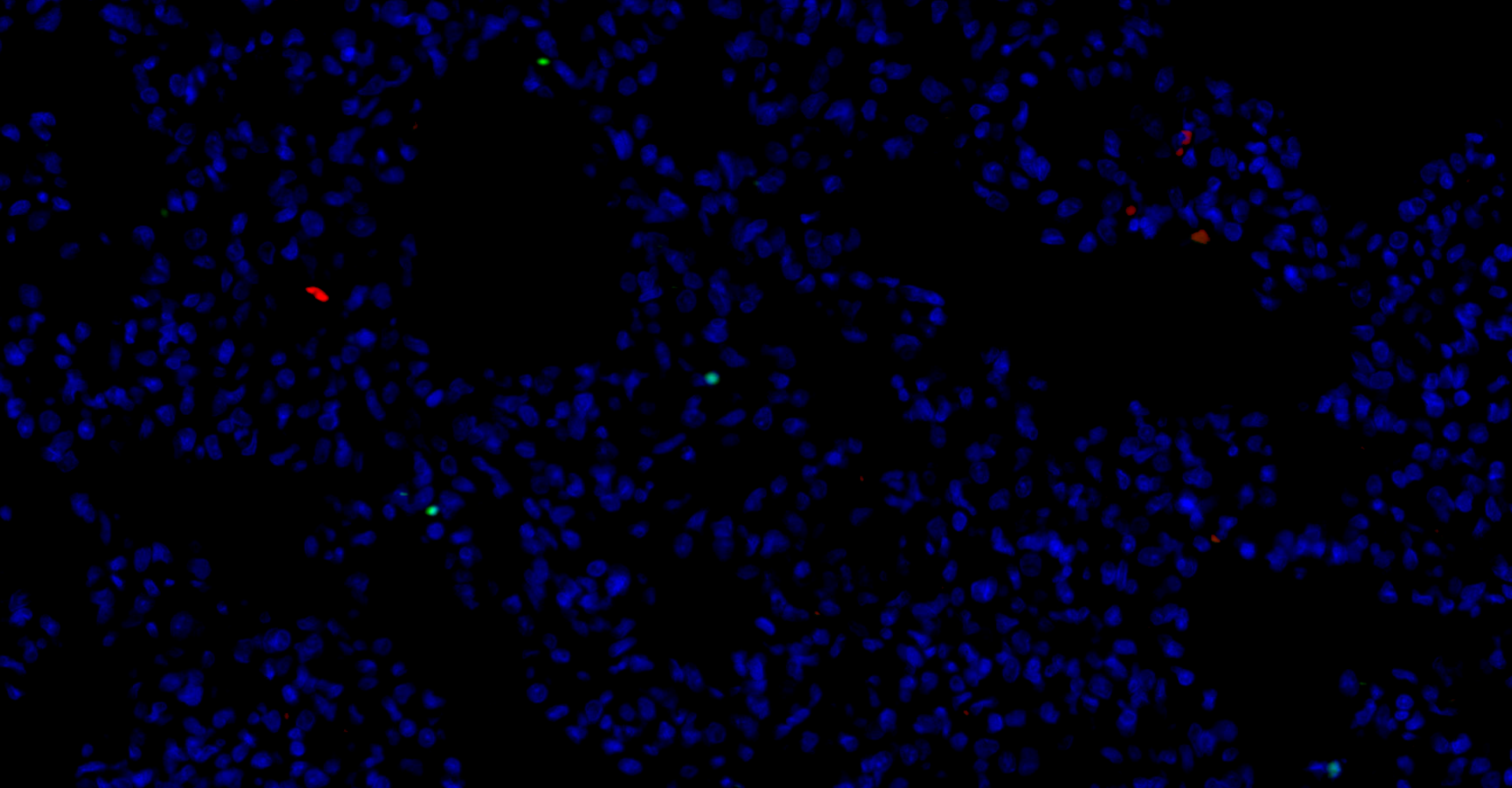

Supplement: Supplementary file 1 [file nutrients-17-02242-s001.zip › Figure S2 Original images/figure6-N-6 TUNEL-LY6G_40.0x.tif]

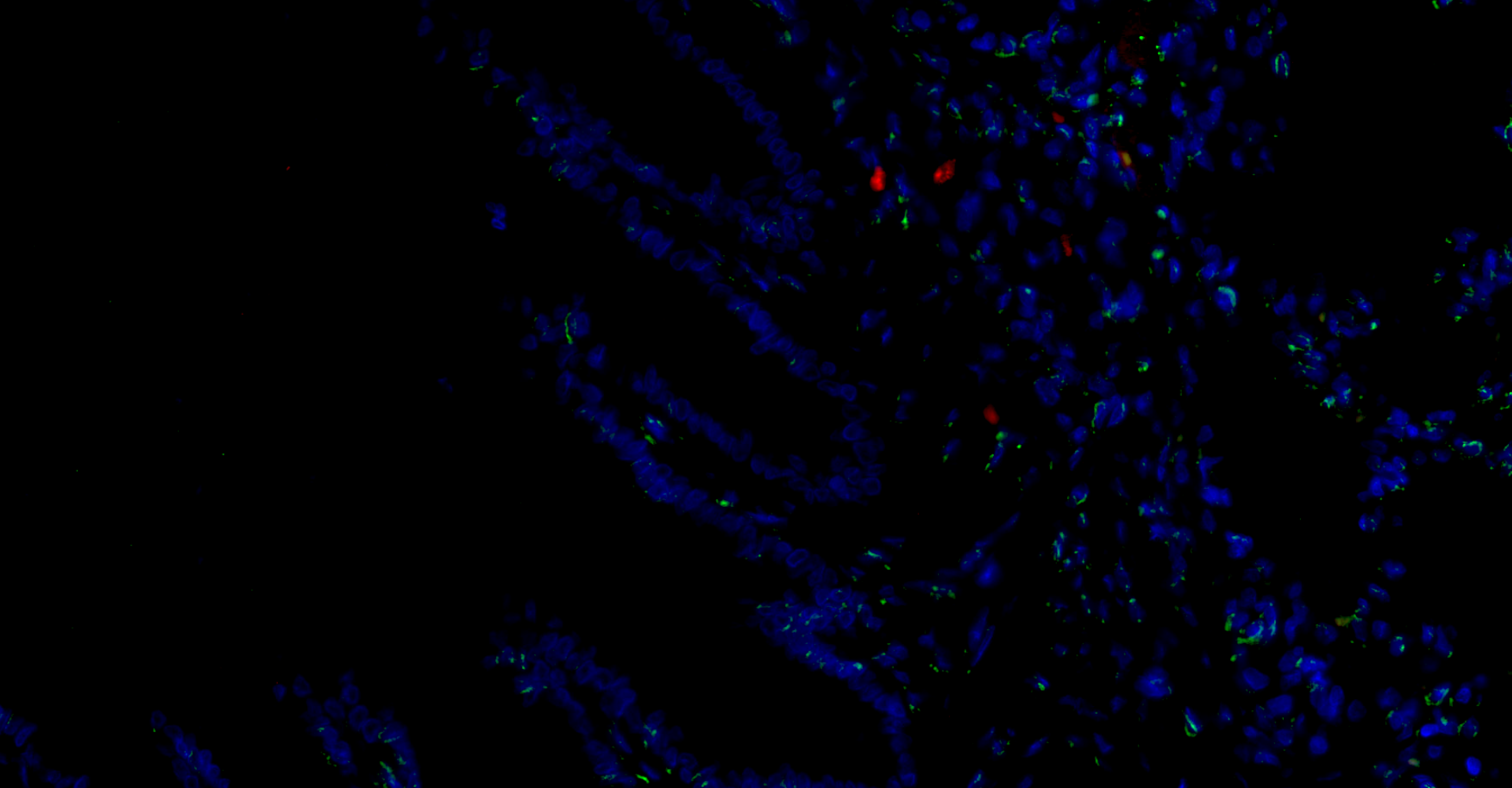

Supplement: Supplementary file 1 [file nutrients-17-02242-s001.zip › Figure S2 Original images/figure6-P-1 LY6G-ACH4_40.0x.tif]

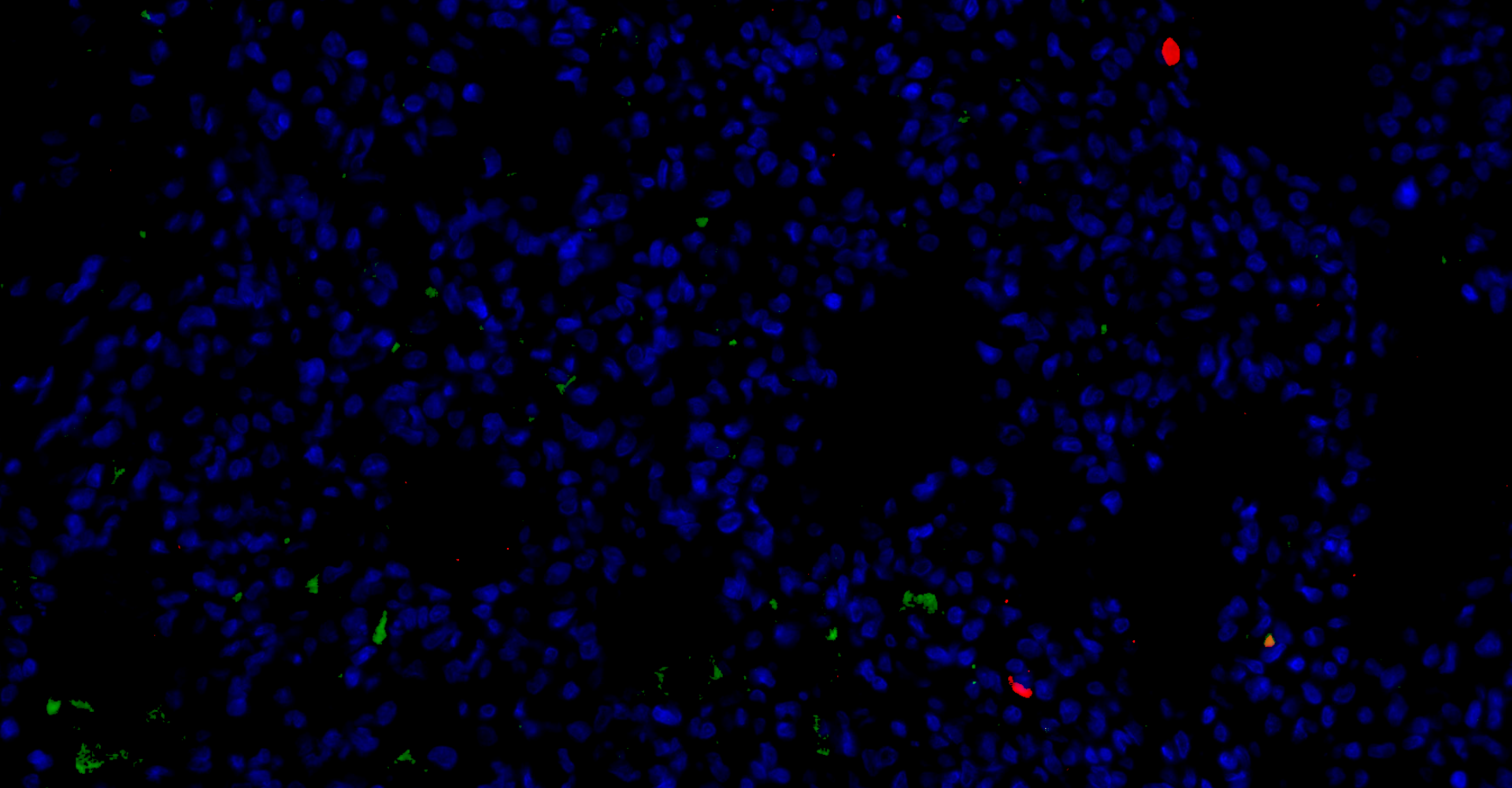

Supplement: Supplementary file 1 [file nutrients-17-02242-s001.zip › Figure S2 Original images/figure6-P-1 LY6G-CITH3_40.0x.tif]

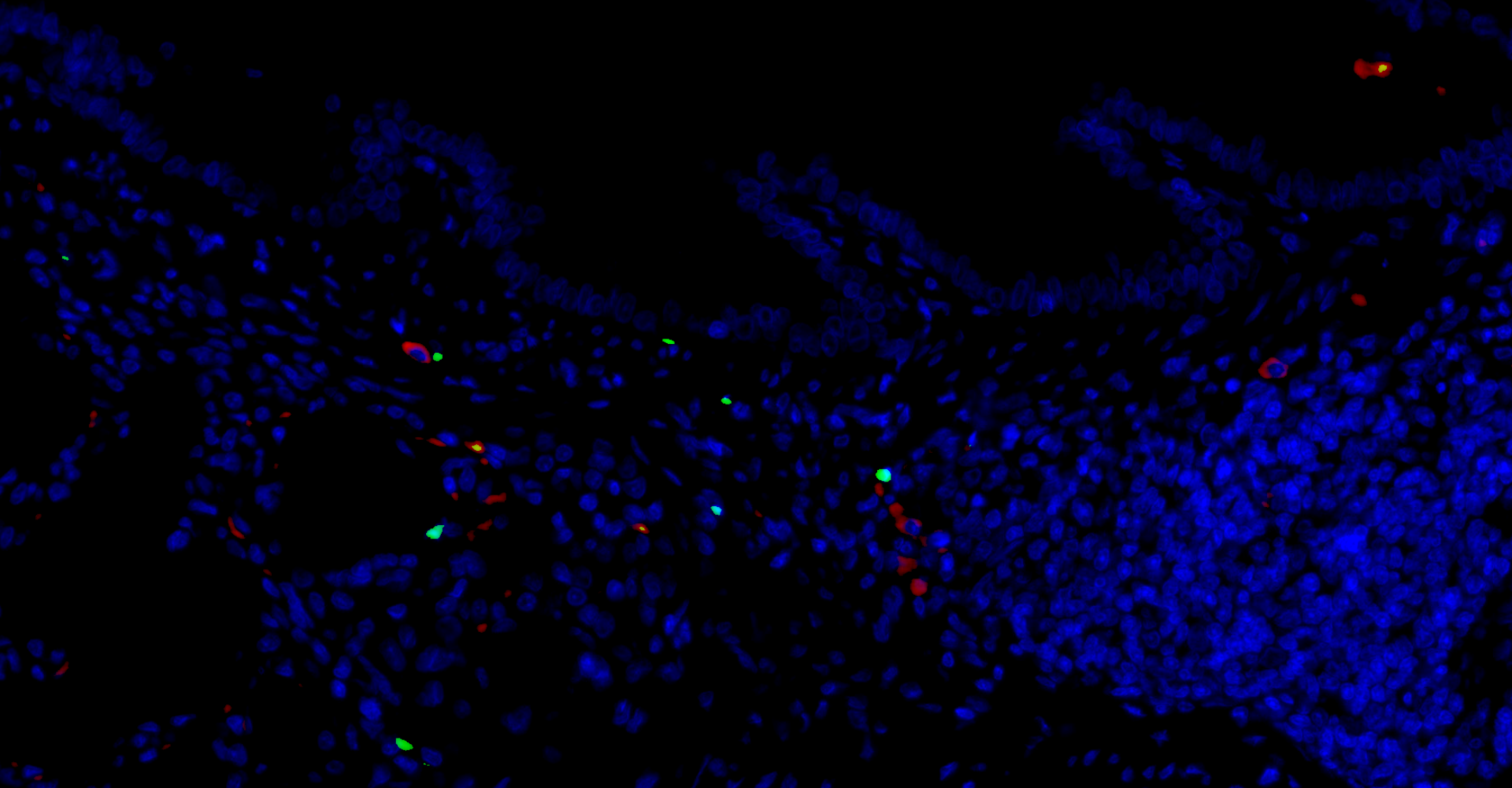

Supplement: Supplementary file 1 [file nutrients-17-02242-s001.zip › Figure S2 Original images/figure6-P-1 TUNEL-LY6G_40.0x.tif]

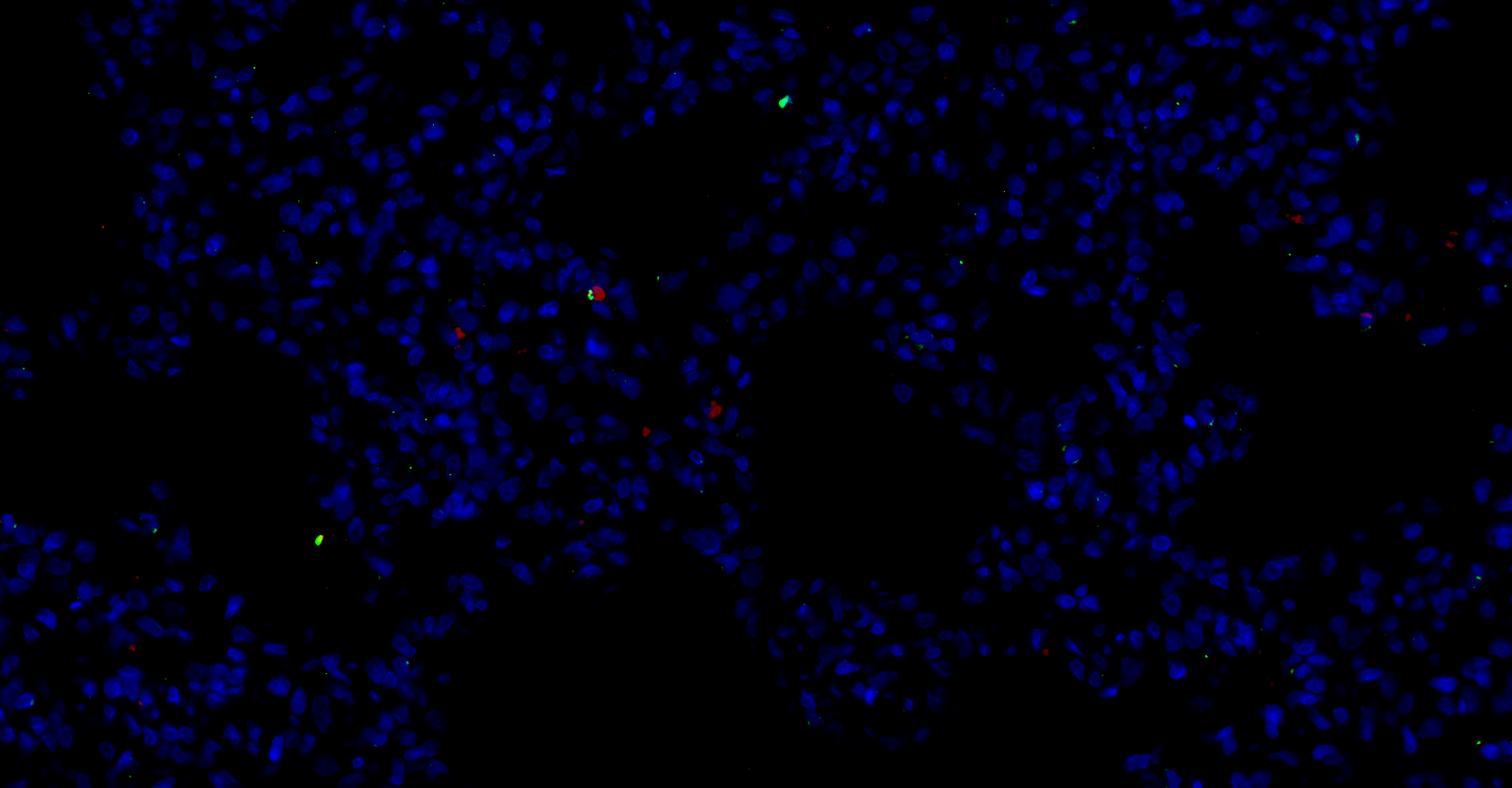

Supplement: Supplementary file 1 [file nutrients-17-02242-s001.zip › Figure S2 Original images/figure6-P-2 LY6G-ACH4_40.0x.tif]

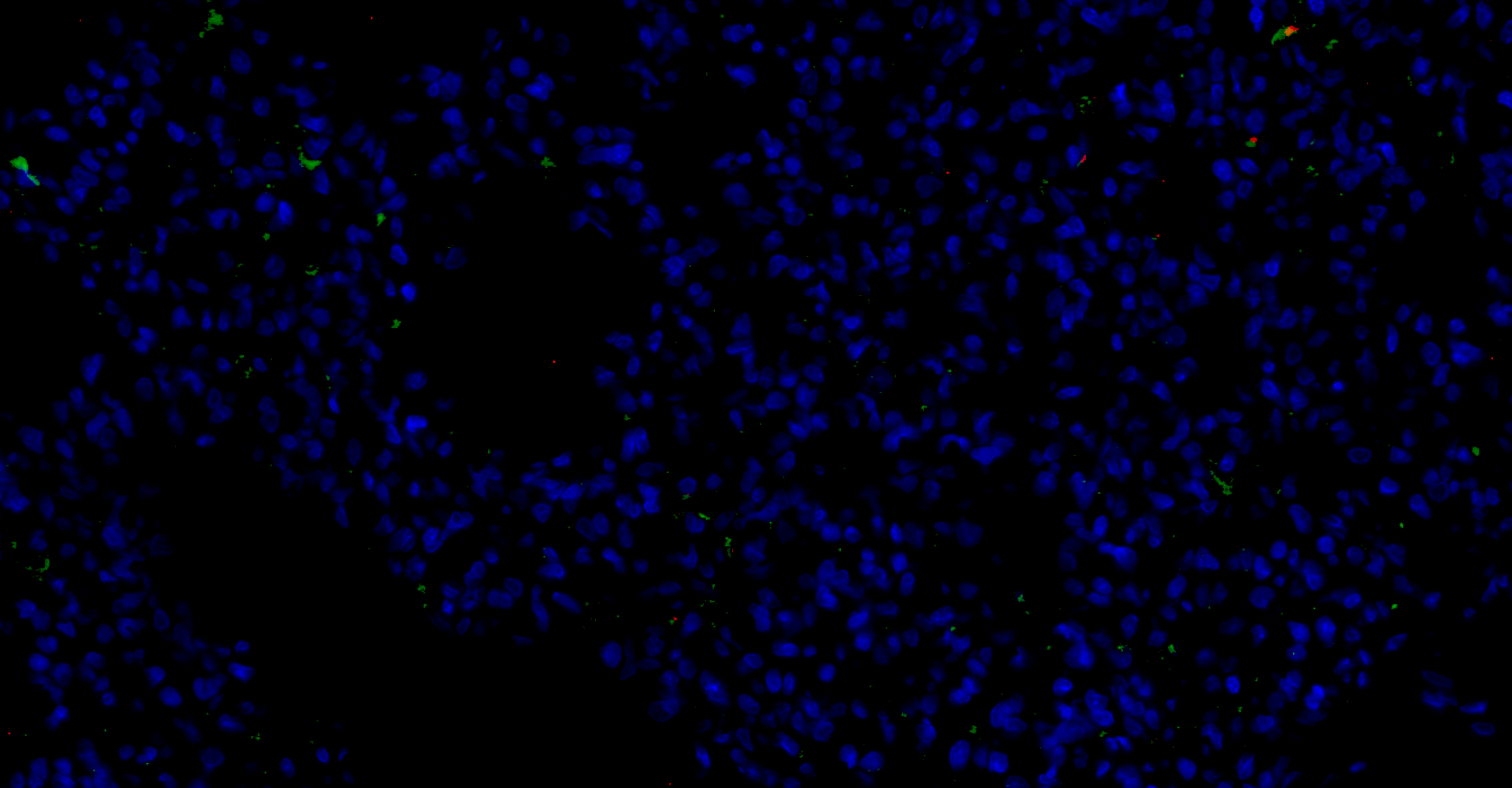

Supplement: Supplementary file 1 [file nutrients-17-02242-s001.zip › Figure S2 Original images/figure6-P-2 LY6G-CITH3_40.0x.tif]

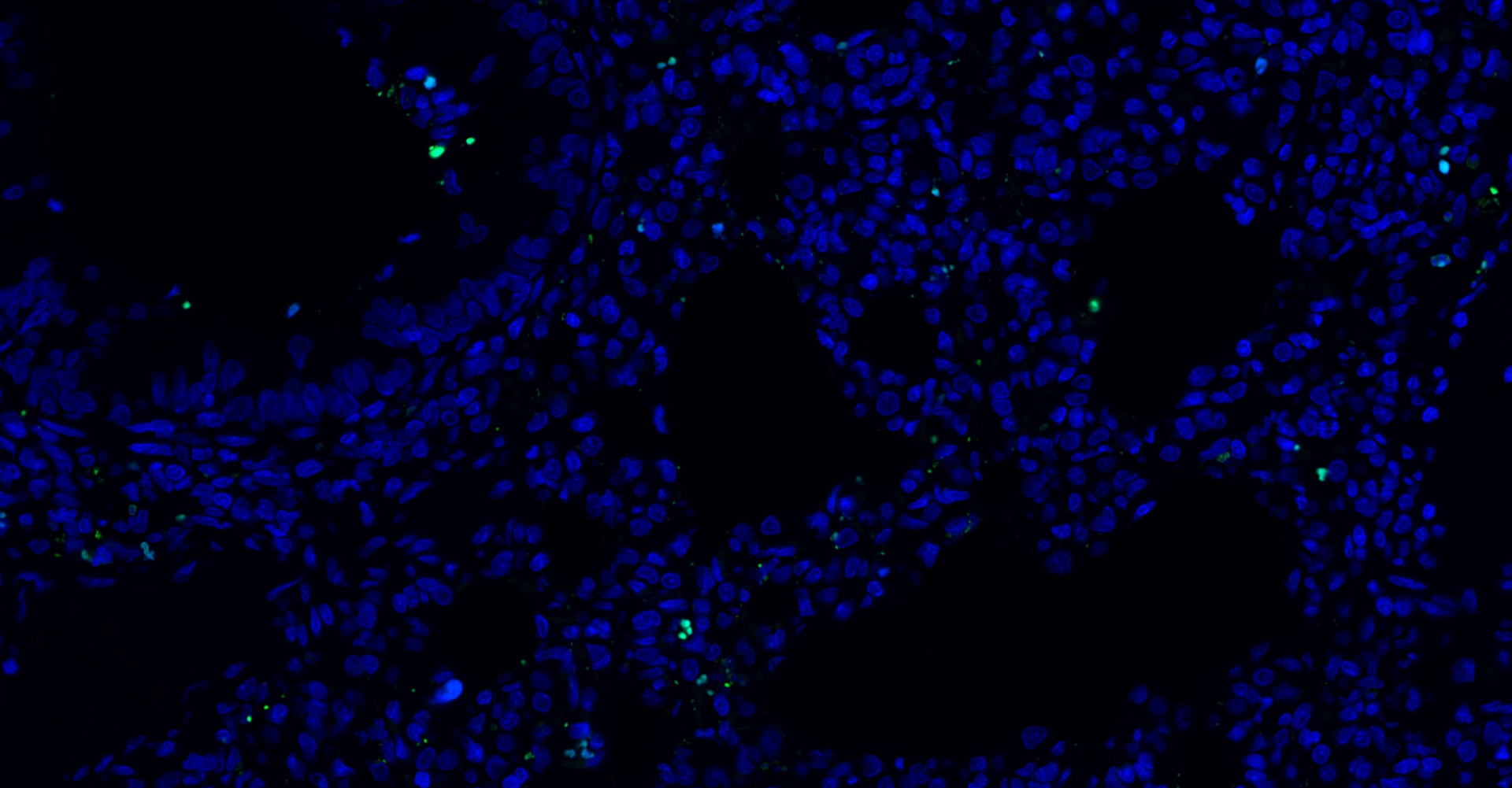

Supplement: Supplementary file 1 [file nutrients-17-02242-s001.zip › Figure S2 Original images/figure6-P-2 TUNEL-LY6G_40.0x.tif]

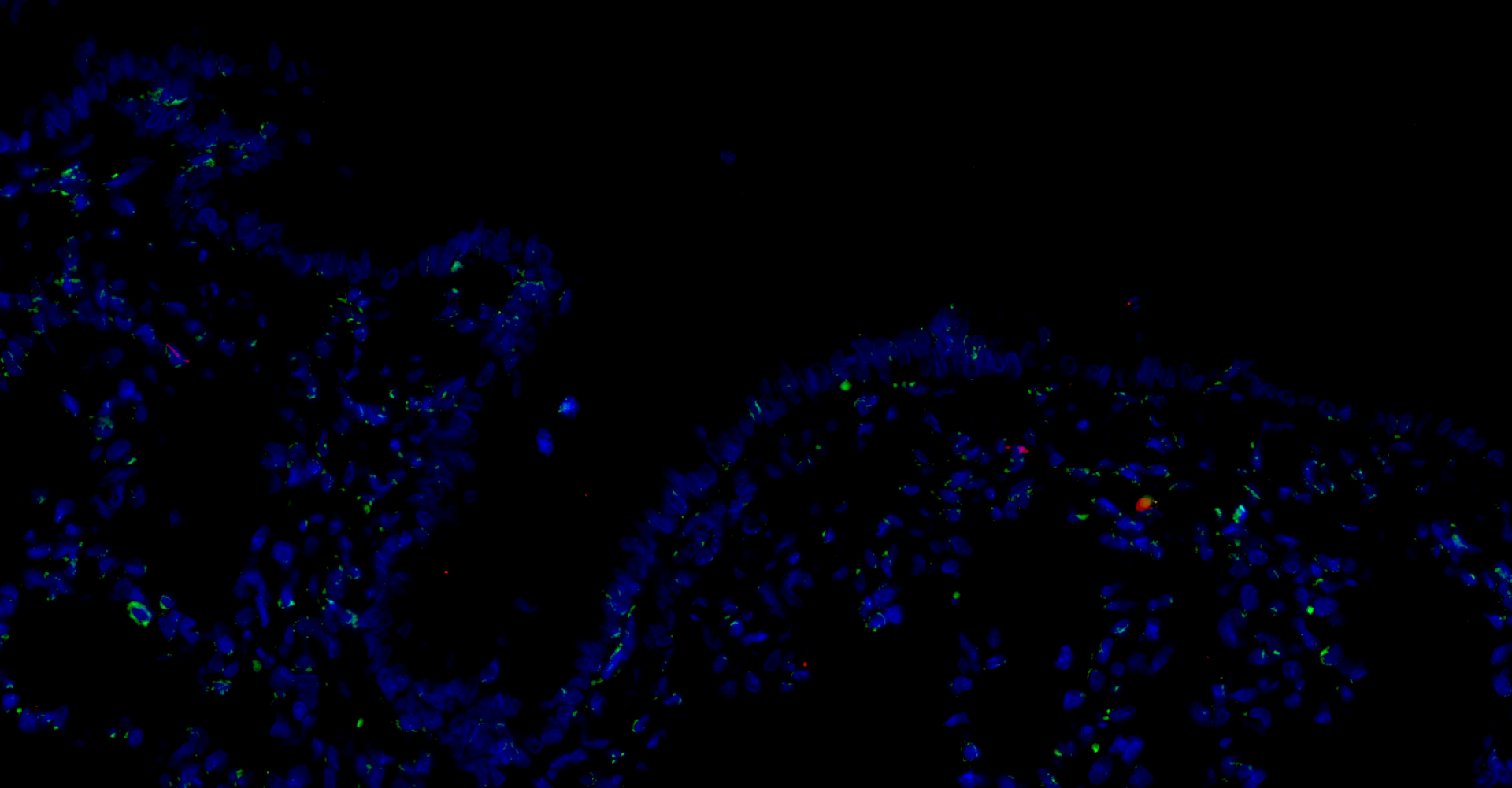

Supplement: Supplementary file 1 [file nutrients-17-02242-s001.zip › Figure S2 Original images/figure6-P-3 LY6G-ACH4_40.0x.tif]

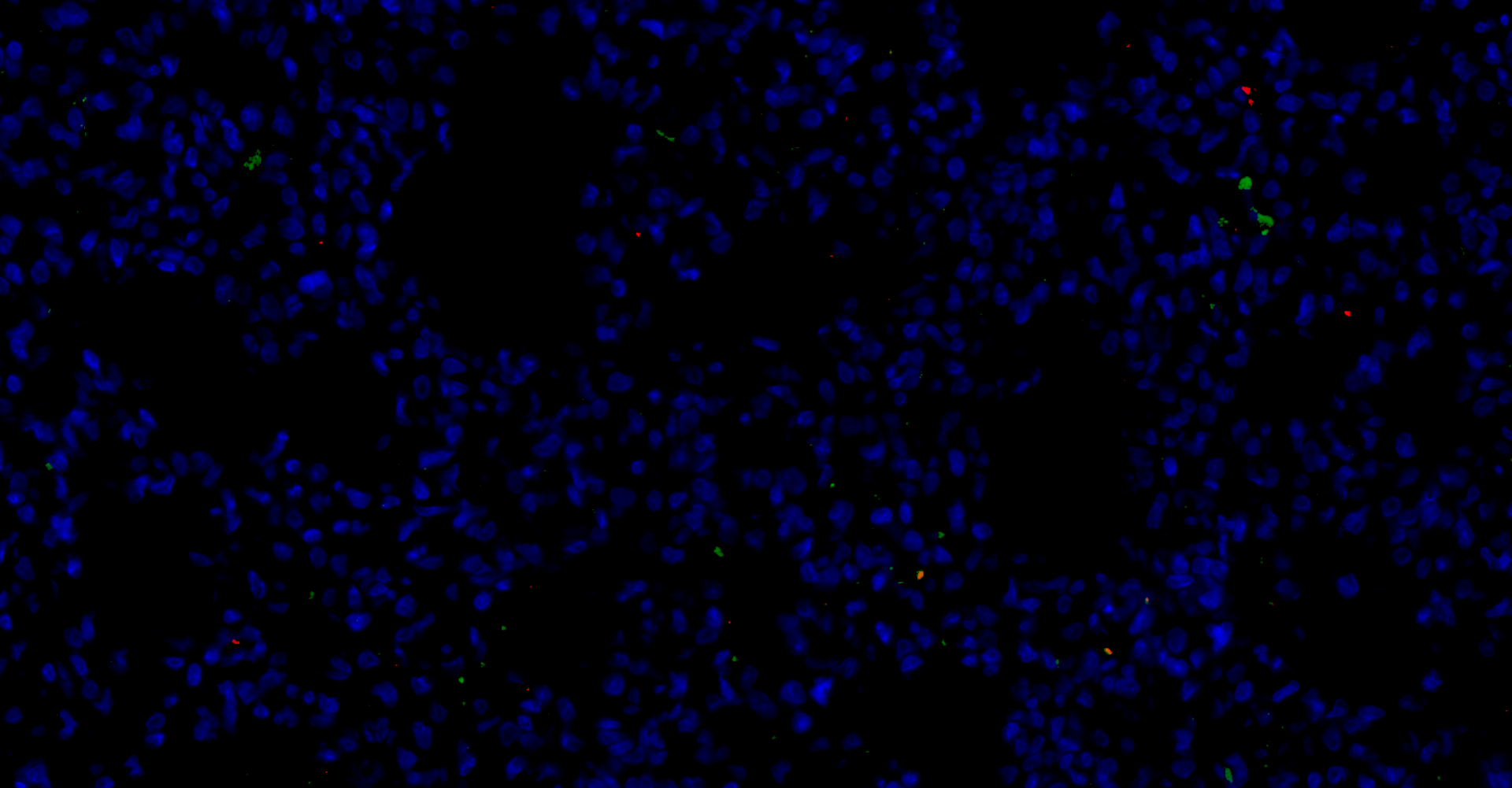

Supplement: Supplementary file 1 [file nutrients-17-02242-s001.zip › Figure S2 Original images/figure6-P-3 LY6G-CITH3_40.0x.tif]

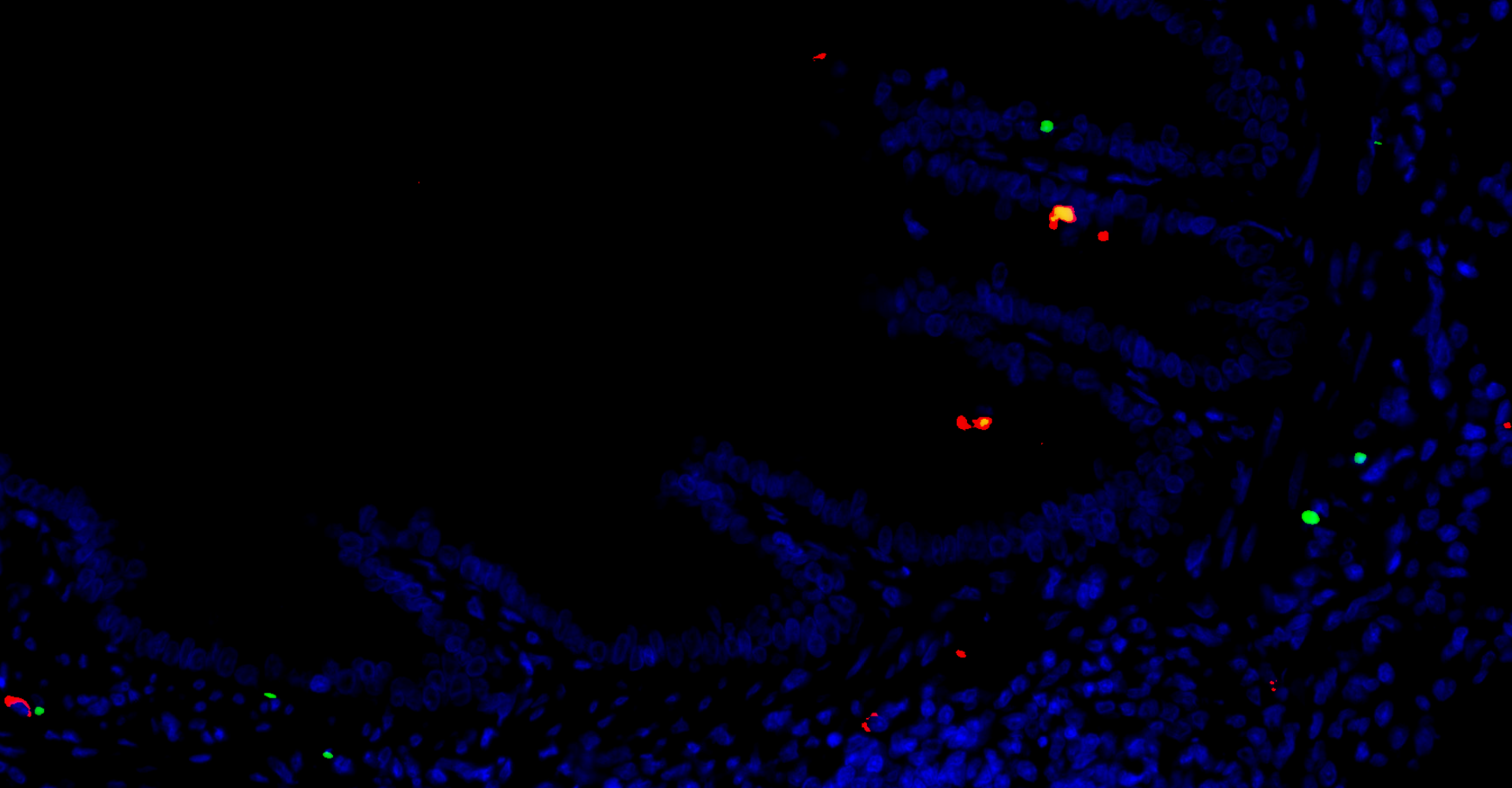

Supplement: Supplementary file 1 [file nutrients-17-02242-s001.zip › Figure S2 Original images/figure6-P-3 TUNEL-LY6G_40.0x.tif]

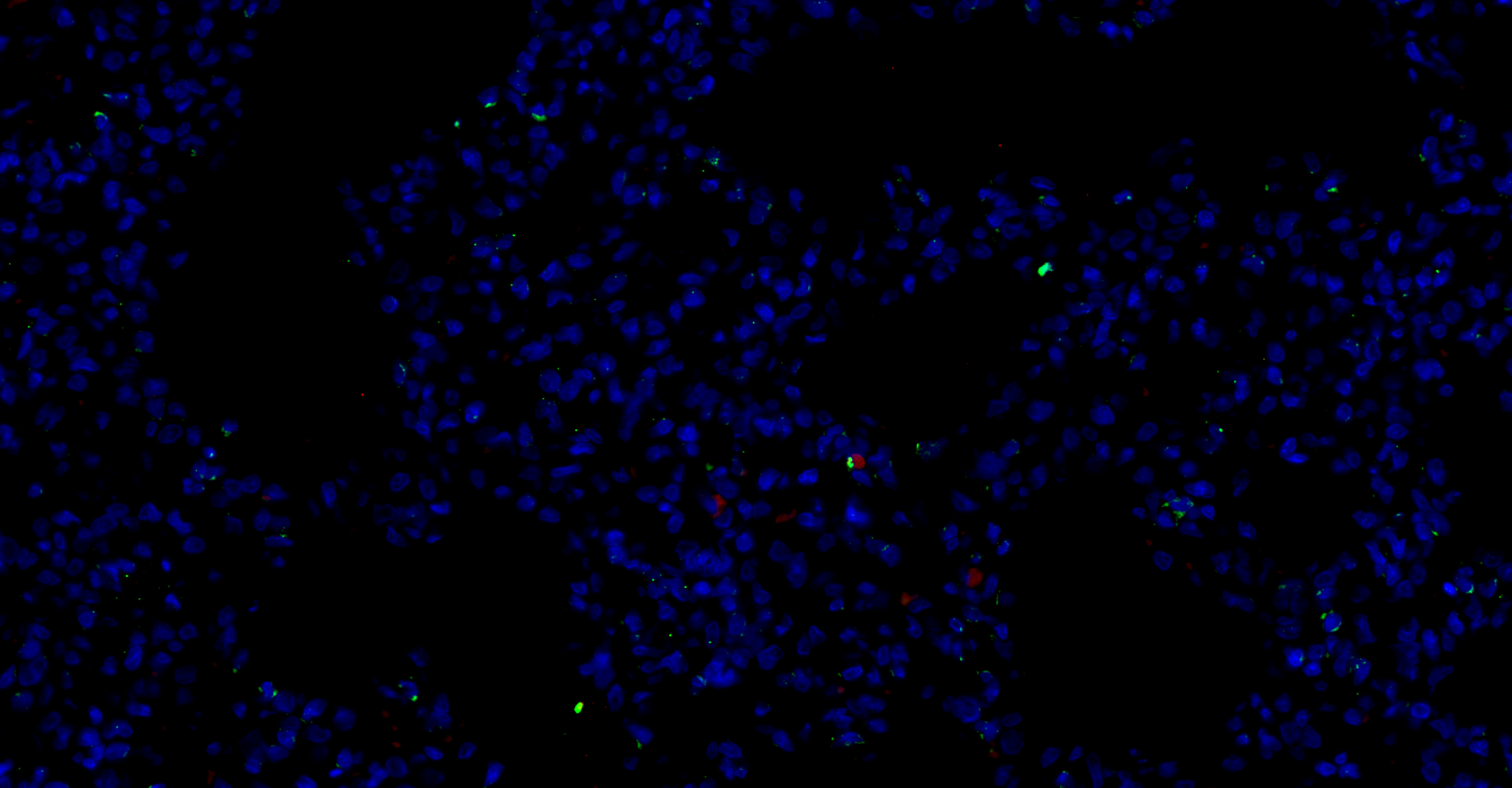

Supplement: Supplementary file 1 [file nutrients-17-02242-s001.zip › Figure S2 Original images/figure6-P-4 LY6G-ACH4_40.0x.tif]

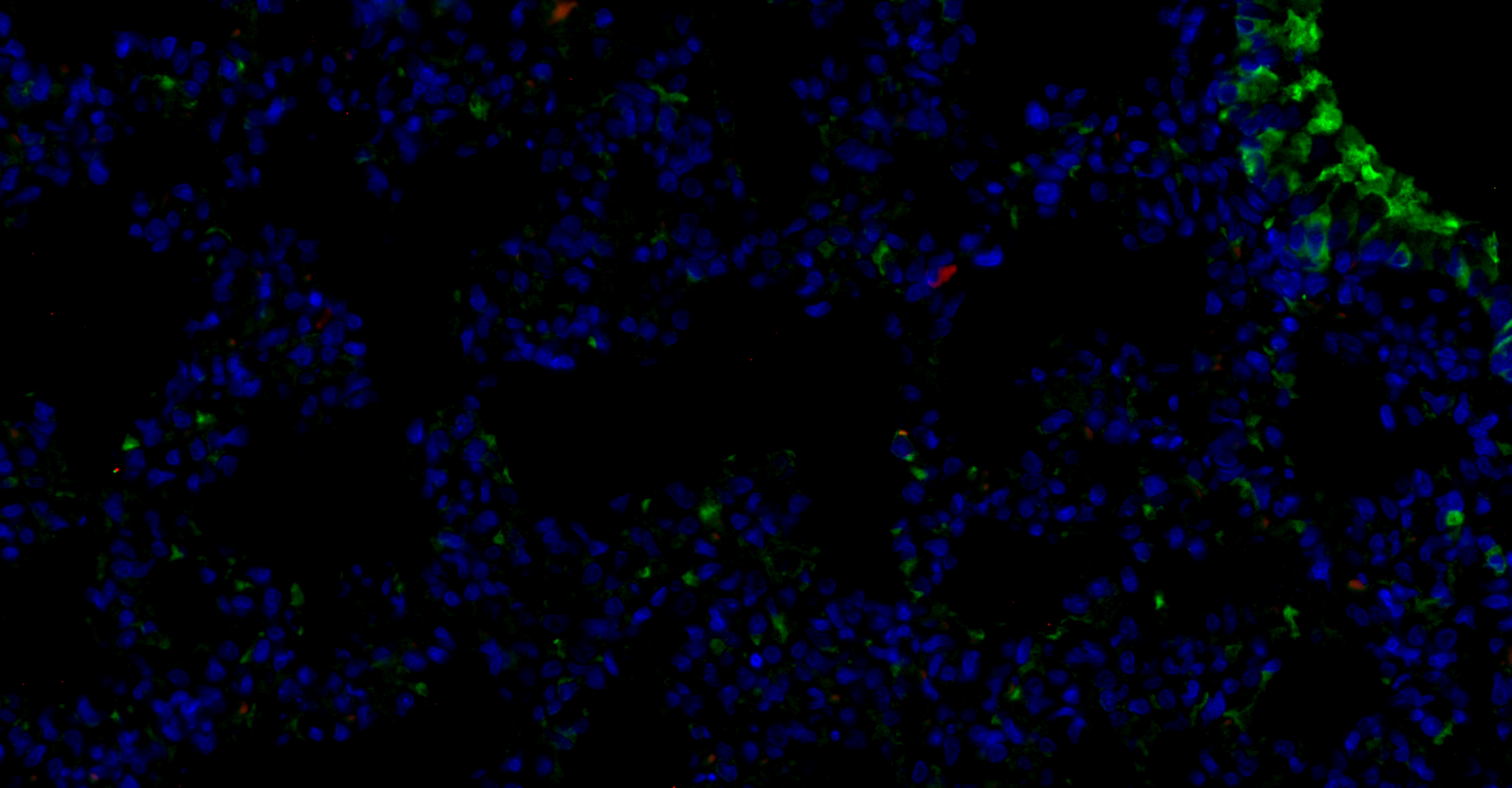

Supplement: Supplementary file 1 [file nutrients-17-02242-s001.zip › Figure S2 Original images/figure6-P-4 LY6G-CITH3_40.0x.tif]

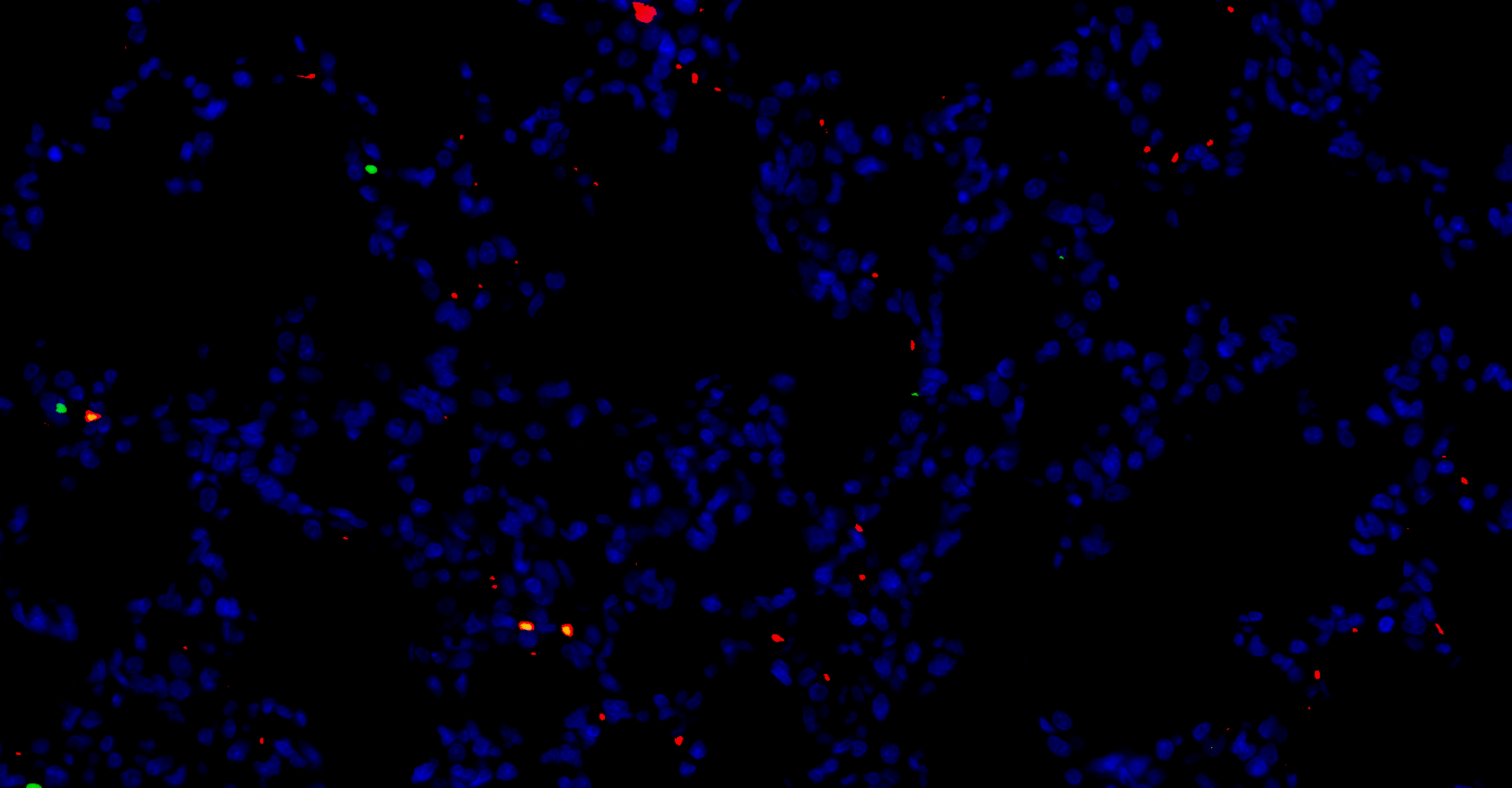

Supplement: Supplementary file 1 [file nutrients-17-02242-s001.zip › Figure S2 Original images/figure6-P-4 TUNEL-LY6G_40.0x.tif]

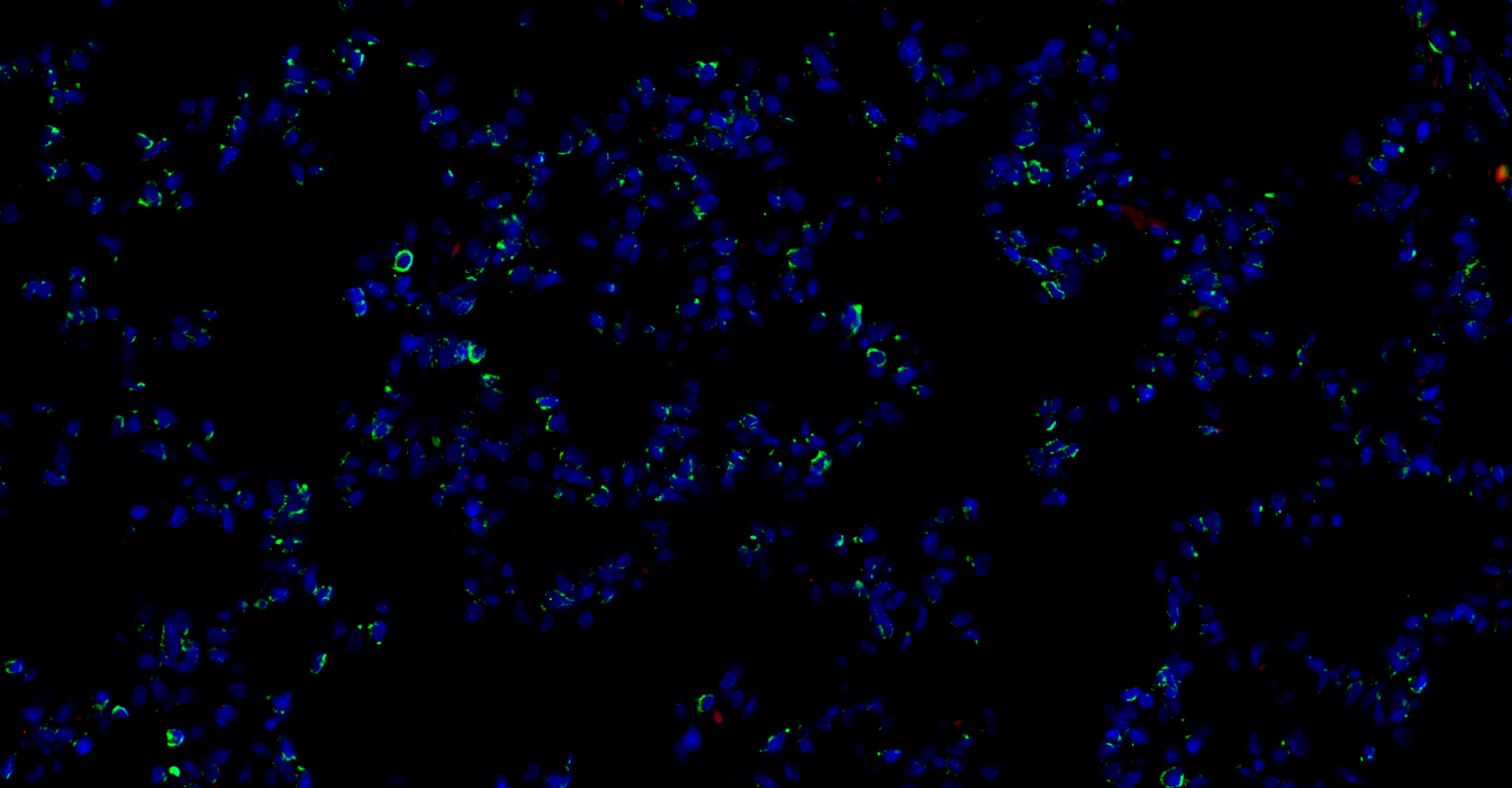

Supplement: Supplementary file 1 [file nutrients-17-02242-s001.zip › Figure S2 Original images/figure6-P-5 LY6G-ACH4_40.0x.tif]

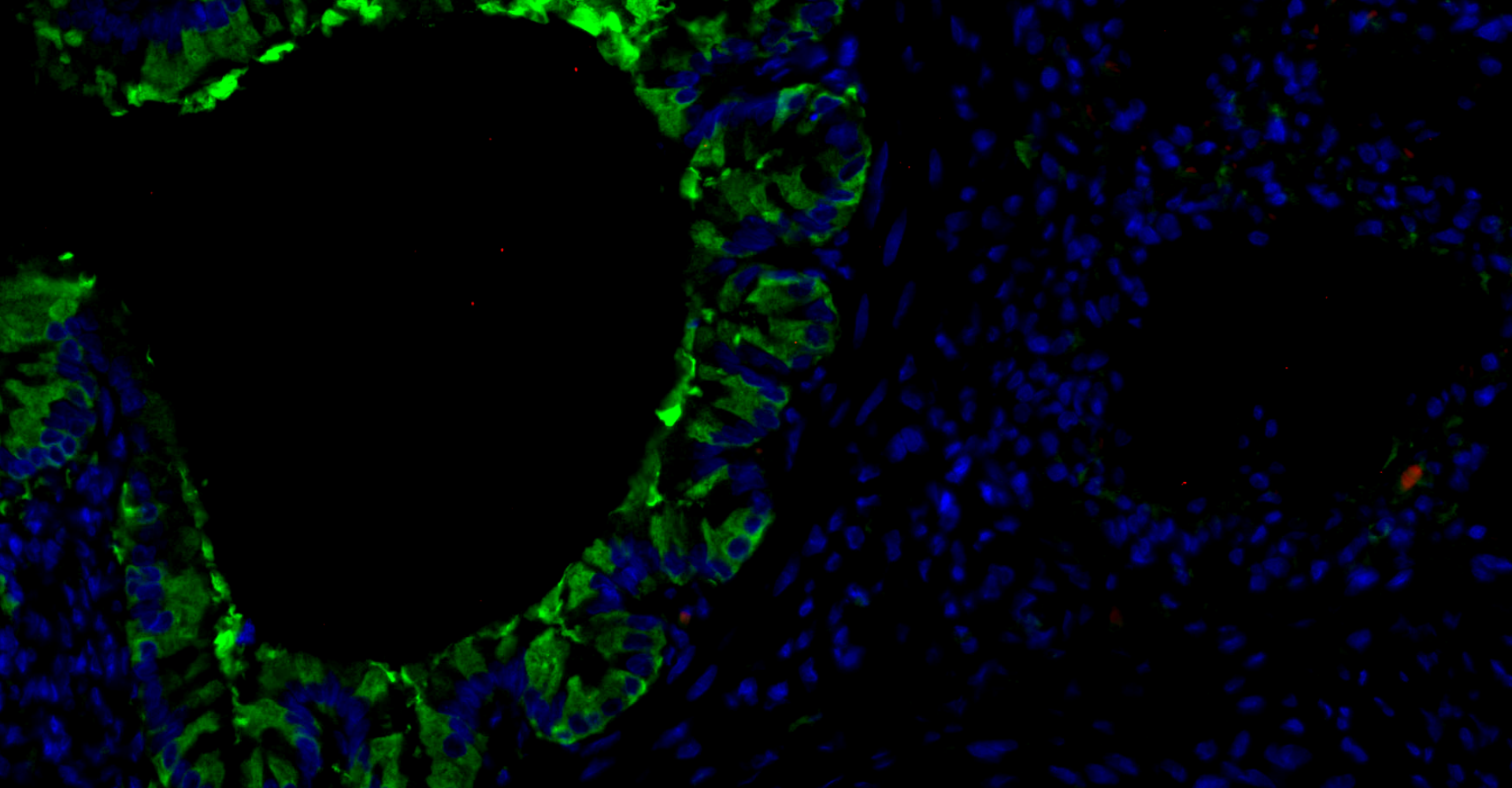

Supplement: Supplementary file 1 [file nutrients-17-02242-s001.zip › Figure S2 Original images/figure6-P-5 LY6G-CITH3_40.0x.tif]

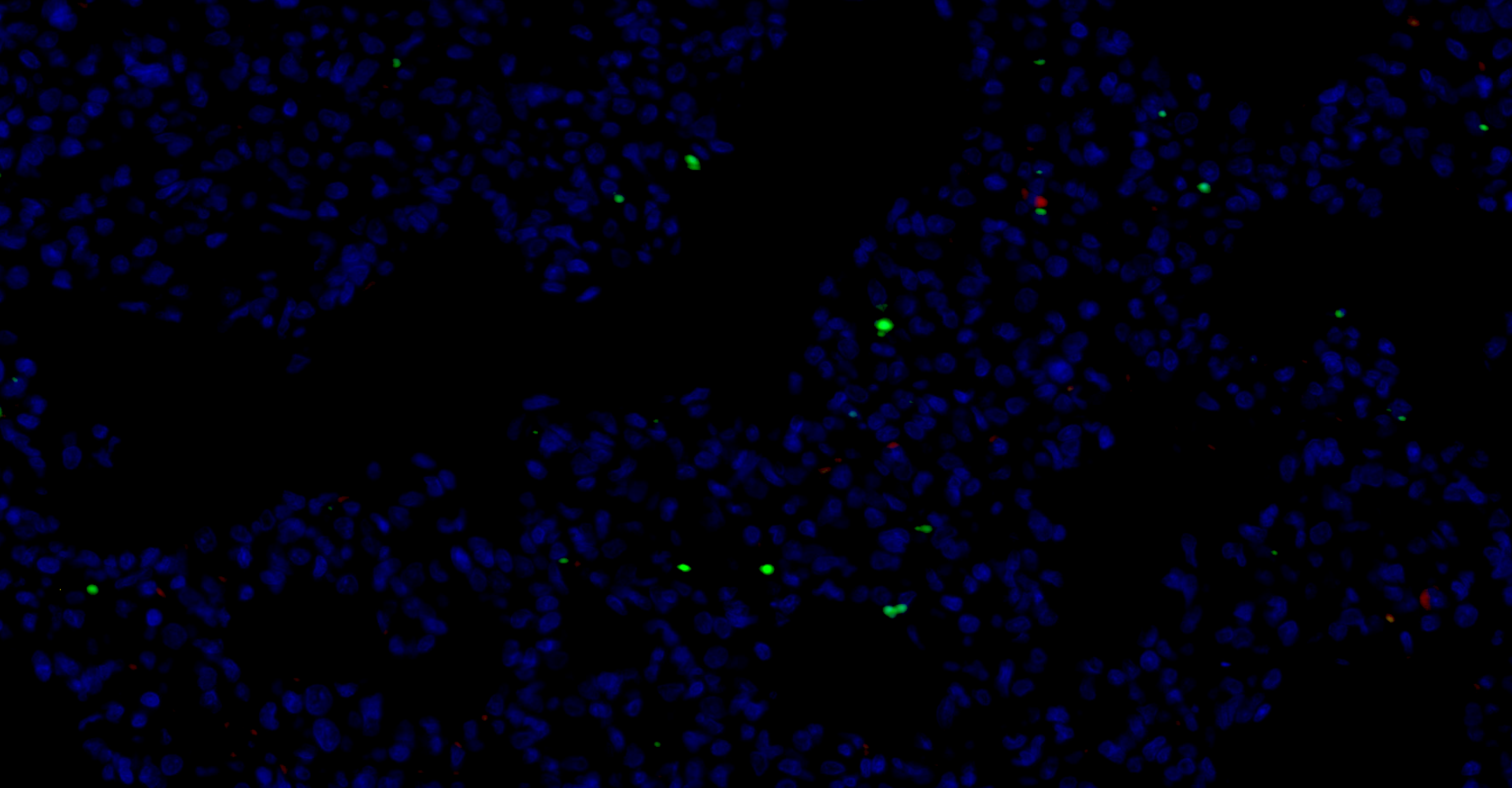

Supplement: Supplementary file 1 [file nutrients-17-02242-s001.zip › Figure S2 Original images/figure6-P-5 TUNEL-LY6G_40.0x.tif]

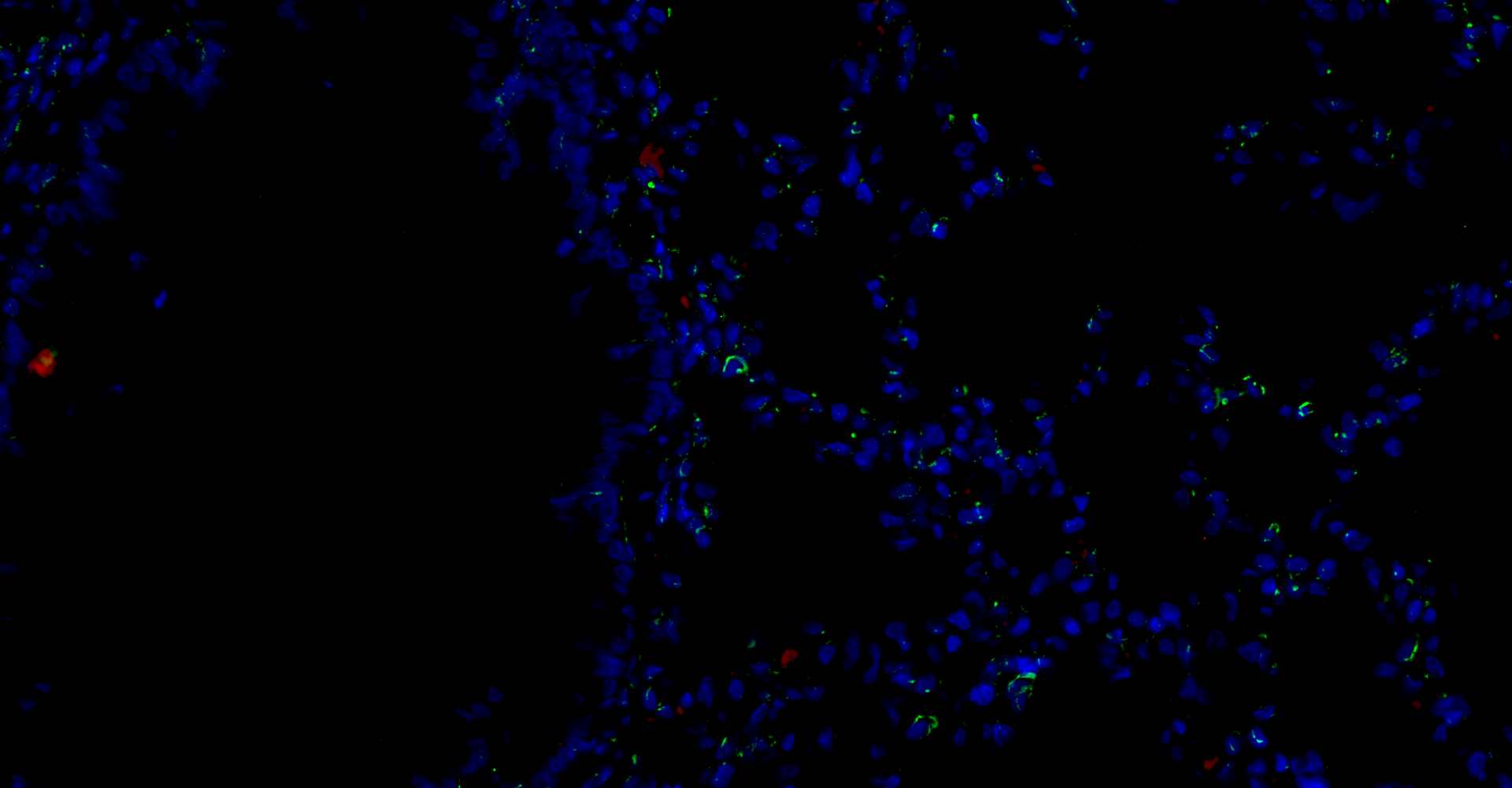

Supplement: Supplementary file 1 [file nutrients-17-02242-s001.zip › Figure S2 Original images/figure6-P-6 LY6G-ACH4_40.0x.tif]

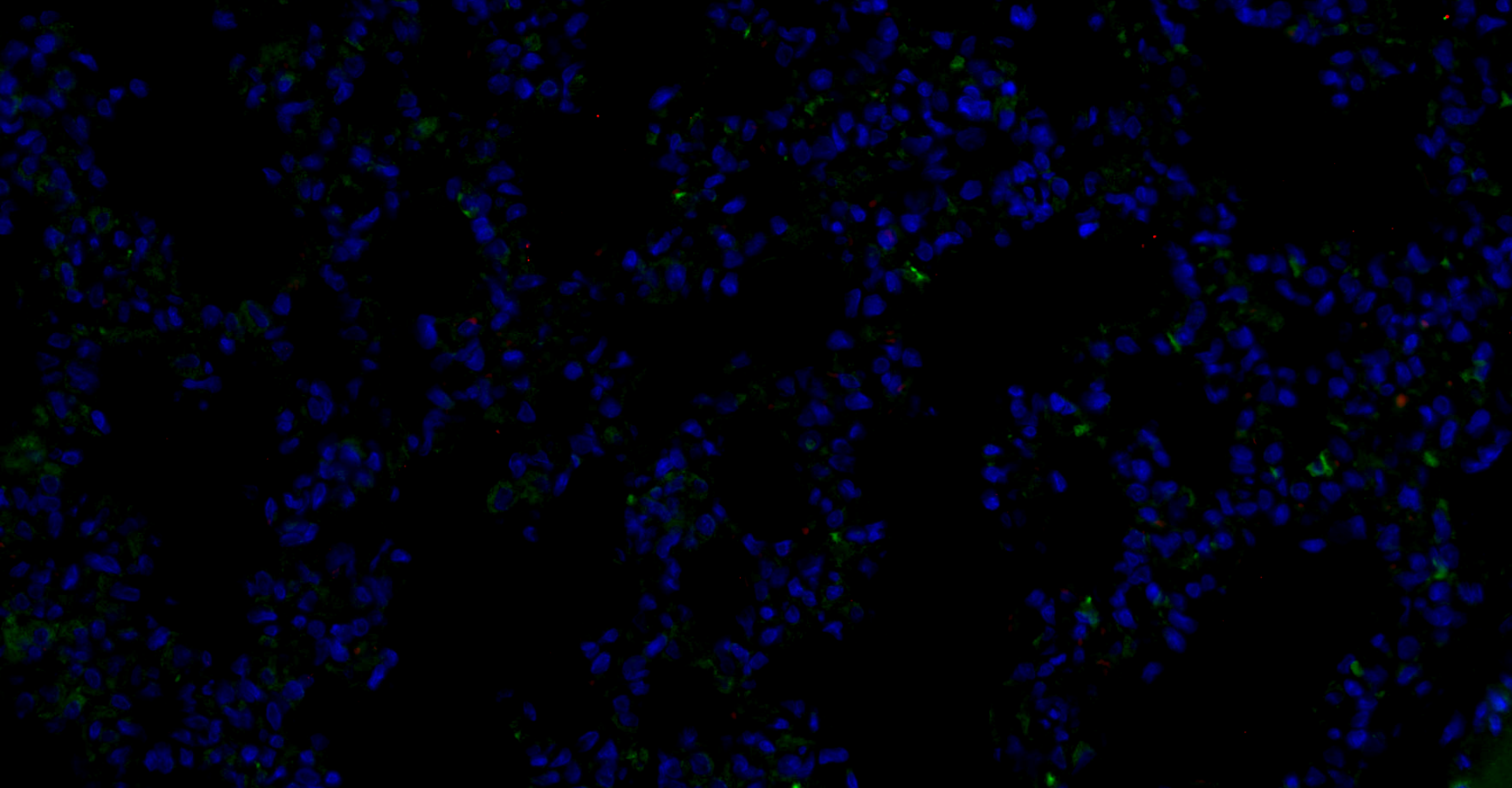

Supplement: Supplementary file 1 [file nutrients-17-02242-s001.zip › Figure S2 Original images/figure6-P-6 LY6G-CITH3_40.0x.tif]

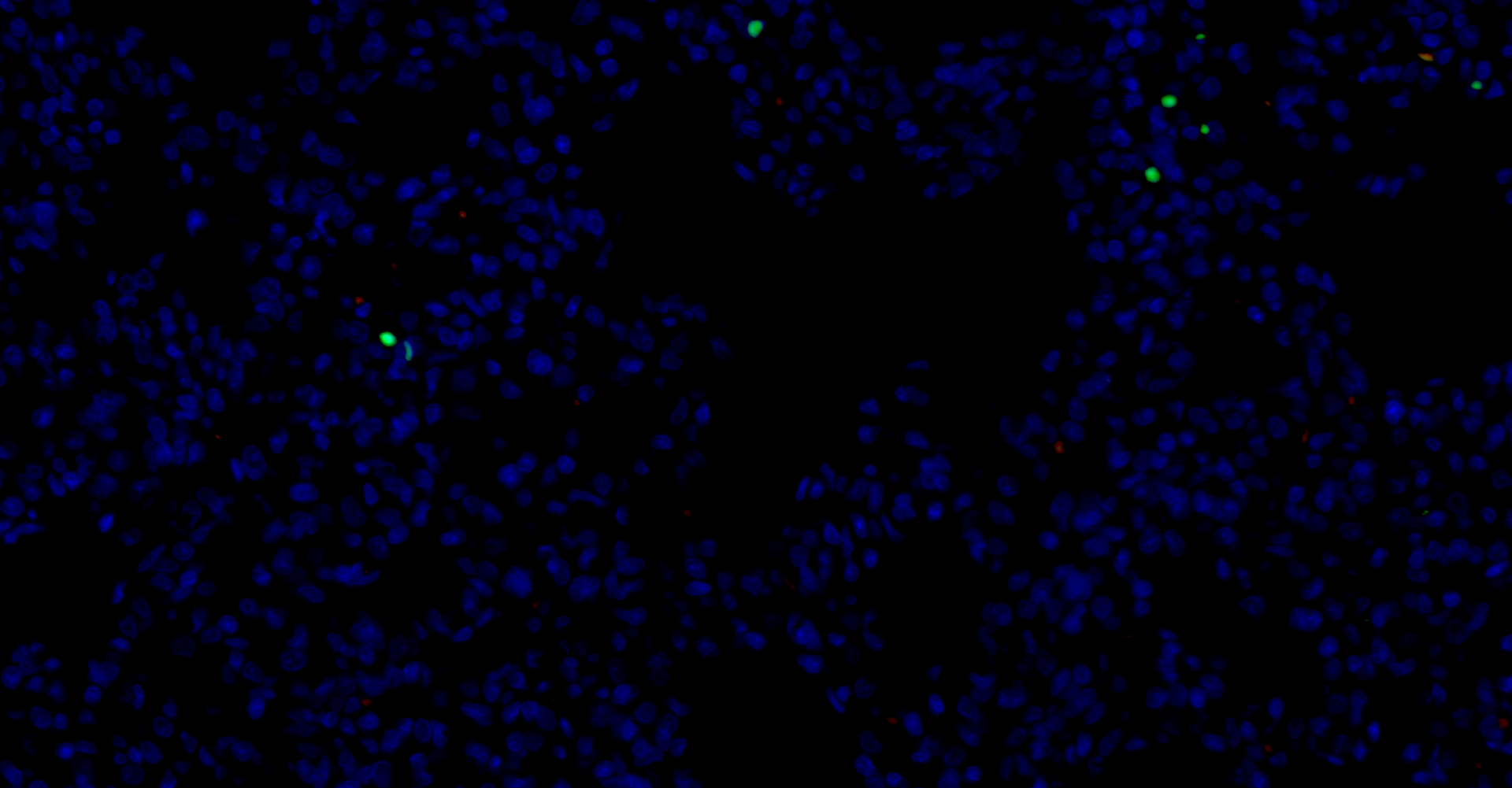

Supplement: Supplementary file 1 [file nutrients-17-02242-s001.zip › Figure S2 Original images/figure6-P-6 TUNEL-LY6G_40.0x.tif]

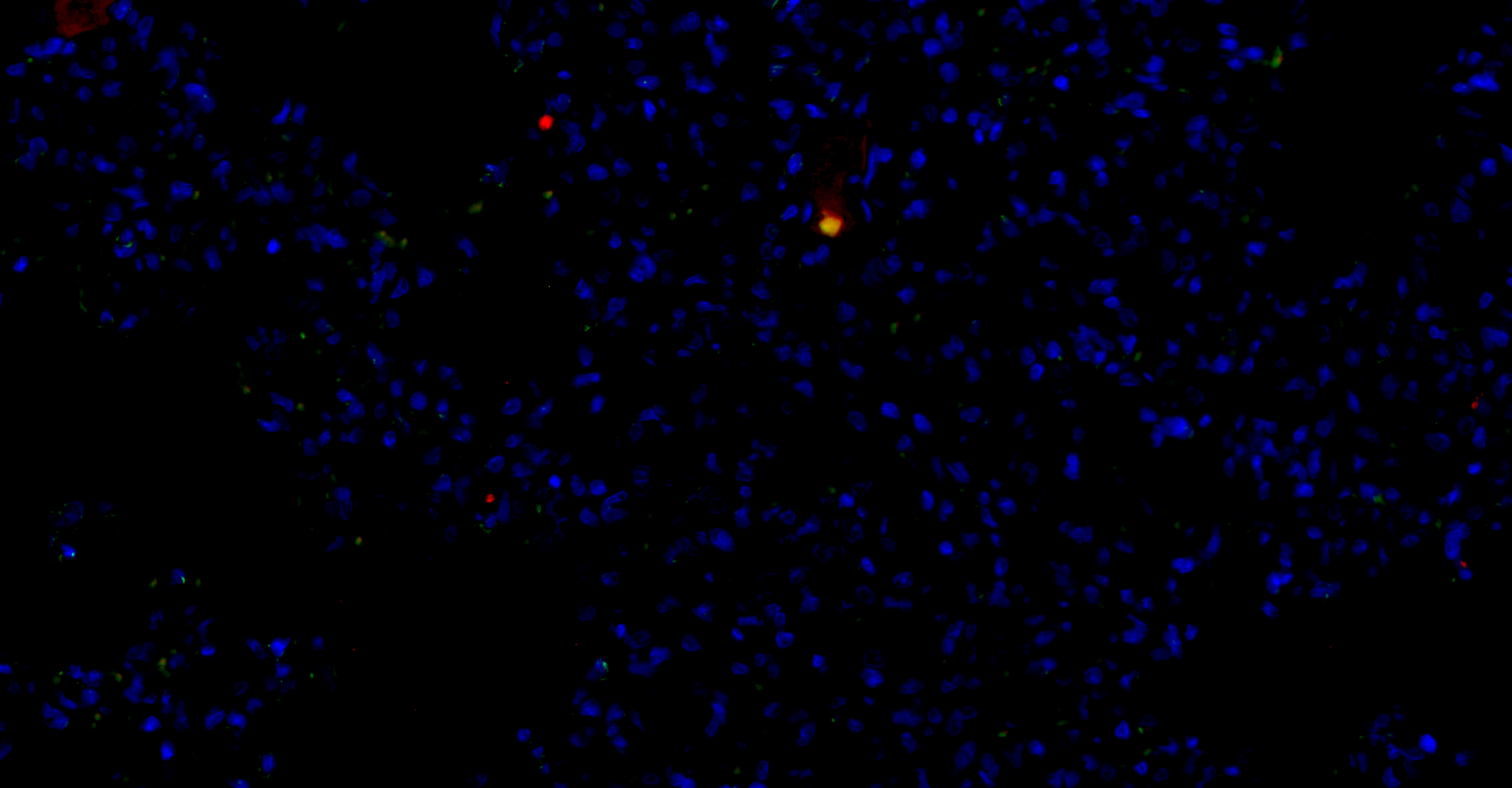

Supplement: Supplementary file 1 [file nutrients-17-02242-s001.zip › Figure S2 Original images/figure6-S-1 LY6G-ACH4_40.0x.tif]

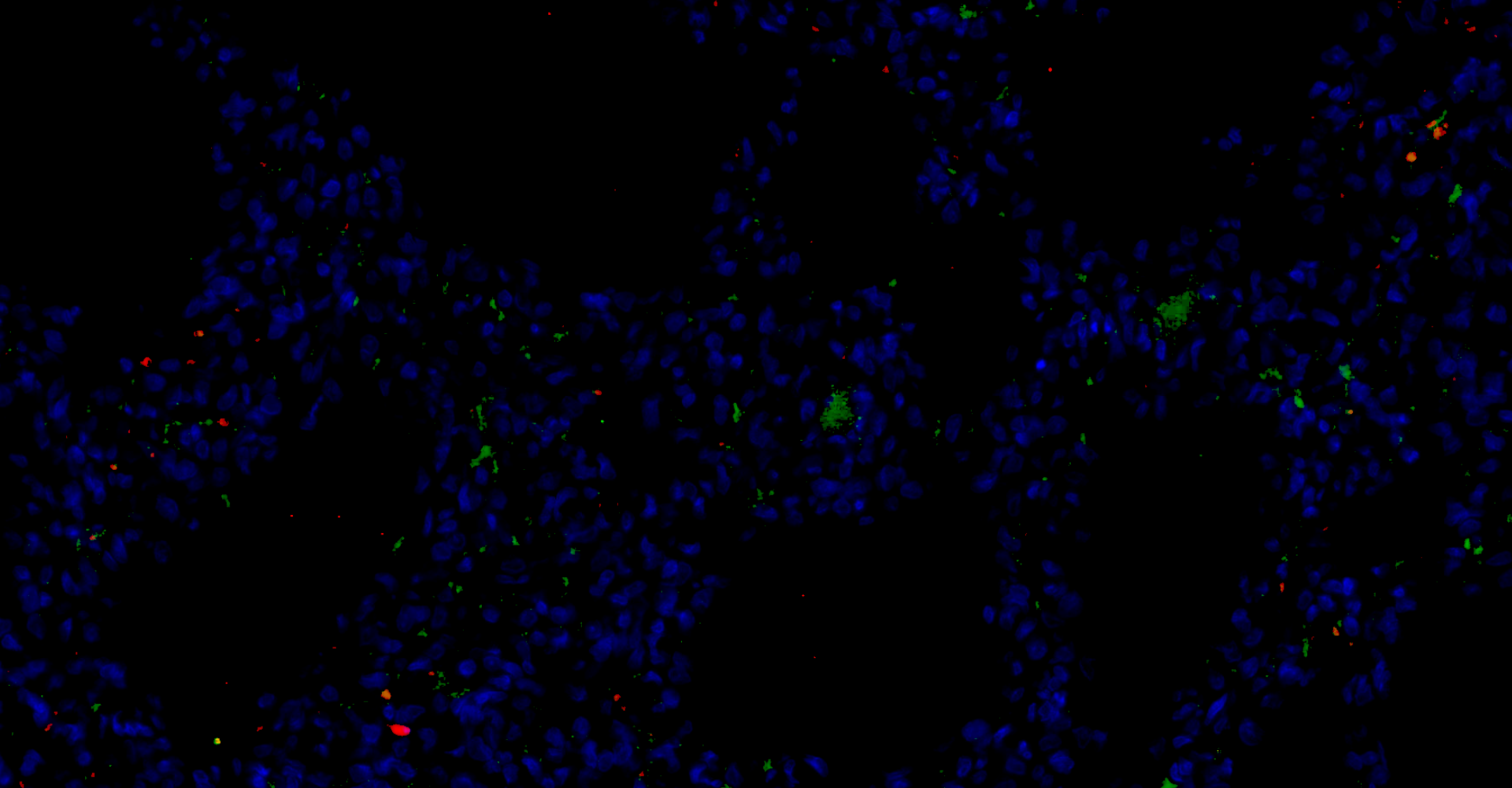

Supplement: Supplementary file 1 [file nutrients-17-02242-s001.zip › Figure S2 Original images/figure6-S-1 LY6G-CITH3_40.0x.tif]

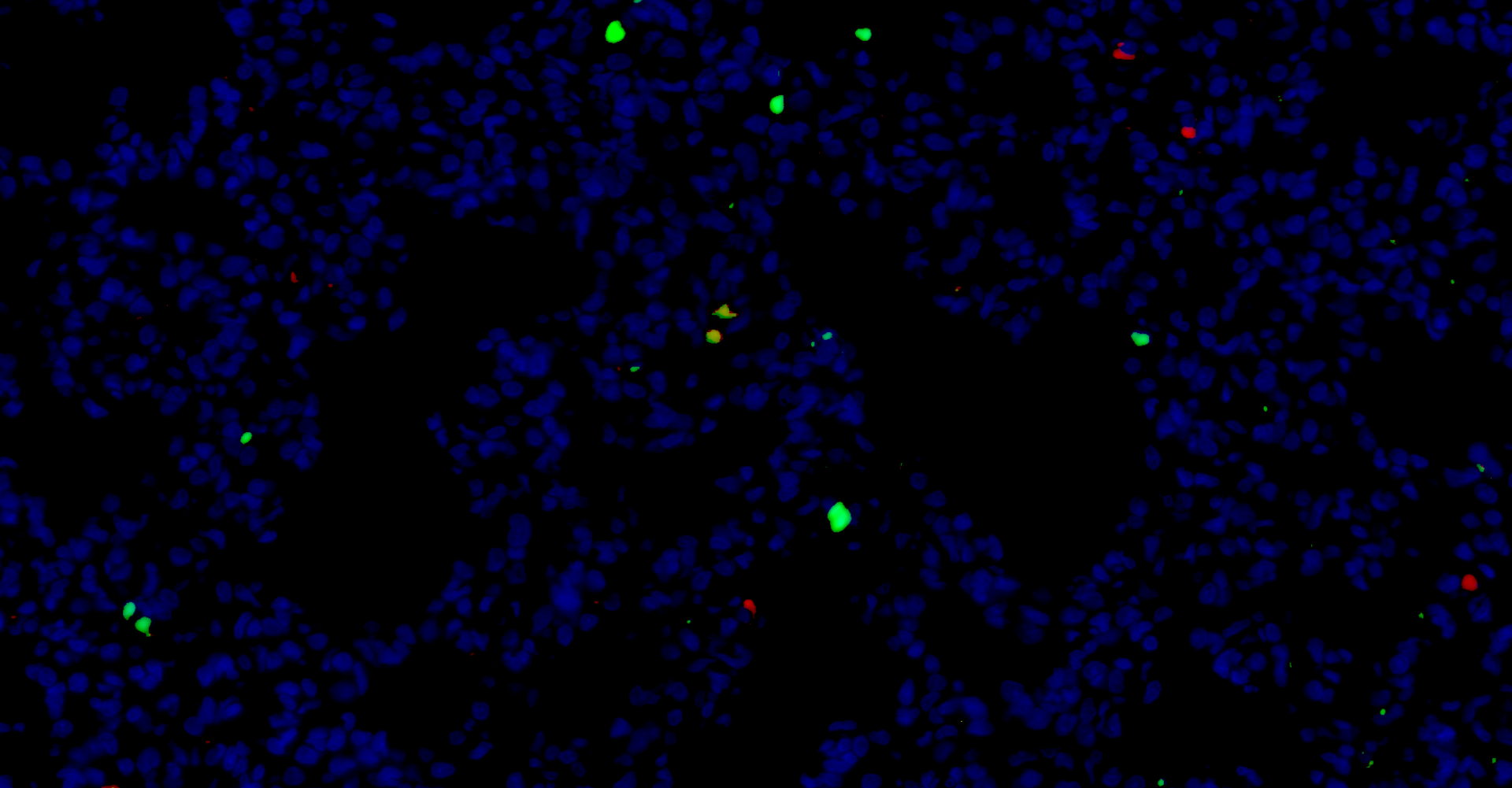

Supplement: Supplementary file 1 [file nutrients-17-02242-s001.zip › Figure S2 Original images/figure6-S-1 TUNEL-LY6G_40.0x.tif]

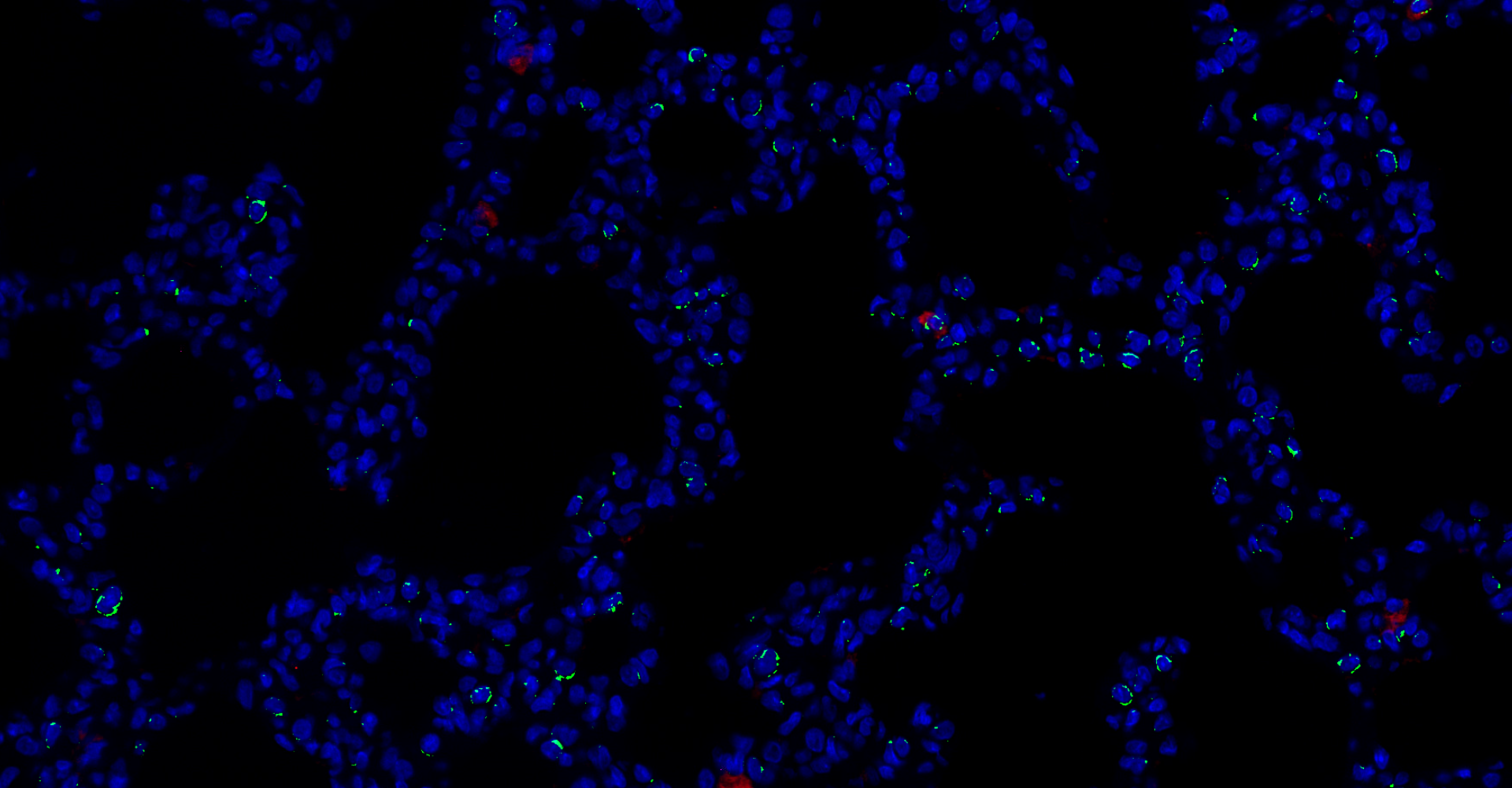

Supplement: Supplementary file 1 [file nutrients-17-02242-s001.zip › Figure S2 Original images/figure6-S-2 LY6G-ACH4_40.0x.tif]

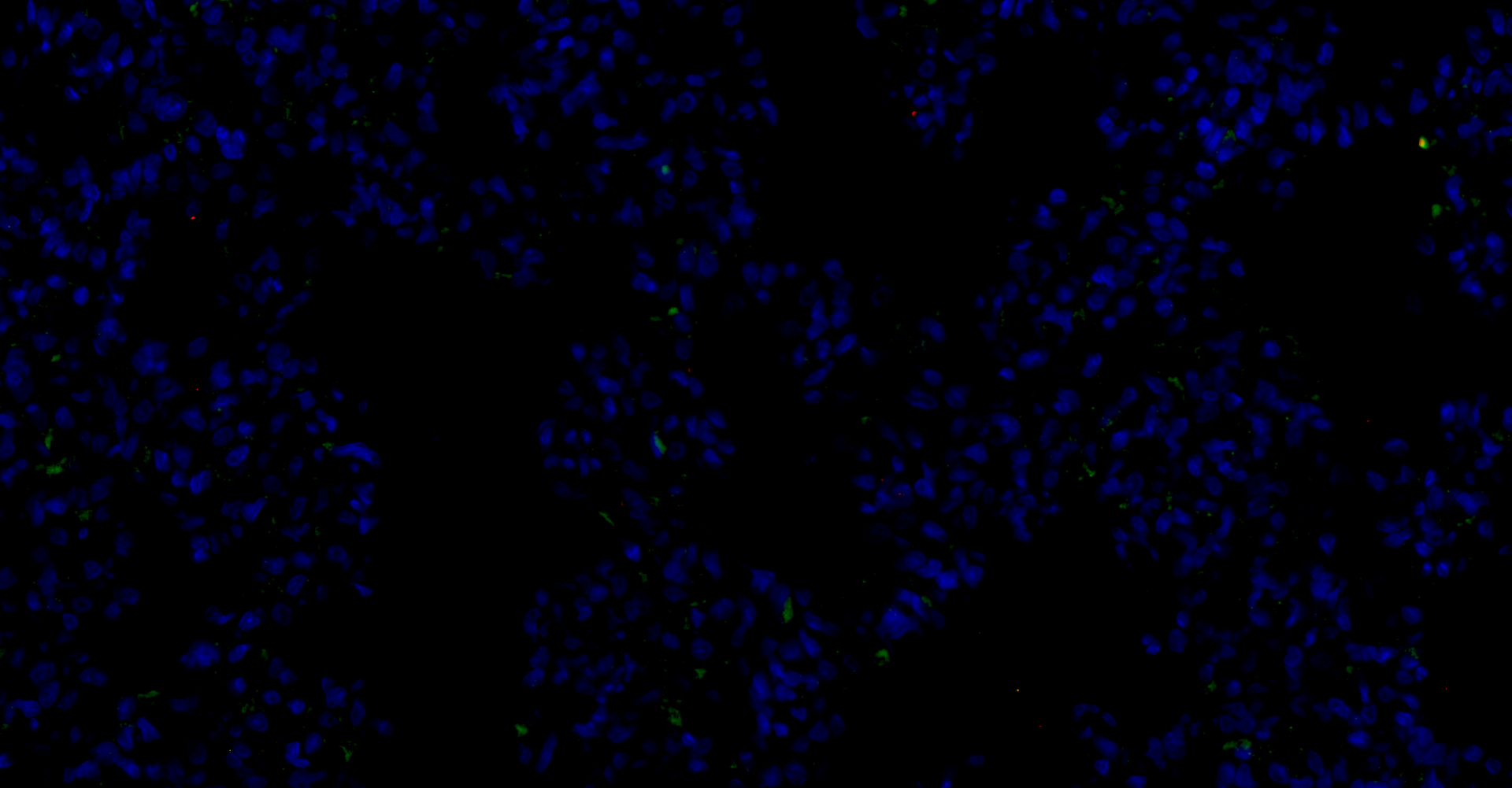

Supplement: Supplementary file 1 [file nutrients-17-02242-s001.zip › Figure S2 Original images/figure6-S-2 LY6G-CITH3_40.0x.tif]

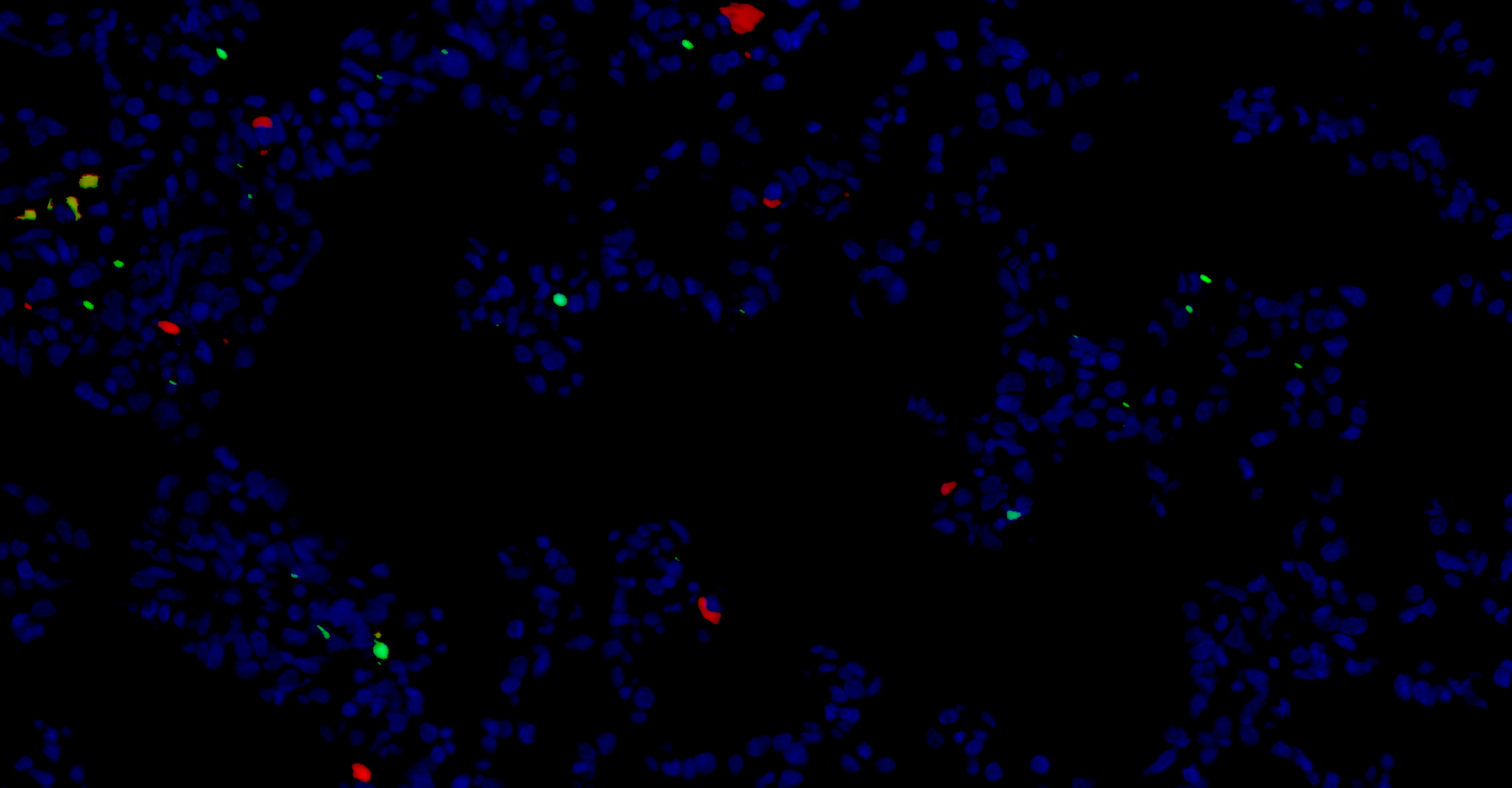

Supplement: Supplementary file 1 [file nutrients-17-02242-s001.zip › Figure S2 Original images/figure6-S-2 TUNEL-LY6G_40.0x.tif]

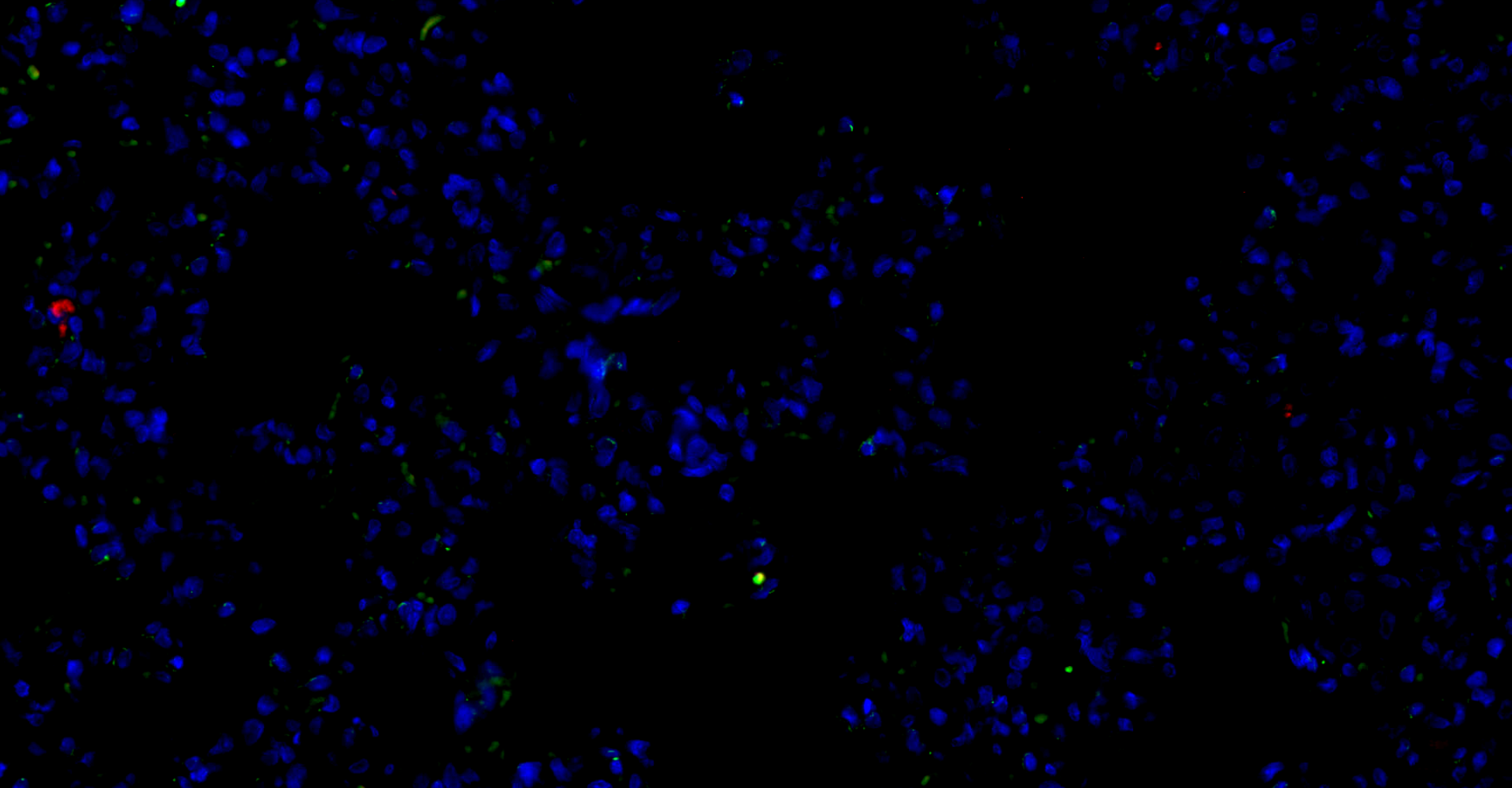

Supplement: Supplementary file 1 [file nutrients-17-02242-s001.zip › Figure S2 Original images/figure6-S-3 LY6G-ACH4_40.0x.tif]

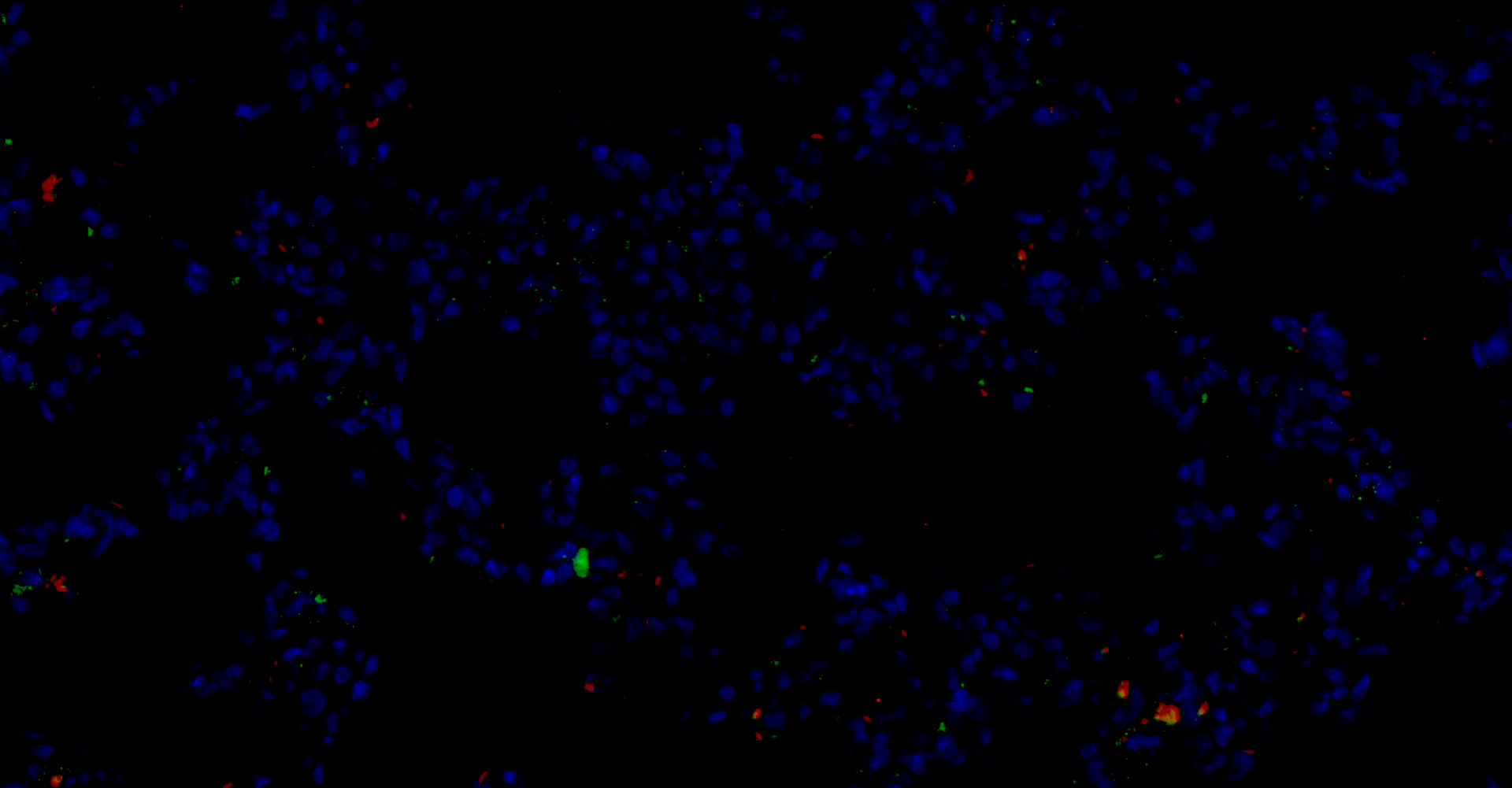

Supplement: Supplementary file 1 [file nutrients-17-02242-s001.zip › Figure S2 Original images/figure6-S-3 LY6G-CITH3_40.0x.tif]

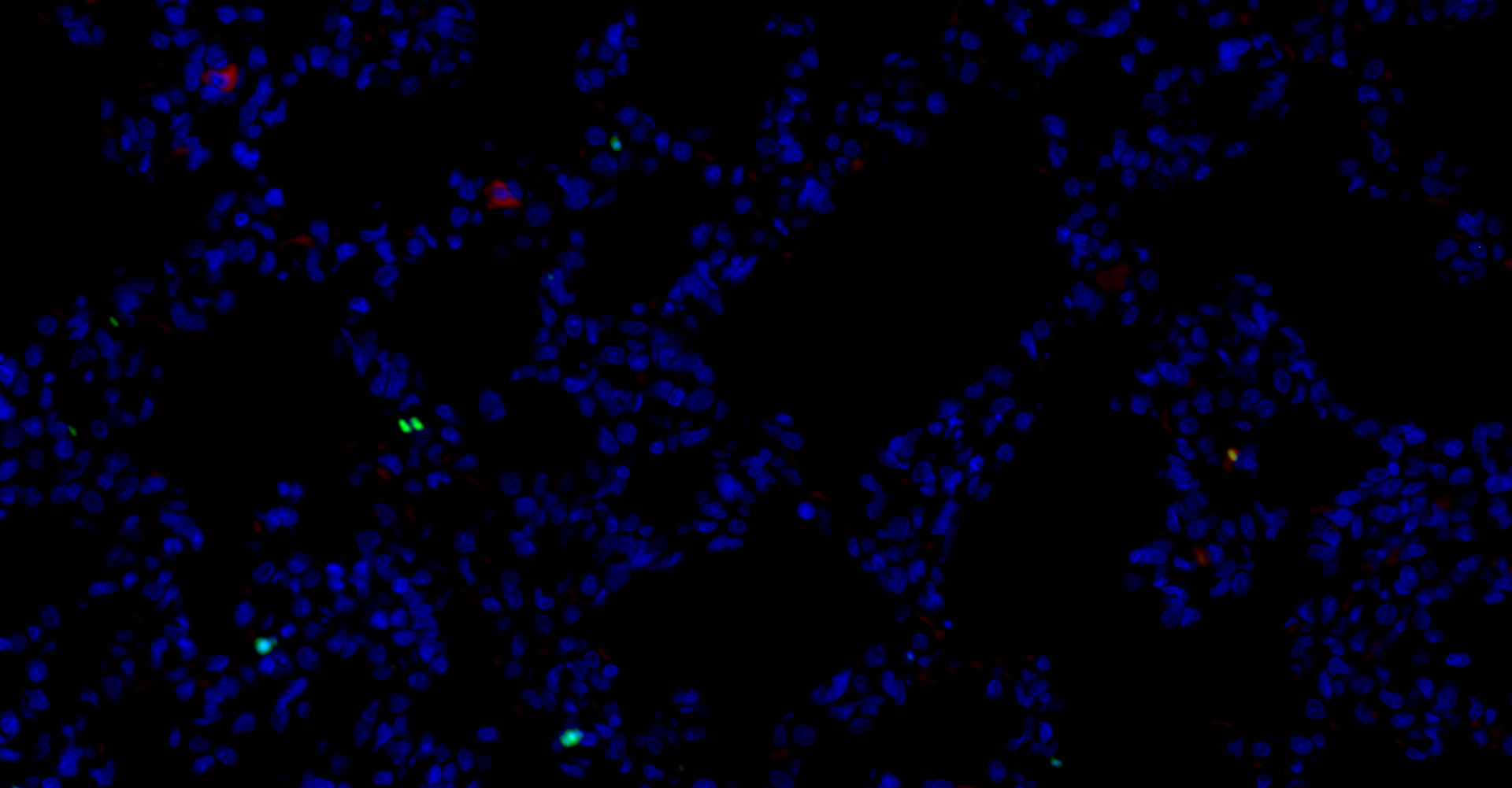

Supplement: Supplementary file 1 [file nutrients-17-02242-s001.zip › Figure S2 Original images/figure6-S-3 TUNEL-LY6G_40.0x.tif]

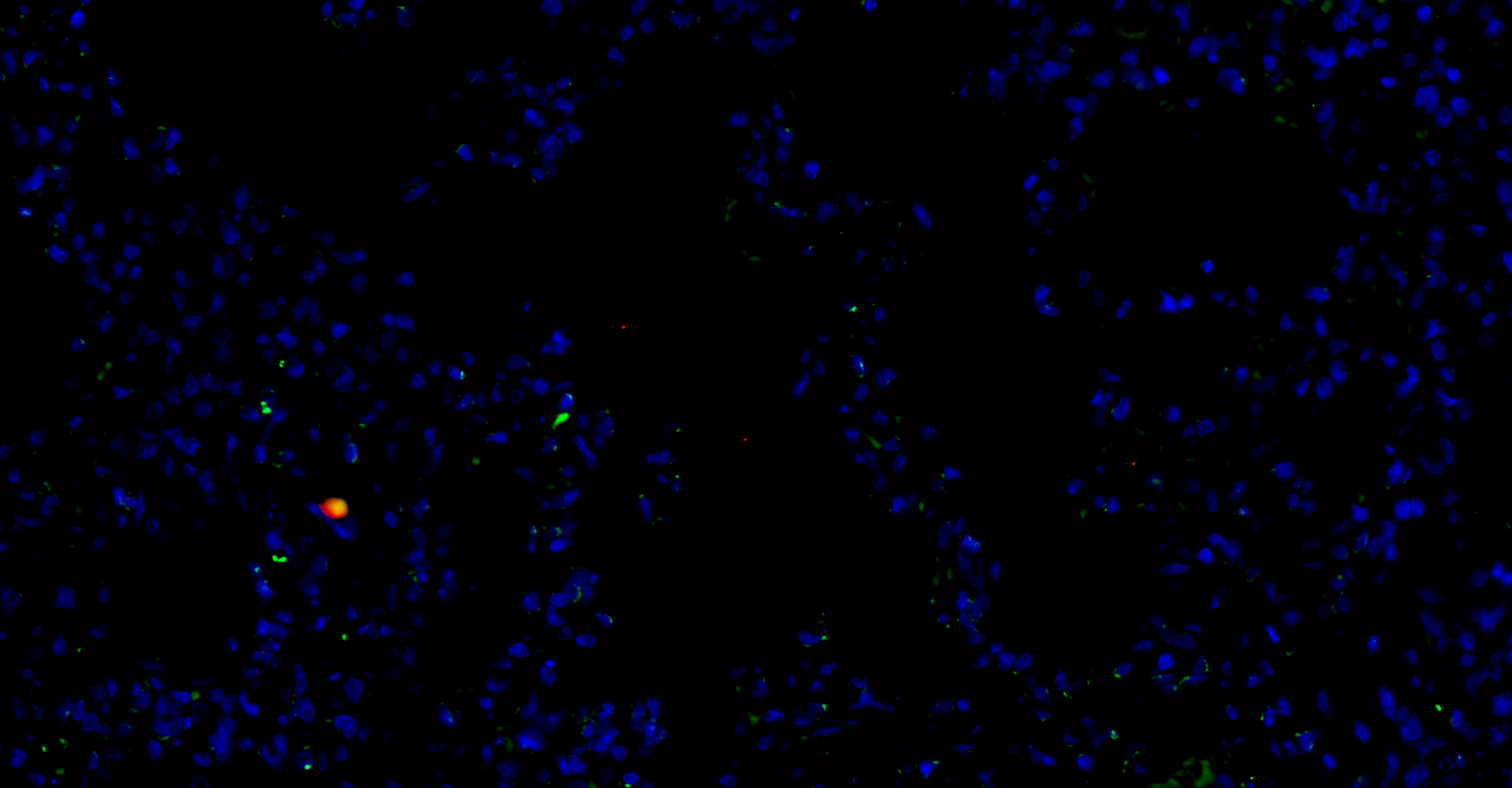

Supplement: Supplementary file 1 [file nutrients-17-02242-s001.zip › Figure S2 Original images/figure6-S-4 LY6G-ACH4_40.0x.tif]

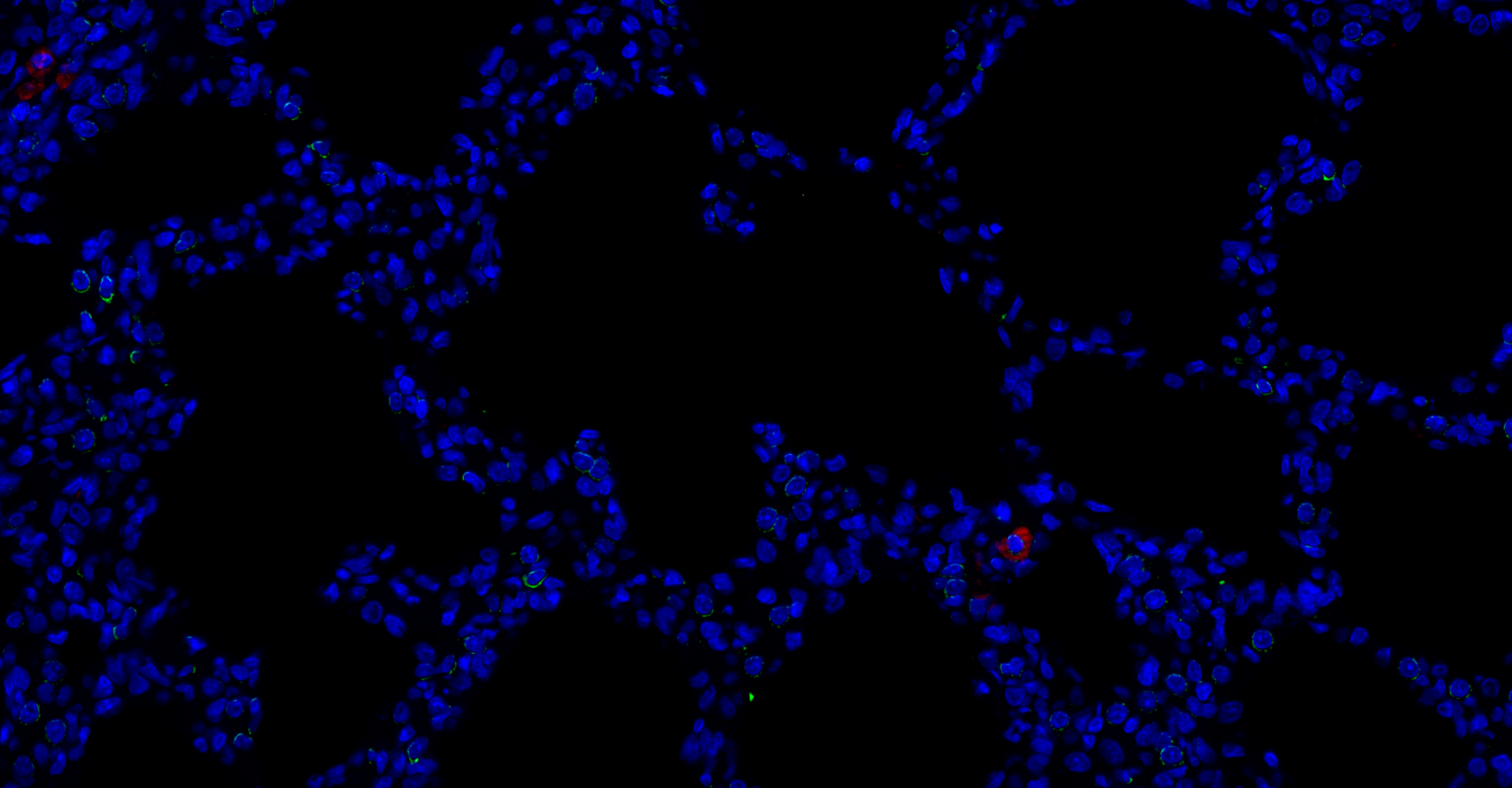

Supplement: Supplementary file 1 [file nutrients-17-02242-s001.zip › Figure S2 Original images/figure6-S-4 LY6G-CITH3_40.0x.tif]

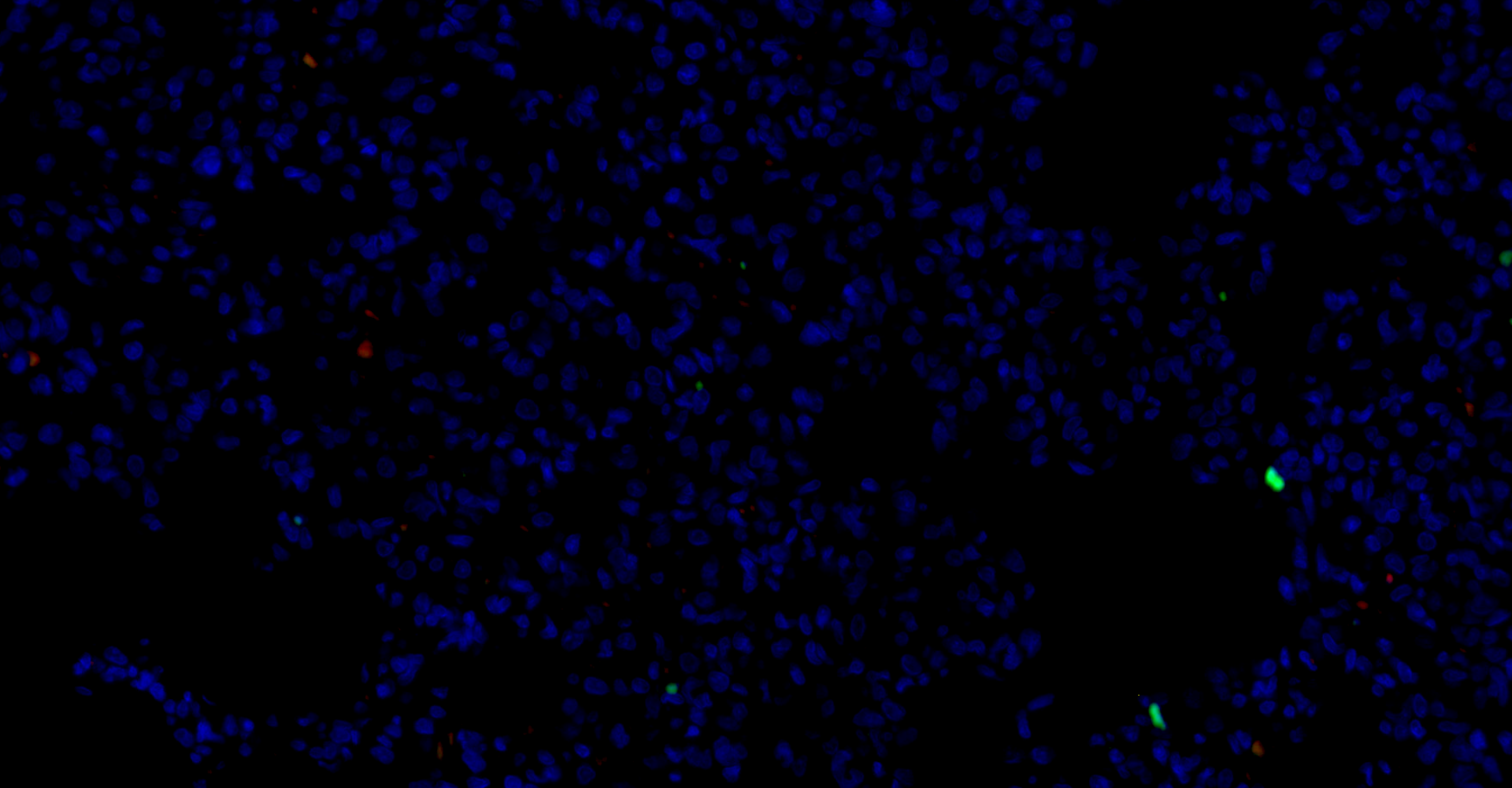

Supplement: Supplementary file 1 [file nutrients-17-02242-s001.zip › Figure S2 Original images/figure6-S-4 TUNEL-LY6G_40.0x.tif]

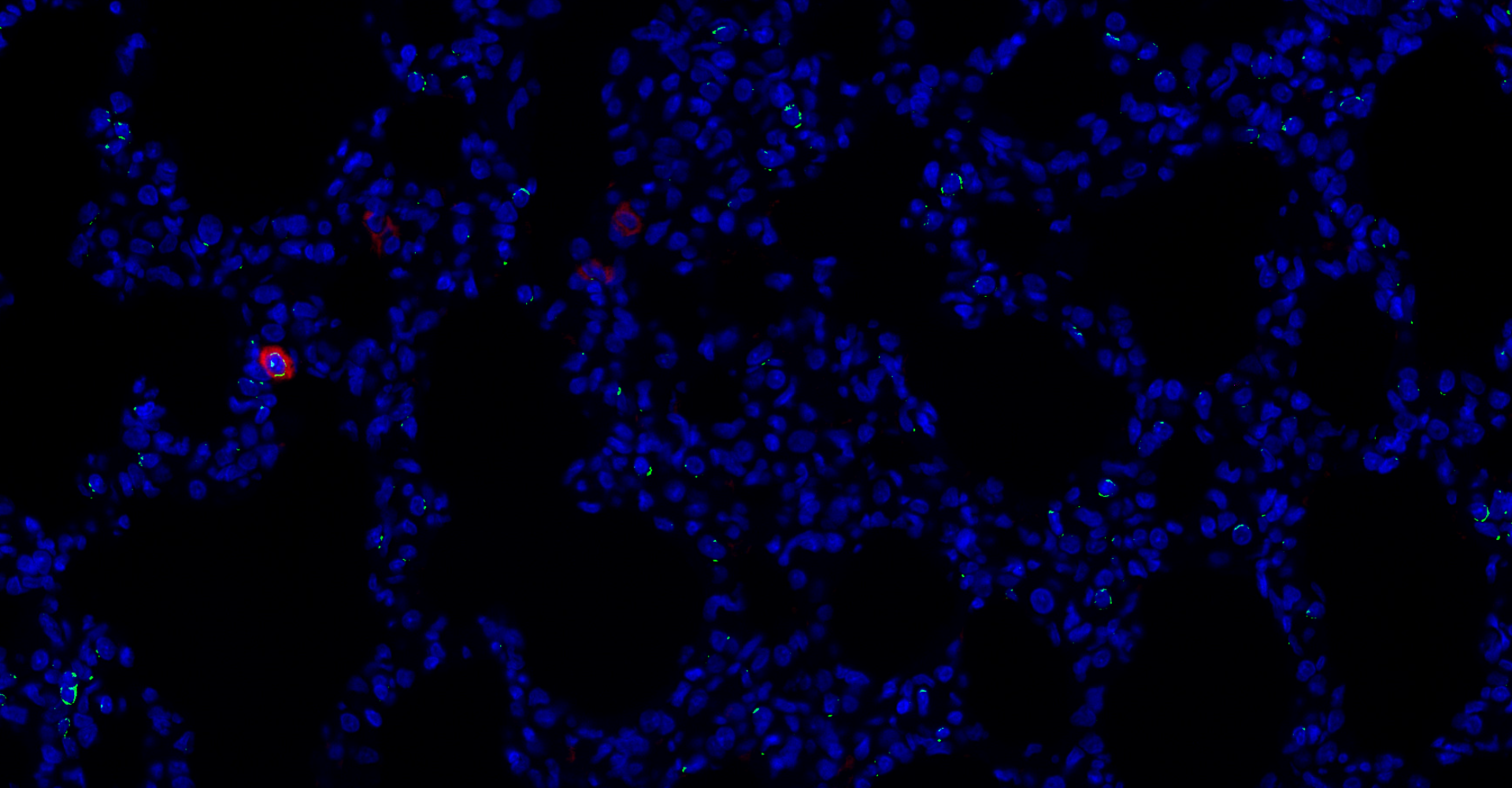

Supplement: Supplementary file 1 [file nutrients-17-02242-s001.zip › Figure S2 Original images/figure6-S-5 LY6G-ACH4_40.0x.tif]

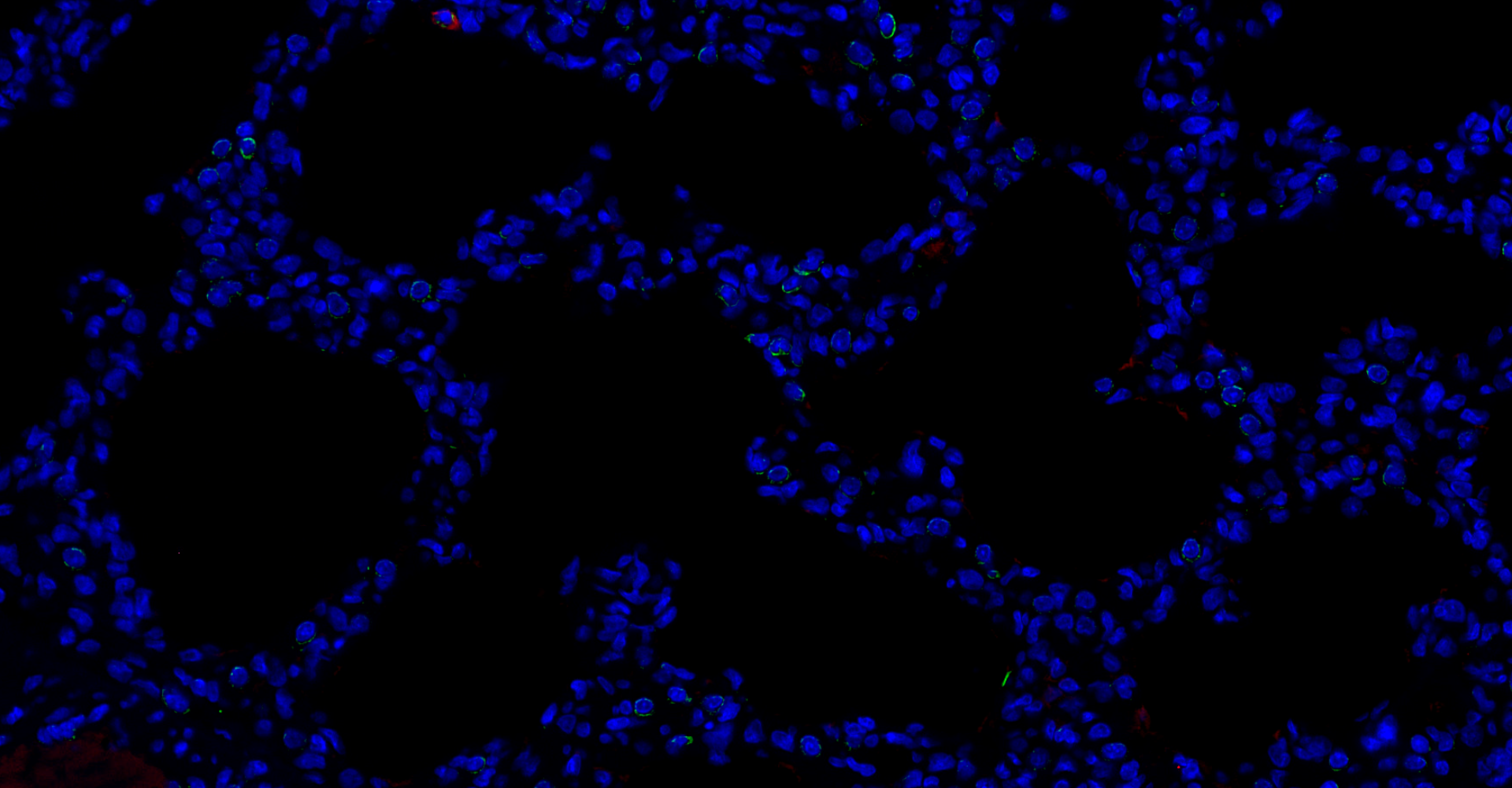

Supplement: Supplementary file 1 [file nutrients-17-02242-s001.zip › Figure S2 Original images/figure6-S-5 LY6G-CITH3_40.0x.tif]

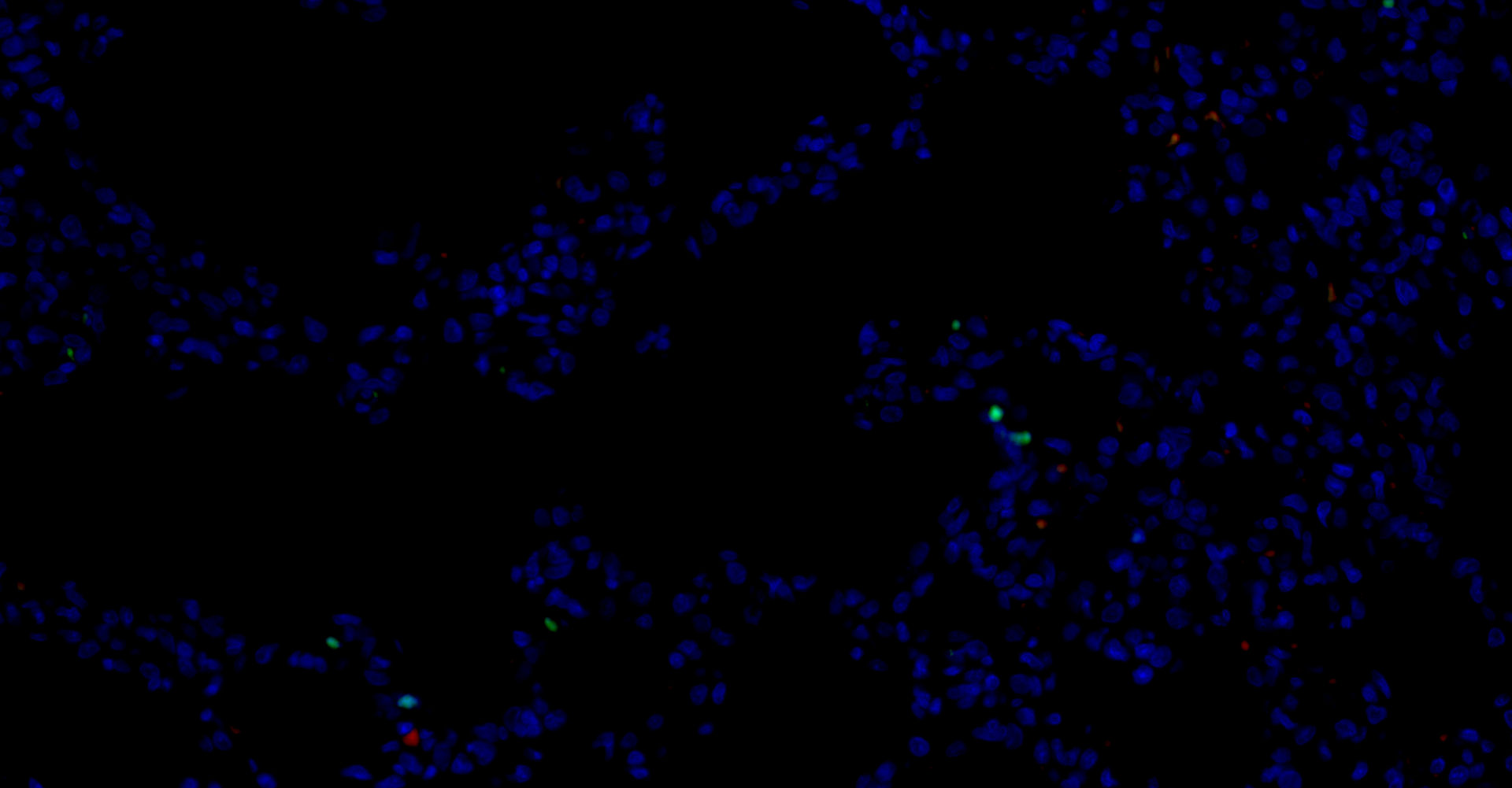

Supplement: Supplementary file 1 [file nutrients-17-02242-s001.zip › Figure S2 Original images/figure6-S-5 TUNEL-LY6G_40.0x.tif]

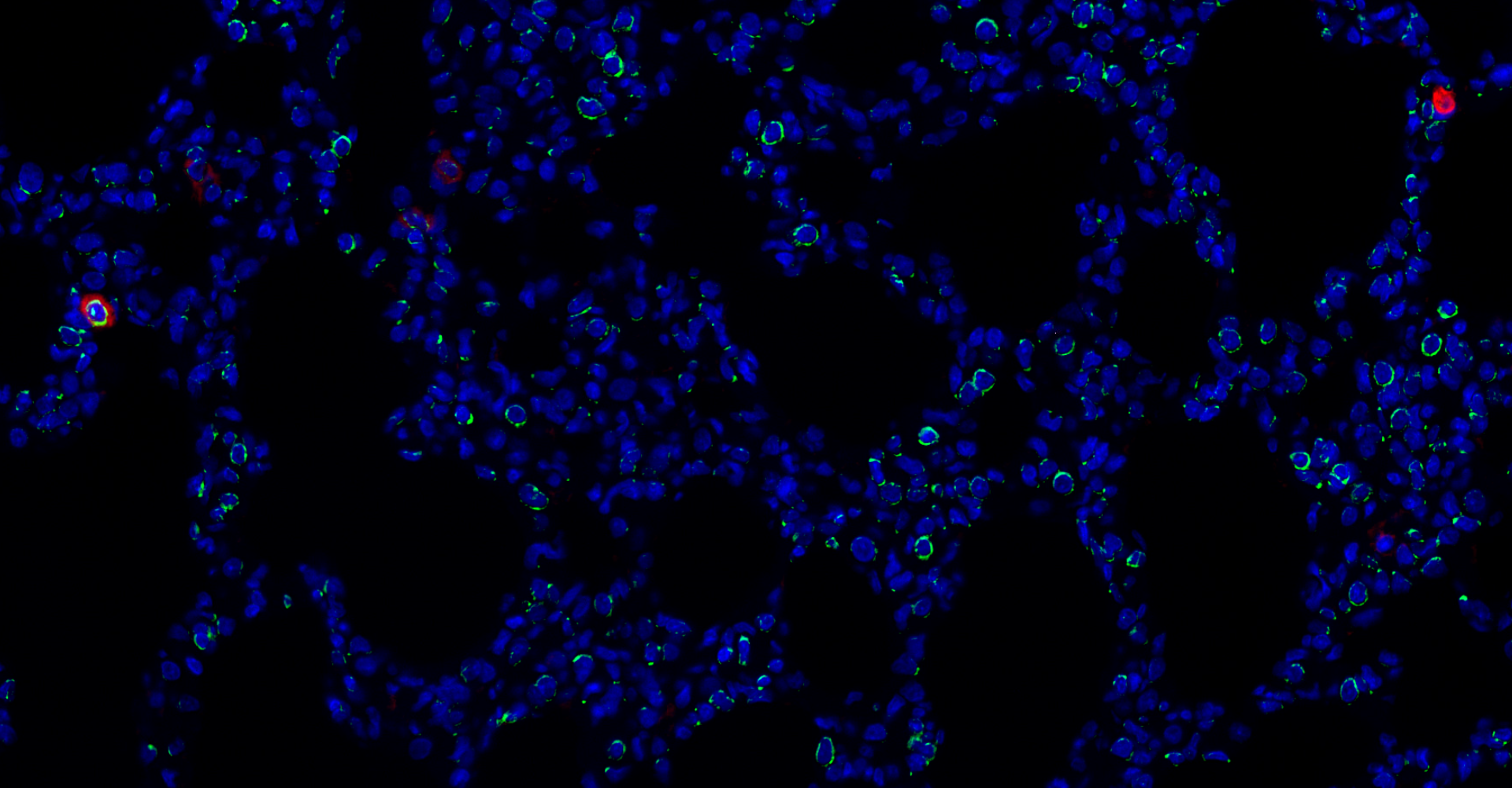

Supplement: Supplementary file 1 [file nutrients-17-02242-s001.zip › Figure S2 Original images/figure6-S-6 LY6G-ACH4_40.0x.tif]

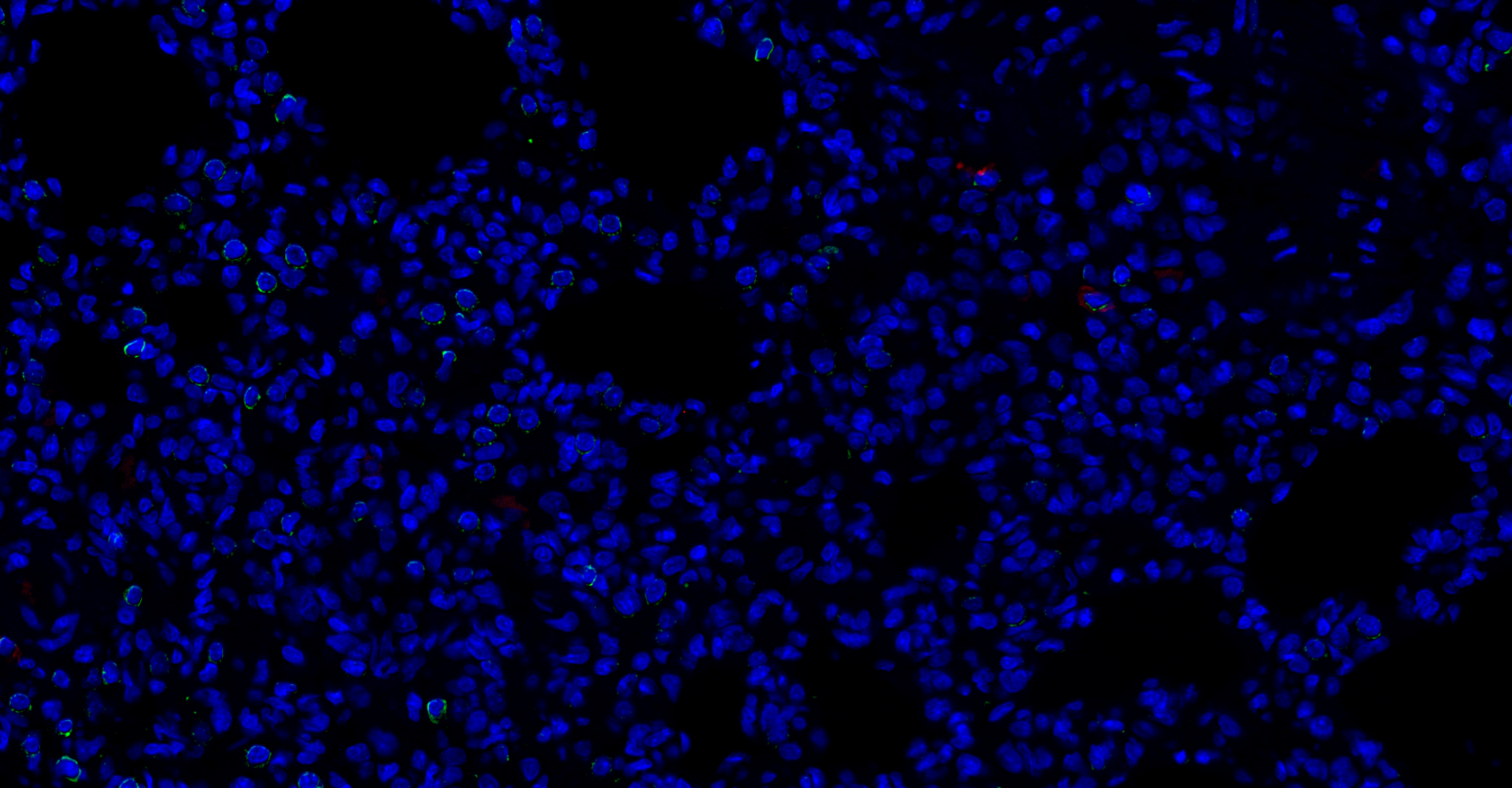

Supplement: Supplementary file 1 [file nutrients-17-02242-s001.zip › Figure S2 Original images/figure6-S-6 LY6G-CITH3_40.0x.tif]

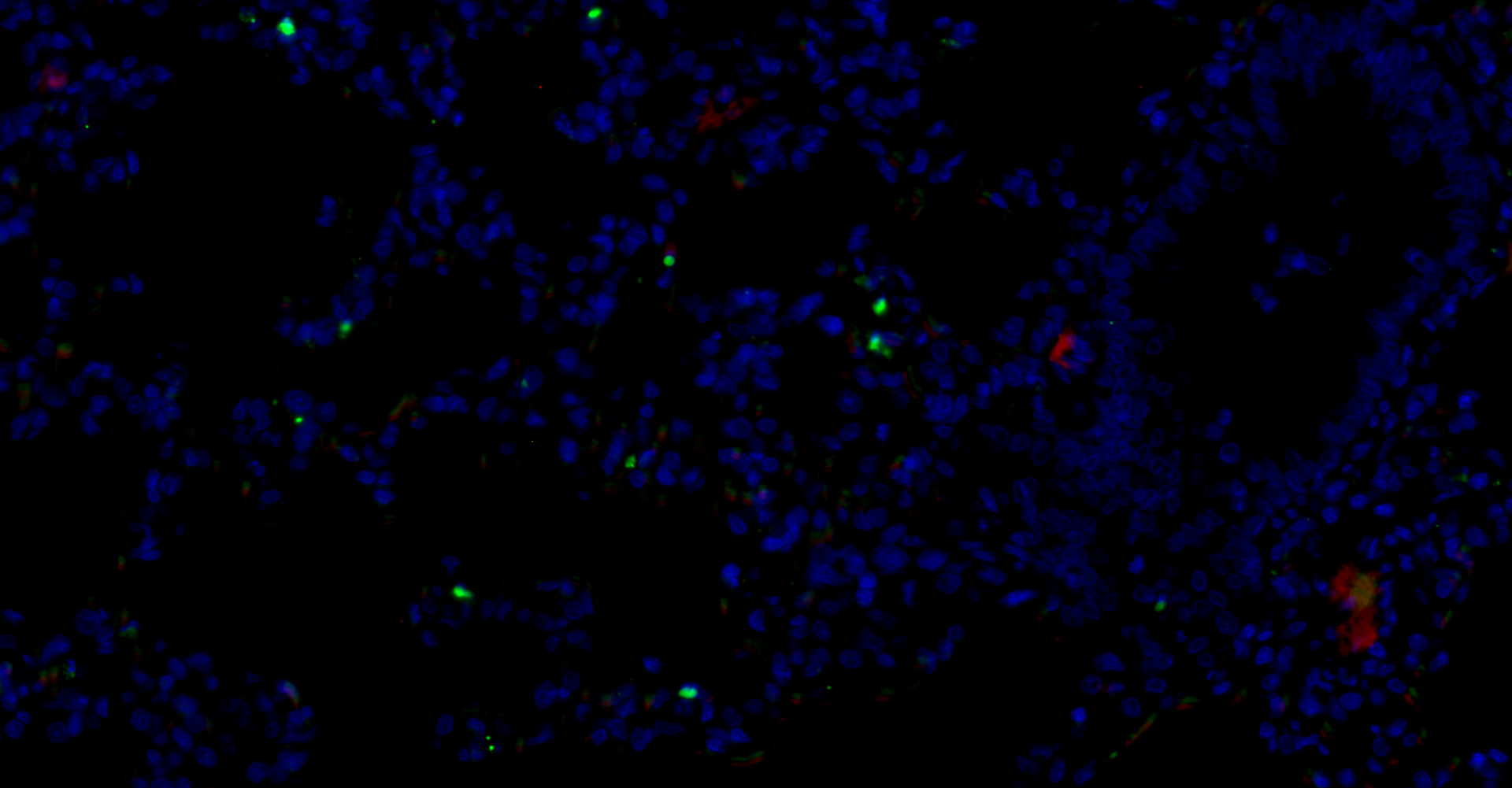

Supplement: Supplementary file 1 [file nutrients-17-02242-s001.zip › Figure S2 Original images/figure6-S-6 TUNEL-LY6G_40.0x.tif]

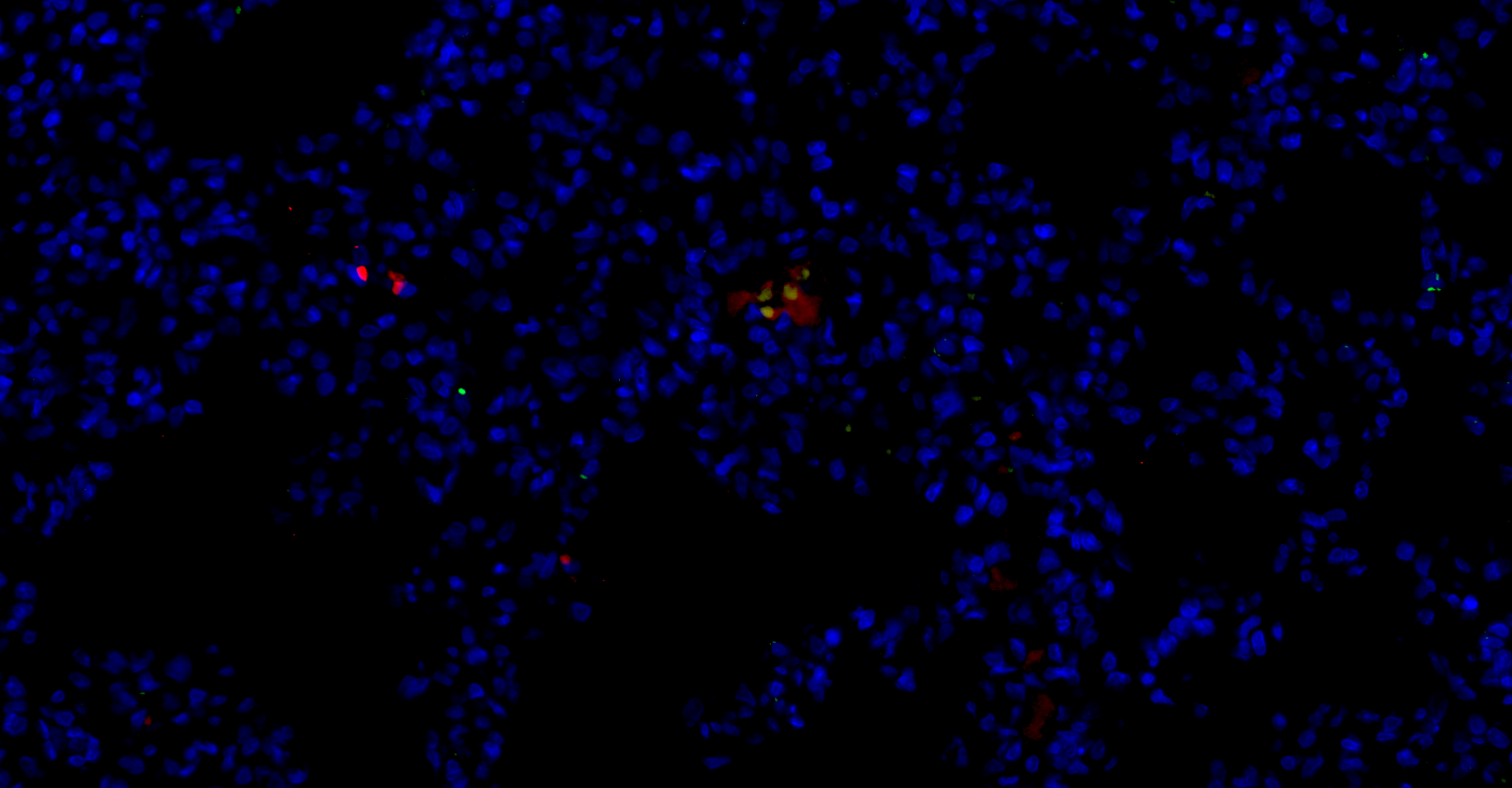

Supplement: Supplementary file 1 [file nutrients-17-02242-s001.zip › Figure S2 Original images/figure6-T-1 LY6G-ACH4_40.0x.tif]

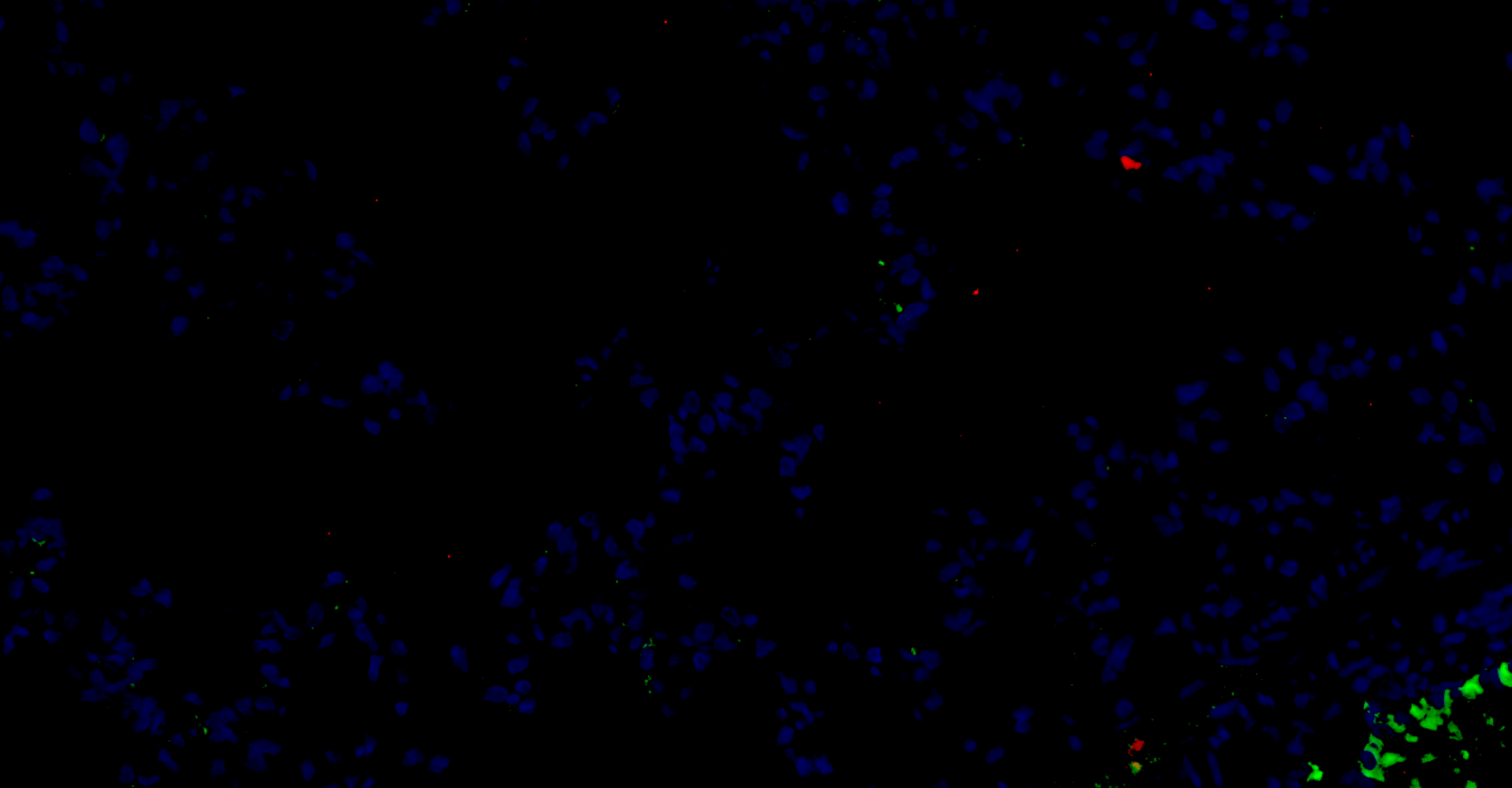

Supplement: Supplementary file 1 [file nutrients-17-02242-s001.zip › Figure S2 Original images/figure6-T-1 LY6G-CITH3_40.0x.tif]

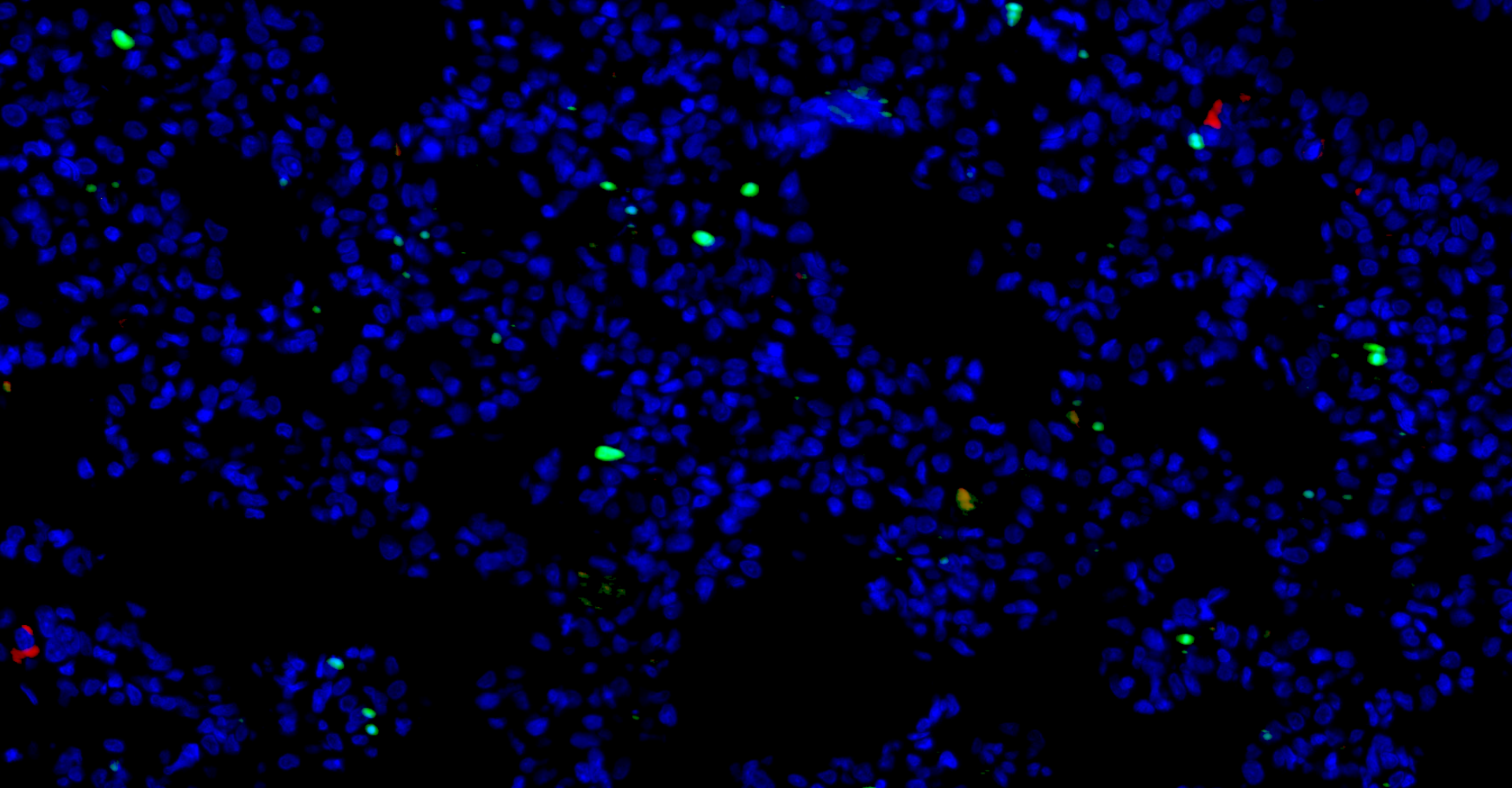

Supplement: Supplementary file 1 [file nutrients-17-02242-s001.zip › Figure S2 Original images/figure6-T-1 TUNEL-LY6G_40.0x.tif]

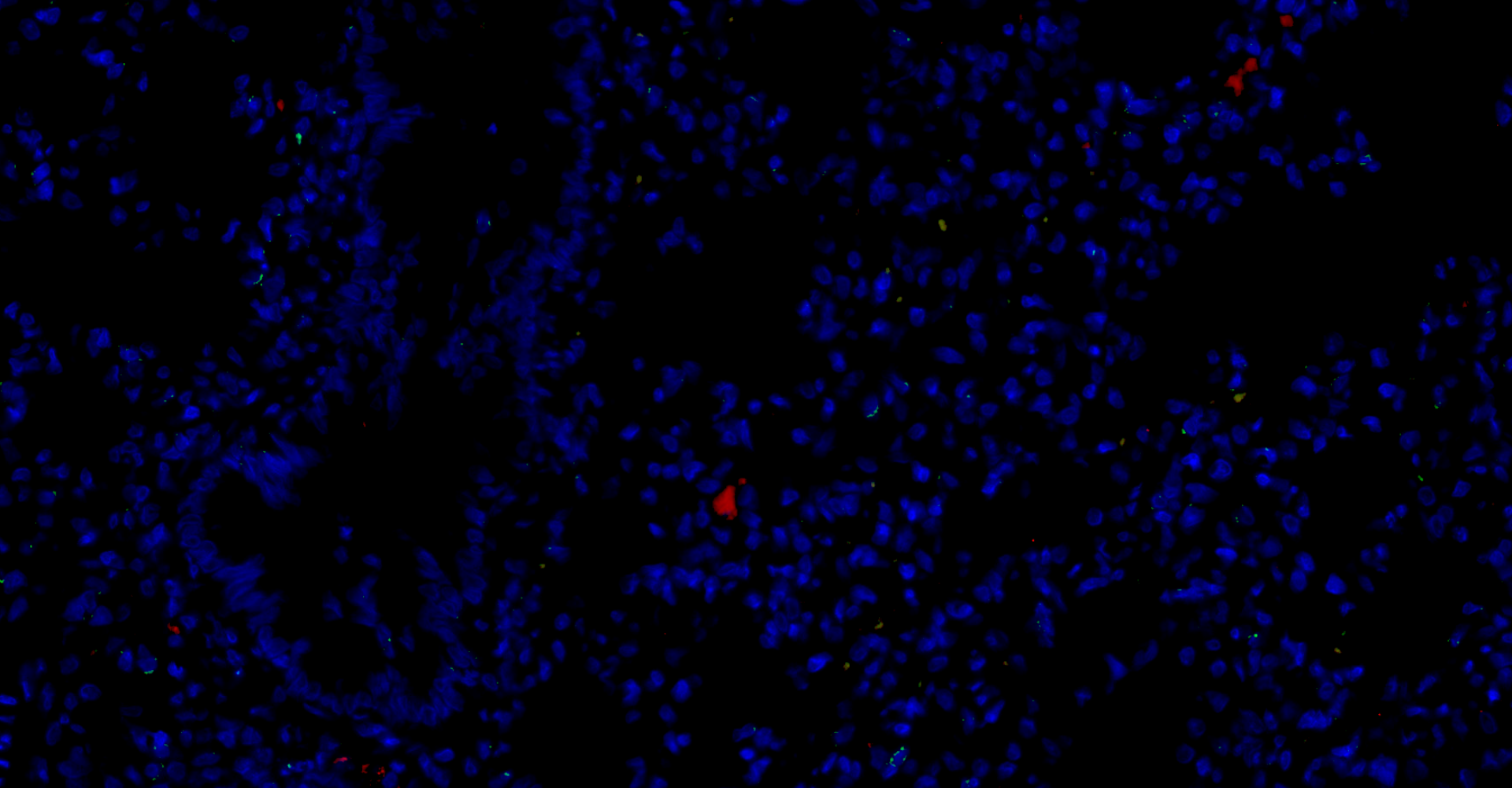

Supplement: Supplementary file 1 [file nutrients-17-02242-s001.zip › Figure S2 Original images/figure6-T-2 LY6G-ACH4_40.0x.tif]

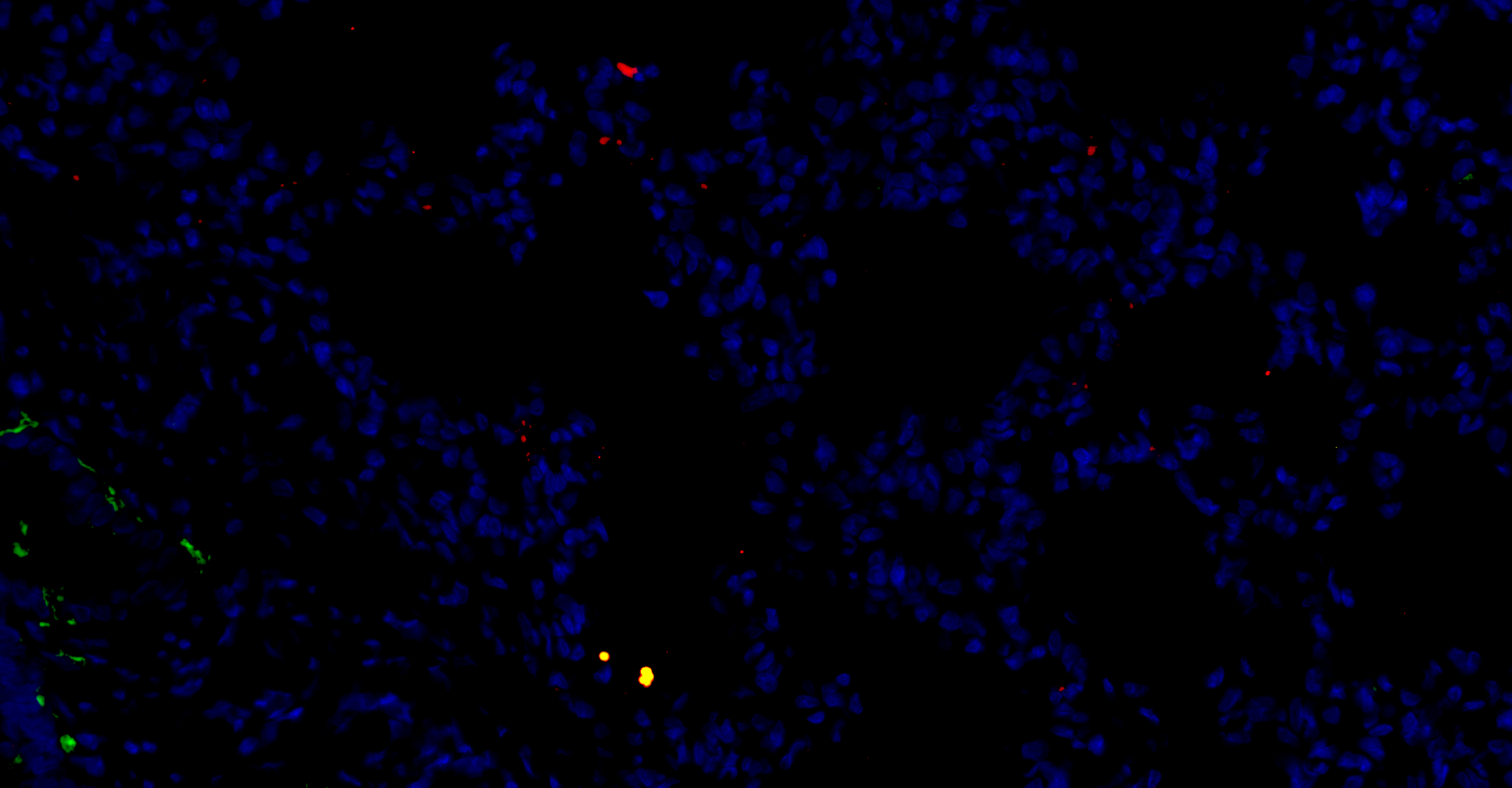

Supplement: Supplementary file 1 [file nutrients-17-02242-s001.zip › Figure S2 Original images/figure6-T-2 LY6G-CITH3_40.0x.tif]

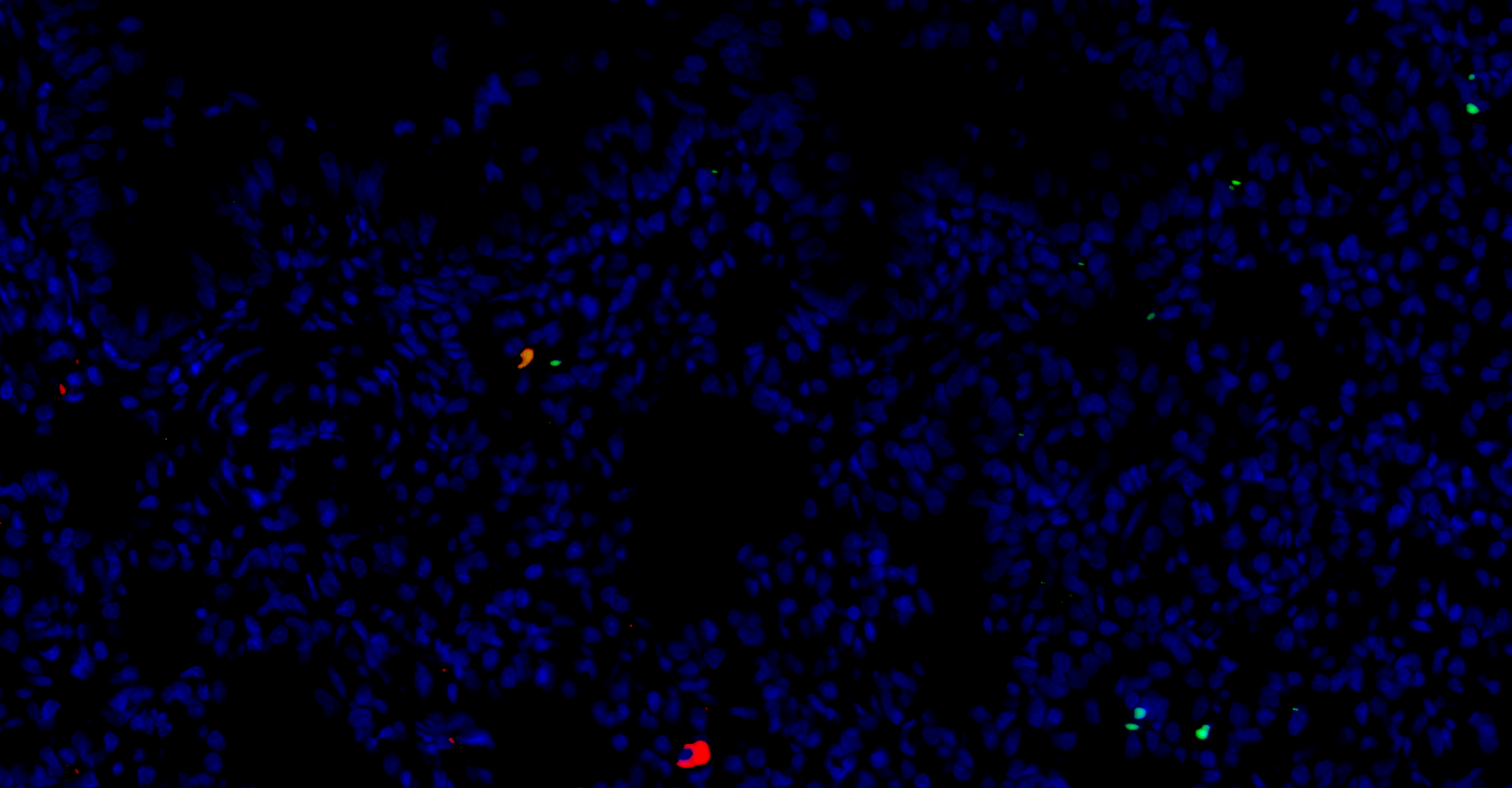

Supplement: Supplementary file 1 [file nutrients-17-02242-s001.zip › Figure S2 Original images/figure6-T-2 TUNEL-LY6G_40.0x.tif]

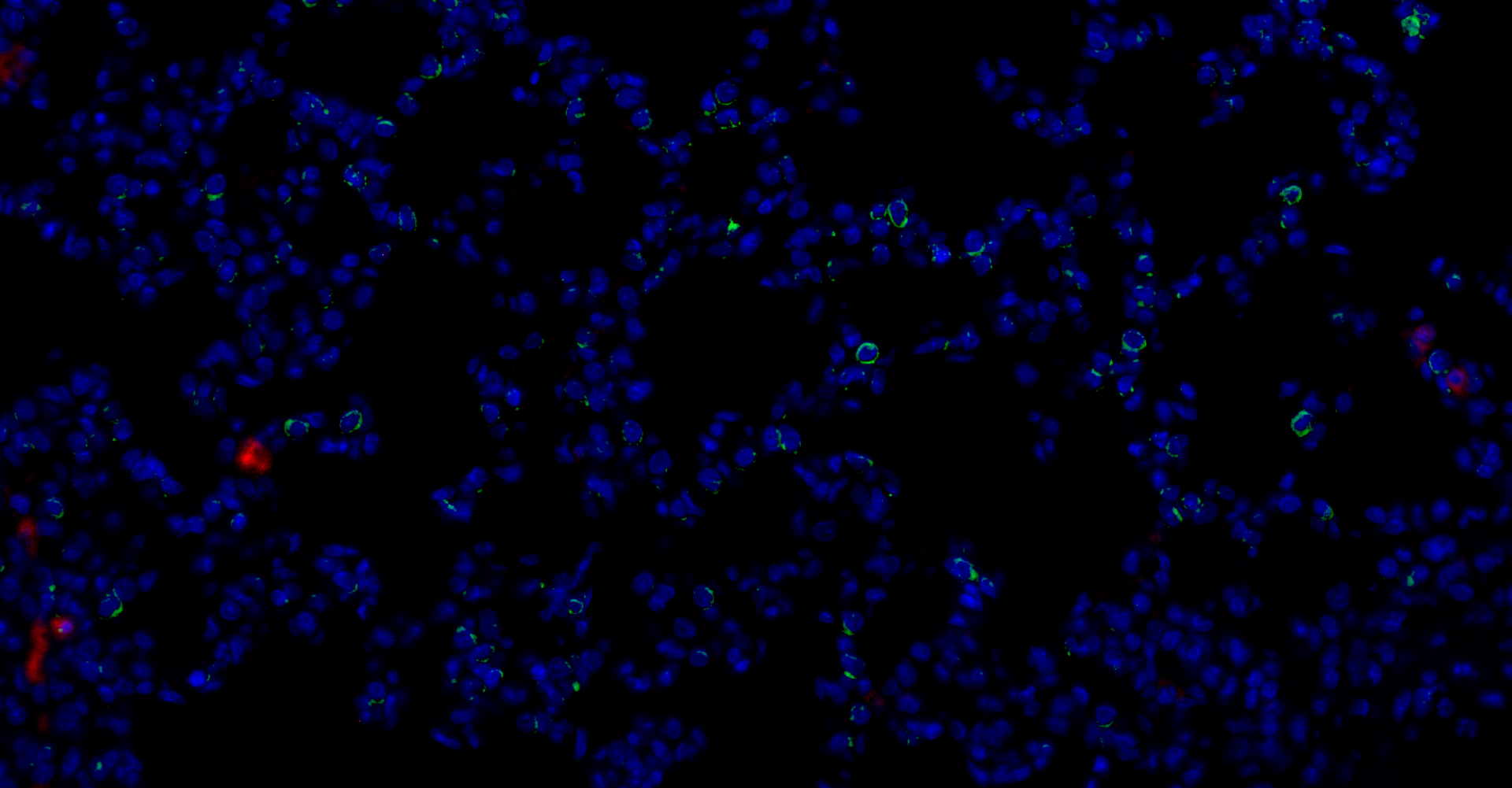

Supplement: Supplementary file 1 [file nutrients-17-02242-s001.zip › Figure S2 Original images/figure6-T-3 LY6G-ACH4_40.0x.tif]

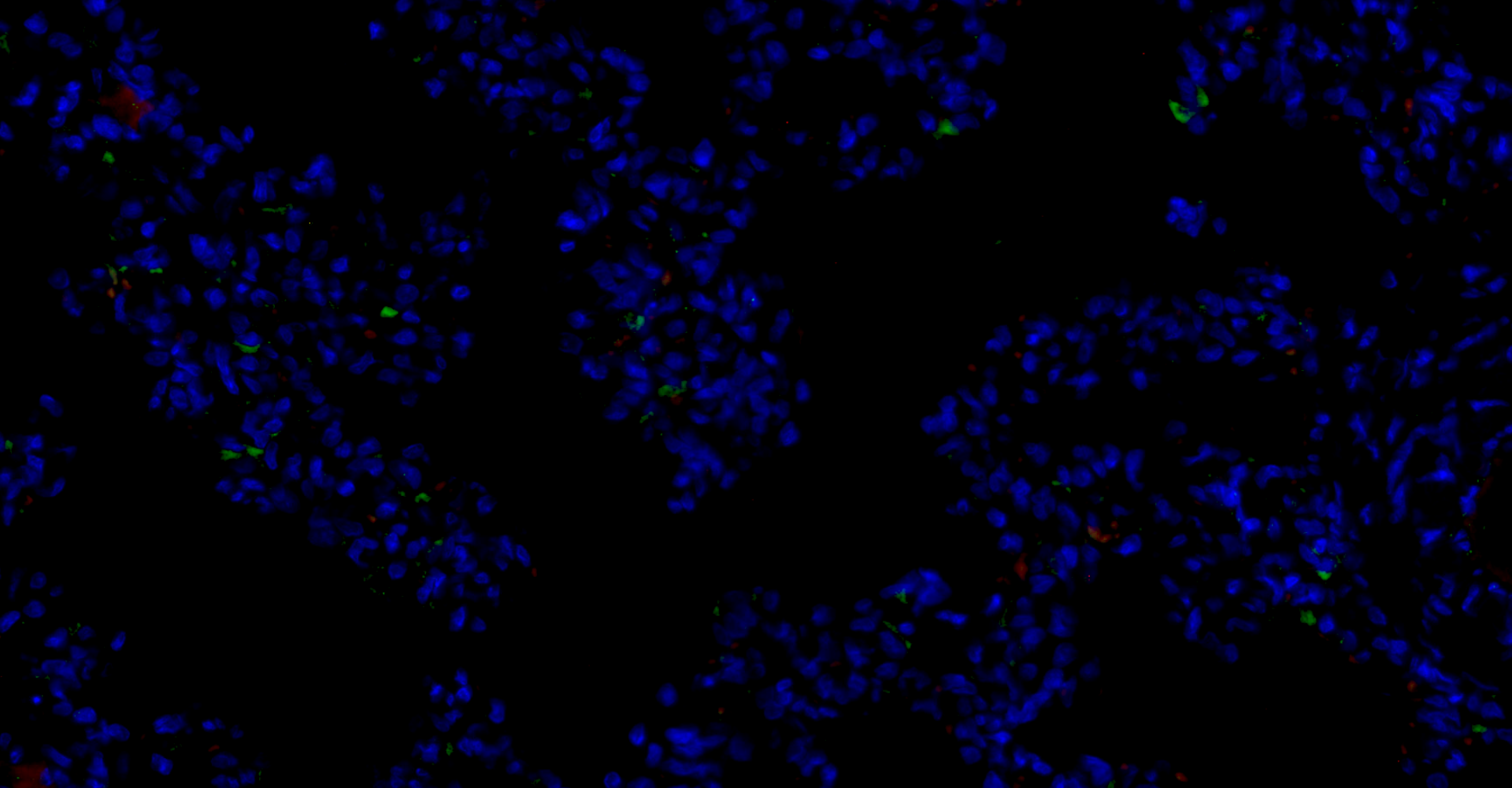

Supplement: Supplementary file 1 [file nutrients-17-02242-s001.zip › Figure S2 Original images/figure6-T-3 LY6G-CITH3_40.0x.tif]

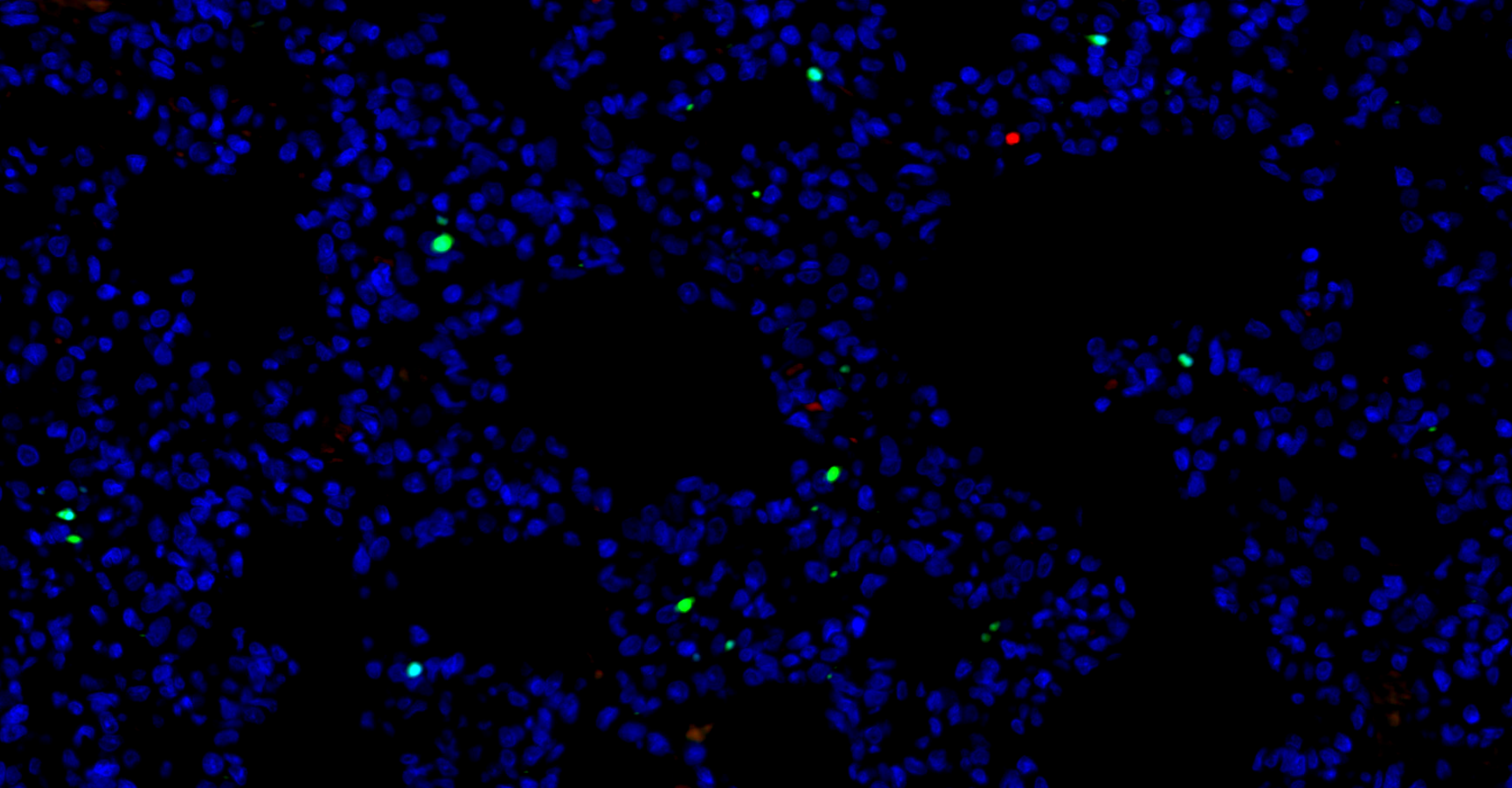

Supplement: Supplementary file 1 [file nutrients-17-02242-s001.zip › Figure S2 Original images/figure6-T-3 TUNEL-LY6G_40.0x.tif]

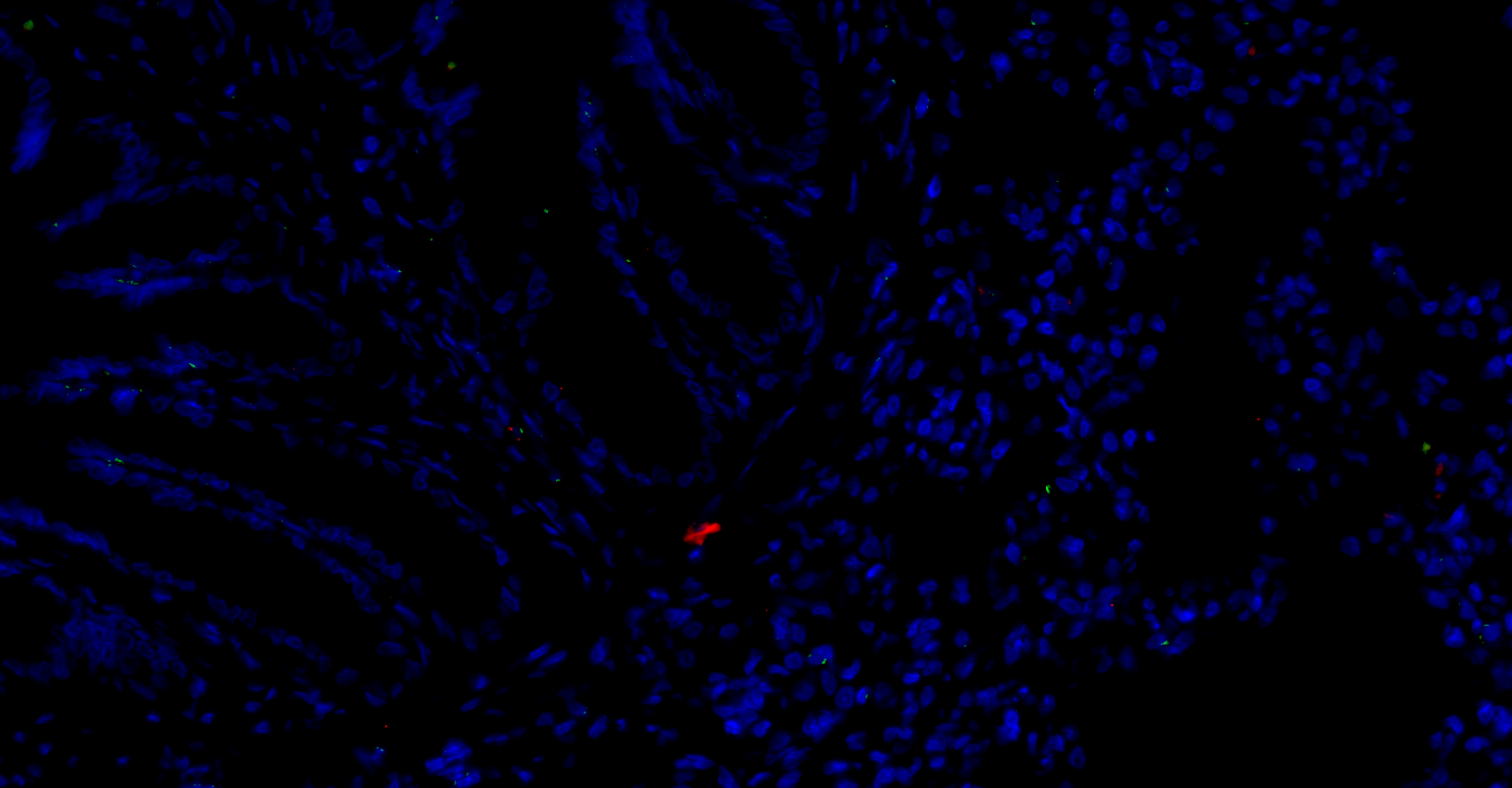

Supplement: Supplementary file 1 [file nutrients-17-02242-s001.zip › Figure S2 Original images/figure6-T-4 LY6G-ACH4_40.0x.tif]

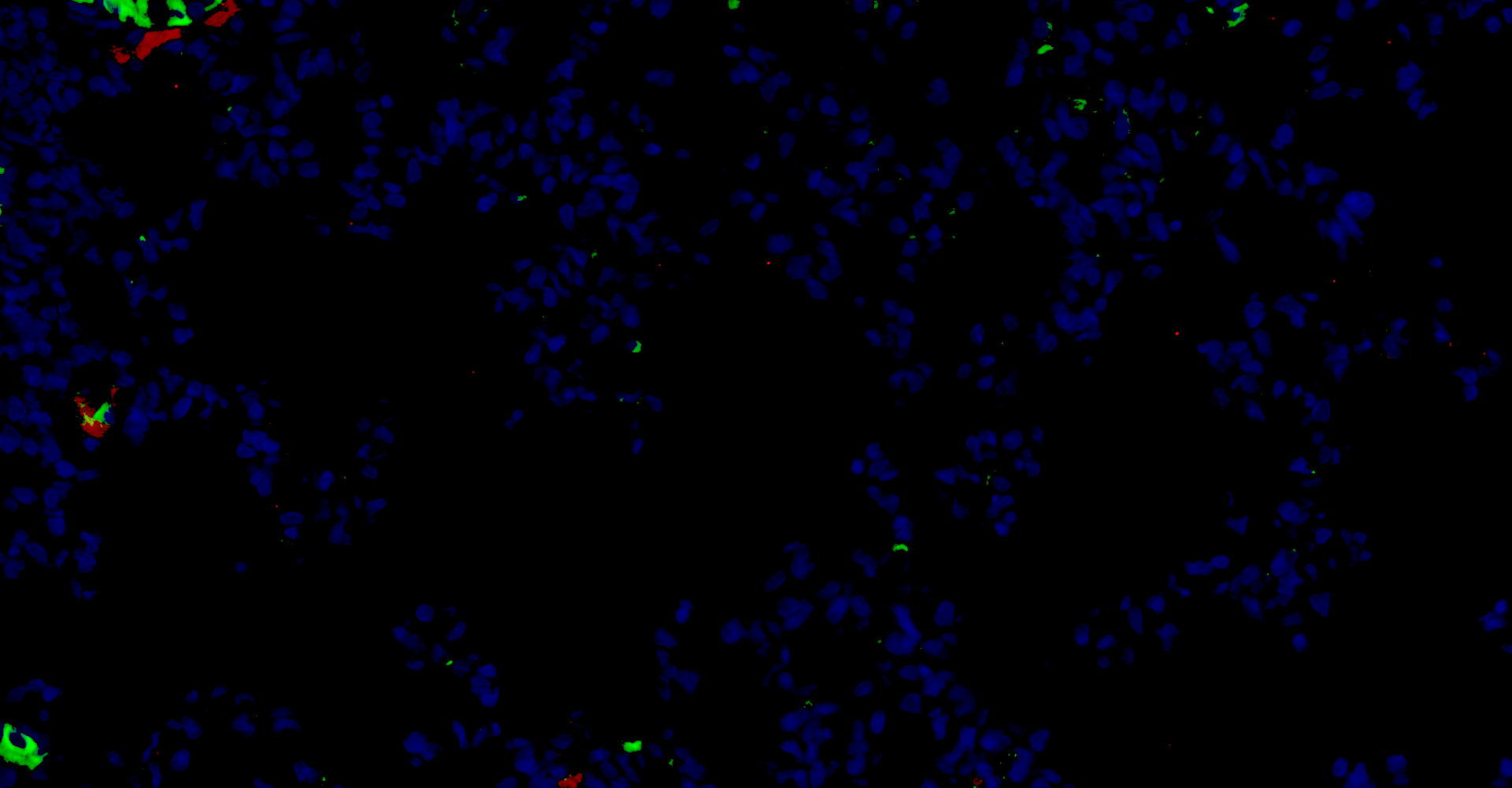

Supplement: Supplementary file 1 [file nutrients-17-02242-s001.zip › Figure S2 Original images/figure6-T-4 LY6G-CITH3_40.0x.tif]

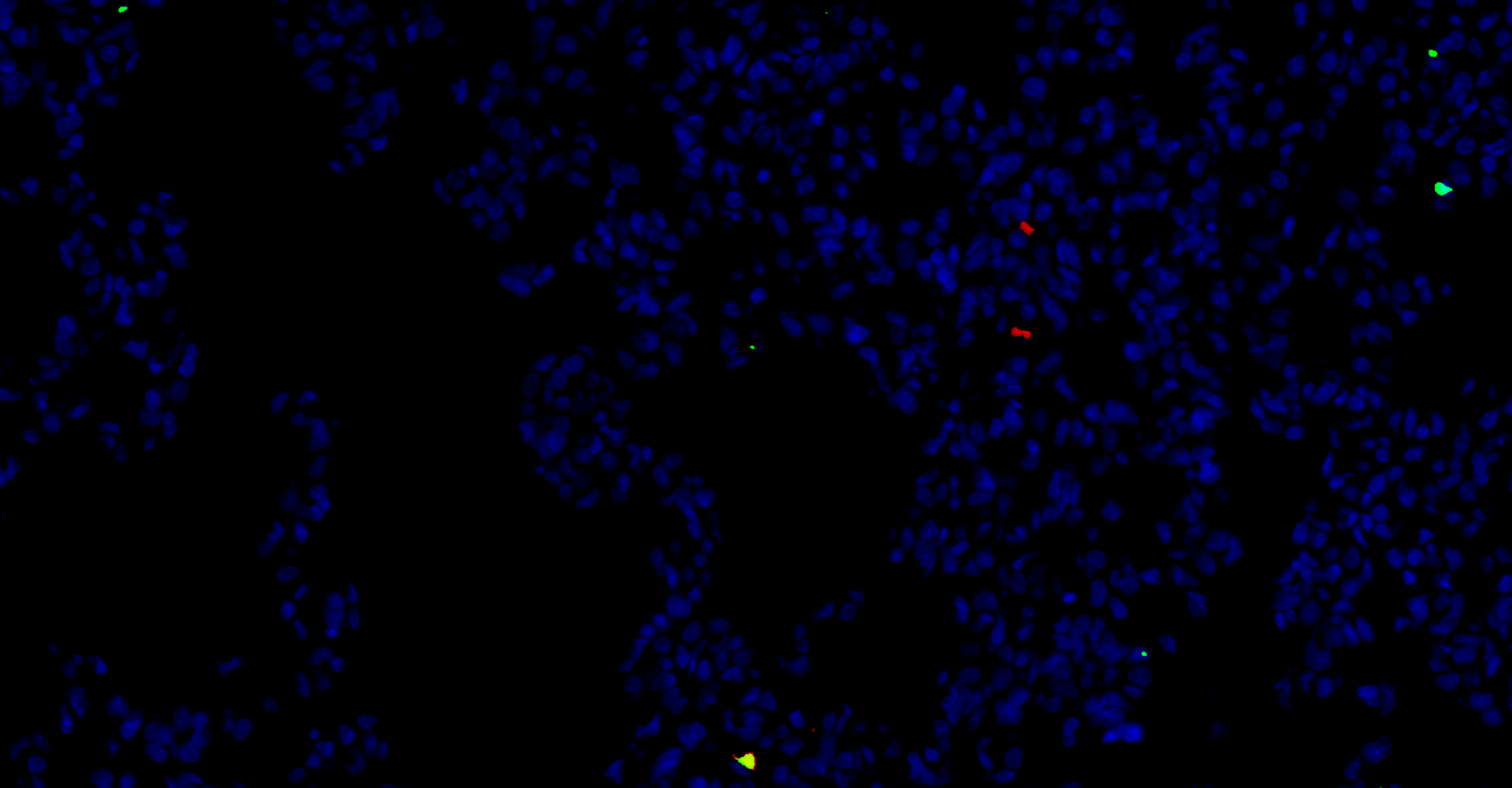

Supplement: Supplementary file 1 [file nutrients-17-02242-s001.zip › Figure S2 Original images/figure6-T-4 TUNEL-LY6G_40.0x.tif]

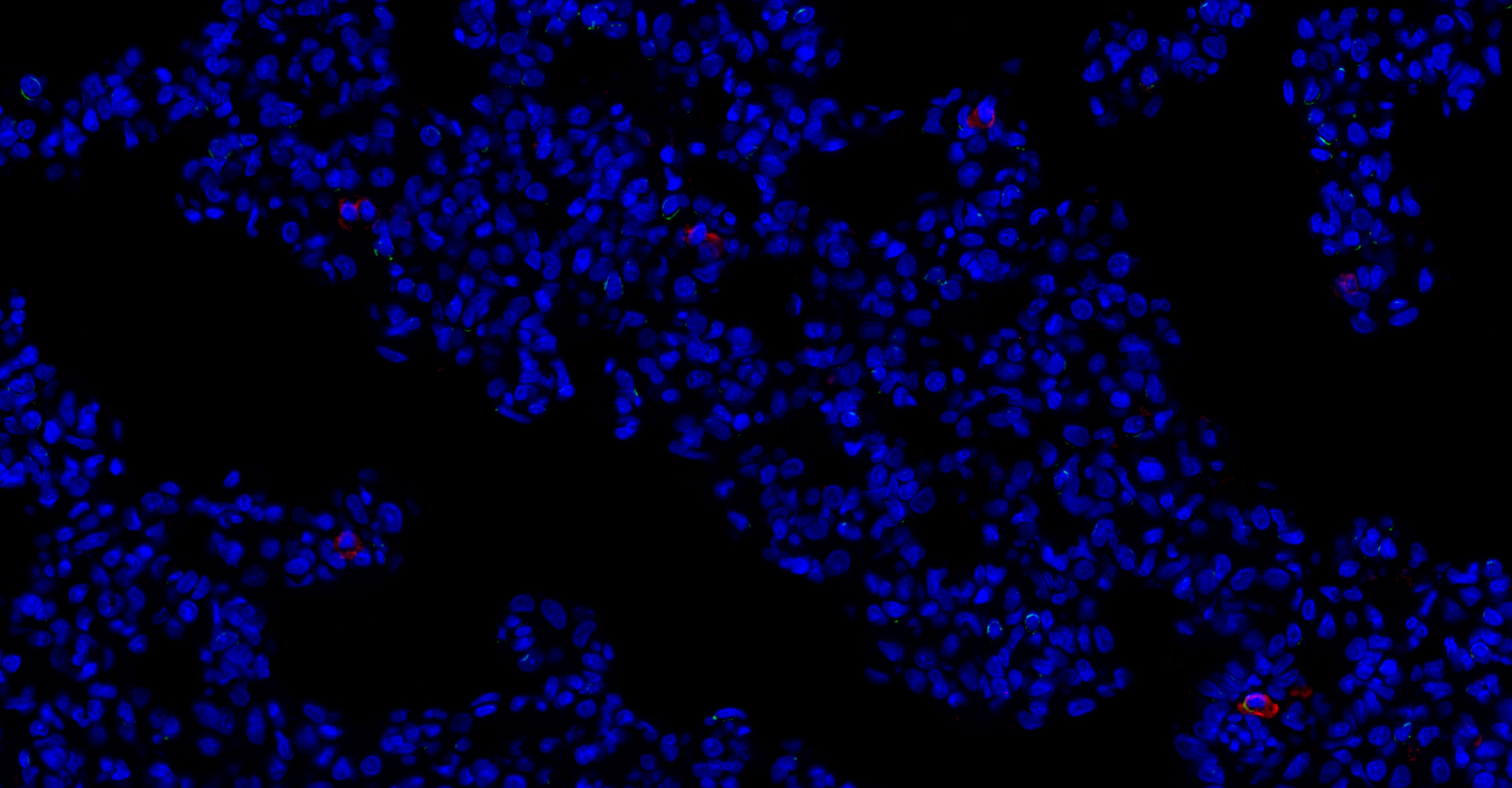

Supplement: Supplementary file 1 [file nutrients-17-02242-s001.zip › Figure S2 Original images/figure6-T-5 LY6G-ACH4_40.0x.tif]

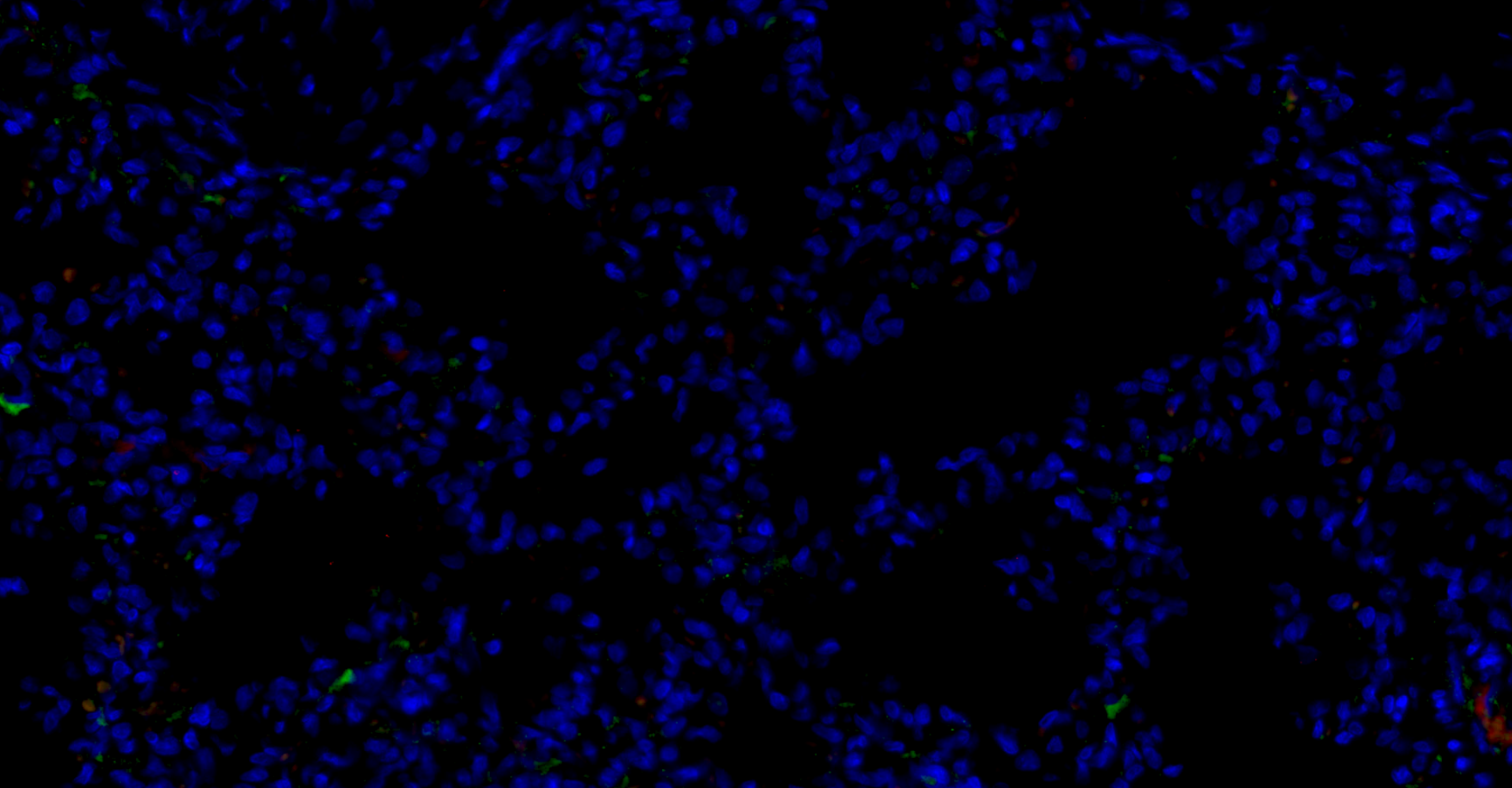

Supplement: Supplementary file 1 [file nutrients-17-02242-s001.zip › Figure S2 Original images/figure6-T-5 LY6G-CITH3_40.0x.tif]

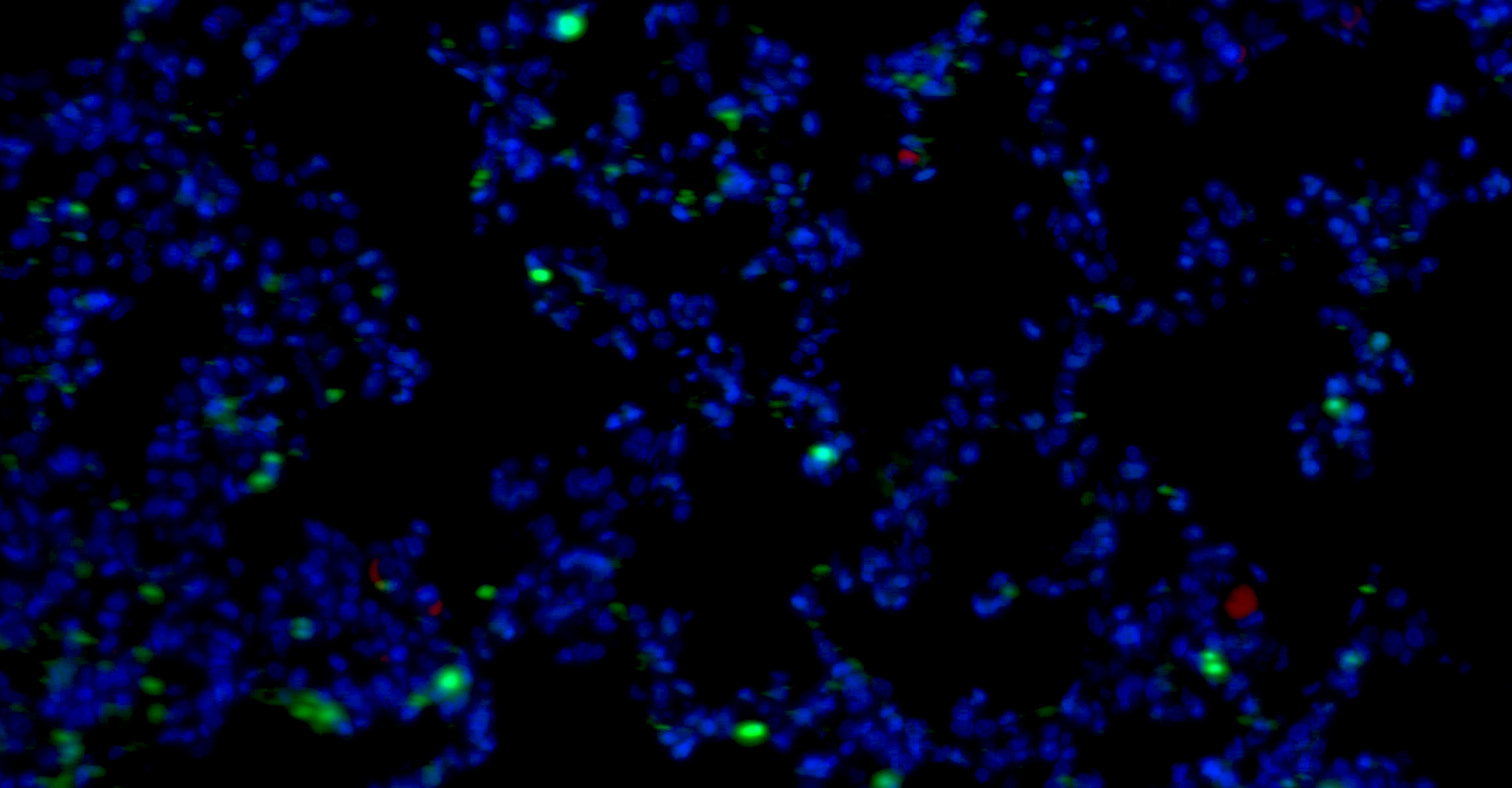

Supplement: Supplementary file 1 [file nutrients-17-02242-s001.zip › Figure S2 Original images/figure6-T-5 TUNEL-LY6G_40.0x.tif]

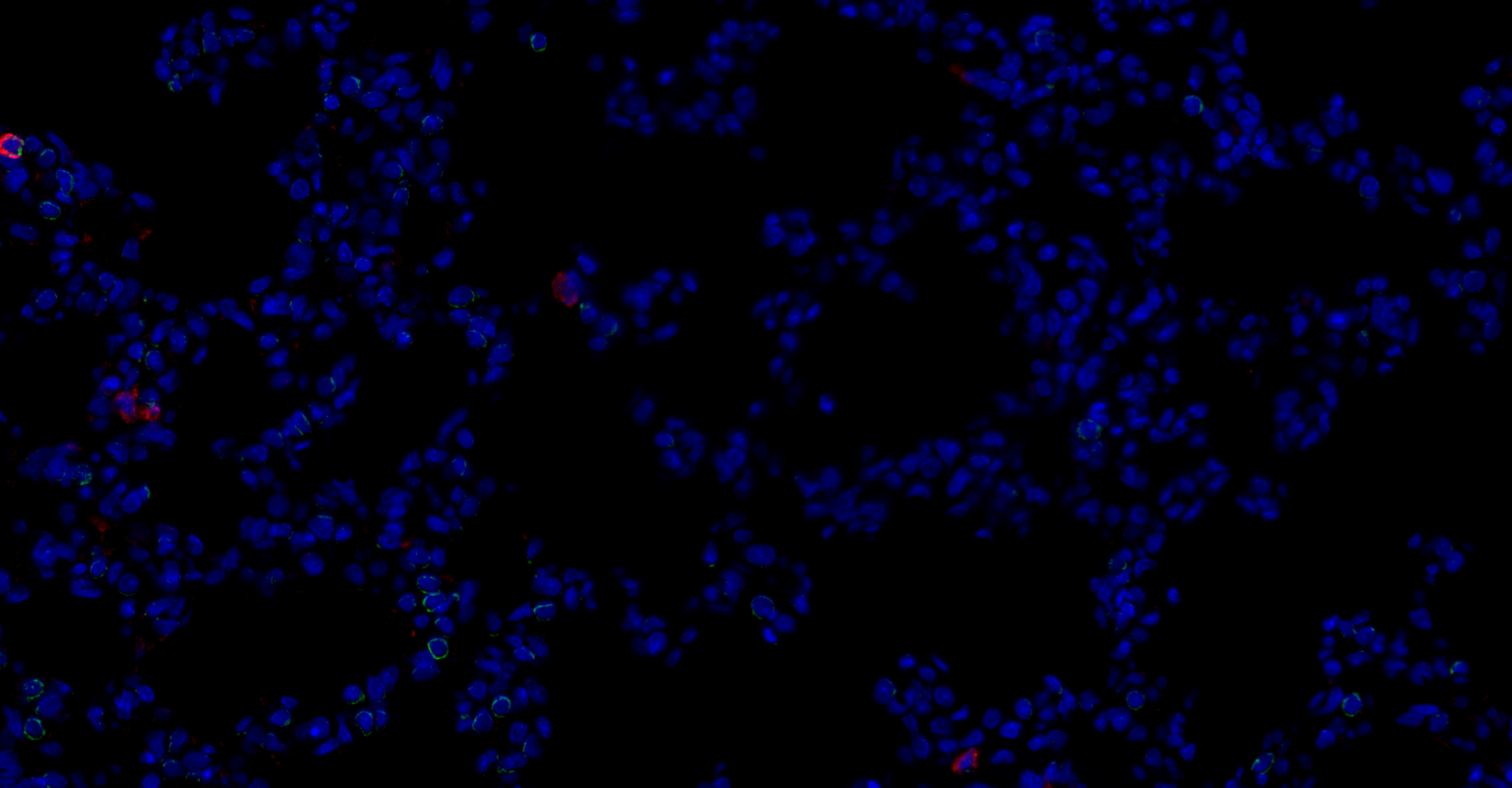

Supplement: Supplementary file 1 [file nutrients-17-02242-s001.zip › Figure S2 Original images/figure6-T-6 LY6G-ACH4_40.0x.tif]

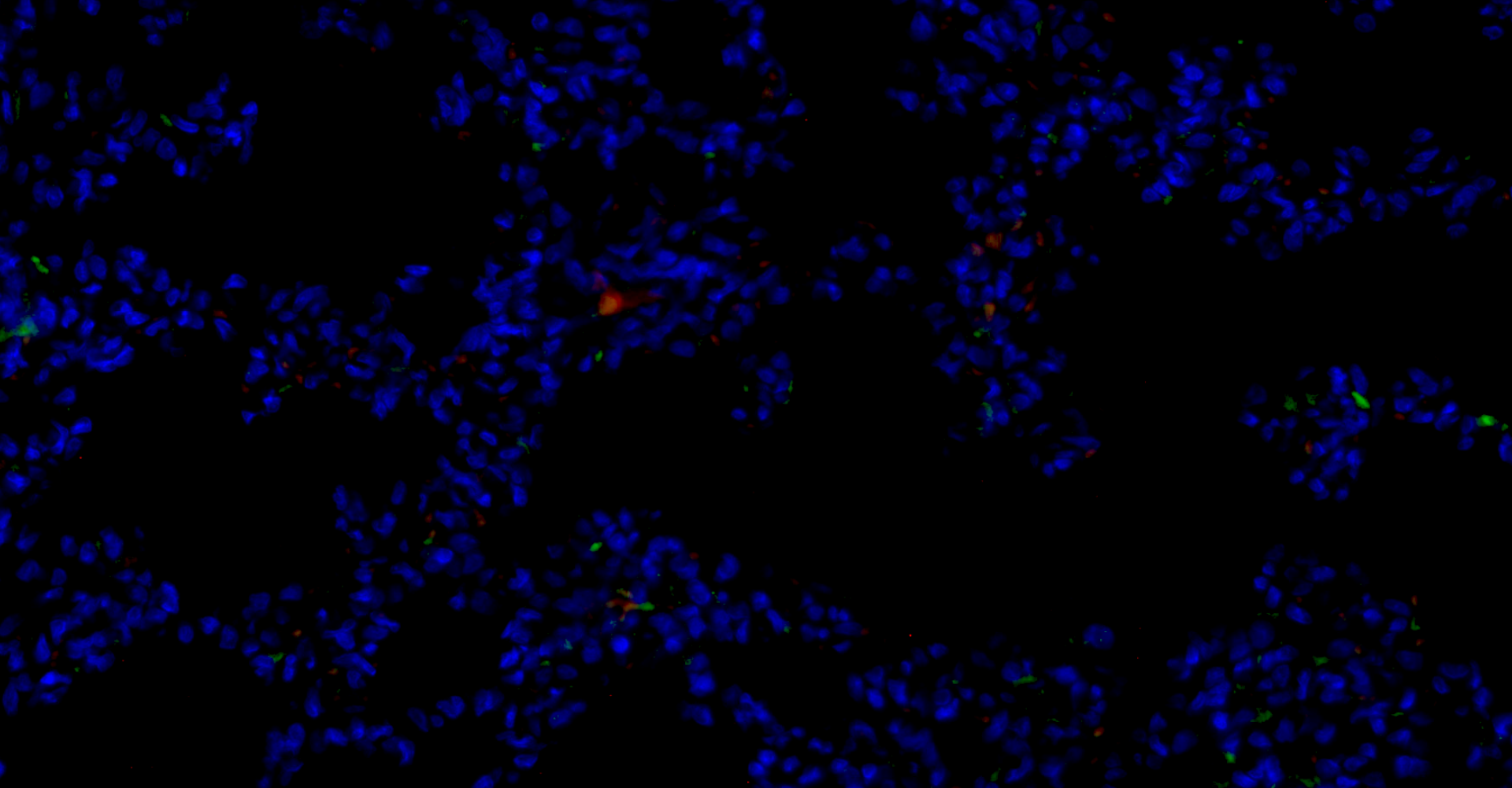

Supplement: Supplementary file 1 [file nutrients-17-02242-s001.zip › Figure S2 Original images/figure6-T-6 LY6G-CITH3_40.0x.tif]

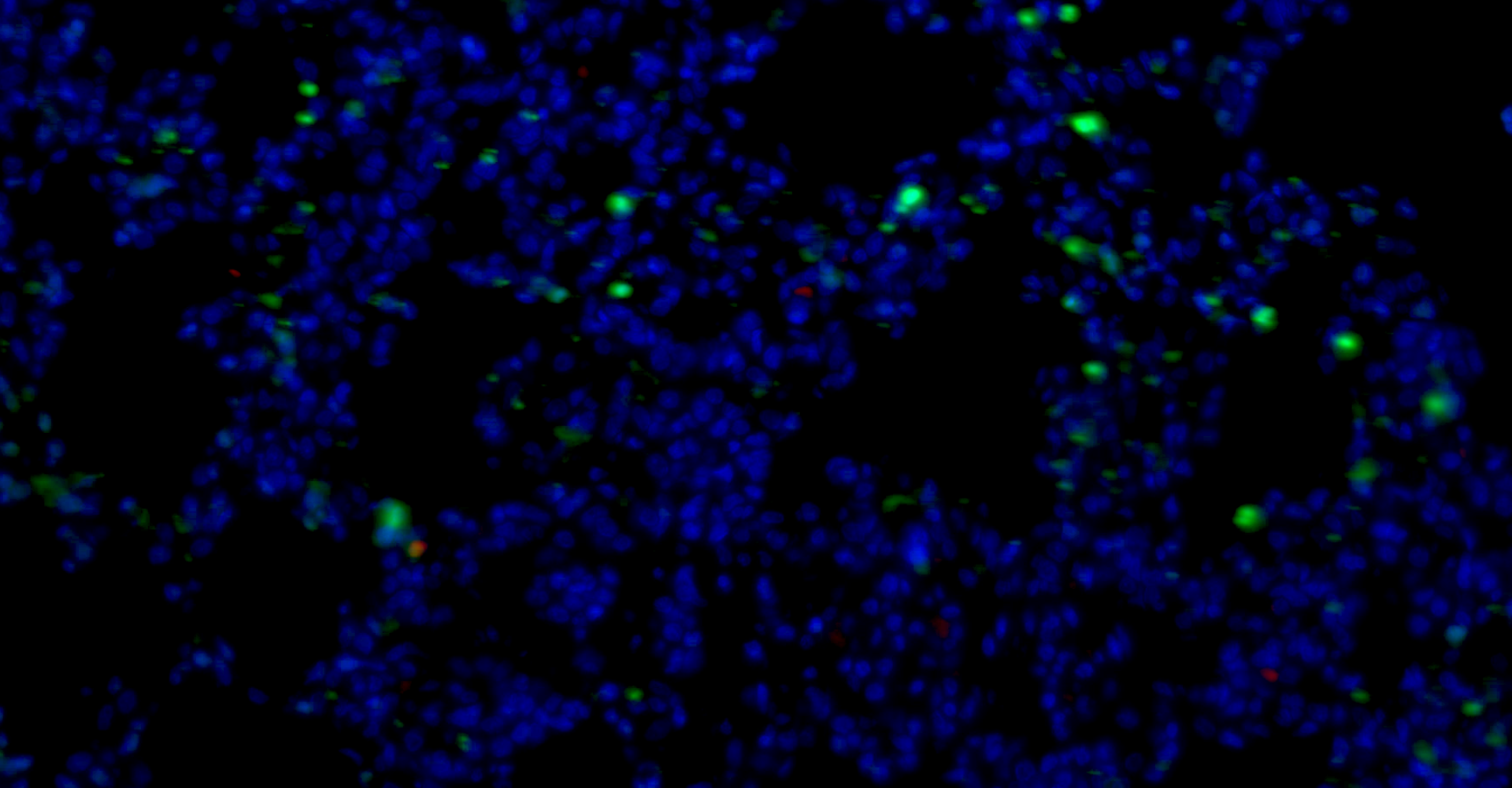

Supplement: Supplementary file 1 [file nutrients-17-02242-s001.zip › Figure S2 Original images/figure6-T-6 TUNEL-LY6G_40.0x.tif]
