# Supplementary figures and images for: High-Calorie Diets Exacerbate Lipopolysaccharide-Induced Pneumonia by Promoting Propionate-Mediated Neutrophil Extracellular Traps (part 1 of 2)
Source: Nutrients. 2025 Jul 7;17(13):2242. doi: 10.3390/nu17132242 (PMC12251575; doi:10.3390/nu17132242)

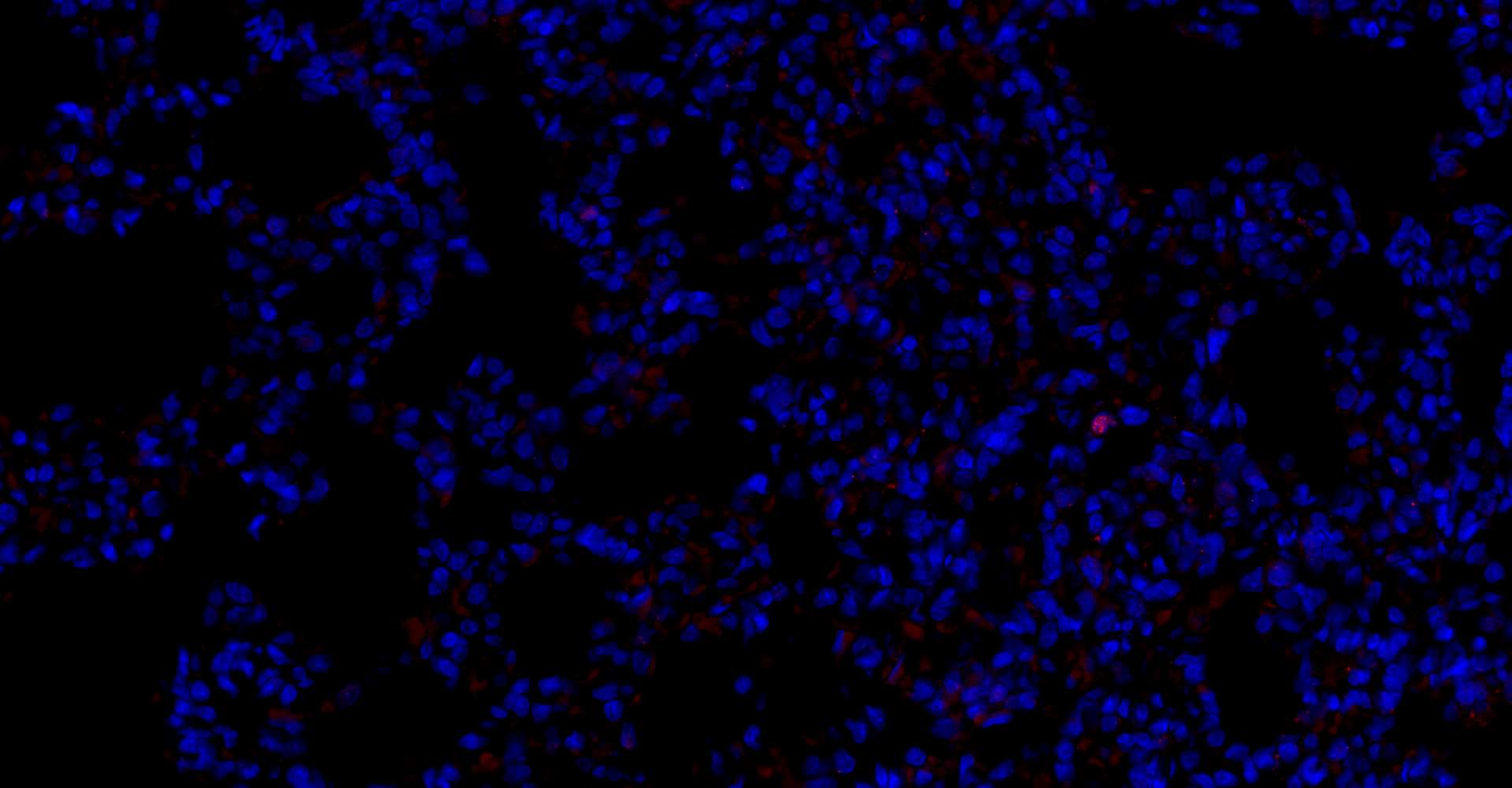

Supplement: Supplementary file 1 [file nutrients-17-02242-s001.zip › Figure S2 Original images/figure2-G-1 citH3 IF_40.0x.tif]

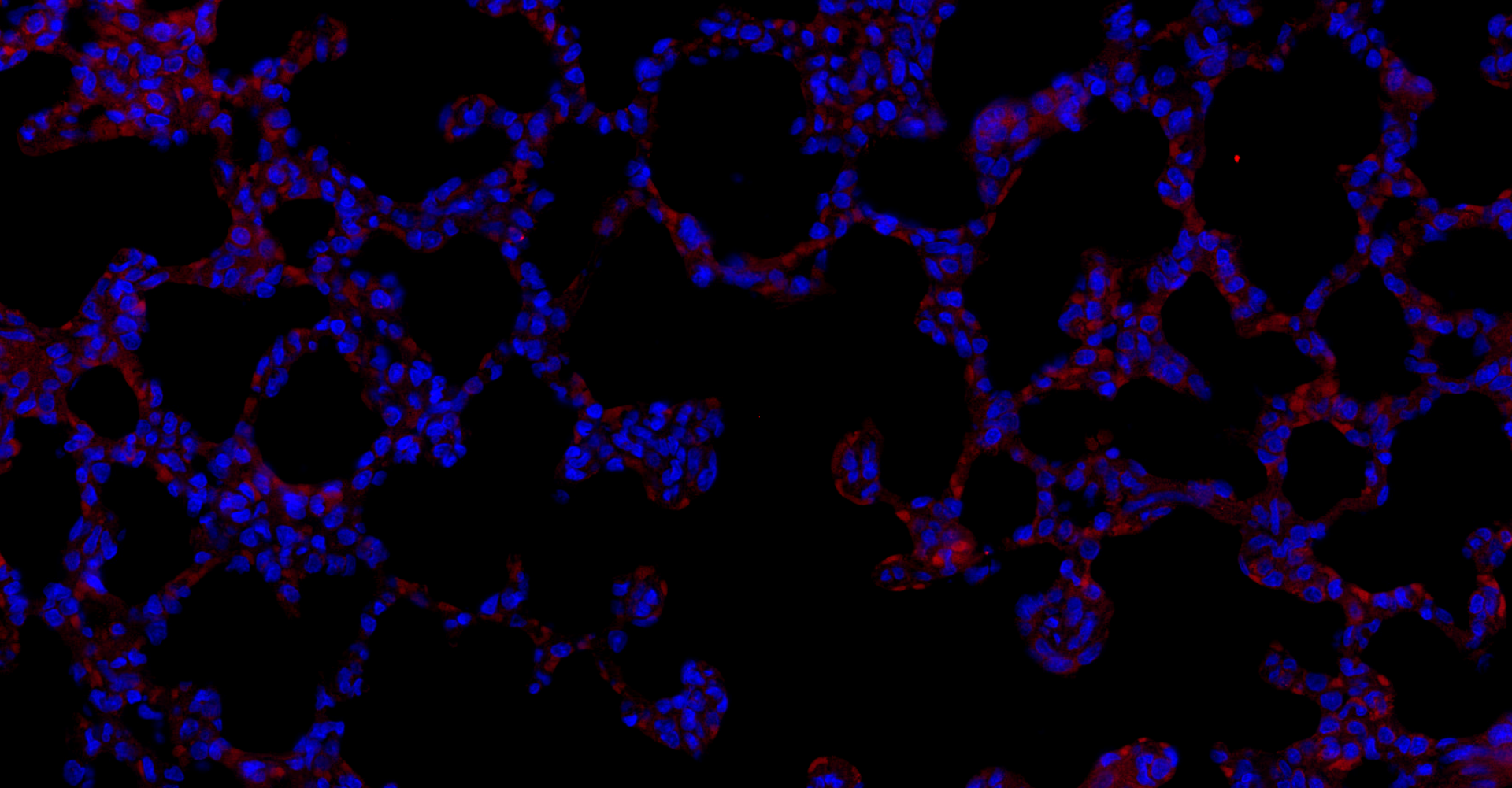

Supplement: Supplementary file 1 [file nutrients-17-02242-s001.zip › Figure S2 Original images/figure2-G-1 ly6g IF_40.0x.tif]

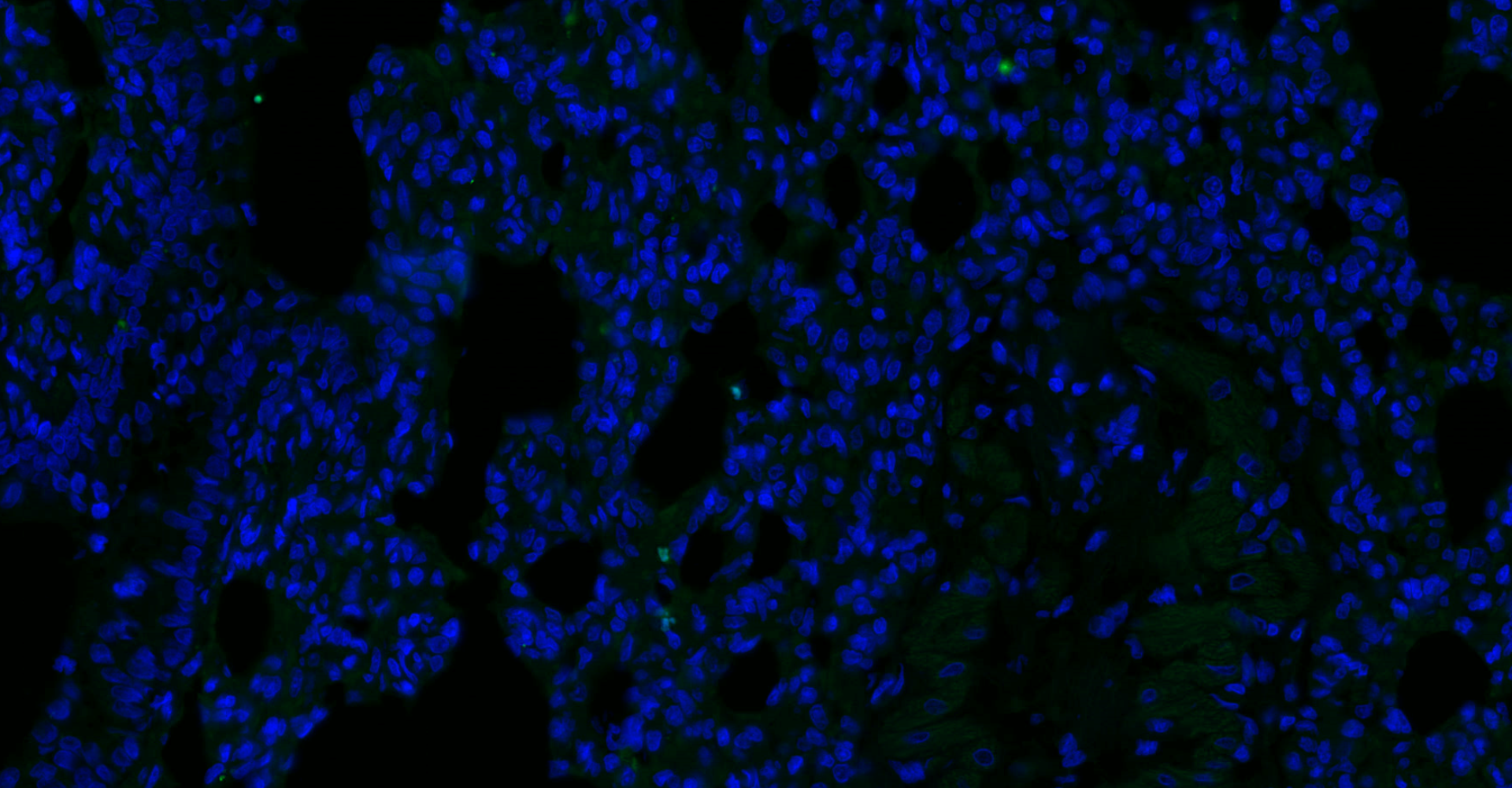

Supplement: Supplementary file 1 [file nutrients-17-02242-s001.zip › Figure S2 Original images/figure2-G-1 tunel IF_40.0x.tif]

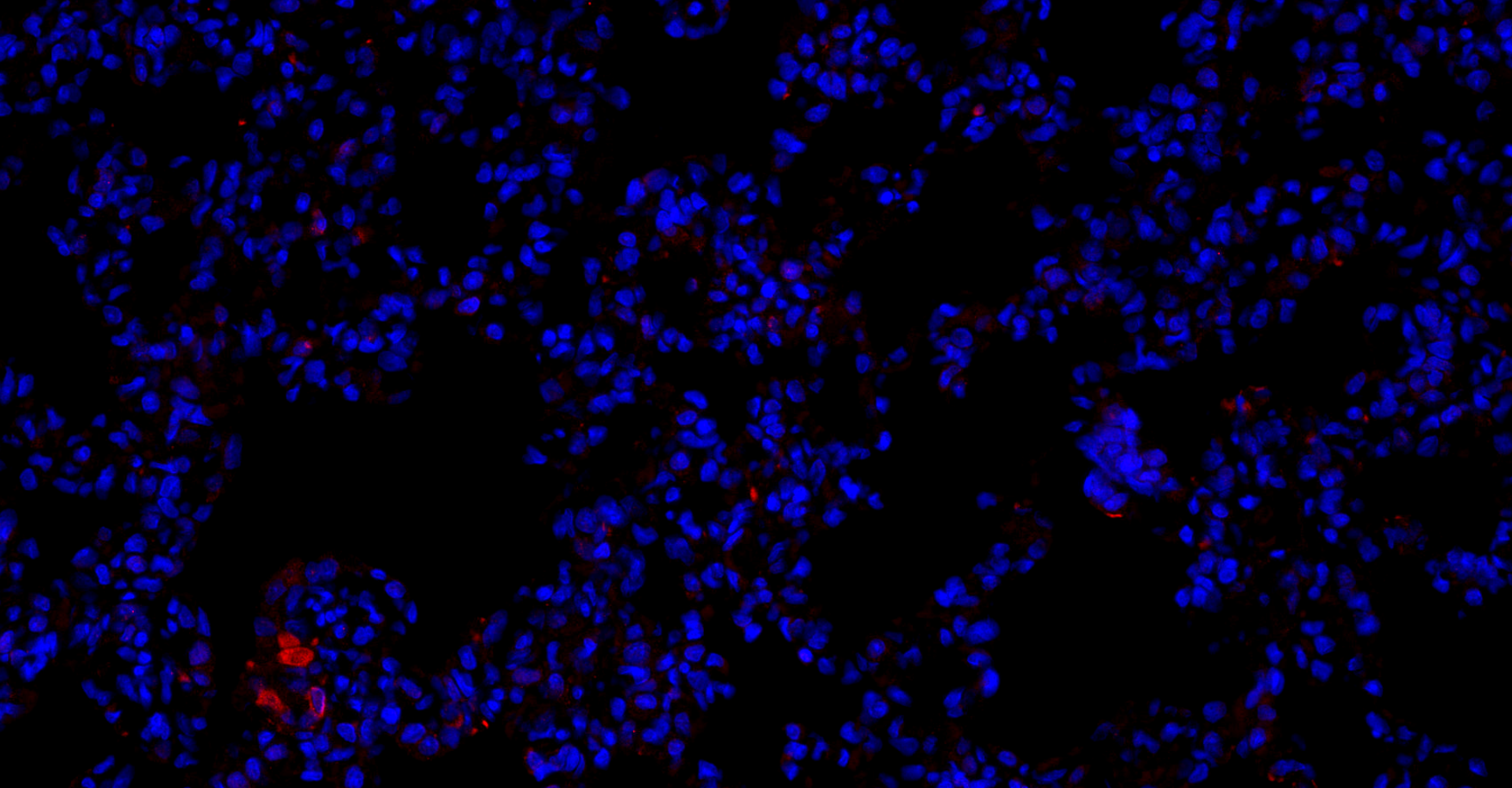

Supplement: Supplementary file 1 [file nutrients-17-02242-s001.zip › Figure S2 Original images/figure2-G-2 citH3 IF_40.0x.tif]

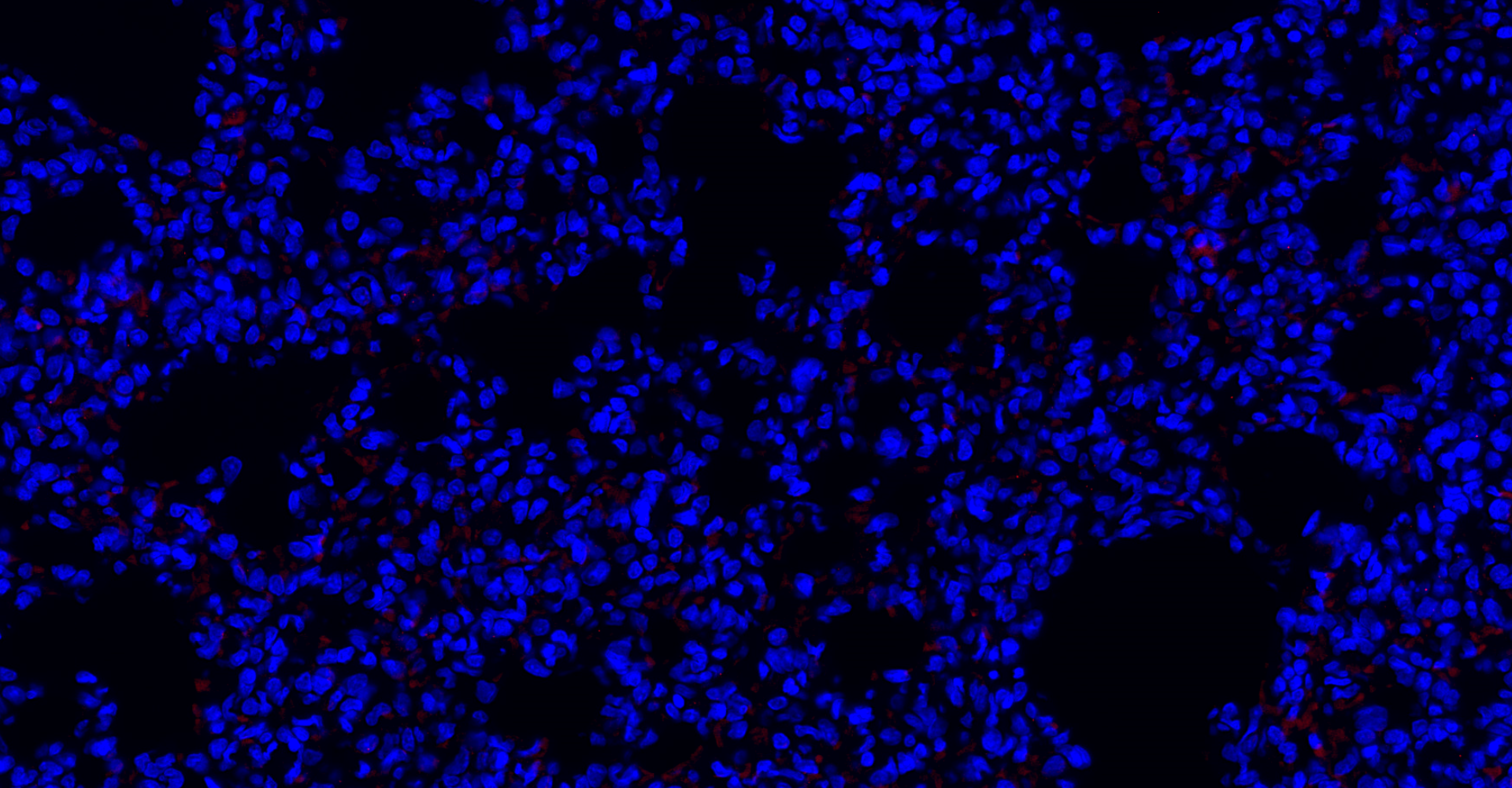

Supplement: Supplementary file 1 [file nutrients-17-02242-s001.zip › Figure S2 Original images/figure2-G-2 ly6g IF_40.0x.tif]

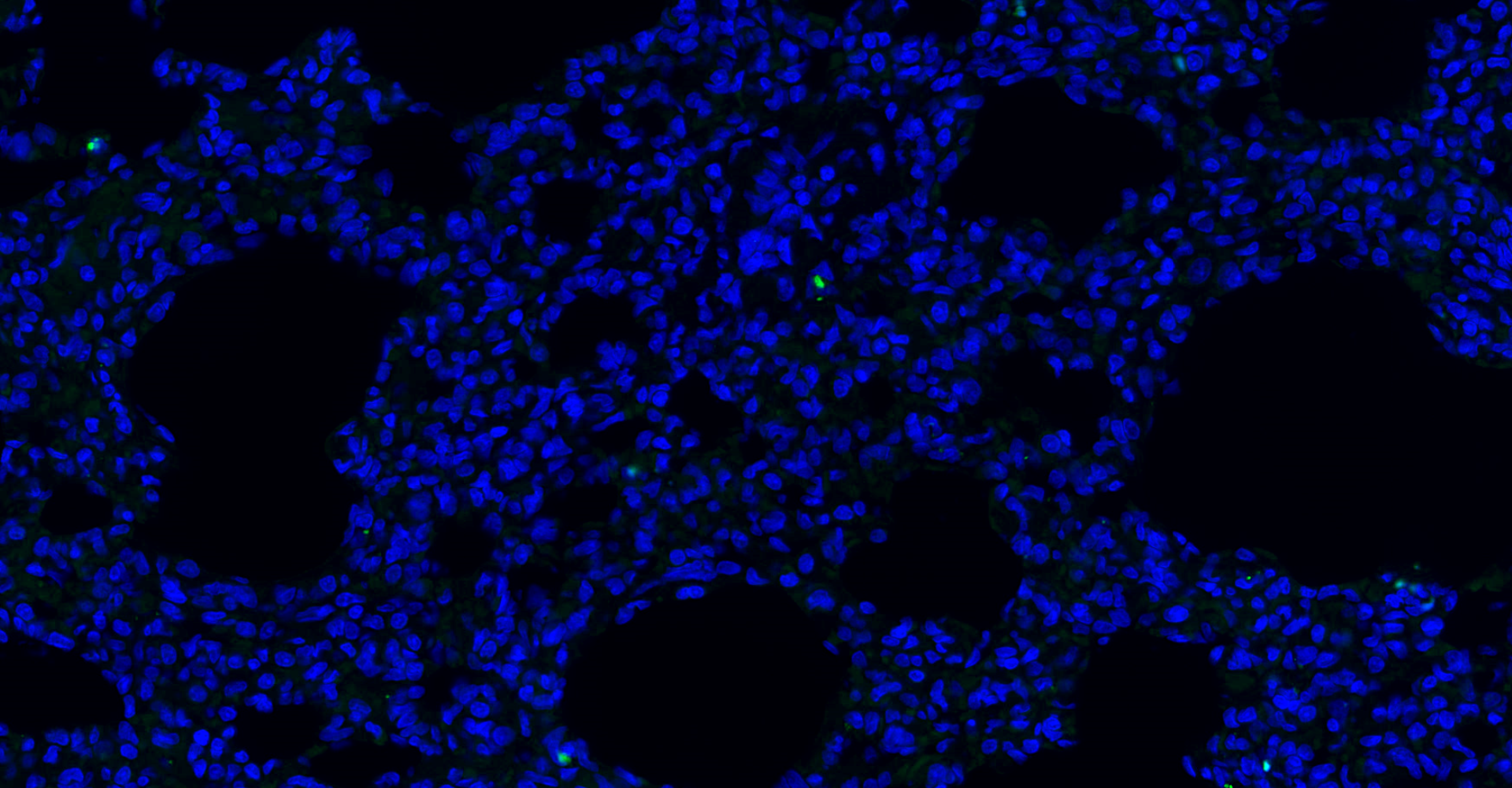

Supplement: Supplementary file 1 [file nutrients-17-02242-s001.zip › Figure S2 Original images/figure2-G-2 tunel IF_40.0x.tif]

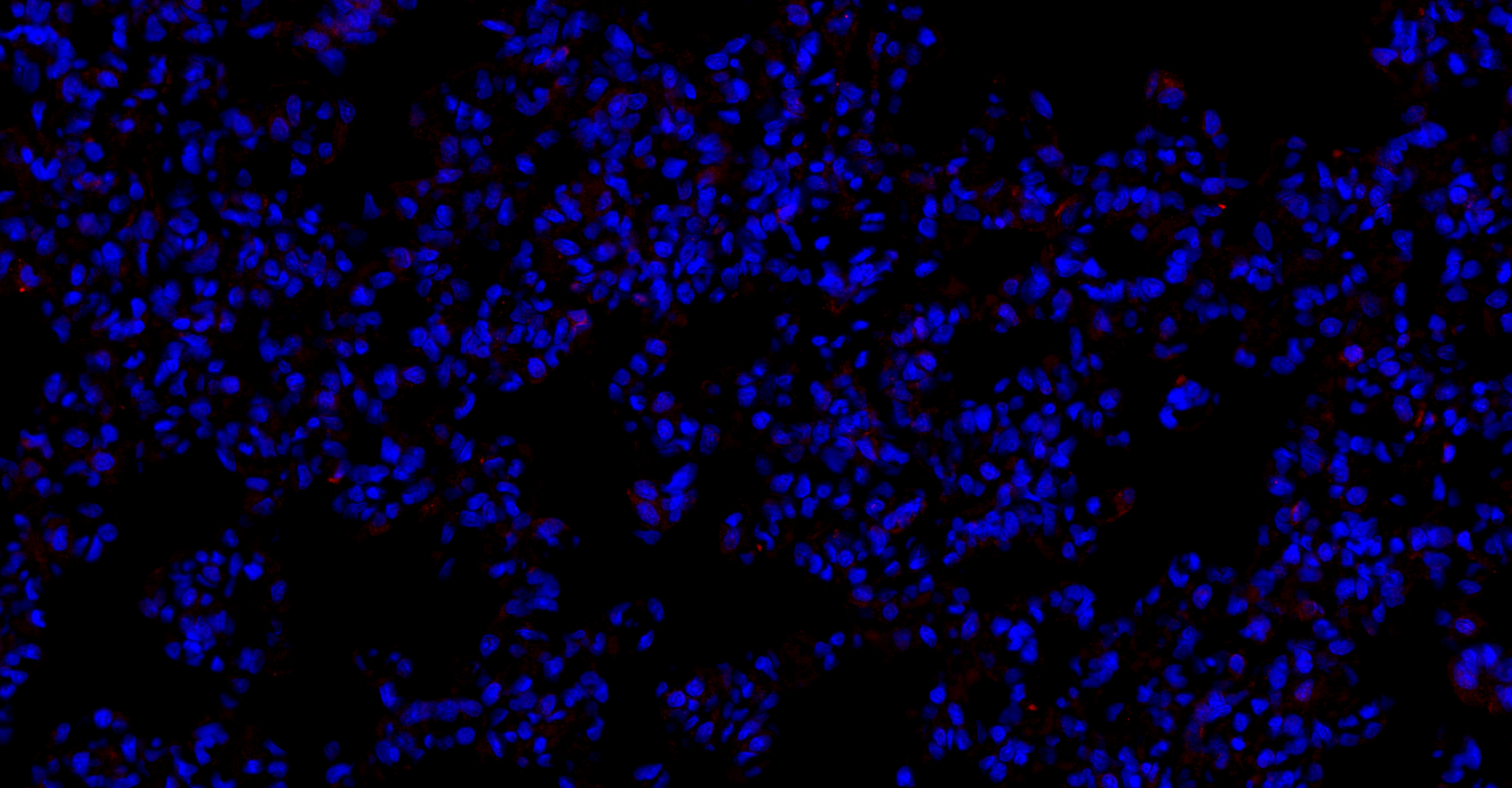

Supplement: Supplementary file 1 [file nutrients-17-02242-s001.zip › Figure S2 Original images/figure2-G-3 citH3 IF_40.0x.tif]

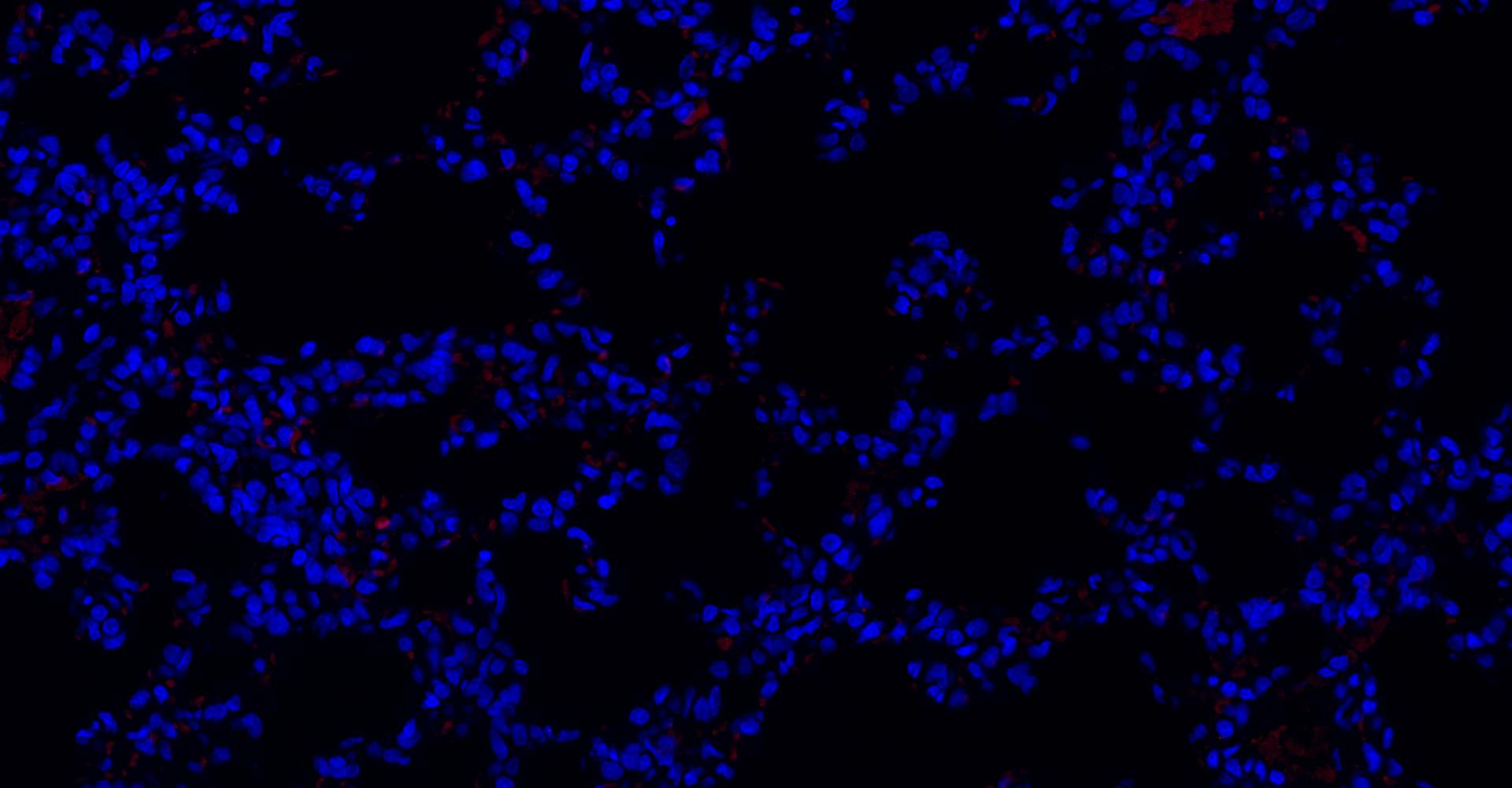

Supplement: Supplementary file 1 [file nutrients-17-02242-s001.zip › Figure S2 Original images/figure2-G-3 ly6g IF_40.0x.tif]

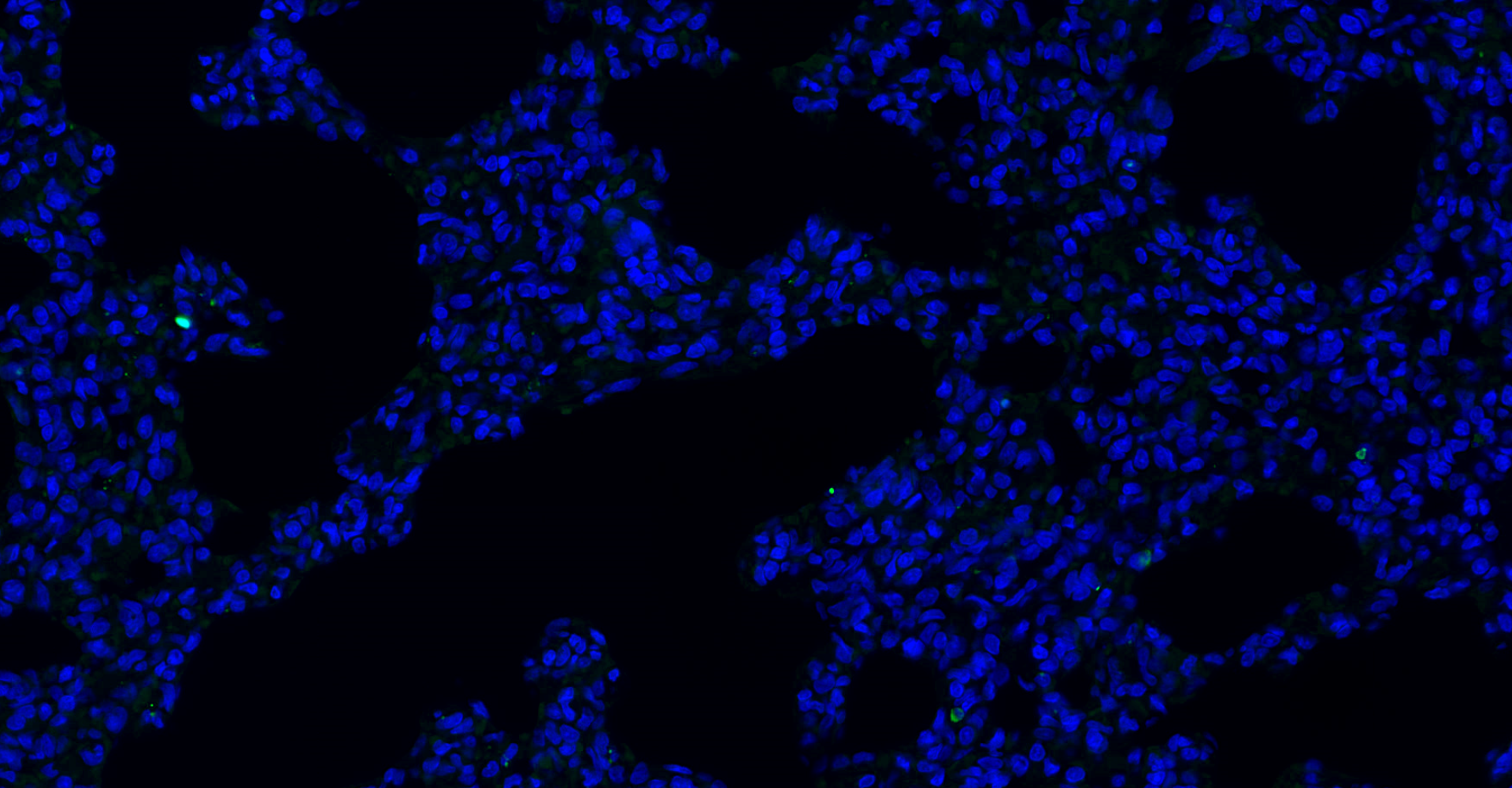

Supplement: Supplementary file 1 [file nutrients-17-02242-s001.zip › Figure S2 Original images/figure2-G-3 tunel IF_40.0x.tif]

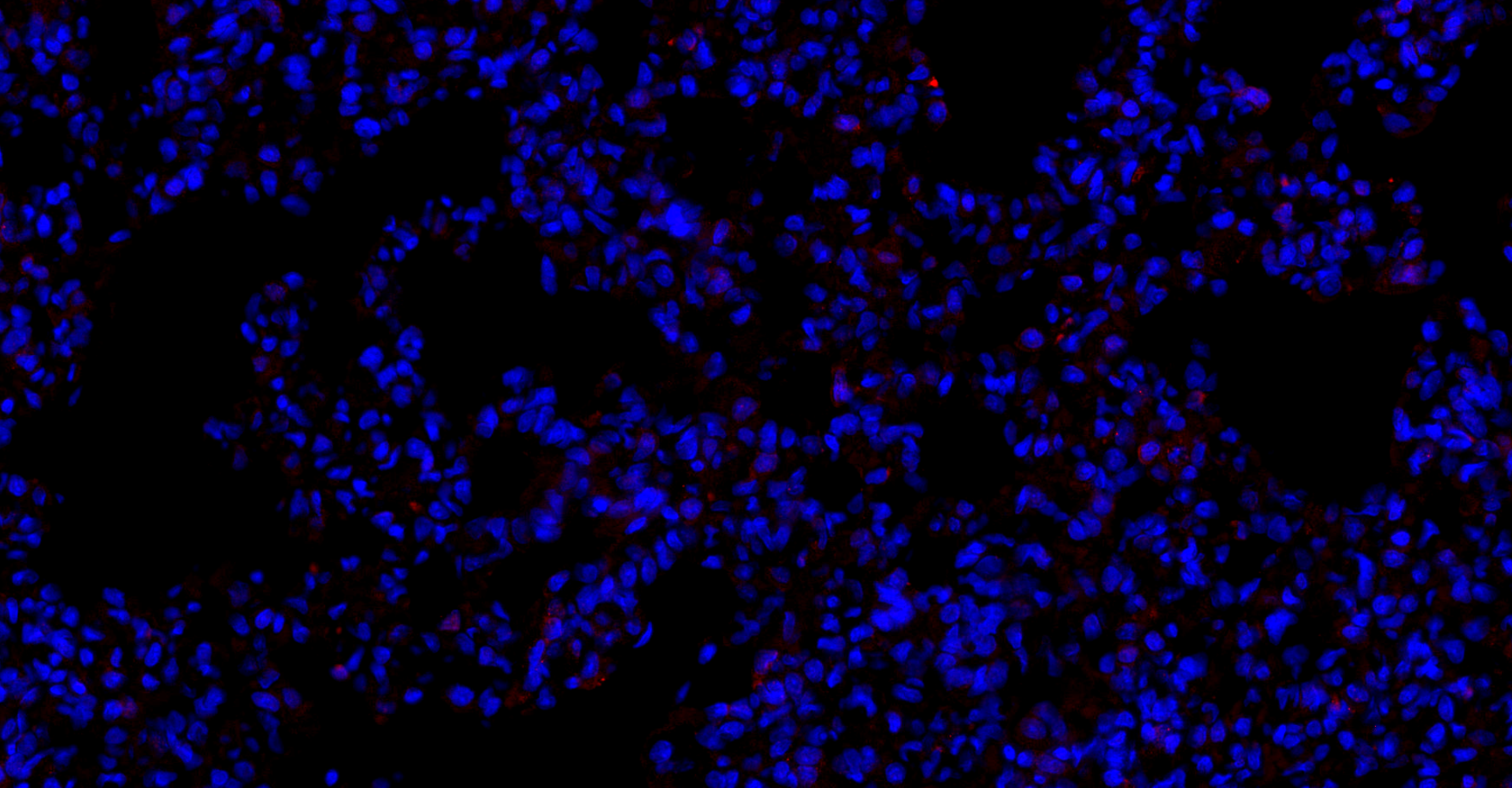

Supplement: Supplementary file 1 [file nutrients-17-02242-s001.zip › Figure S2 Original images/figure2-G-4 citH3 IF_40.0x.tif]

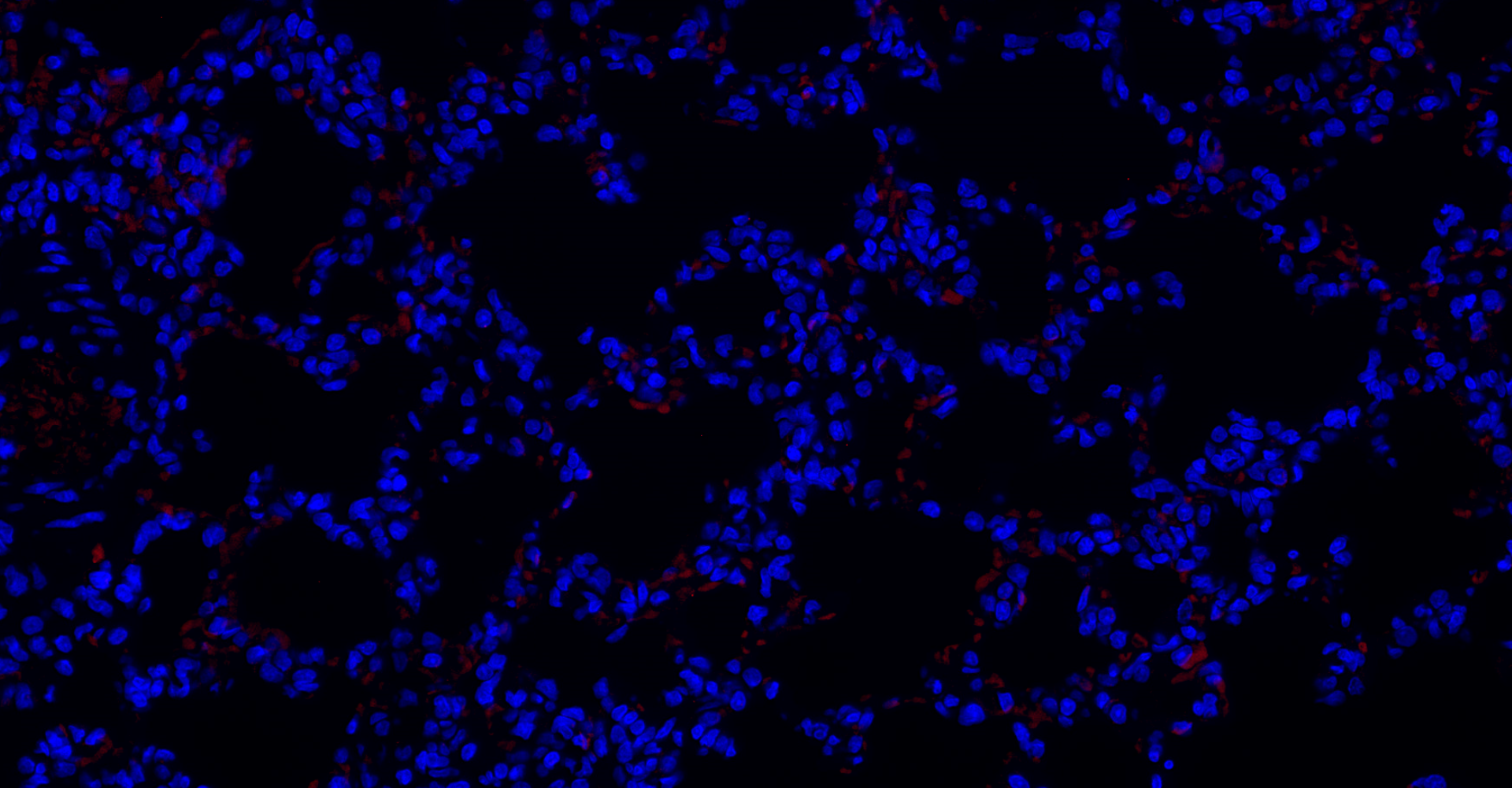

Supplement: Supplementary file 1 [file nutrients-17-02242-s001.zip › Figure S2 Original images/figure2-G-4 ly6g IF_40.0x.tif]

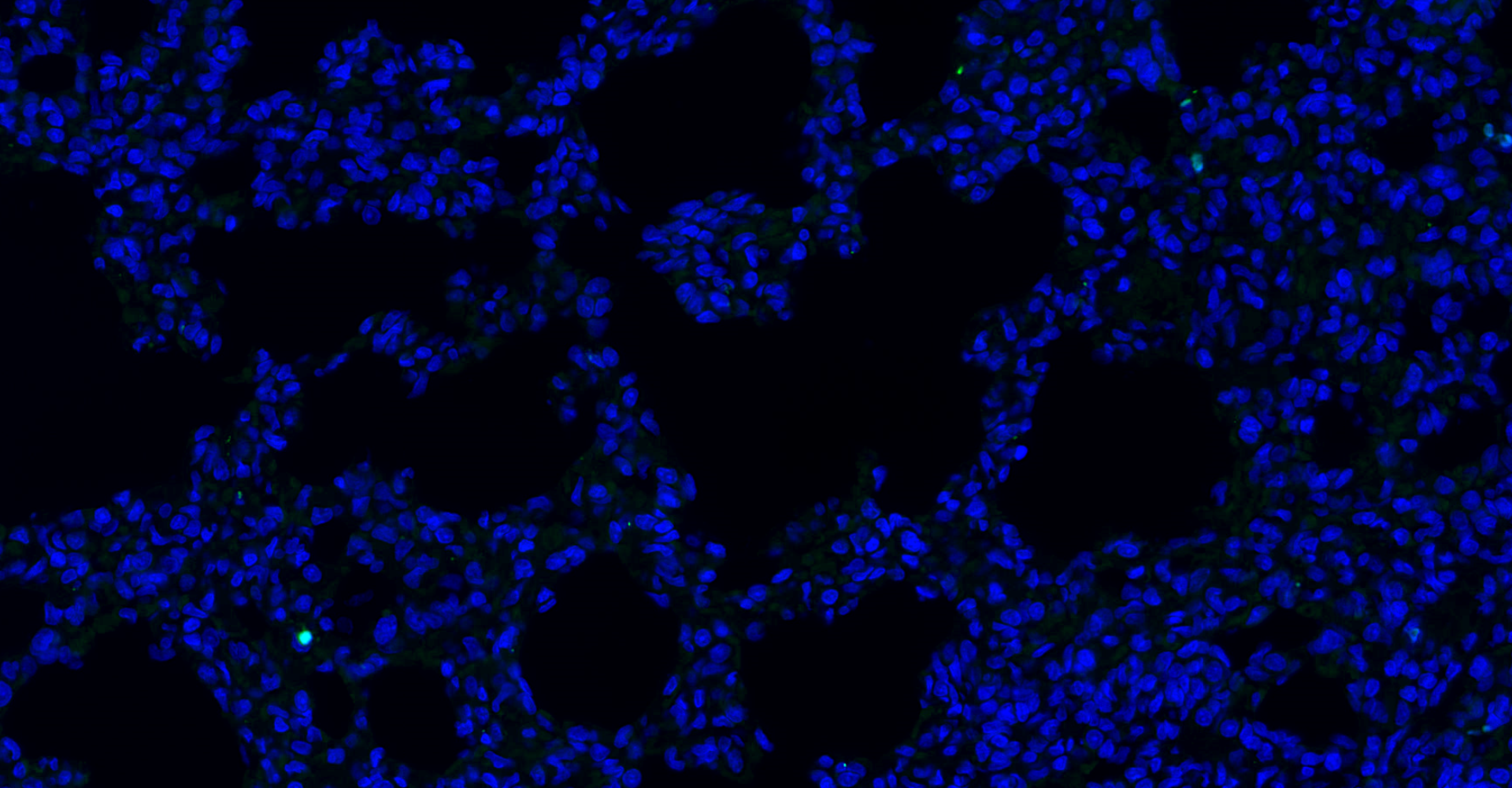

Supplement: Supplementary file 1 [file nutrients-17-02242-s001.zip › Figure S2 Original images/figure2-G-4 tunel IF_40.0x.tif]

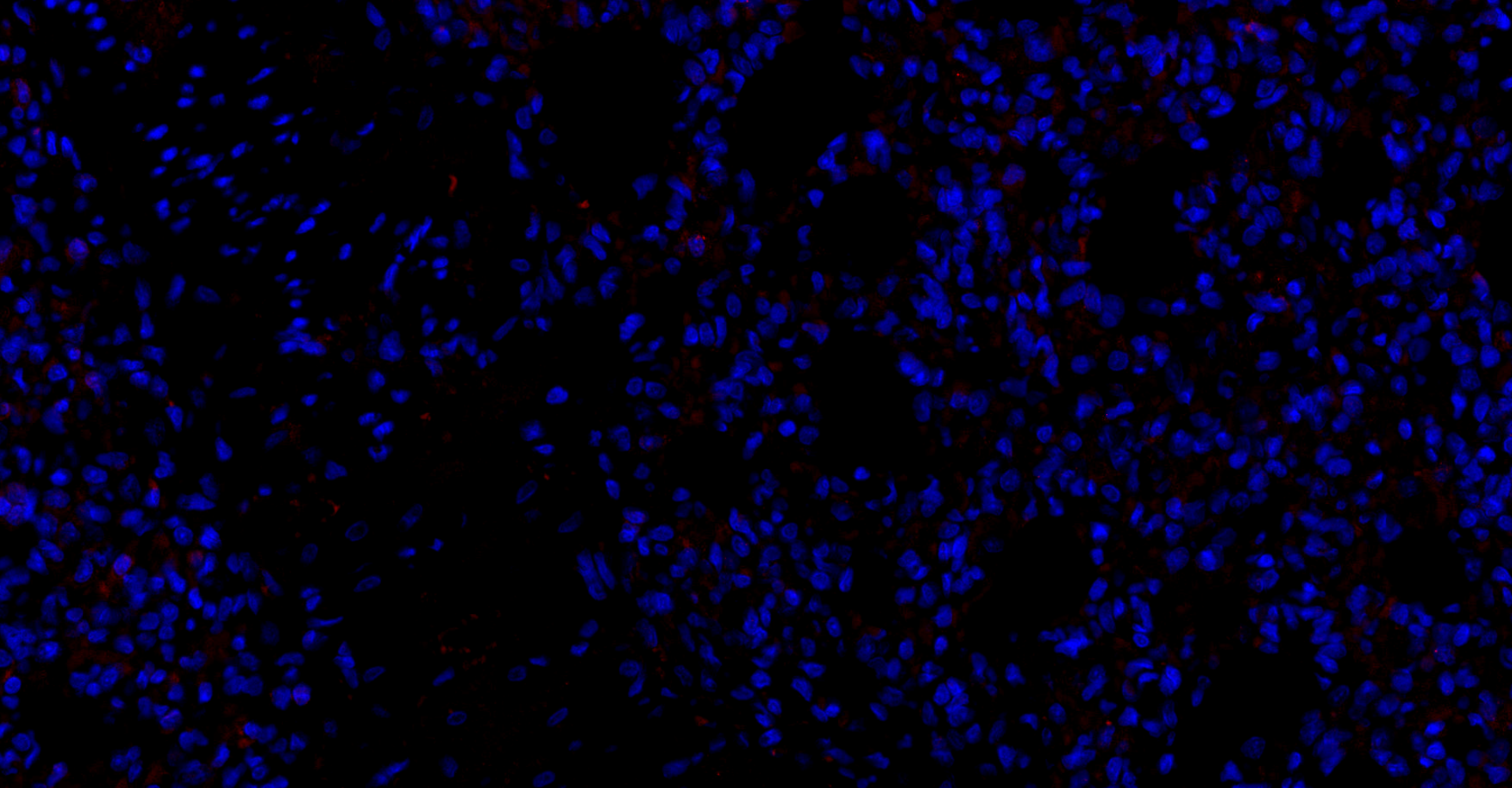

Supplement: Supplementary file 1 [file nutrients-17-02242-s001.zip › Figure S2 Original images/figure2-G-5 citH3 IF_40.0x.tif]

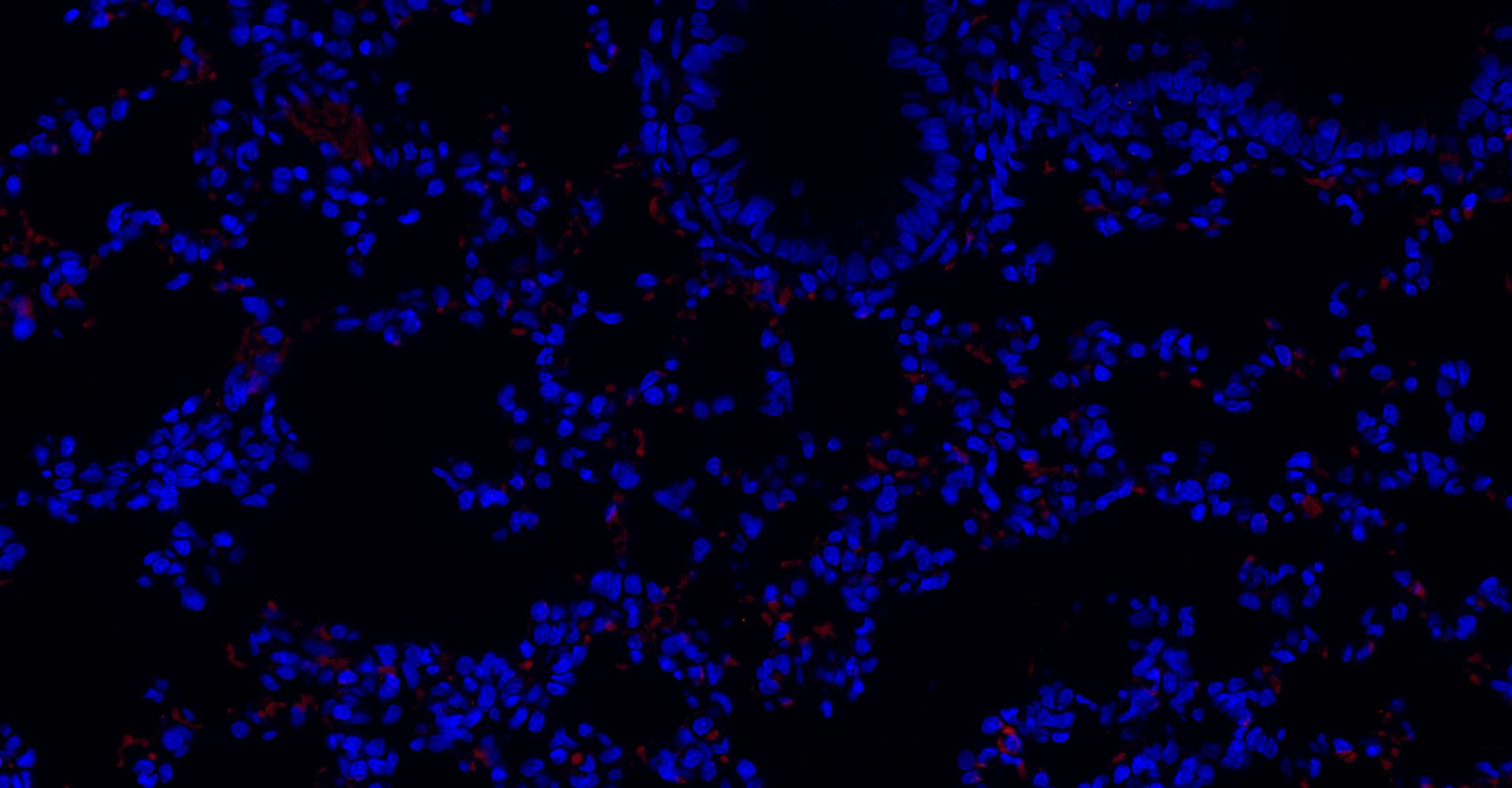

Supplement: Supplementary file 1 [file nutrients-17-02242-s001.zip › Figure S2 Original images/figure2-G-5 ly6g IF_40.0x.tif]

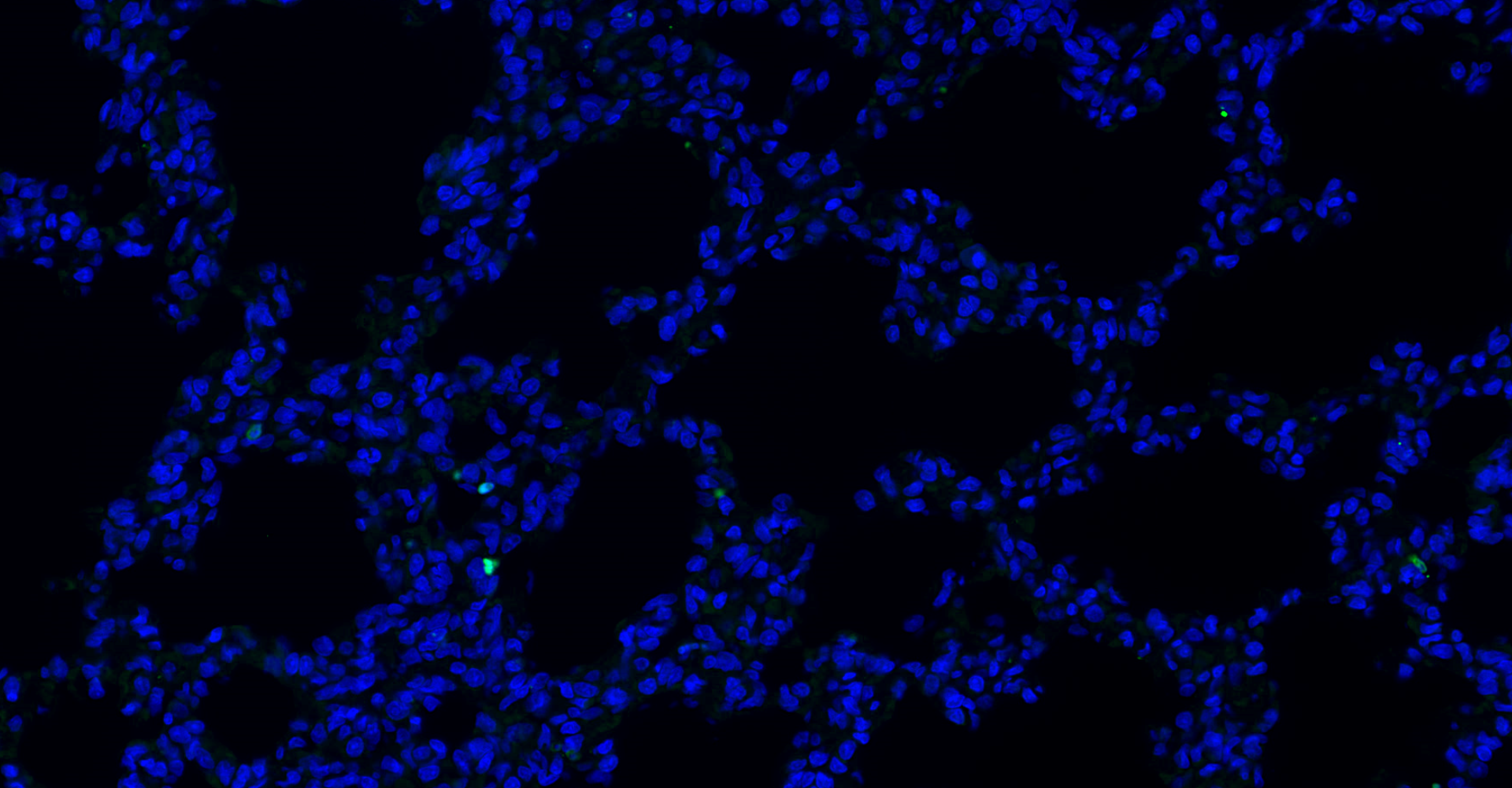

Supplement: Supplementary file 1 [file nutrients-17-02242-s001.zip › Figure S2 Original images/figure2-G-5 tunel IF_40.0x.tif]

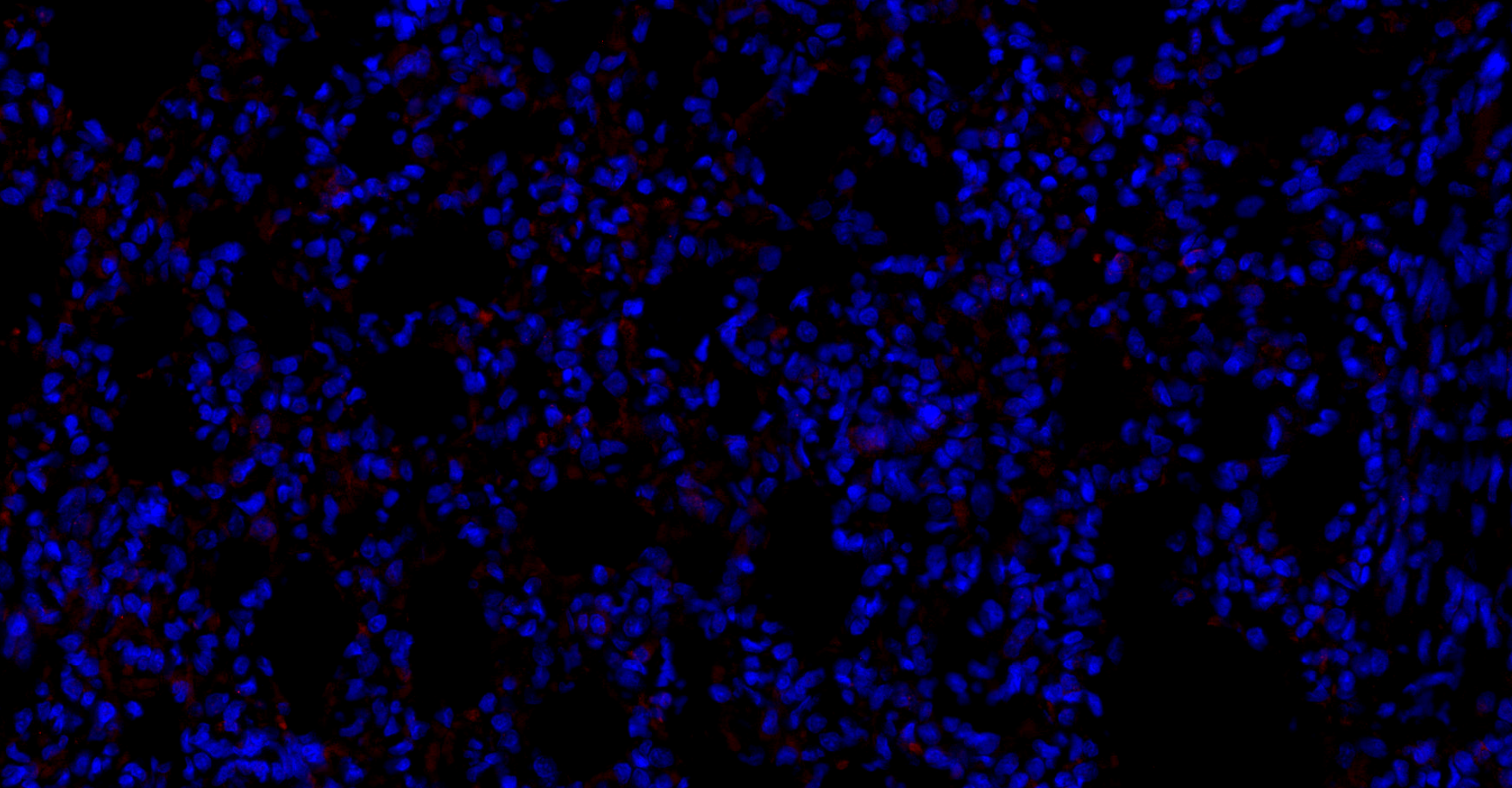

Supplement: Supplementary file 1 [file nutrients-17-02242-s001.zip › Figure S2 Original images/figure2-G-6 citH3 IF_40.0x.tif]

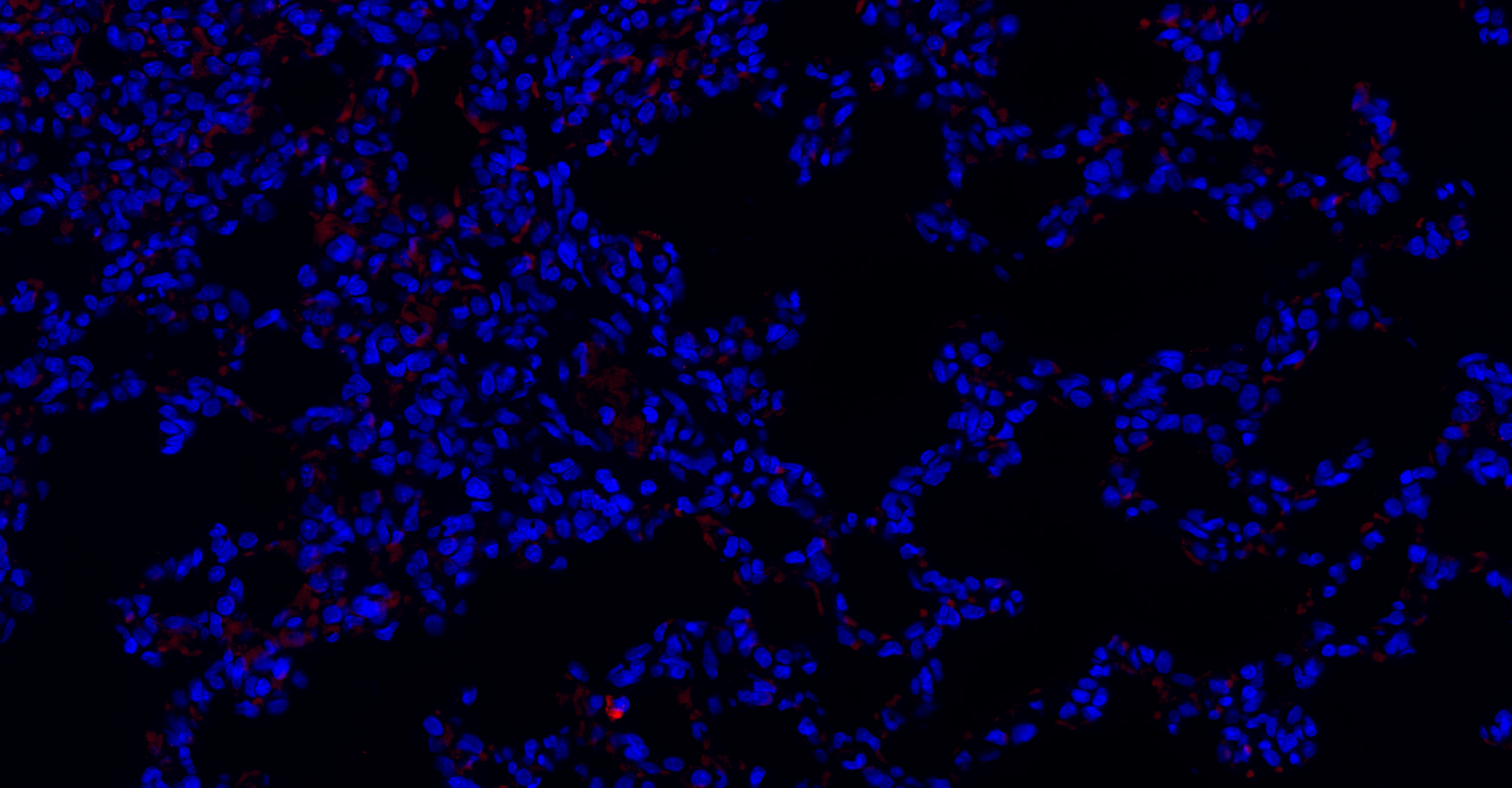

Supplement: Supplementary file 1 [file nutrients-17-02242-s001.zip › Figure S2 Original images/figure2-G-6 ly6g IF_40.0x.tif]

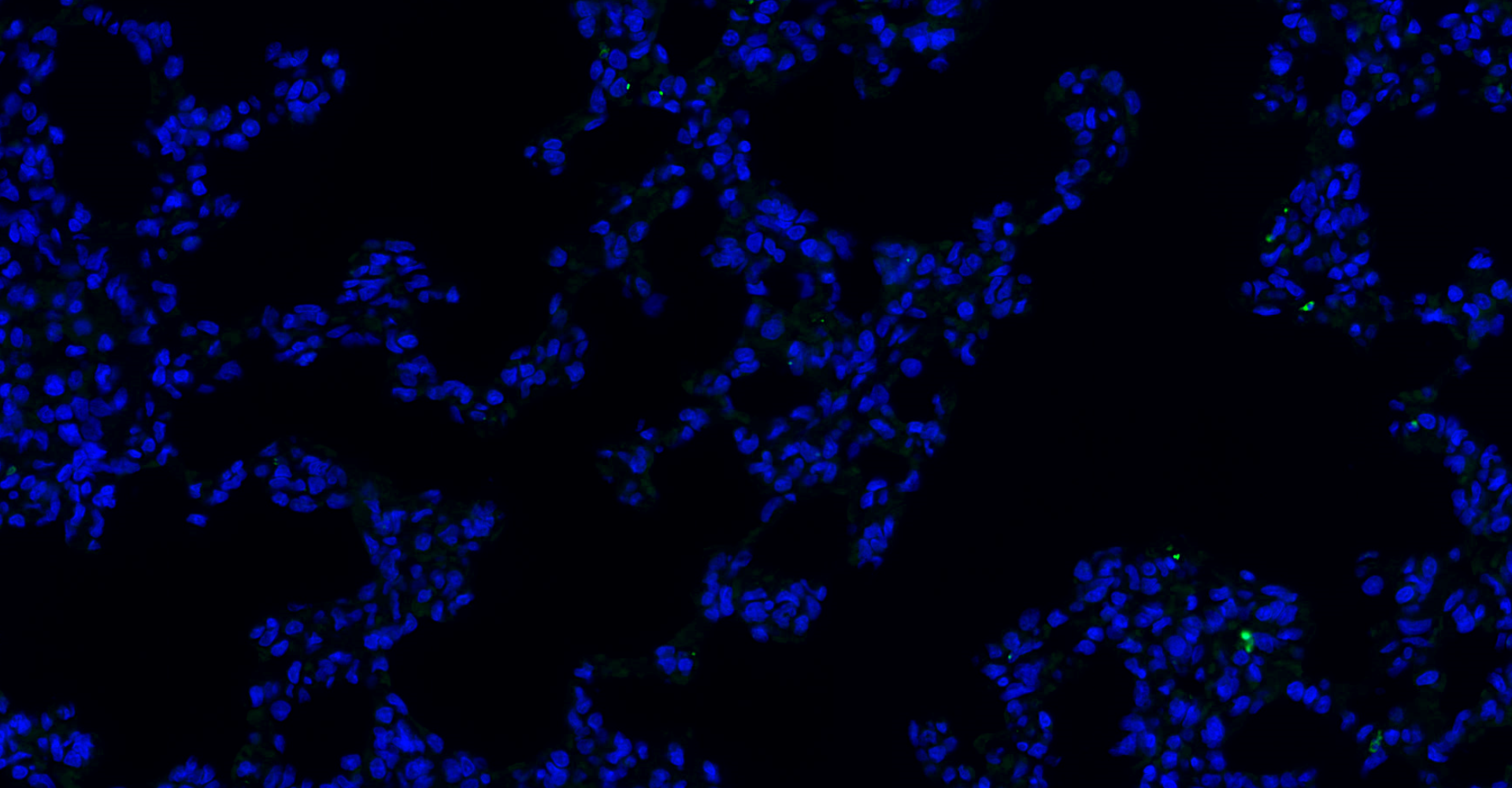

Supplement: Supplementary file 1 [file nutrients-17-02242-s001.zip › Figure S2 Original images/figure2-G-6 tunel IF_40.0x.tif]

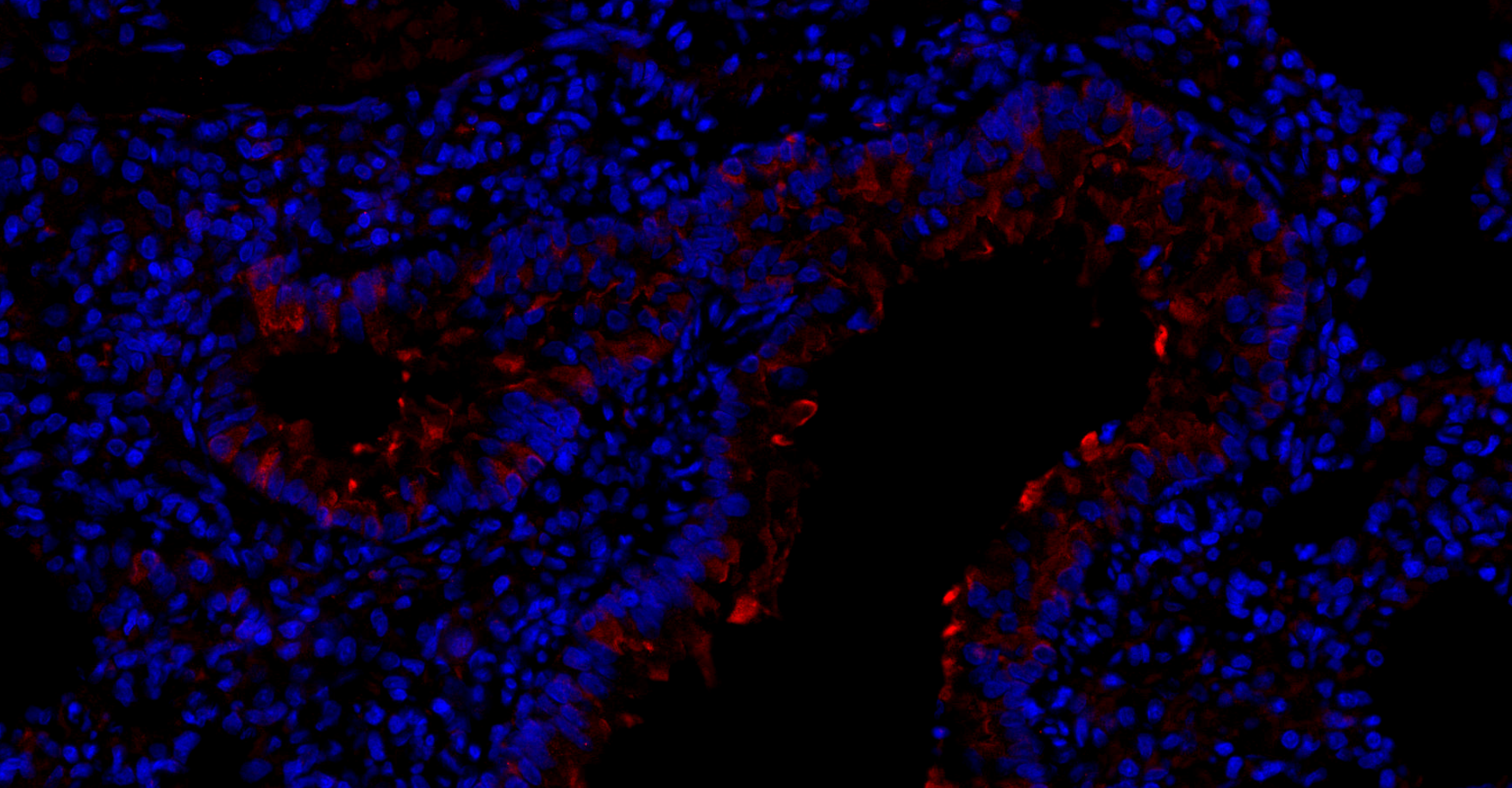

Supplement: Supplementary file 1 [file nutrients-17-02242-s001.zip › Figure S2 Original images/figure2-GP-1 cith3 IF_40.0x.tif]

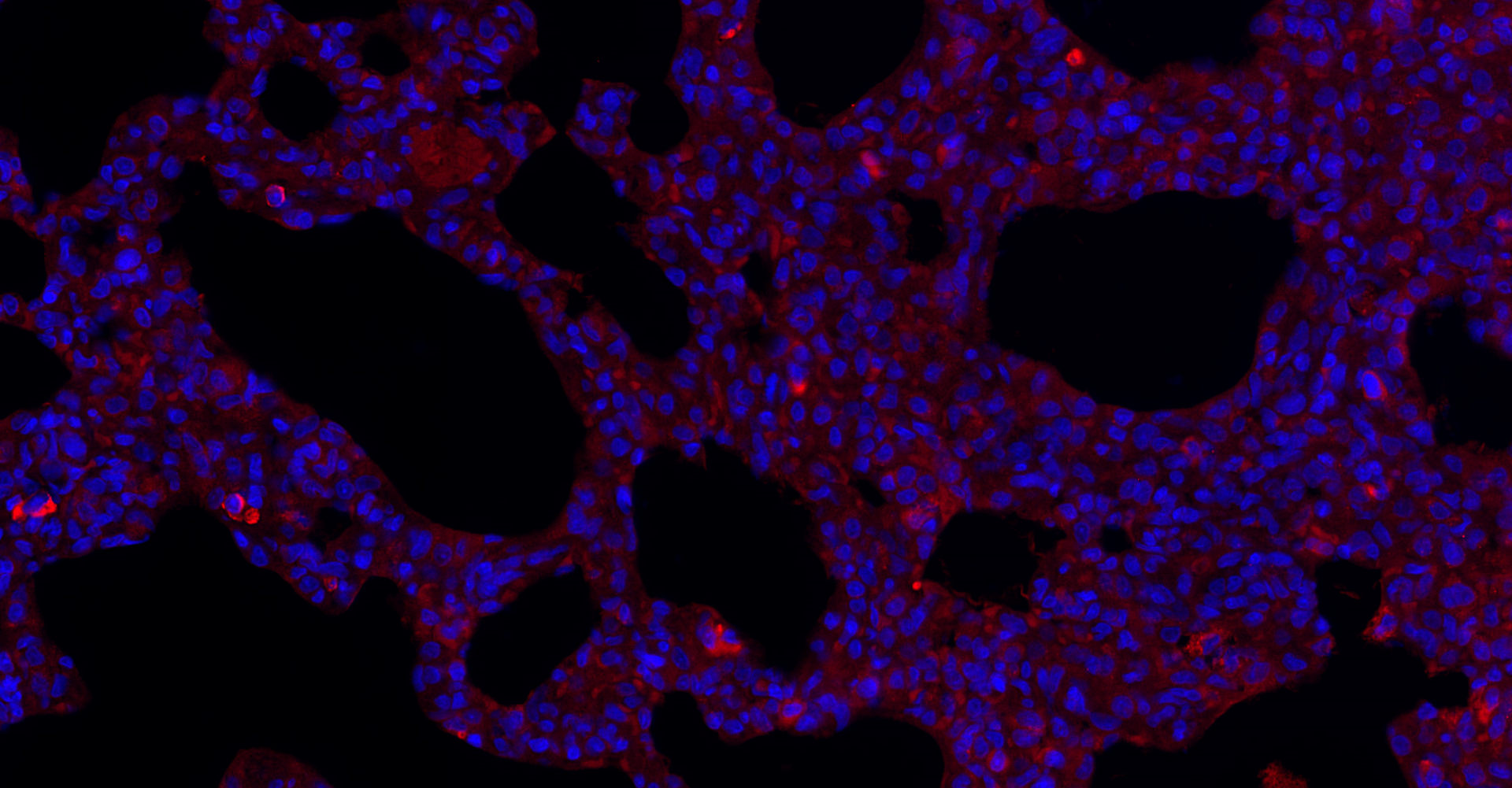

Supplement: Supplementary file 1 [file nutrients-17-02242-s001.zip › Figure S2 Original images/figure2-GP-1 ly6g IF_40.0x.tif]

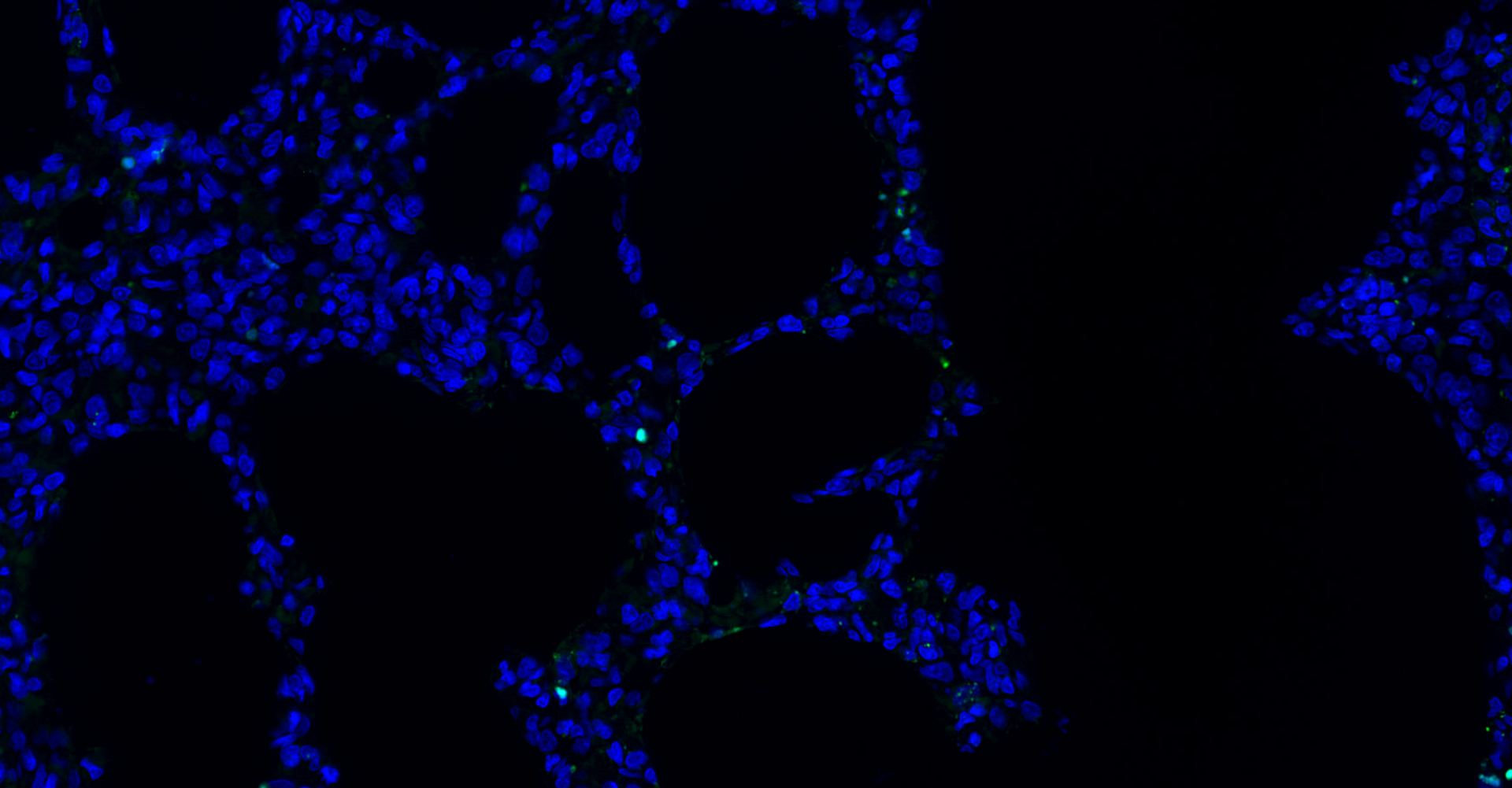

Supplement: Supplementary file 1 [file nutrients-17-02242-s001.zip › Figure S2 Original images/figure2-GP-1 tunnel IF_40.0x.tif]

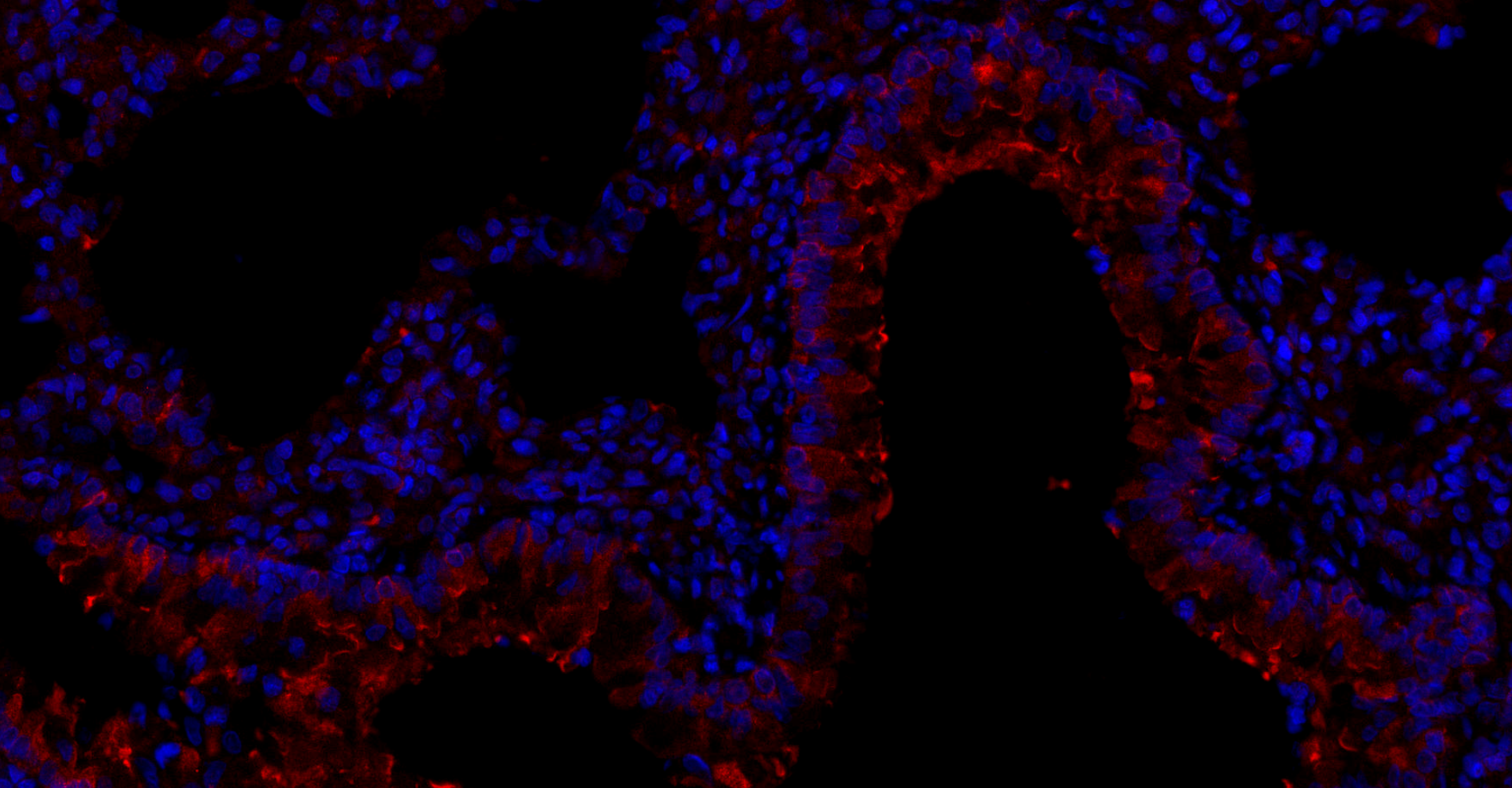

Supplement: Supplementary file 1 [file nutrients-17-02242-s001.zip › Figure S2 Original images/figure2-GP-2 cith3 IF_40.0x.tif]

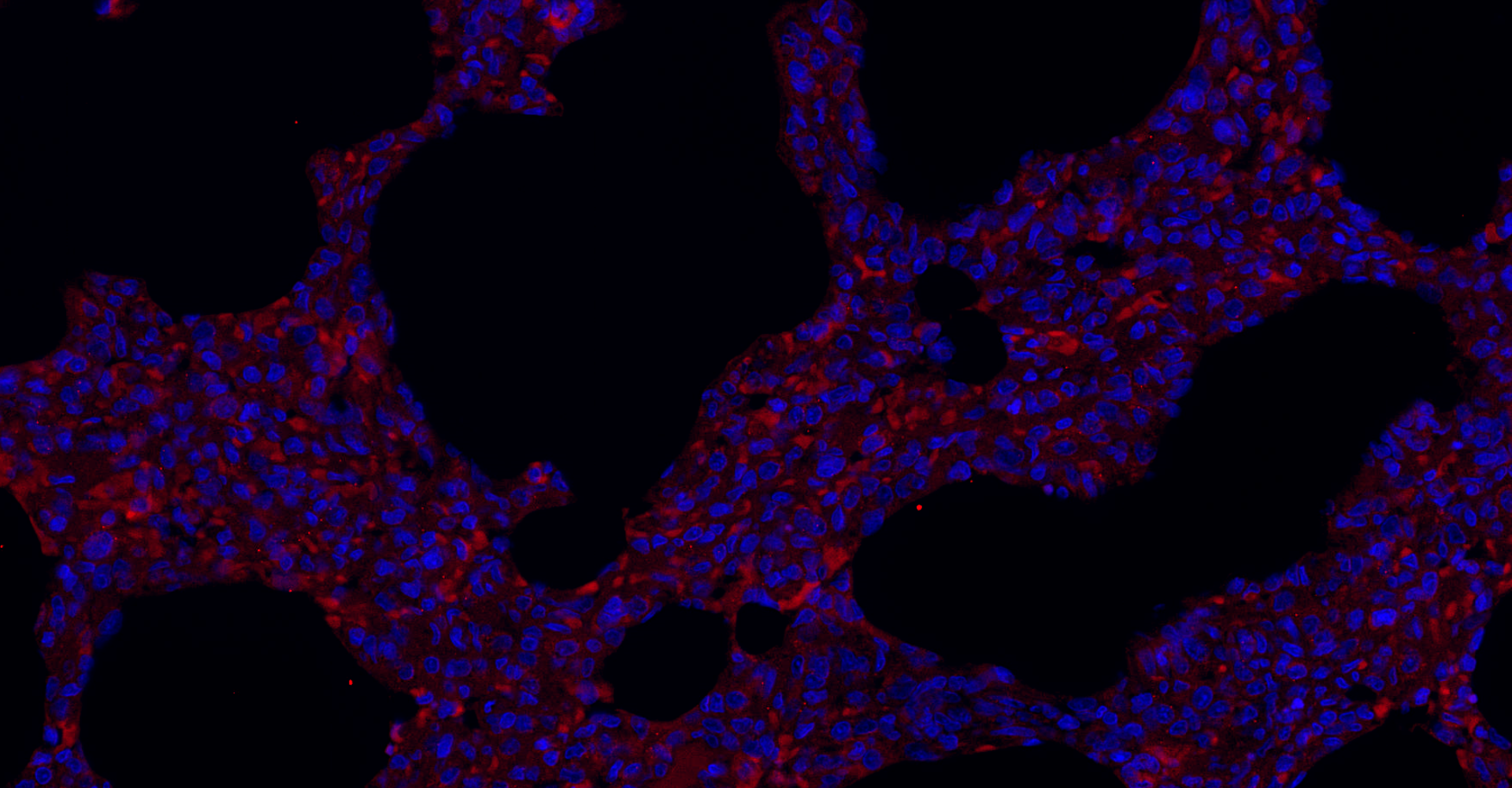

Supplement: Supplementary file 1 [file nutrients-17-02242-s001.zip › Figure S2 Original images/figure2-GP-2 ly6g IF_40.0x.tif]

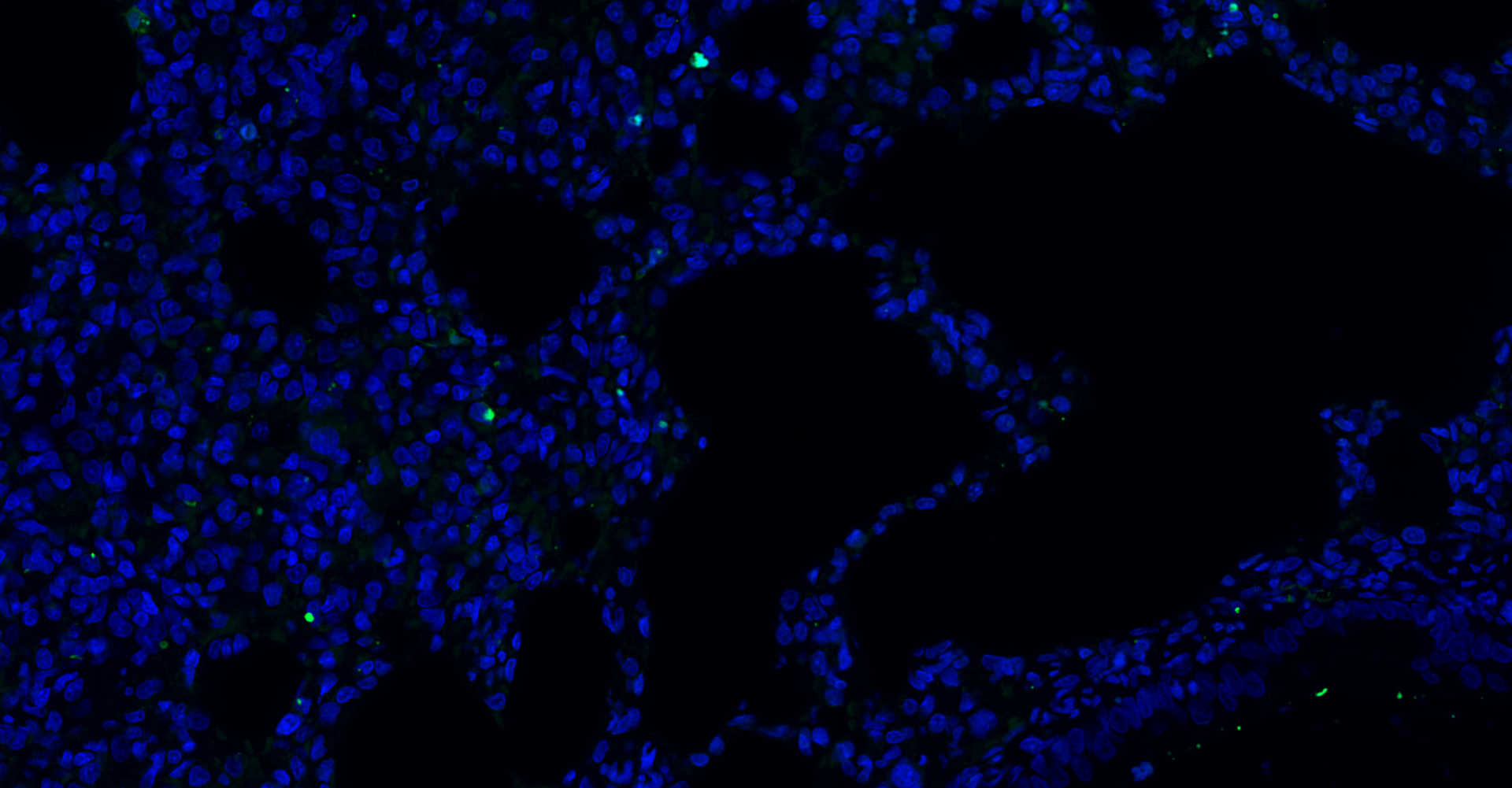

Supplement: Supplementary file 1 [file nutrients-17-02242-s001.zip › Figure S2 Original images/figure2-GP-2 tunnel IF_40.0x.tif]

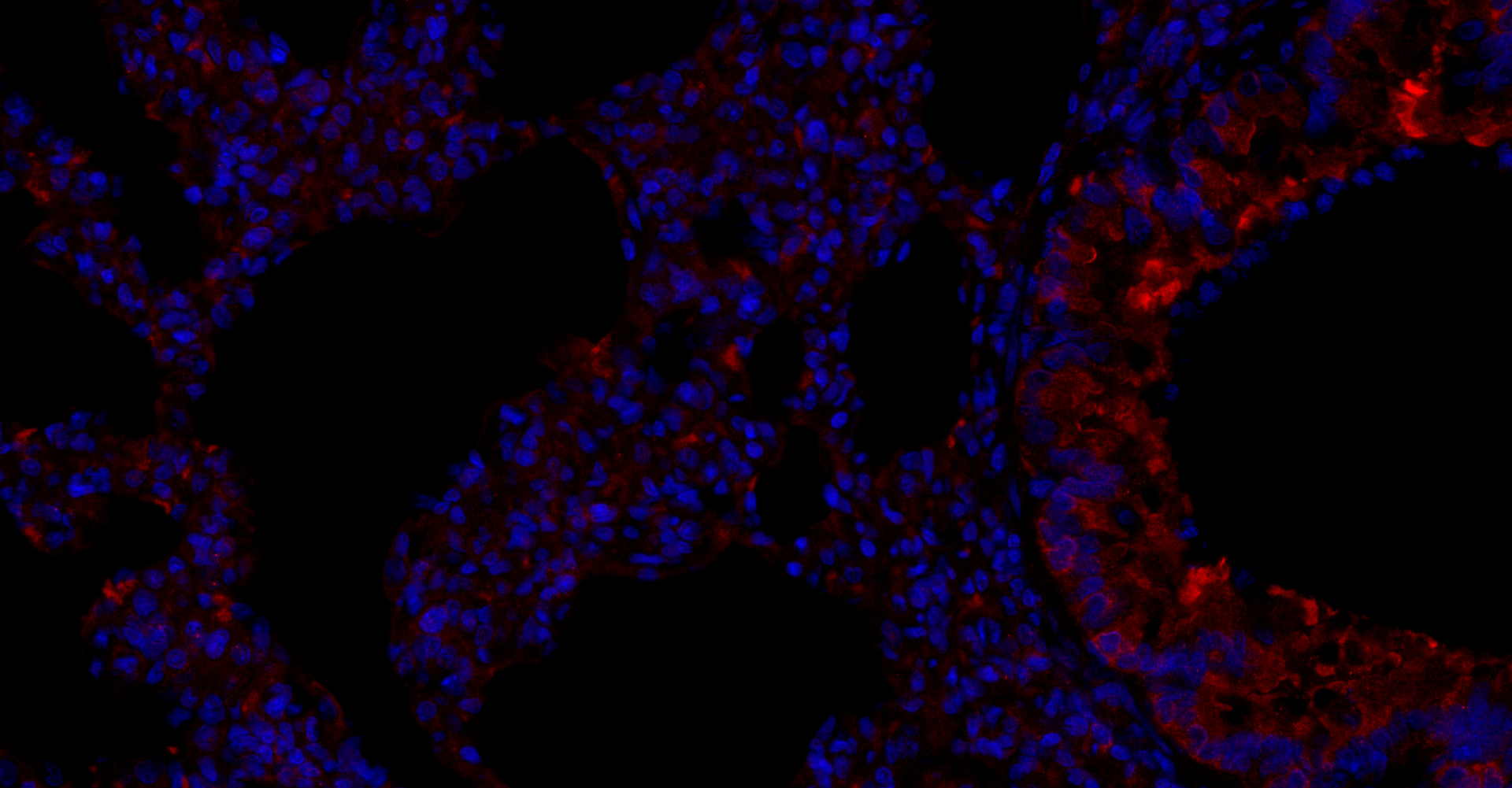

Supplement: Supplementary file 1 [file nutrients-17-02242-s001.zip › Figure S2 Original images/figure2-GP-3 cith3 IF_40.0x.tif]

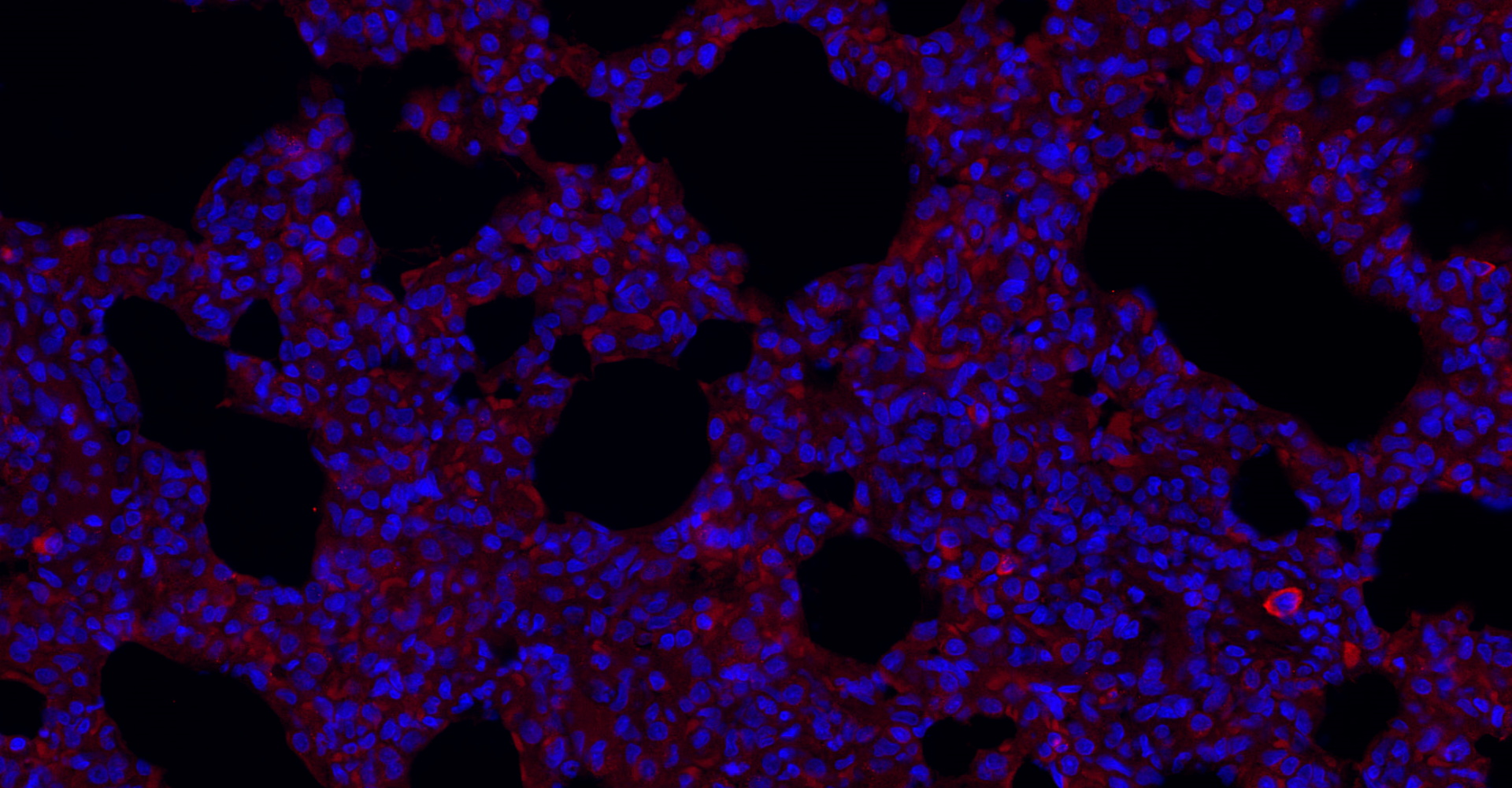

Supplement: Supplementary file 1 [file nutrients-17-02242-s001.zip › Figure S2 Original images/figure2-GP-3 ly6g IF_40.0x.tif]

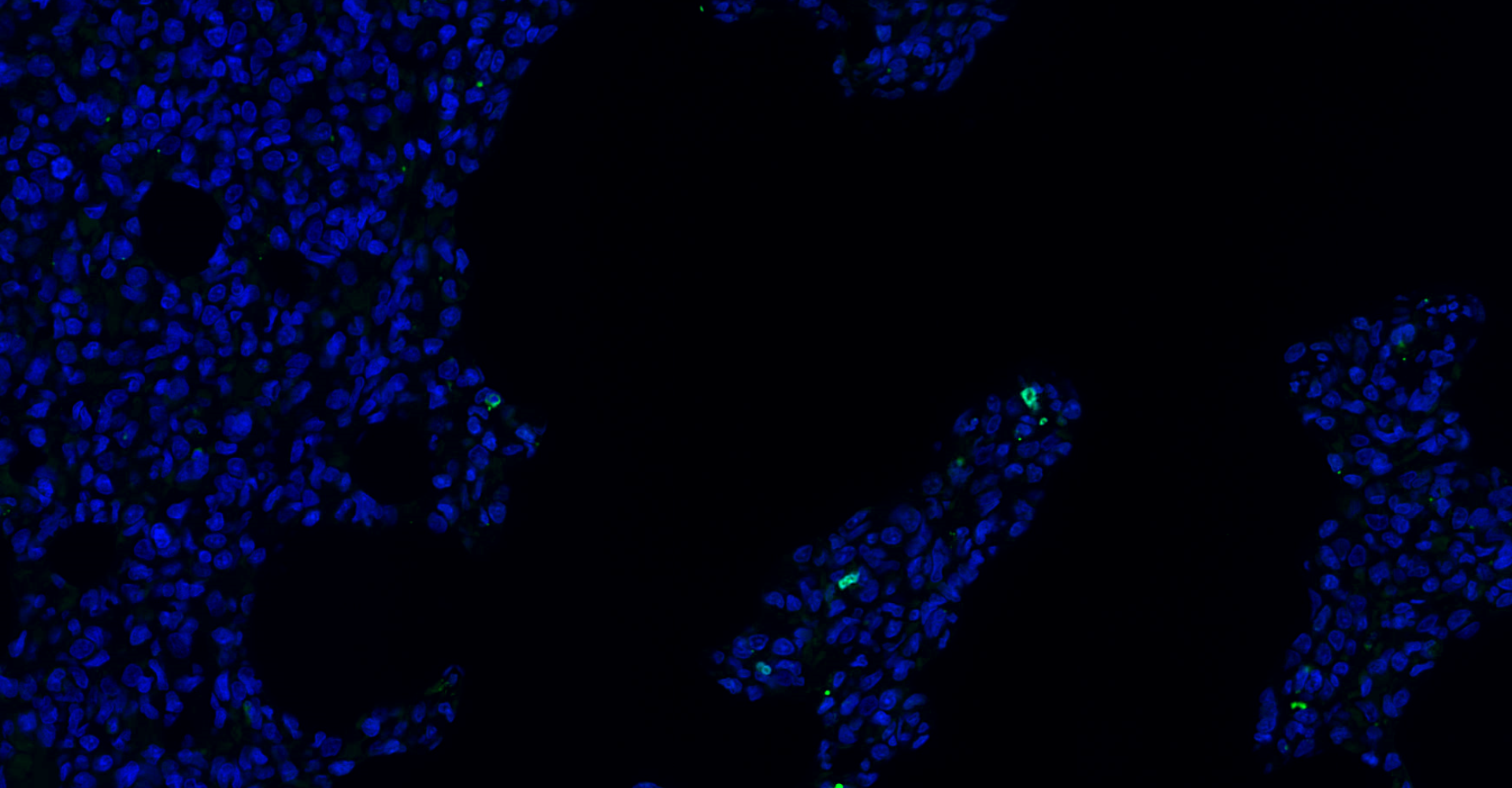

Supplement: Supplementary file 1 [file nutrients-17-02242-s001.zip › Figure S2 Original images/figure2-GP-3 tunnel IF_40.0x.tif]

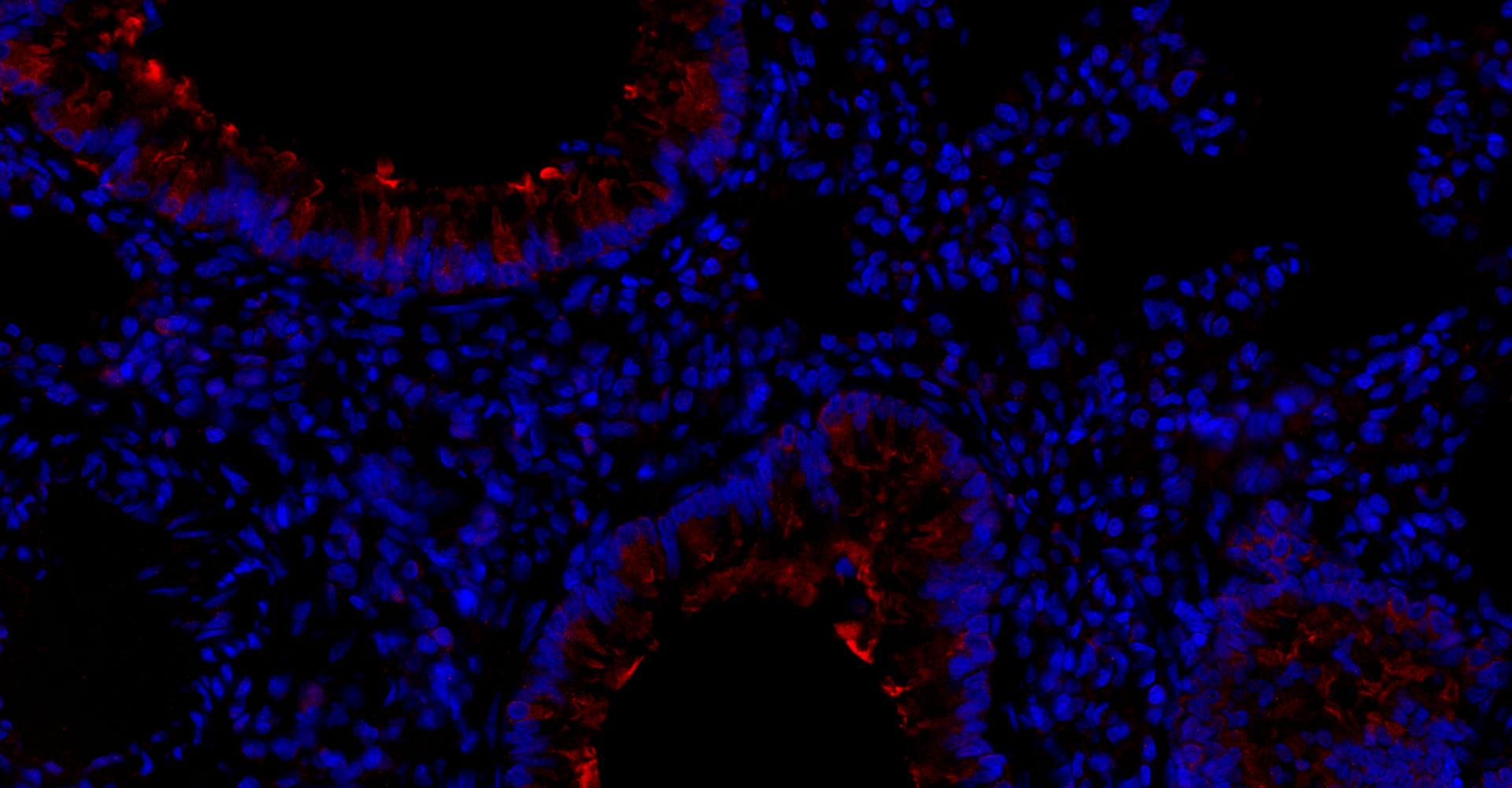

Supplement: Supplementary file 1 [file nutrients-17-02242-s001.zip › Figure S2 Original images/figure2-GP-4 cith3 IF_40.0x.tif]

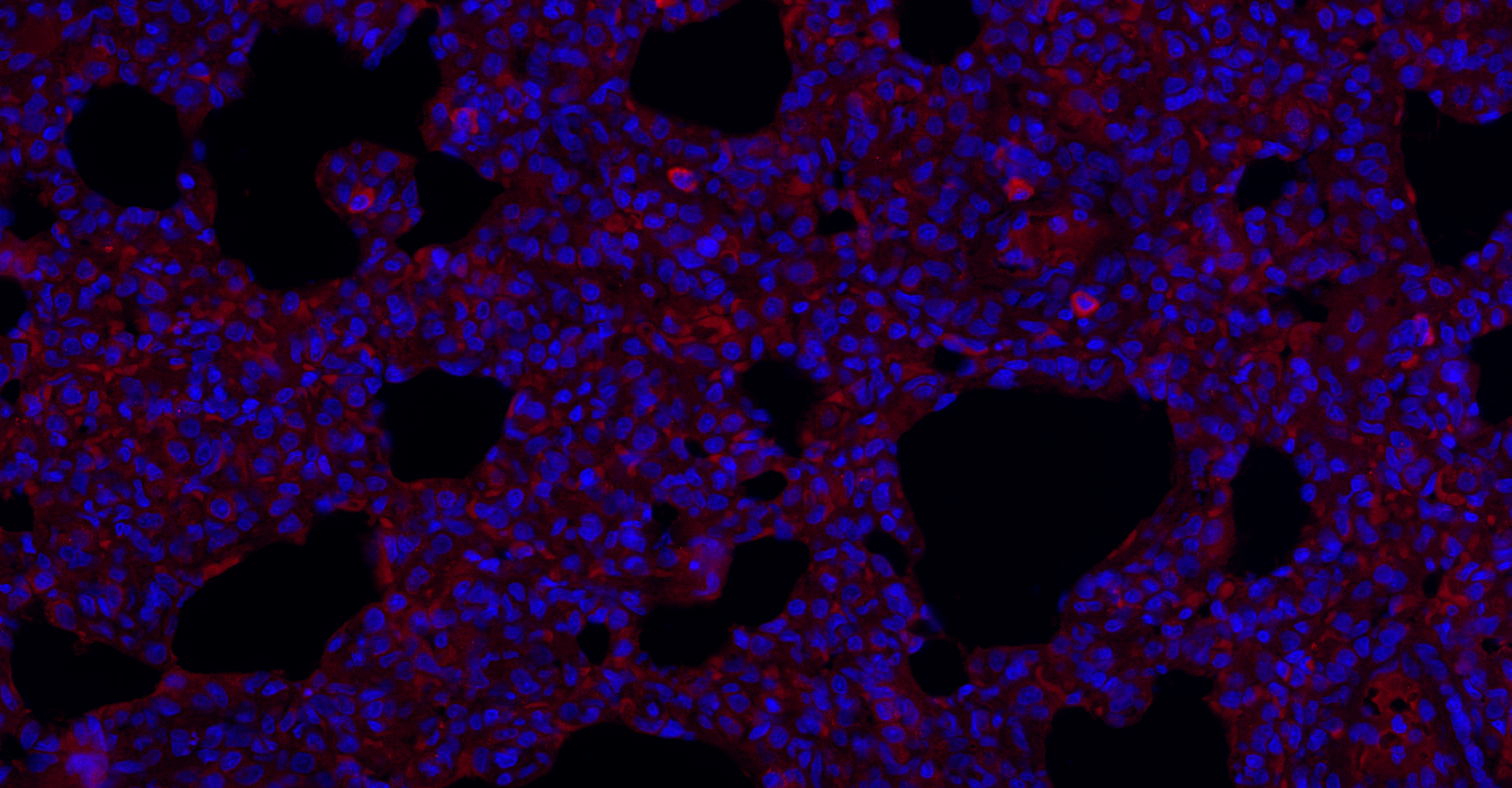

Supplement: Supplementary file 1 [file nutrients-17-02242-s001.zip › Figure S2 Original images/figure2-GP-4 ly6g IF_40.0x.tif]

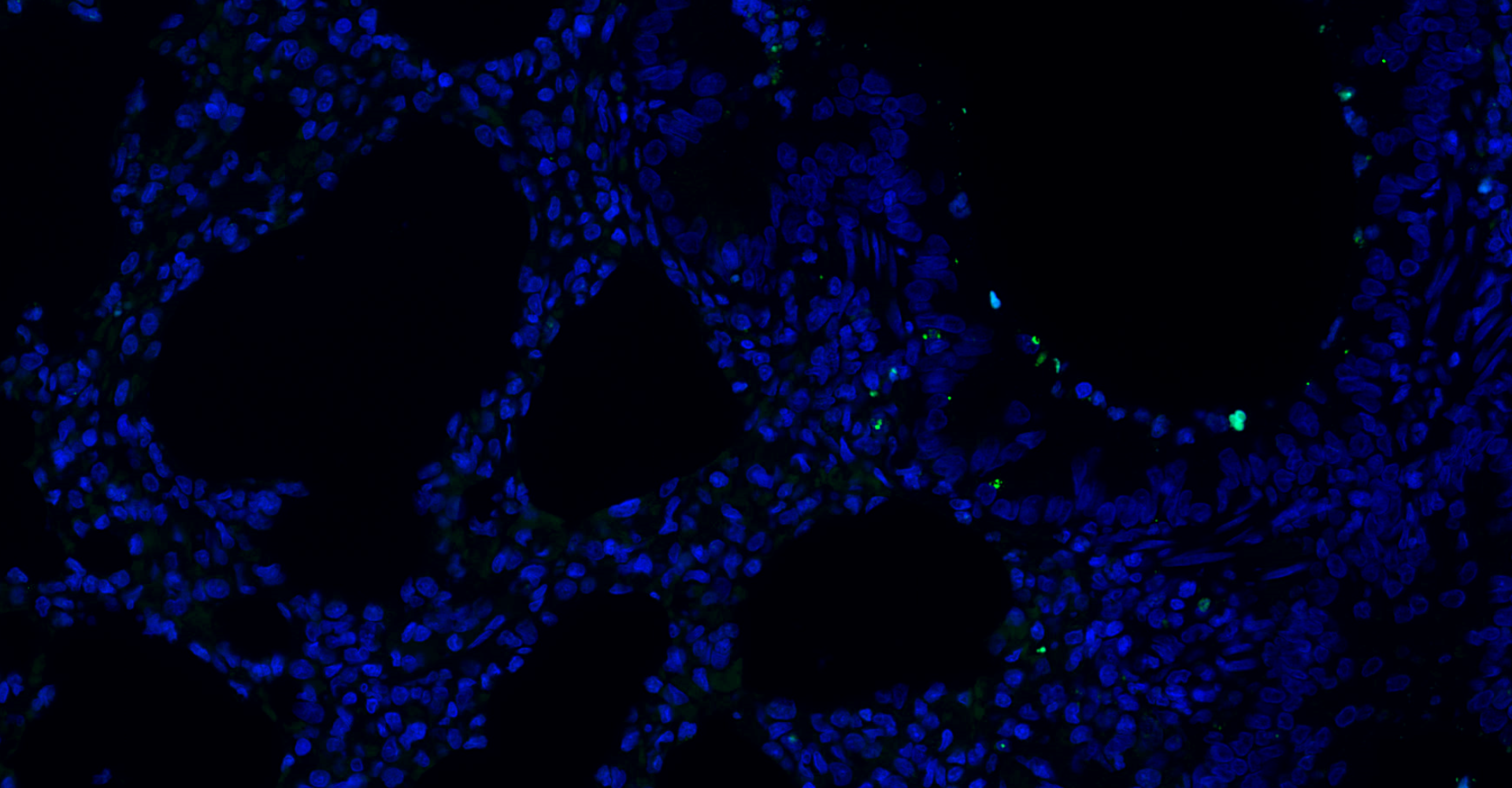

Supplement: Supplementary file 1 [file nutrients-17-02242-s001.zip › Figure S2 Original images/figure2-GP-4 tunnel IF_40.0x.tif]

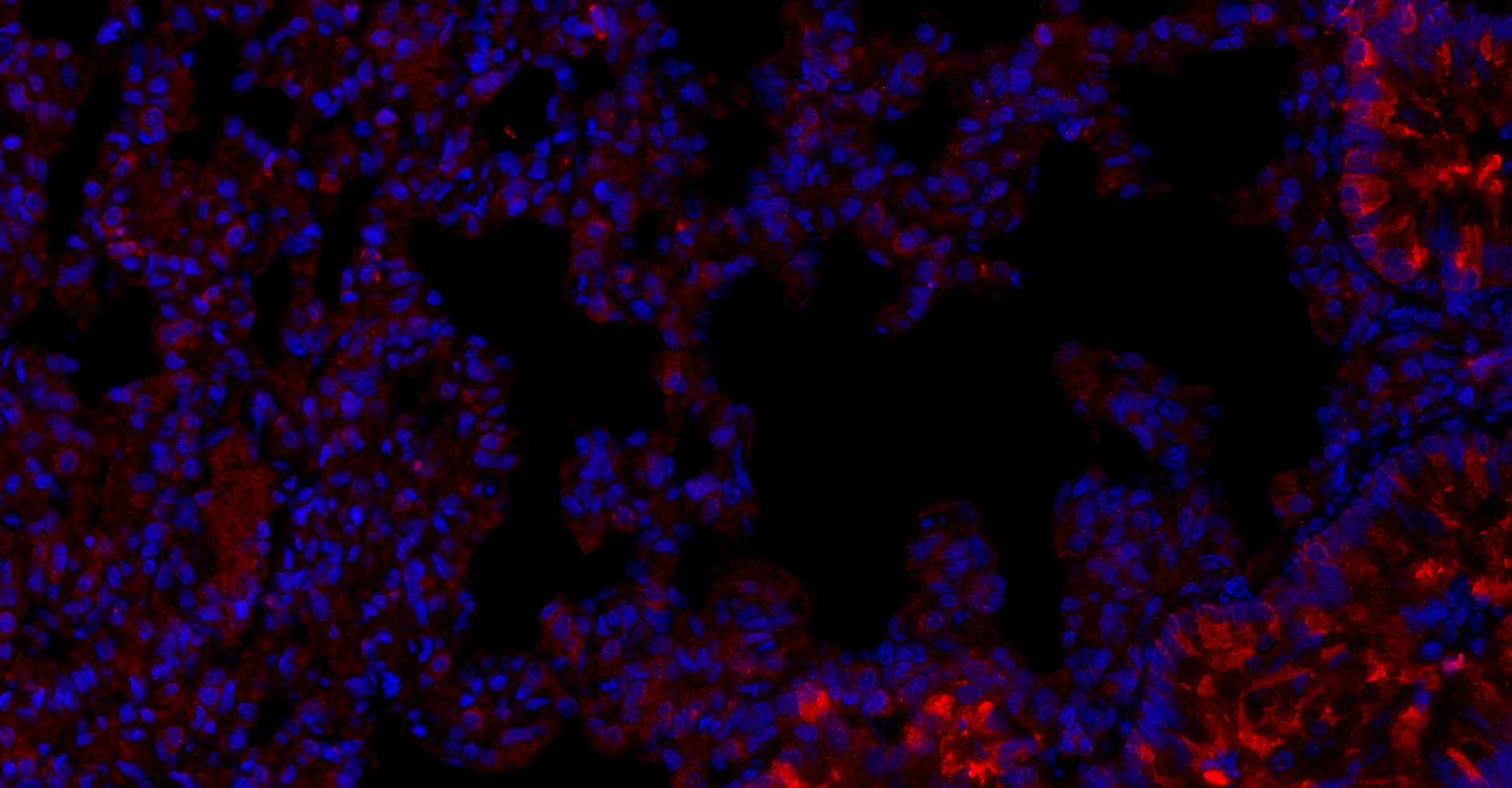

Supplement: Supplementary file 1 [file nutrients-17-02242-s001.zip › Figure S2 Original images/figure2-GP-5 cith3 IF_40.0x.tif]

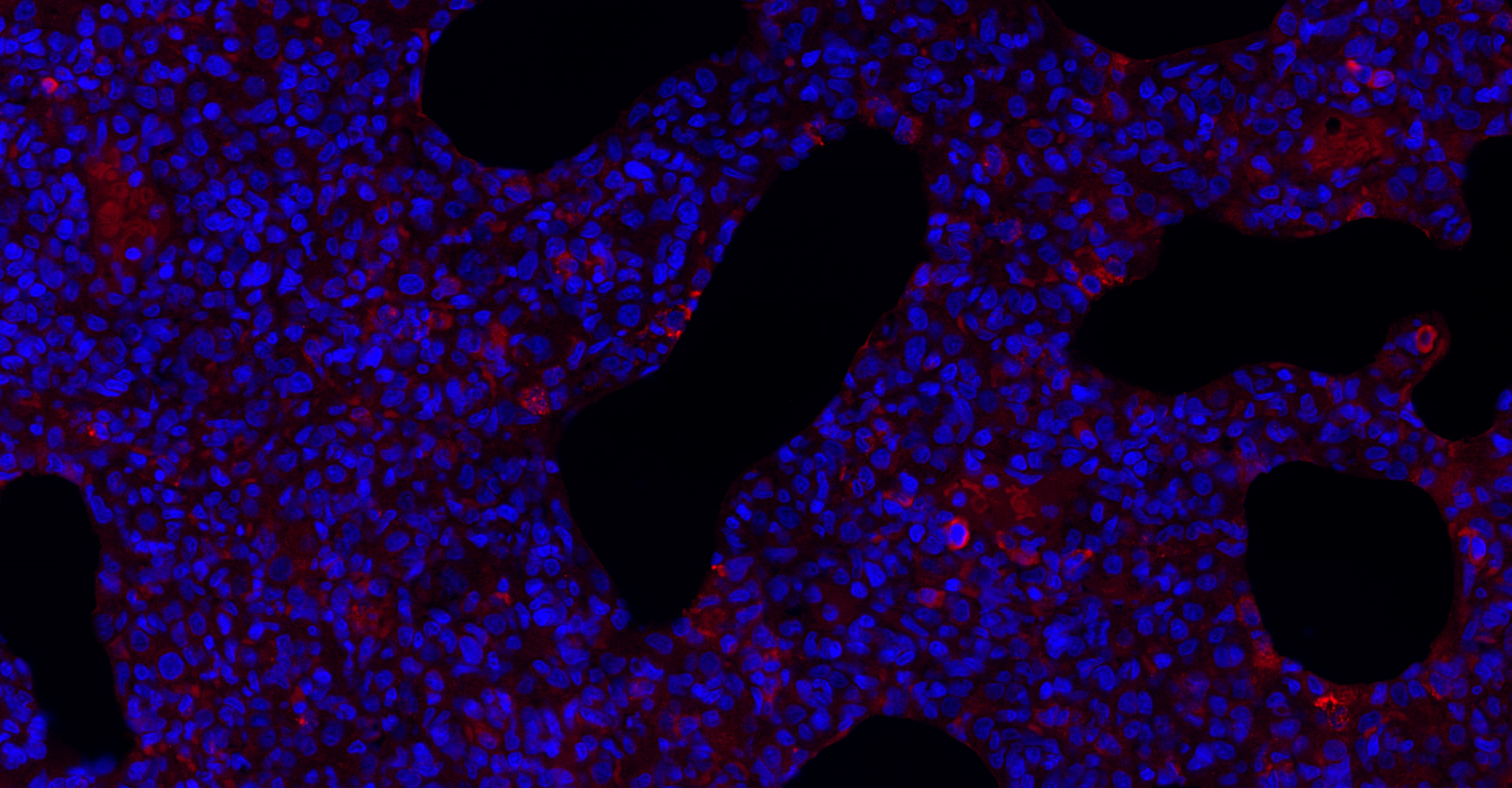

Supplement: Supplementary file 1 [file nutrients-17-02242-s001.zip › Figure S2 Original images/figure2-GP-5 ly6g IF_40.0x.tif]

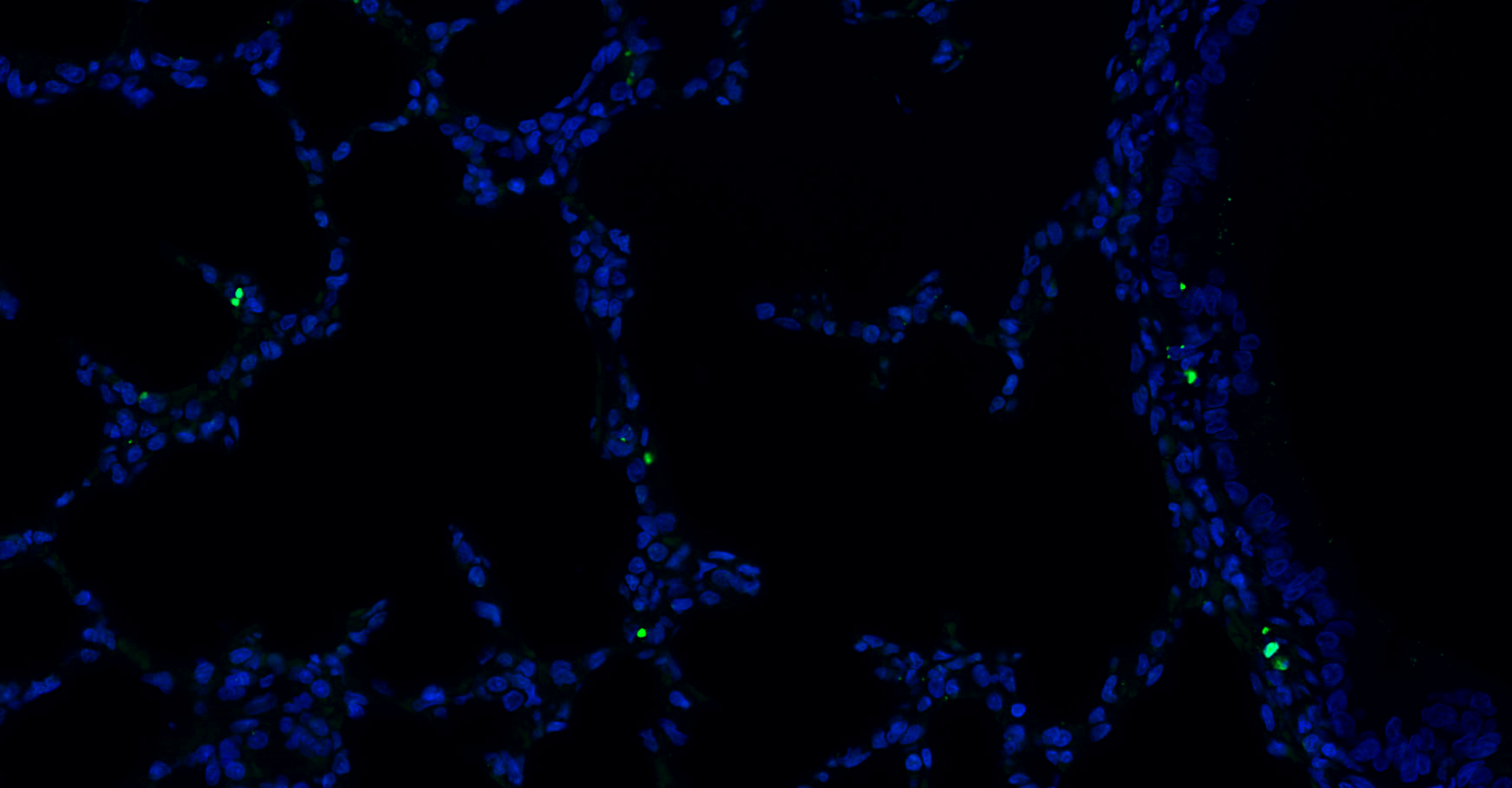

Supplement: Supplementary file 1 [file nutrients-17-02242-s001.zip › Figure S2 Original images/figure2-GP-5 tunnel IF_40.0x.tif]

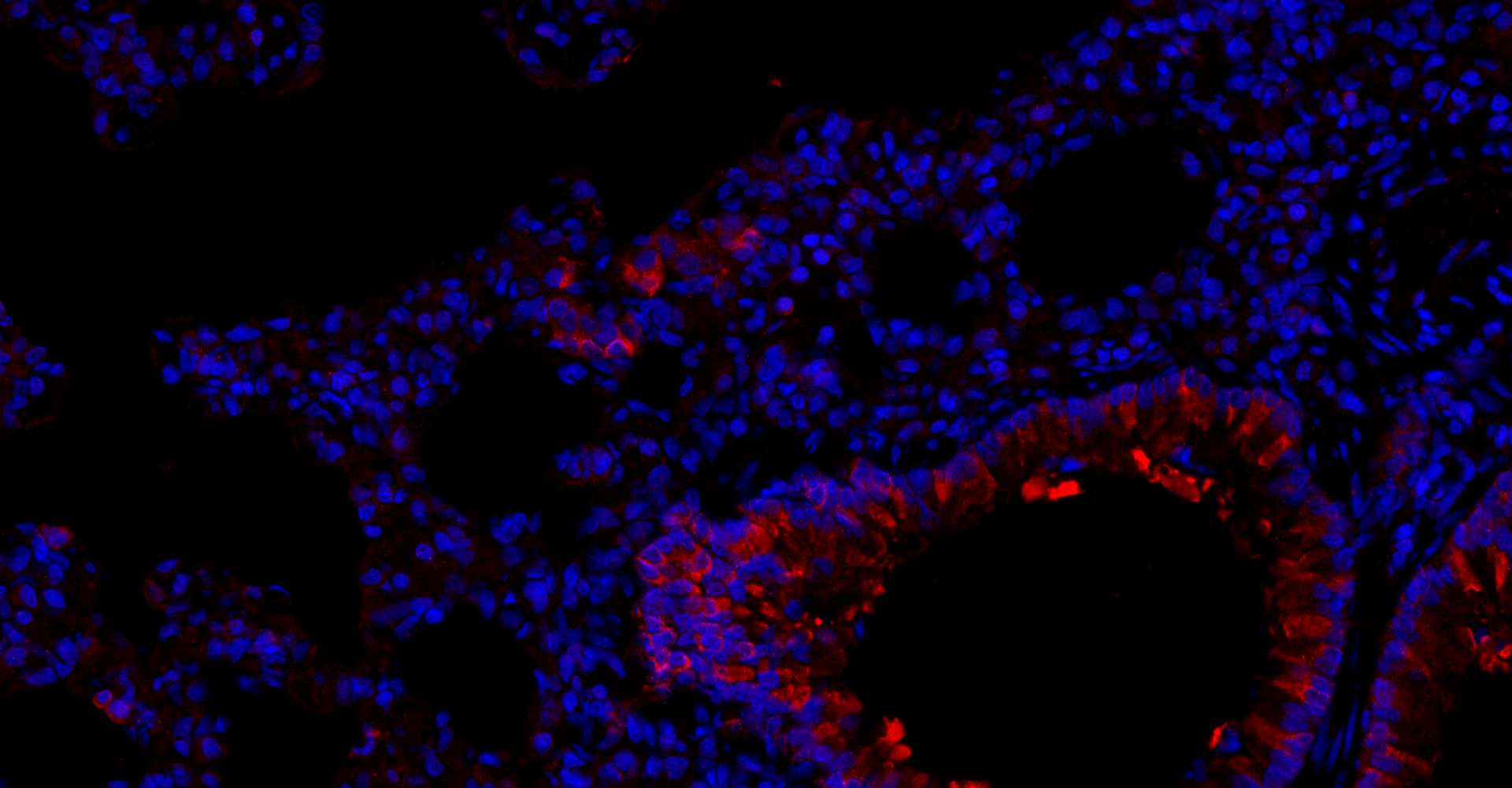

Supplement: Supplementary file 1 [file nutrients-17-02242-s001.zip › Figure S2 Original images/figure2-GP-6 cith3 IF_40.0x.tif]

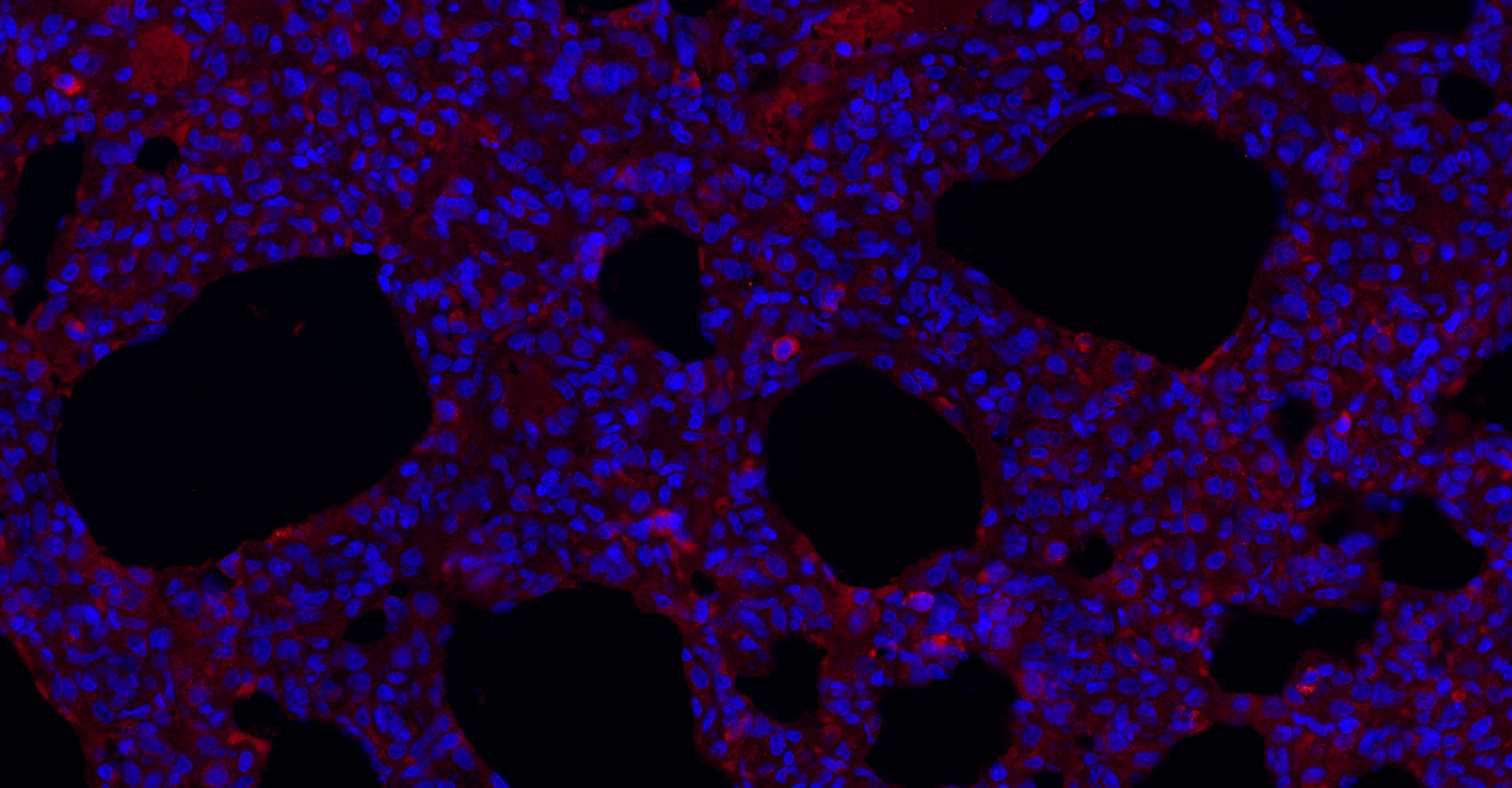

Supplement: Supplementary file 1 [file nutrients-17-02242-s001.zip › Figure S2 Original images/figure2-GP-6 ly6g IF_40.0x.tif]

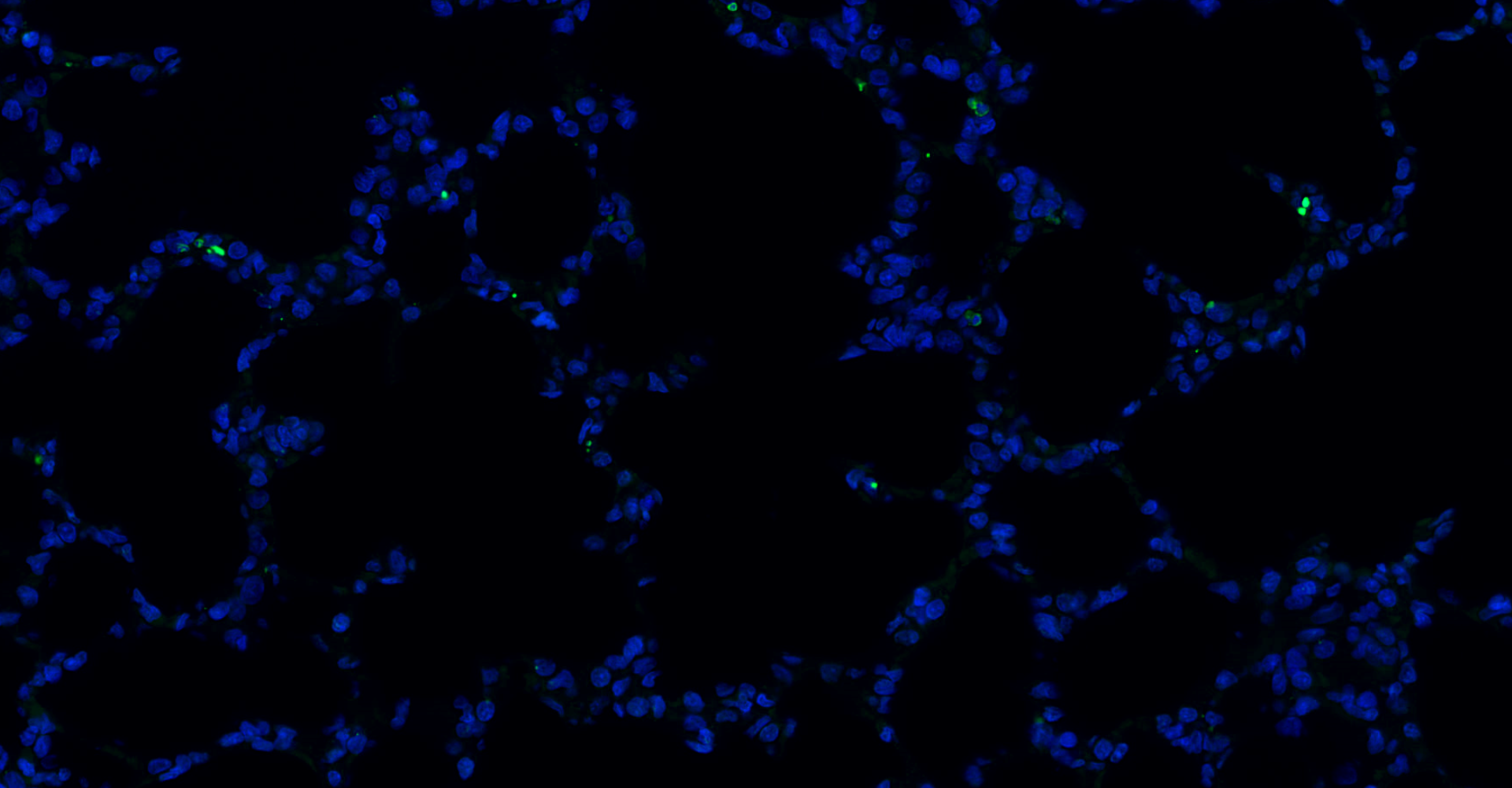

Supplement: Supplementary file 1 [file nutrients-17-02242-s001.zip › Figure S2 Original images/figure2-GP-6 tunnel IF_40.0x.tif]

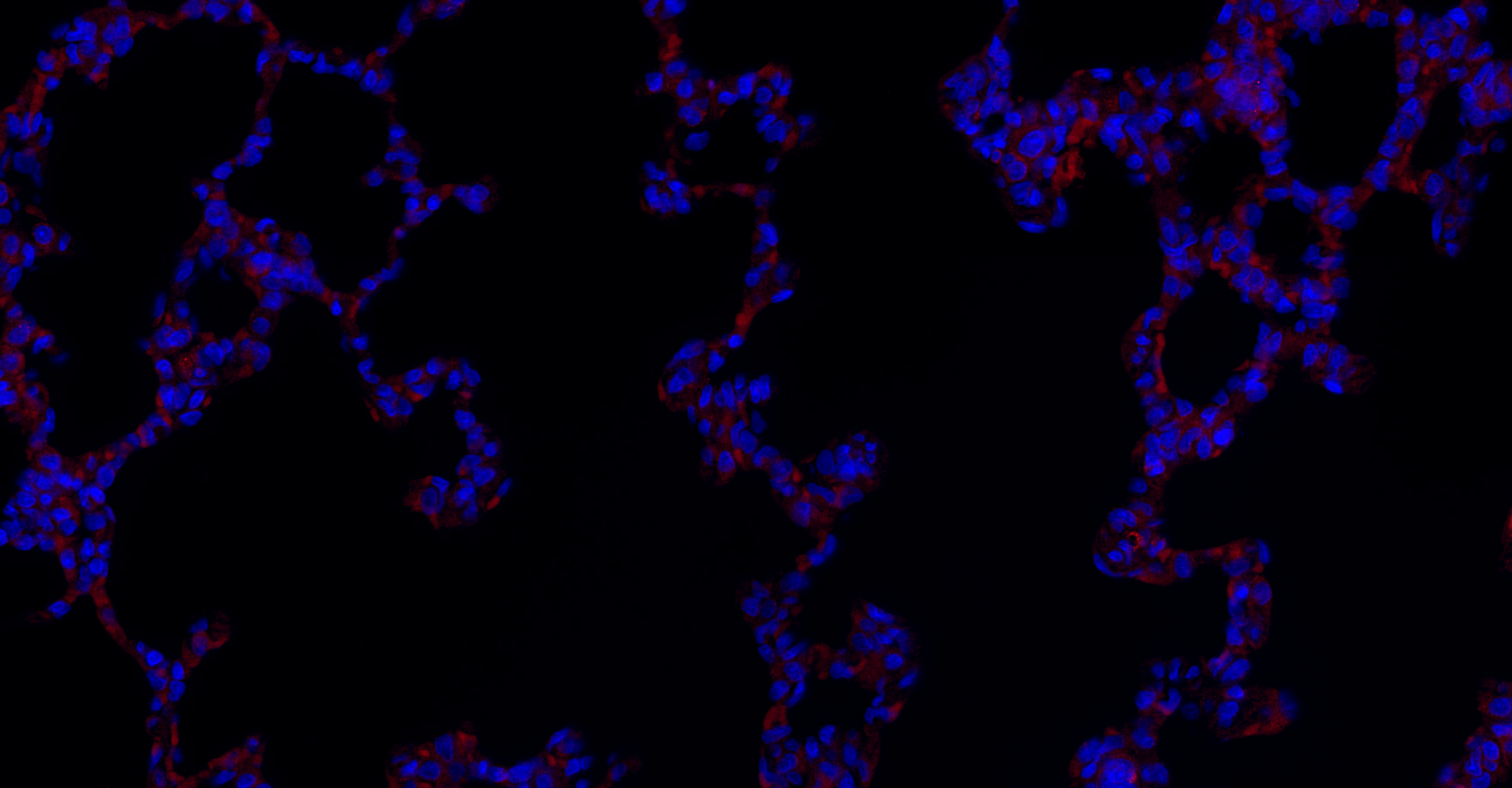

Supplement: Supplementary file 1 [file nutrients-17-02242-s001.zip › Figure S2 Original images/figure2-N-1 citH3 IF_40.0x.tif]

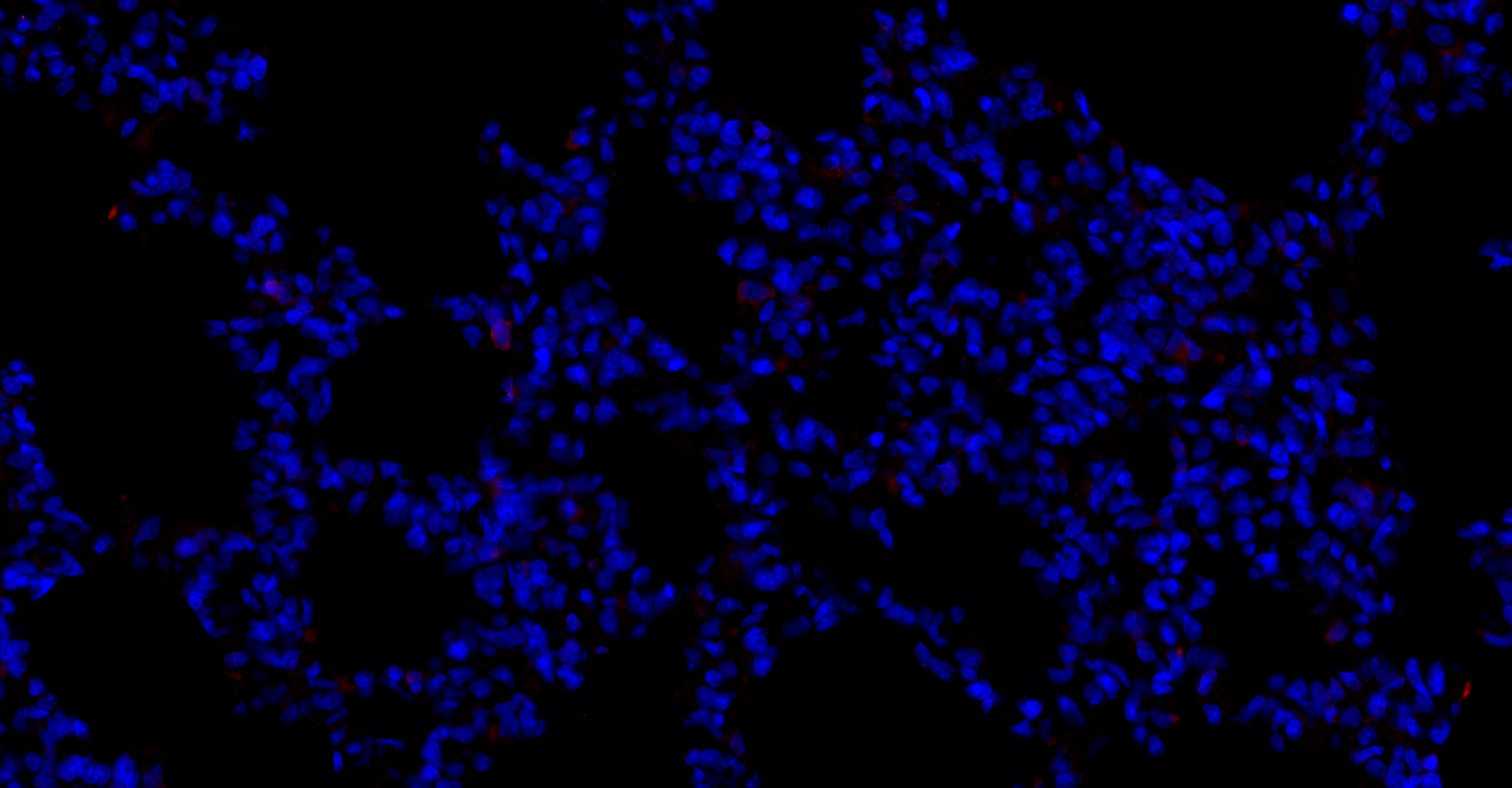

Supplement: Supplementary file 1 [file nutrients-17-02242-s001.zip › Figure S2 Original images/figure2-N-1 ly6g IF_40.0x.tif]

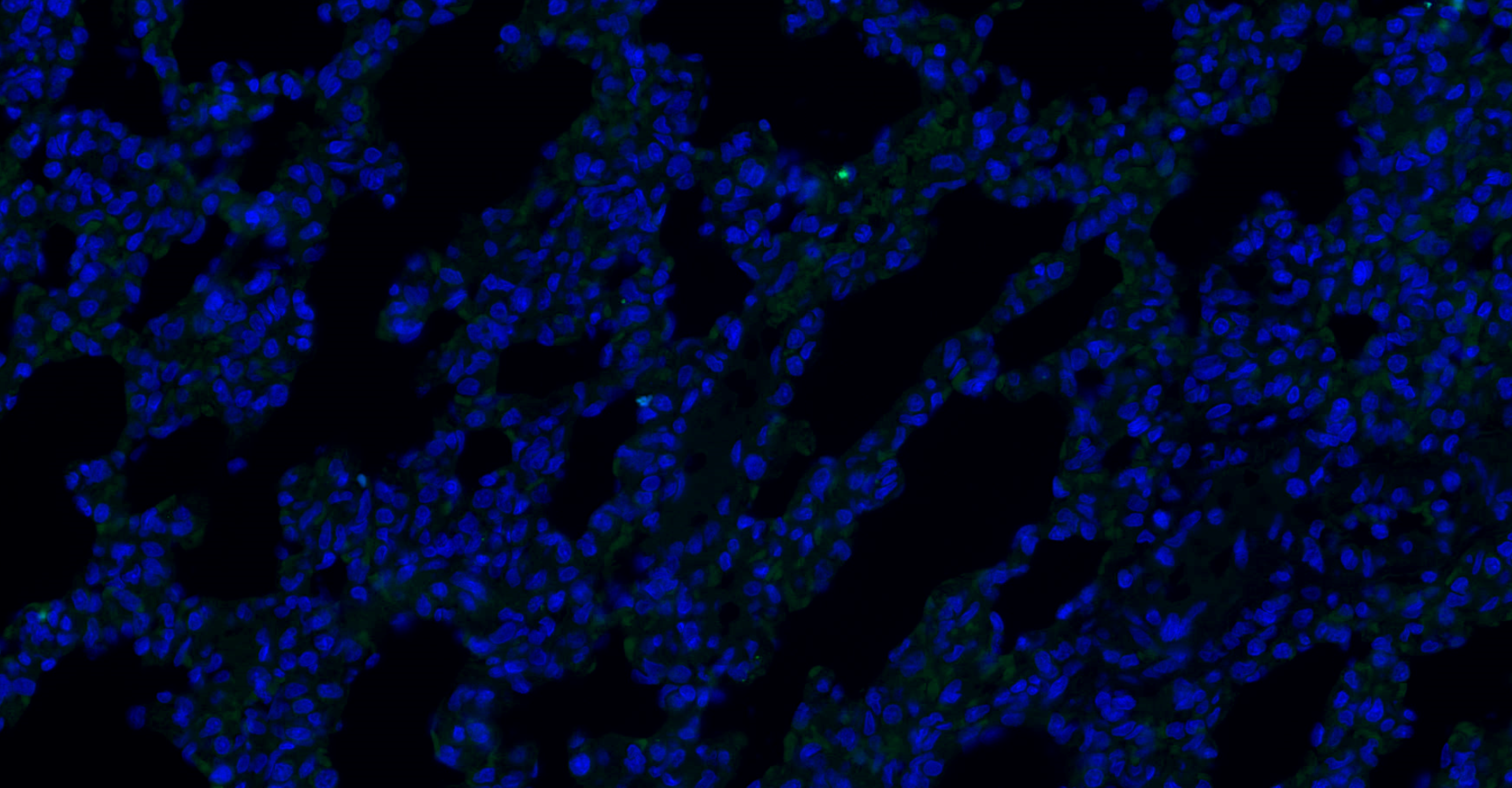

Supplement: Supplementary file 1 [file nutrients-17-02242-s001.zip › Figure S2 Original images/figure2-N-1 tunel IF_40.0x.tif]

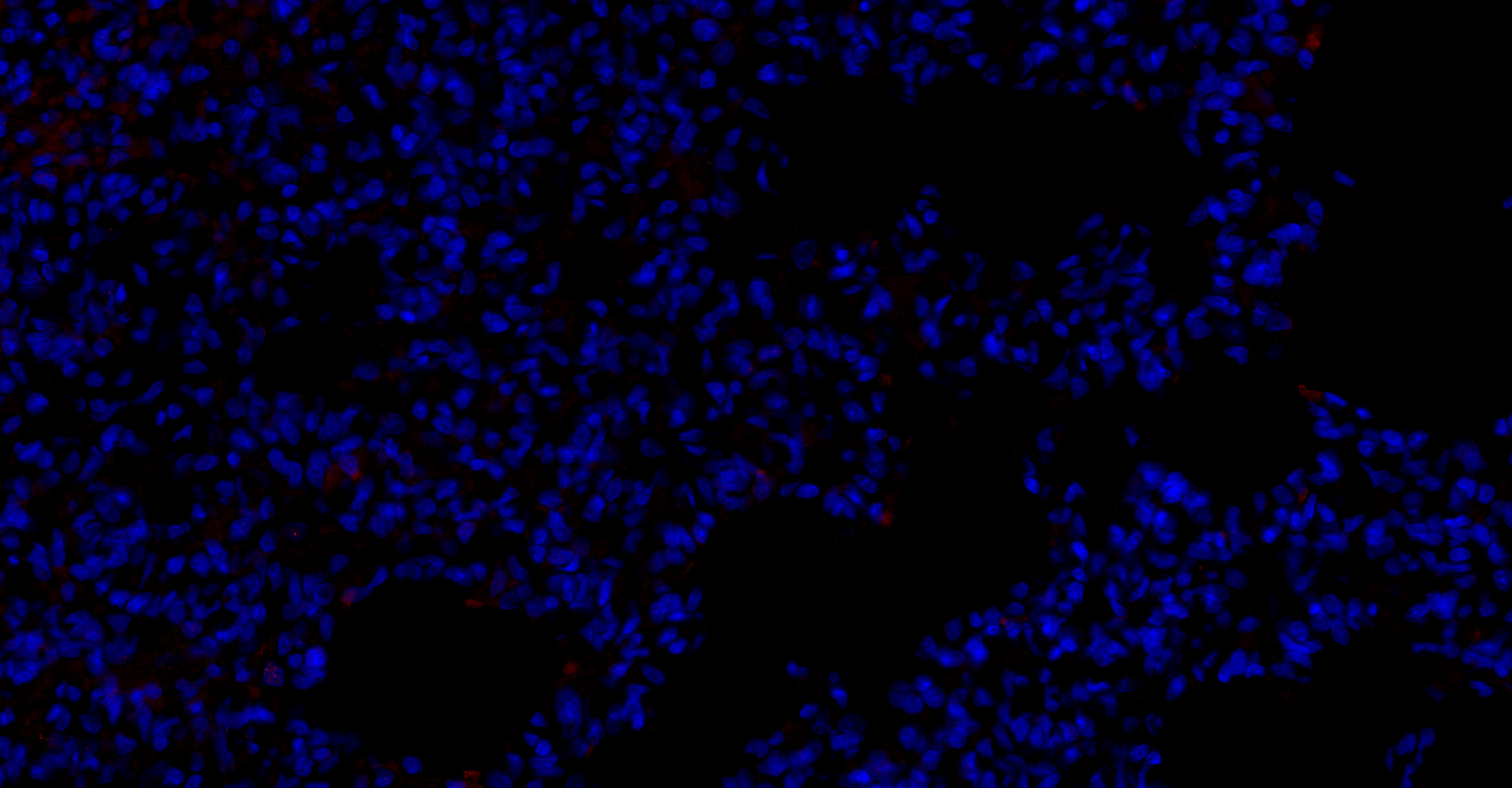

Supplement: Supplementary file 1 [file nutrients-17-02242-s001.zip › Figure S2 Original images/figure2-N-2 citH3 IF_40.0x.tif]

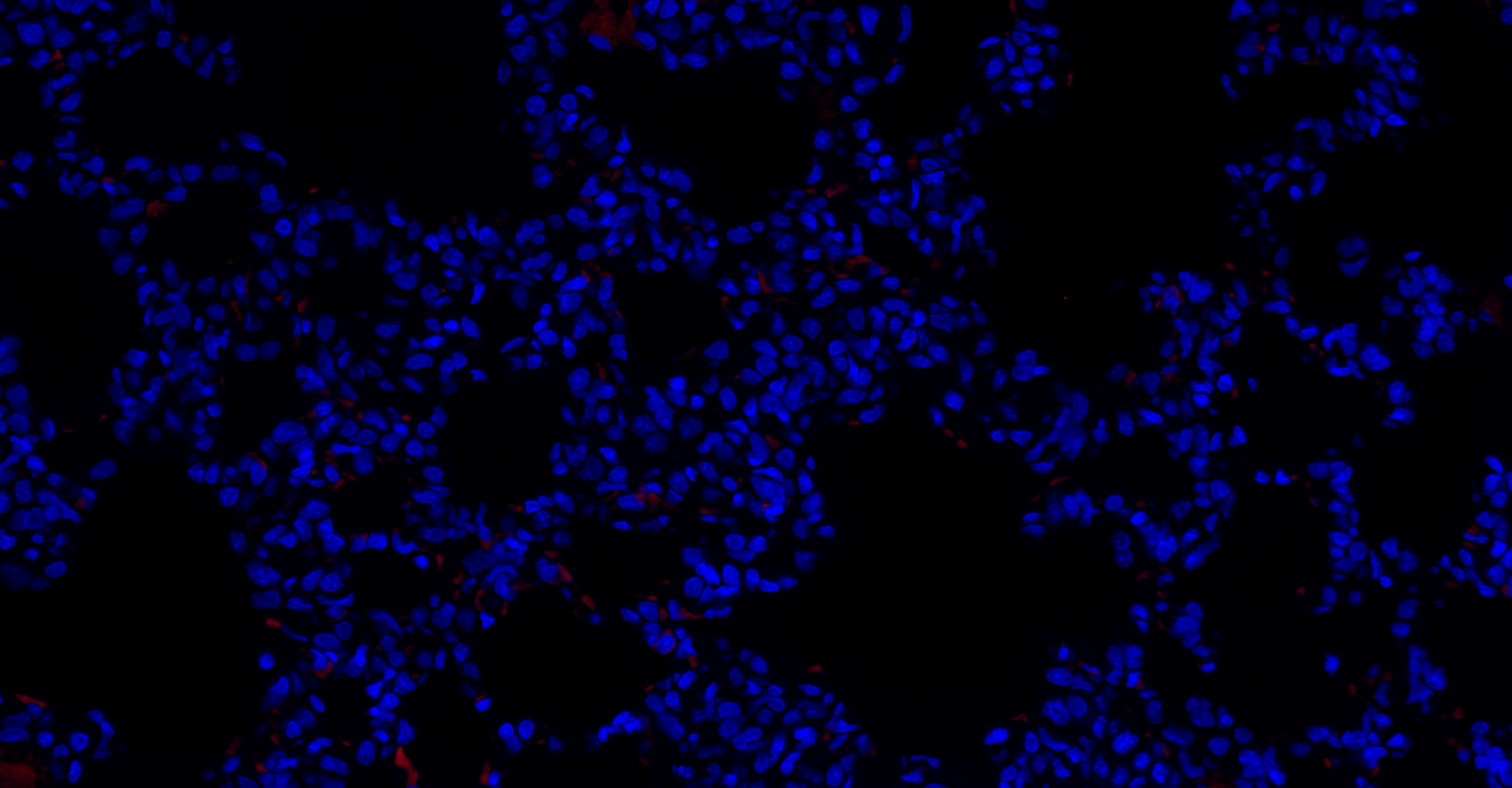

Supplement: Supplementary file 1 [file nutrients-17-02242-s001.zip › Figure S2 Original images/figure2-N-2 ly6g IF_40.0x.tif]

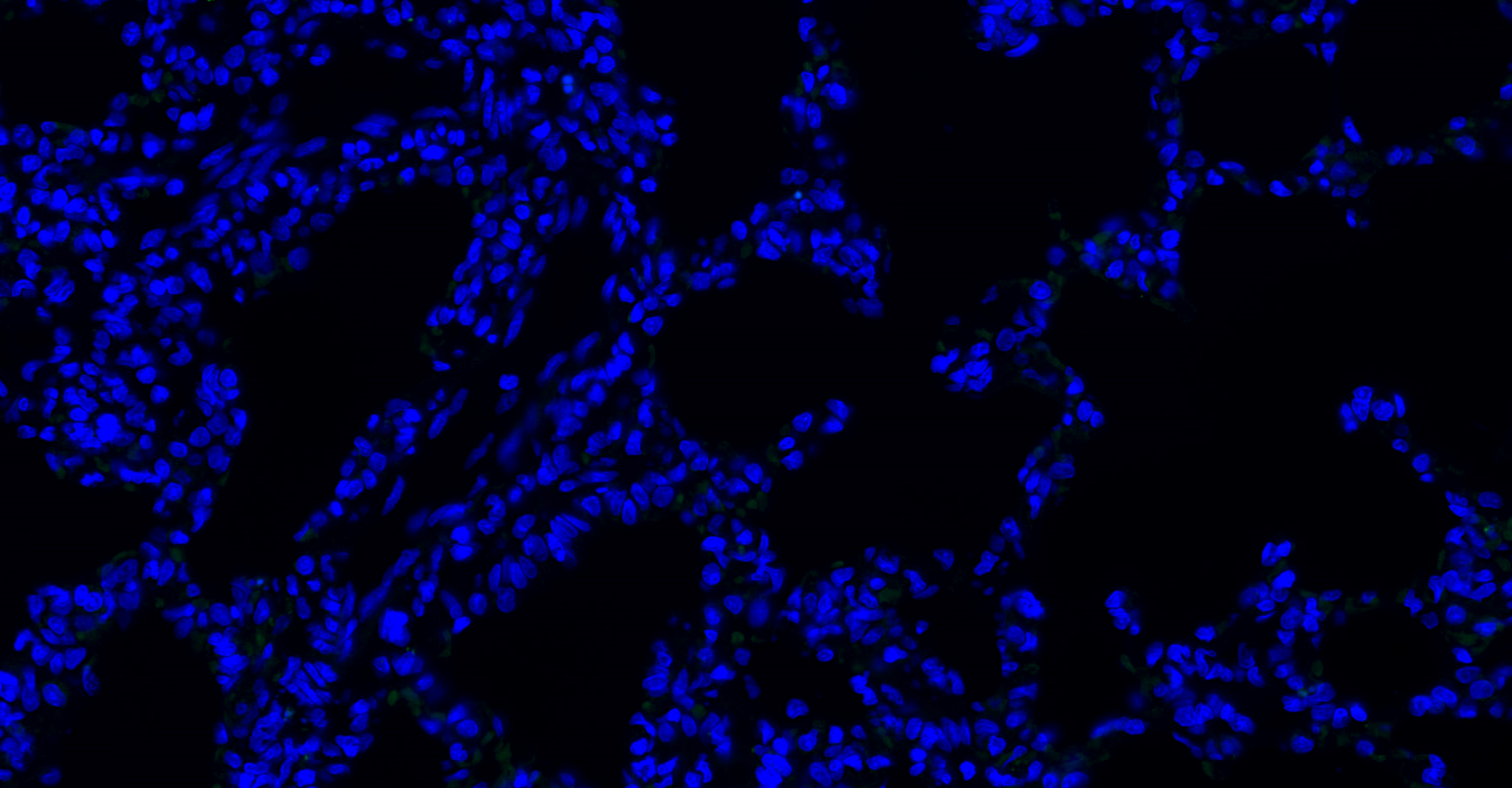

Supplement: Supplementary file 1 [file nutrients-17-02242-s001.zip › Figure S2 Original images/figure2-N-2 tunel IF_40.0x.tif]

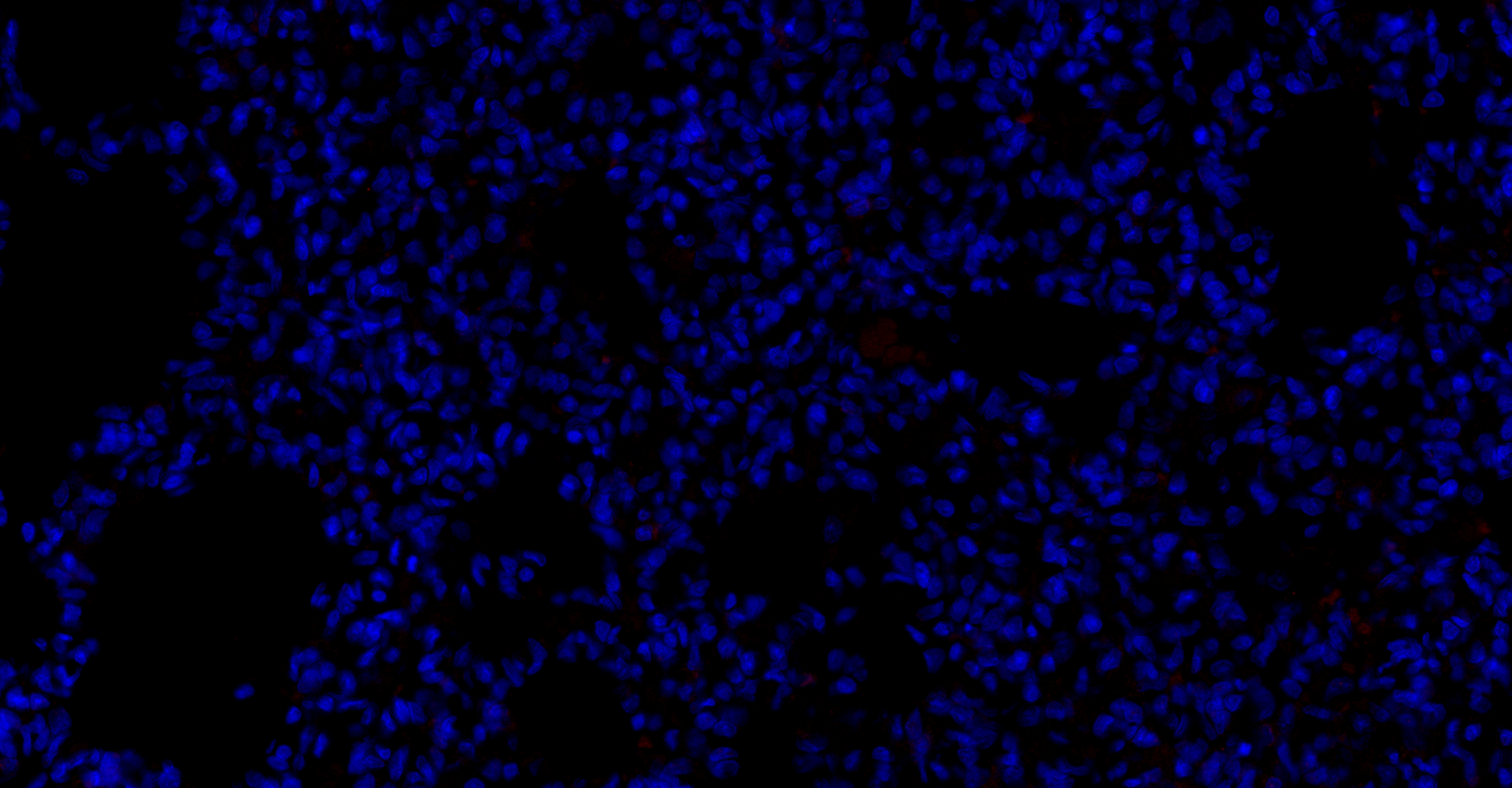

Supplement: Supplementary file 1 [file nutrients-17-02242-s001.zip › Figure S2 Original images/figure2-N-3 citH3 IF_40.0x.tif]

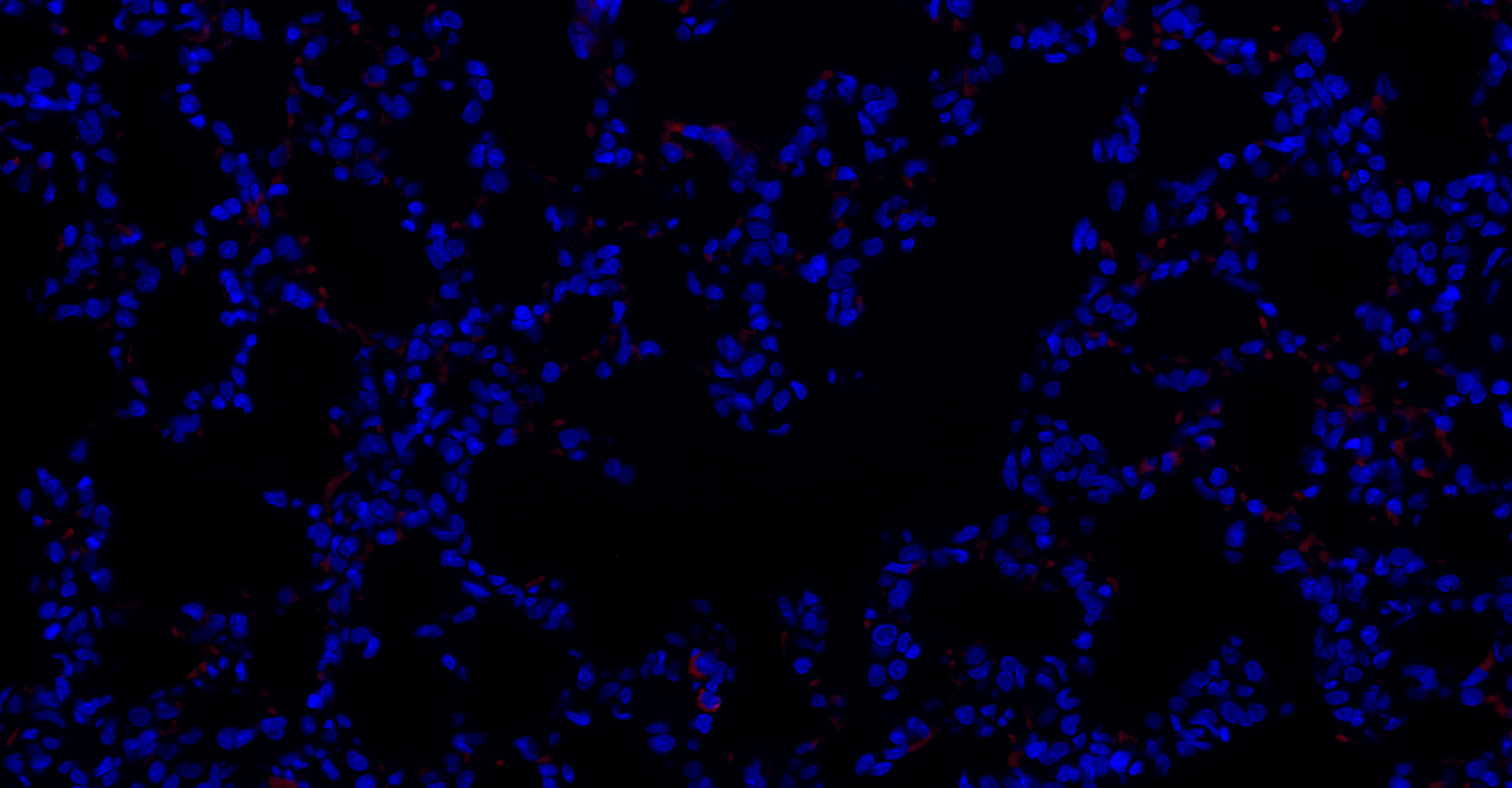

Supplement: Supplementary file 1 [file nutrients-17-02242-s001.zip › Figure S2 Original images/figure2-N-3 ly6g IF_40.0x.tif]

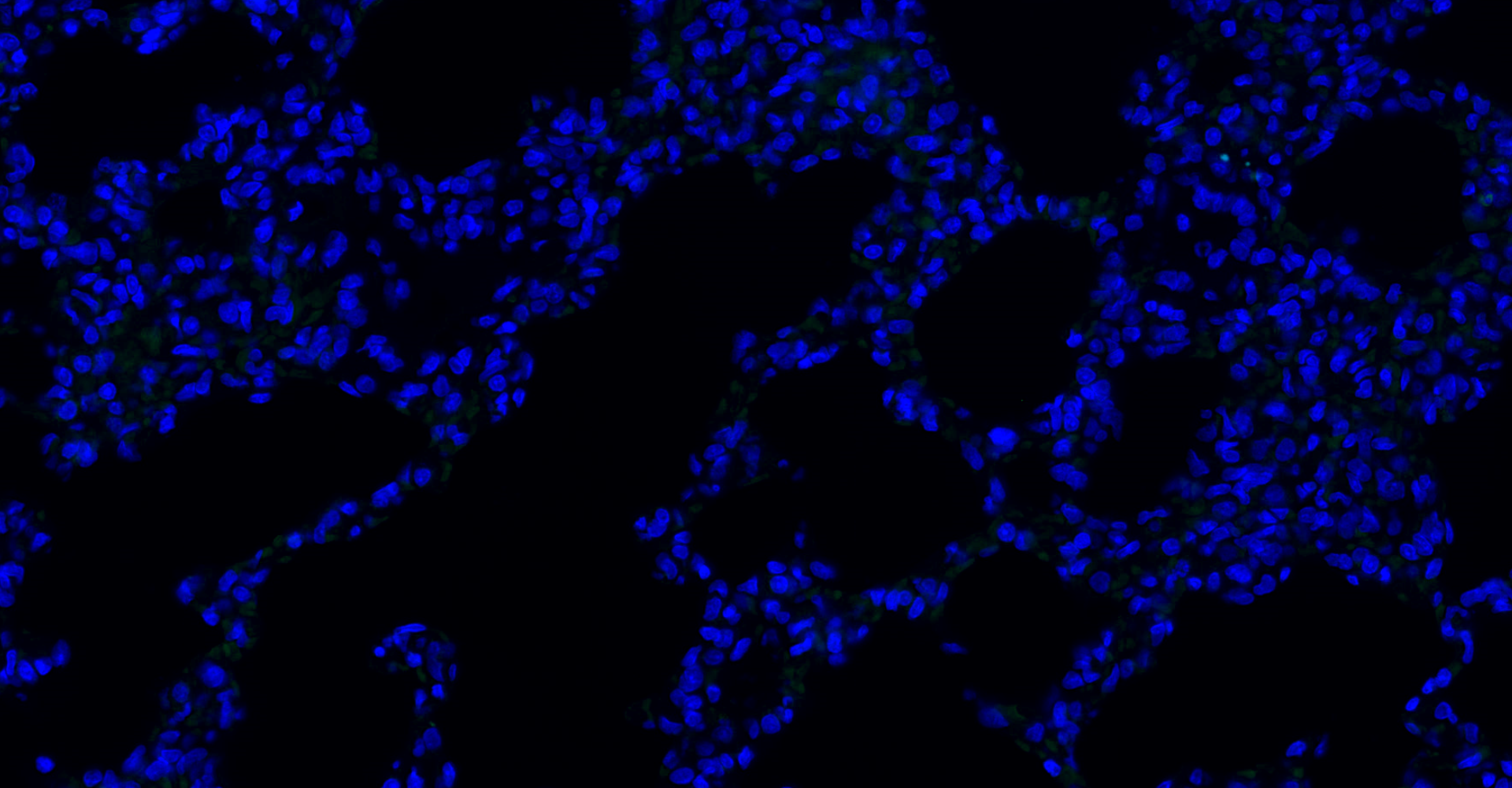

Supplement: Supplementary file 1 [file nutrients-17-02242-s001.zip › Figure S2 Original images/figure2-N-3 tunel IF_40.0x.tif]

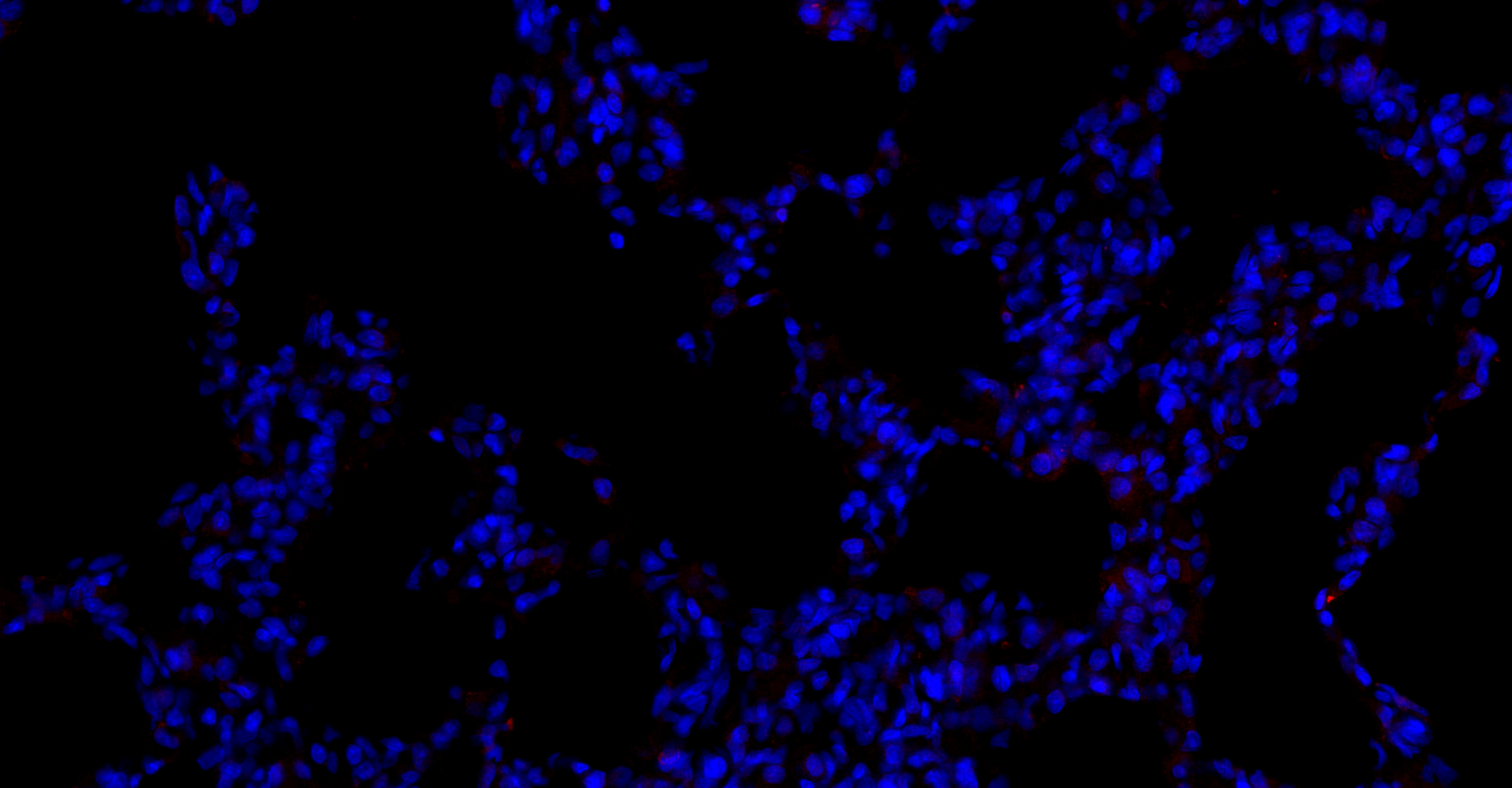

Supplement: Supplementary file 1 [file nutrients-17-02242-s001.zip › Figure S2 Original images/figure2-N-4 citH3 IF_40.0x.tif]

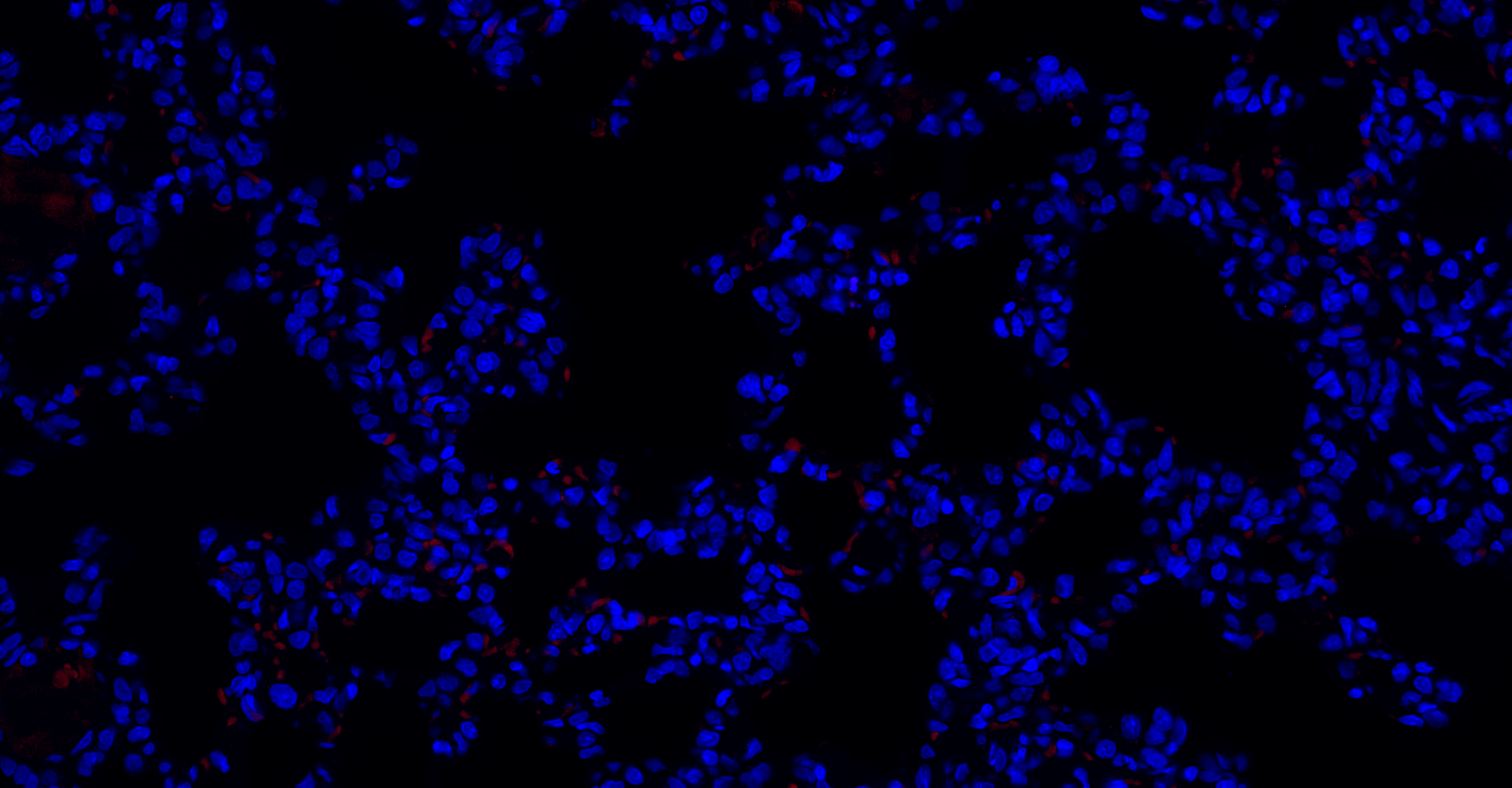

Supplement: Supplementary file 1 [file nutrients-17-02242-s001.zip › Figure S2 Original images/figure2-N-4 ly6g IF_40.0x.tif]

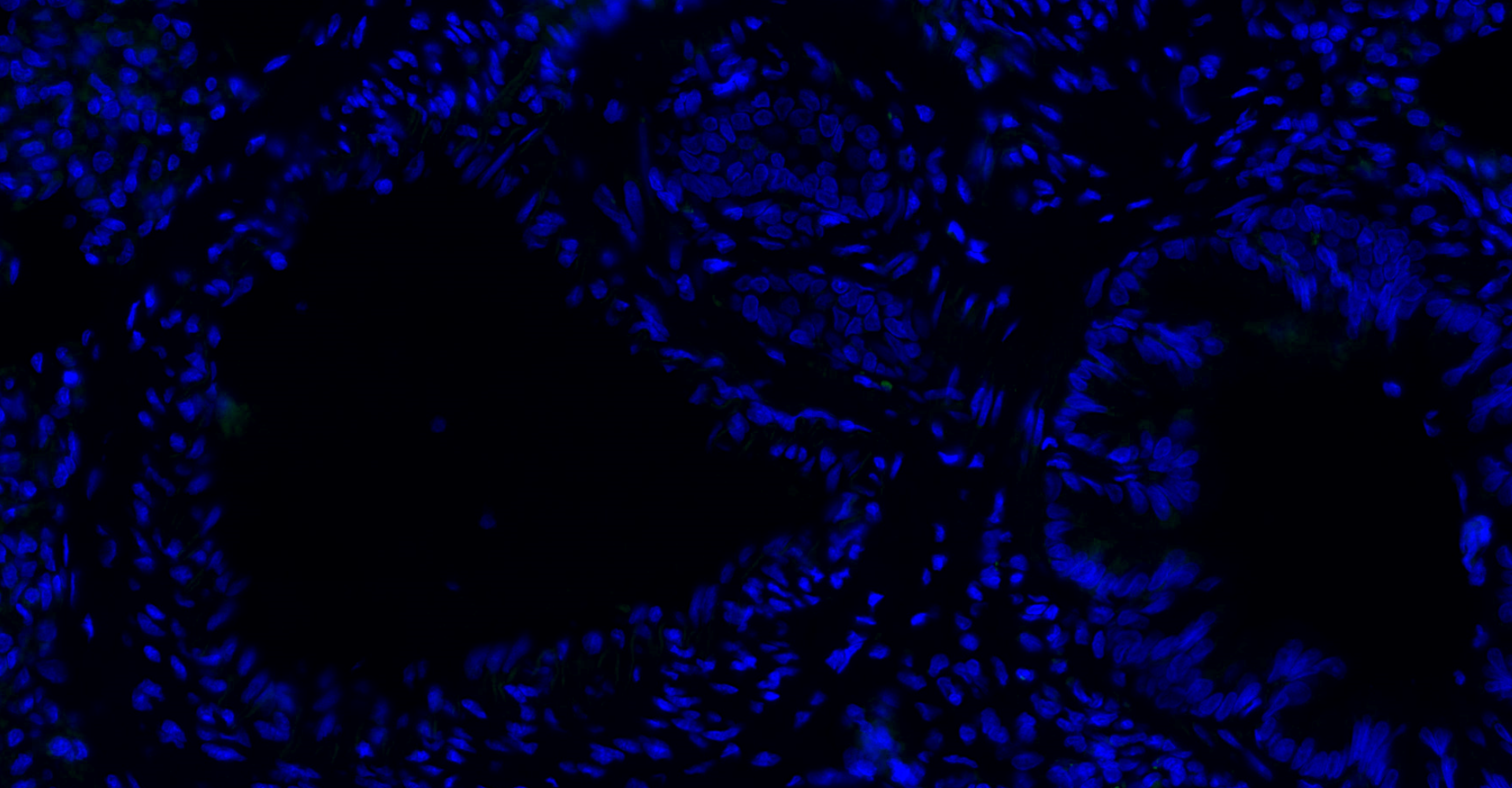

Supplement: Supplementary file 1 [file nutrients-17-02242-s001.zip › Figure S2 Original images/figure2-N-4 tunel IF_40.0x.tif]

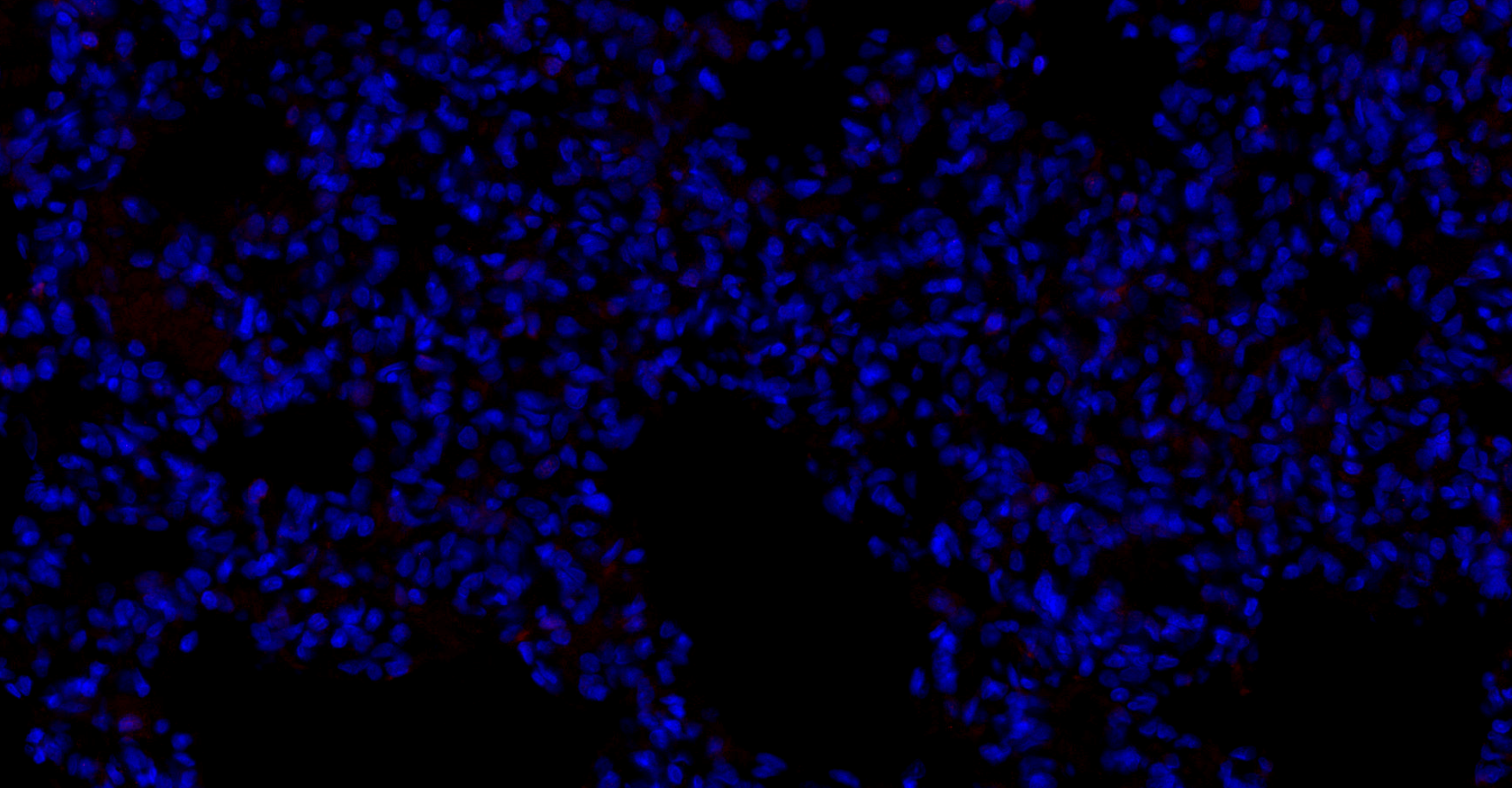

Supplement: Supplementary file 1 [file nutrients-17-02242-s001.zip › Figure S2 Original images/figure2-N-5 citH3 IF_40.0x.tif]

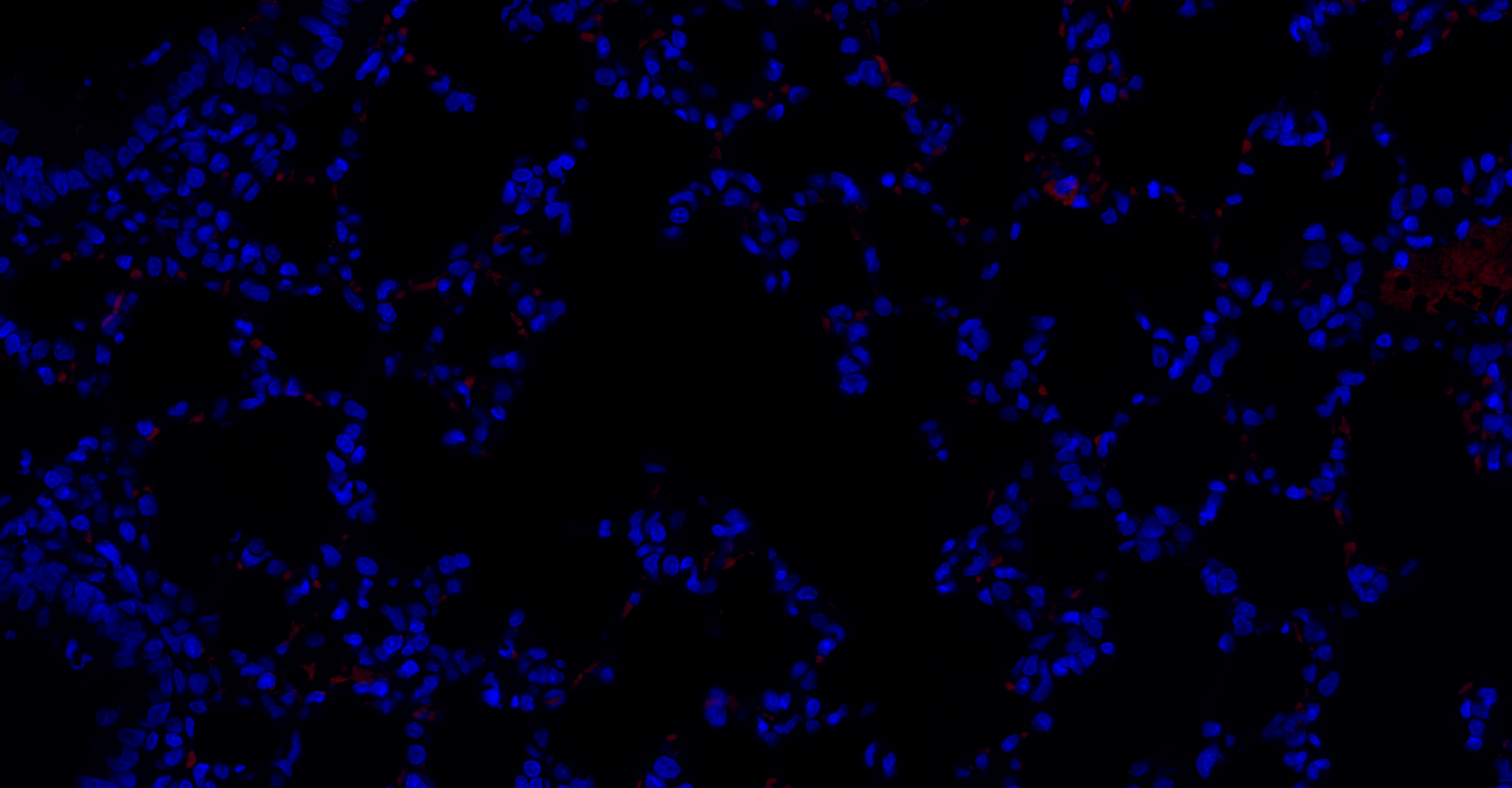

Supplement: Supplementary file 1 [file nutrients-17-02242-s001.zip › Figure S2 Original images/figure2-N-5 ly6g IF_40.0x.tif]

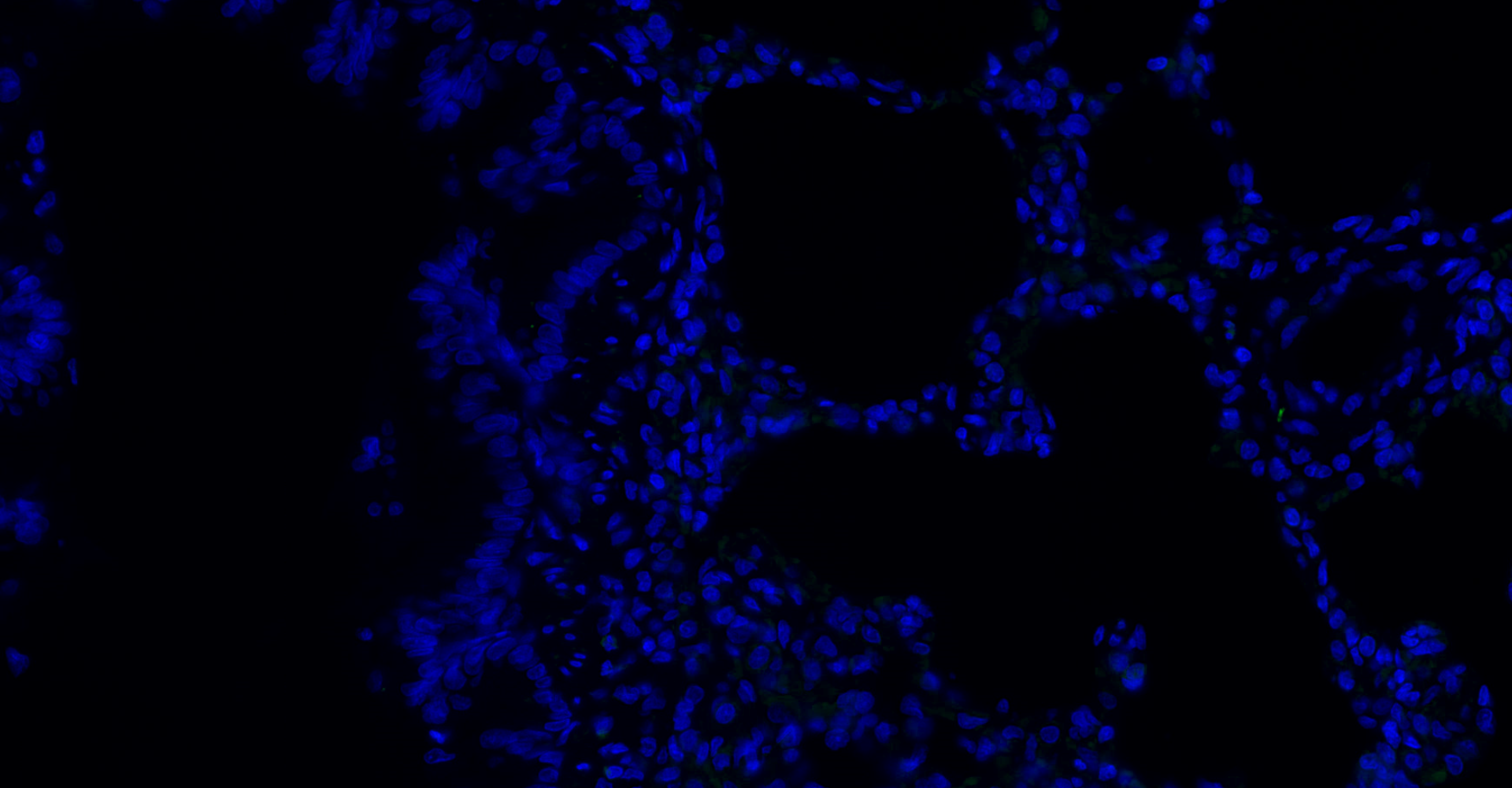

Supplement: Supplementary file 1 [file nutrients-17-02242-s001.zip › Figure S2 Original images/figure2-N-5 tunel IF_40.0x.tif]

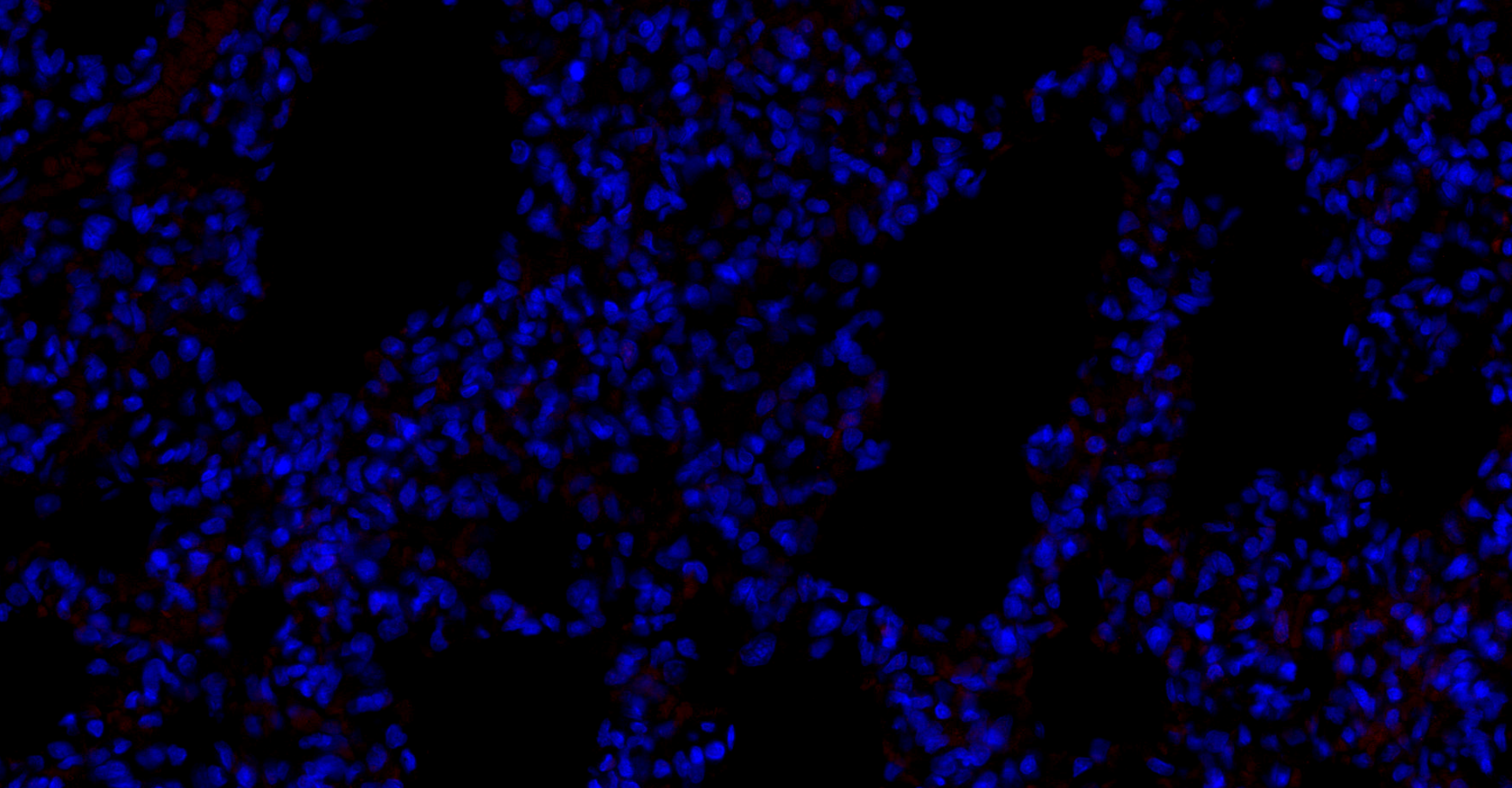

Supplement: Supplementary file 1 [file nutrients-17-02242-s001.zip › Figure S2 Original images/figure2-N-6 citH3 IF_40.0x.tif]

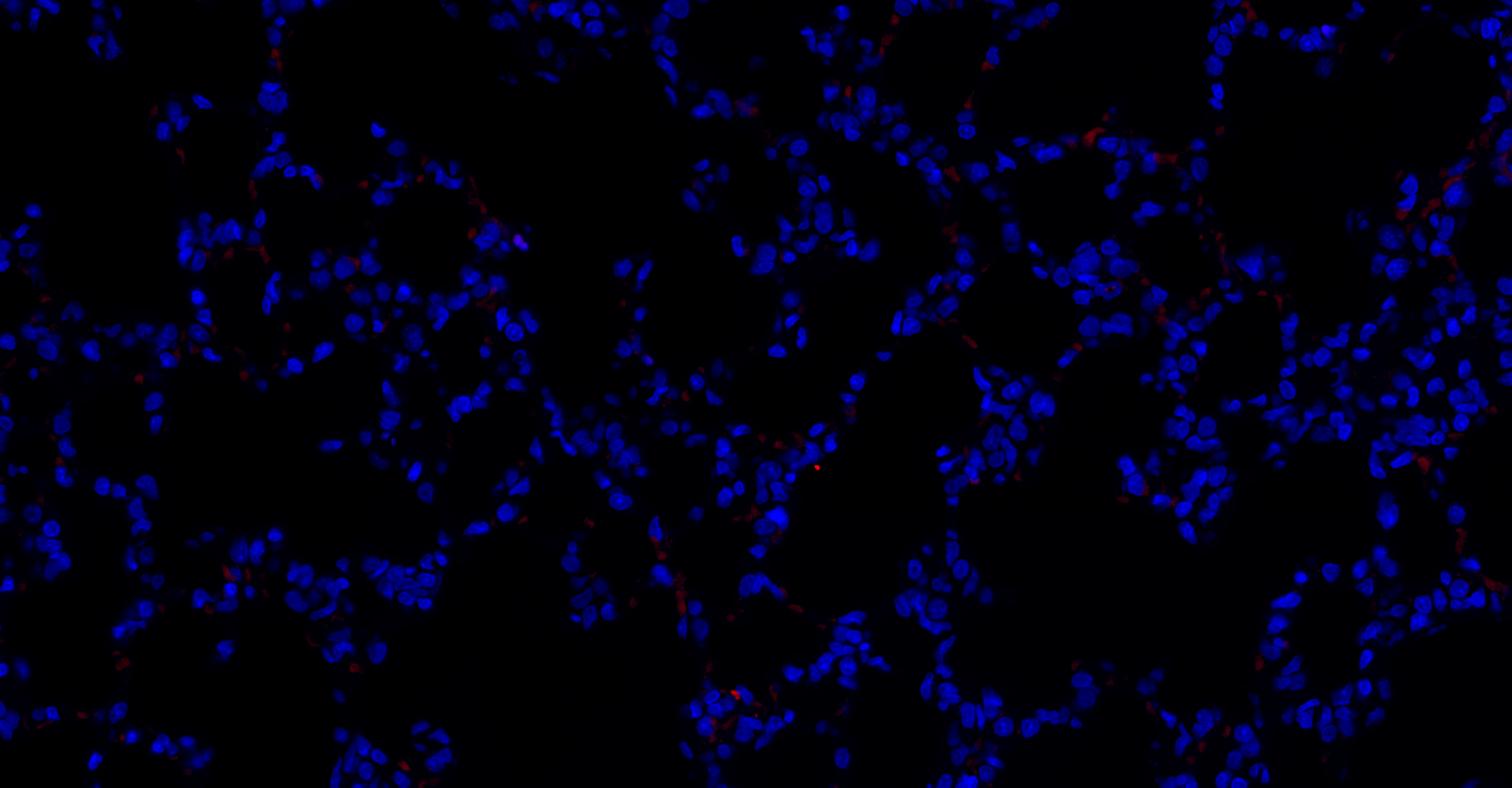

Supplement: Supplementary file 1 [file nutrients-17-02242-s001.zip › Figure S2 Original images/figure2-N-6 ly6g IF_40.0x.tif]

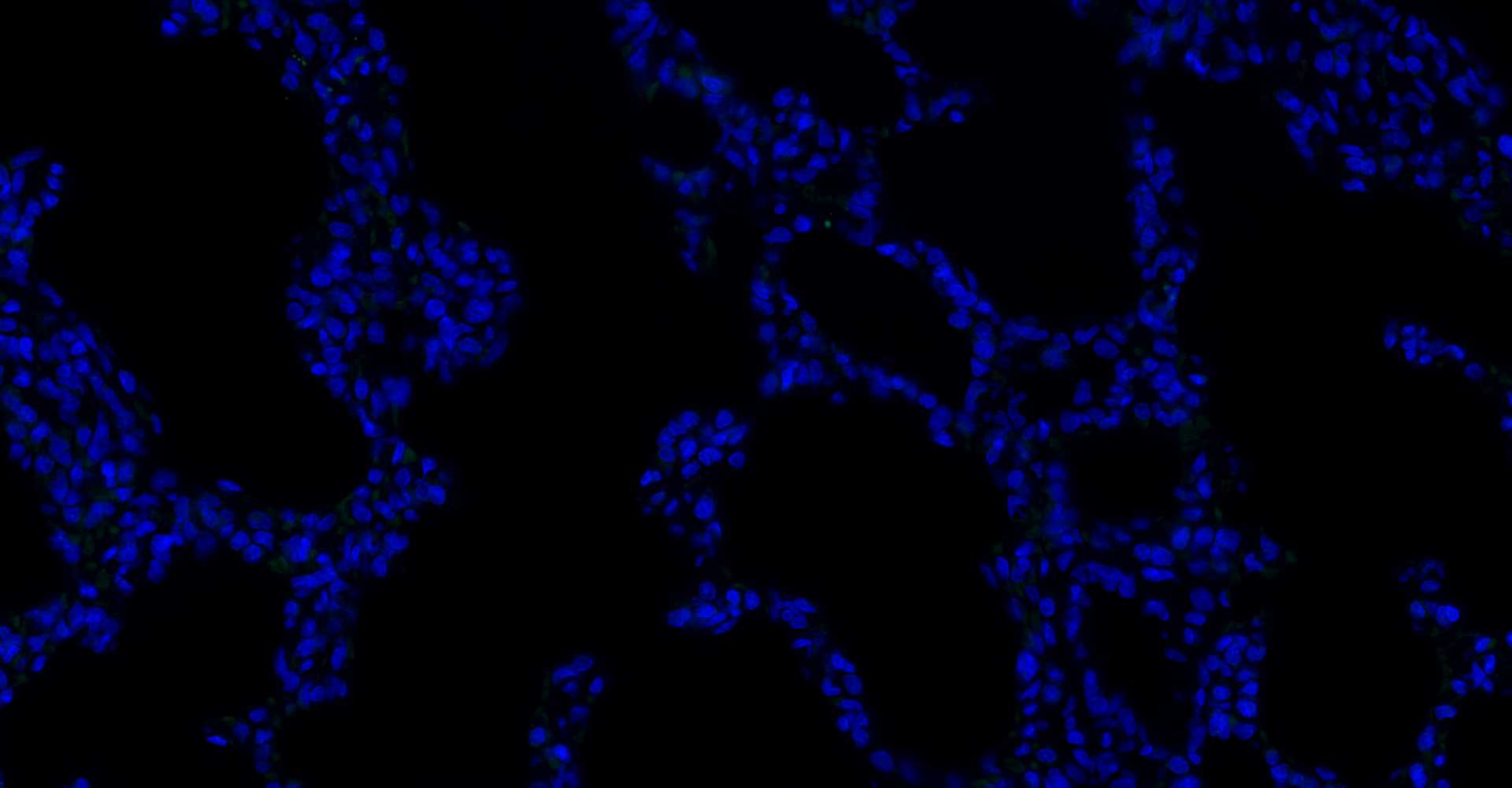

Supplement: Supplementary file 1 [file nutrients-17-02242-s001.zip › Figure S2 Original images/figure2-N-6 tunel IF_40.0x.tif]

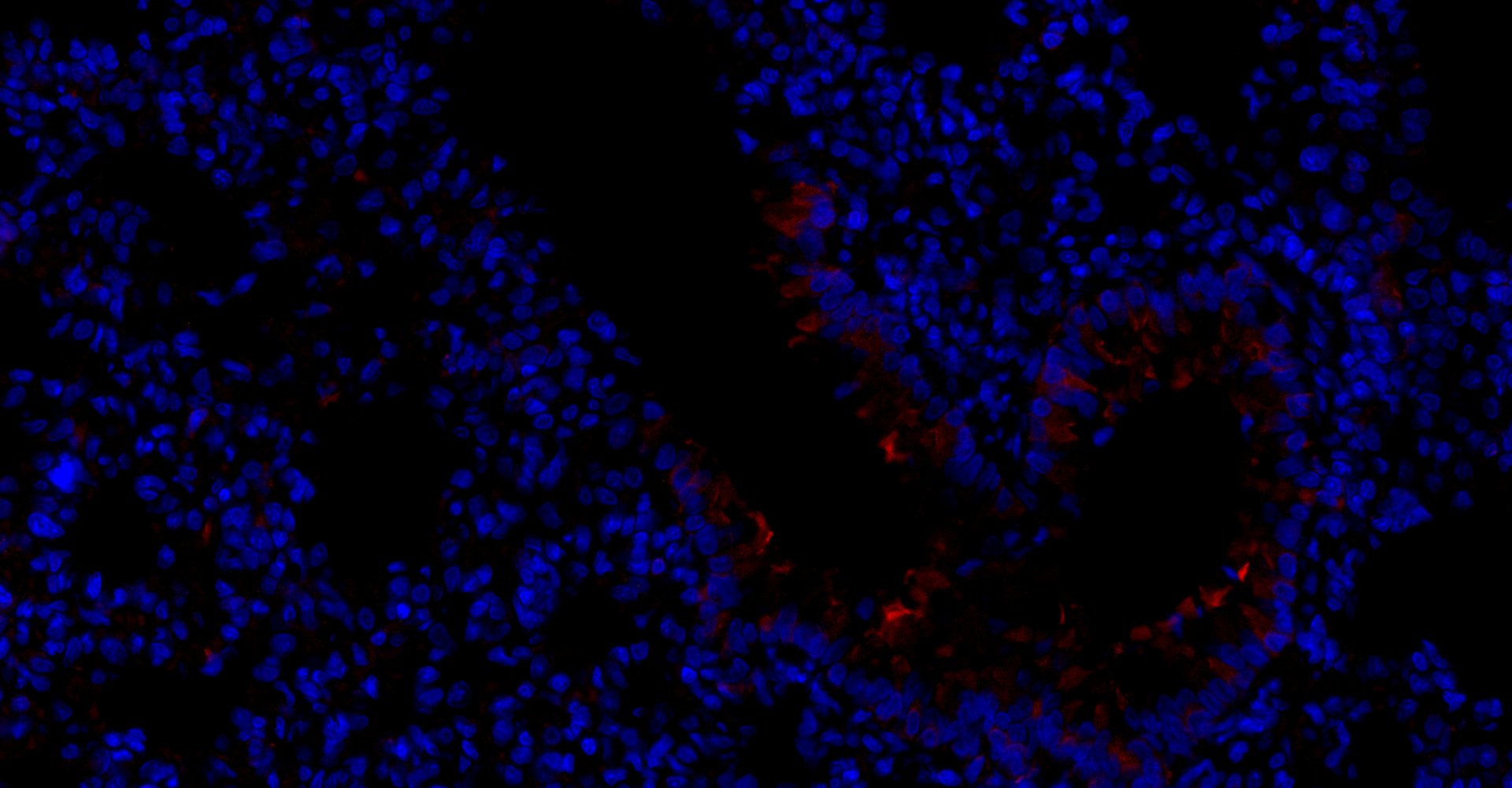

Supplement: Supplementary file 1 [file nutrients-17-02242-s001.zip › Figure S2 Original images/figure2-P-1 citH3 IF_40.0x.tif]

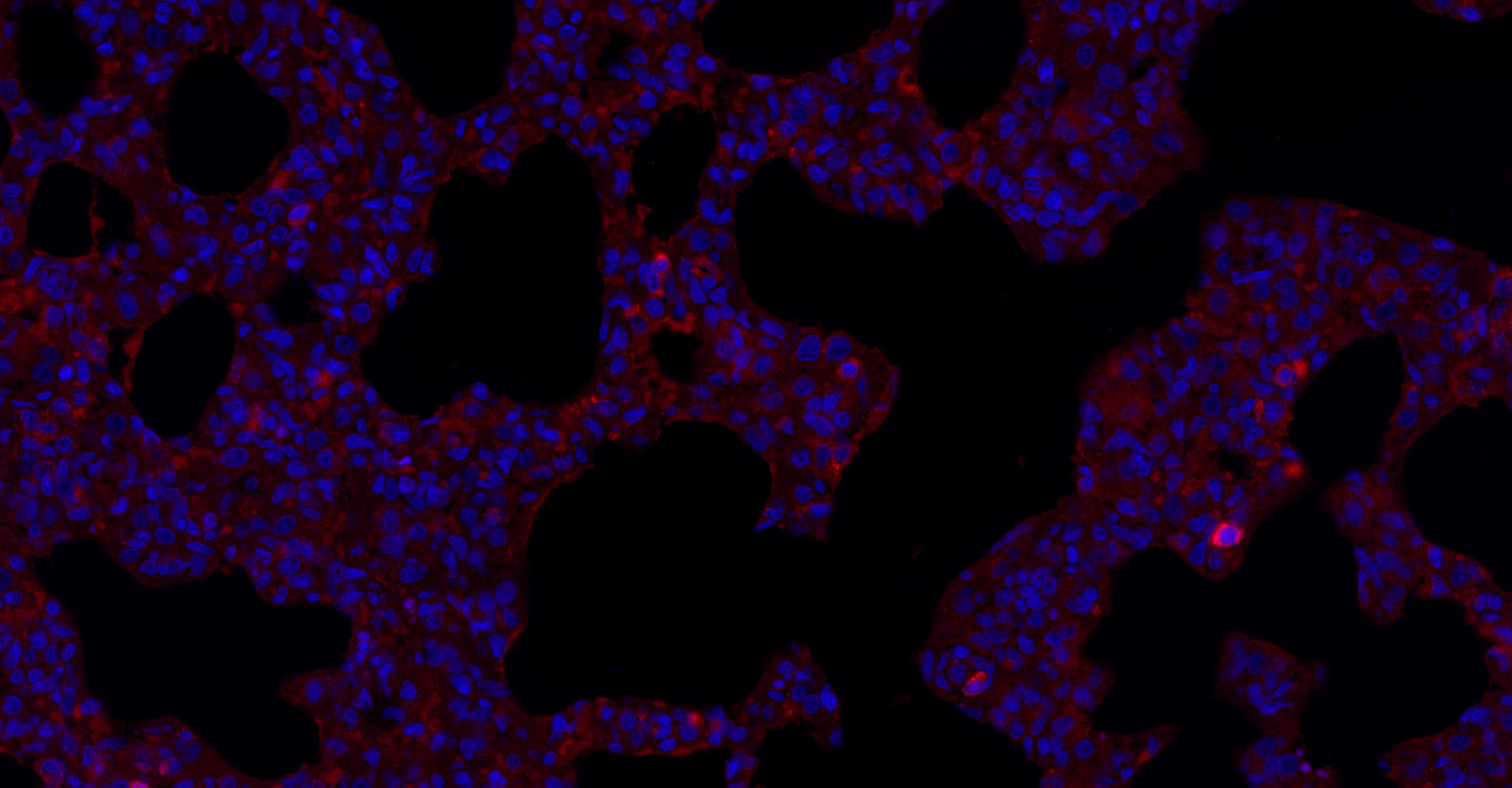

Supplement: Supplementary file 1 [file nutrients-17-02242-s001.zip › Figure S2 Original images/figure2-P-1 ly6g IF_40.0x.tif]

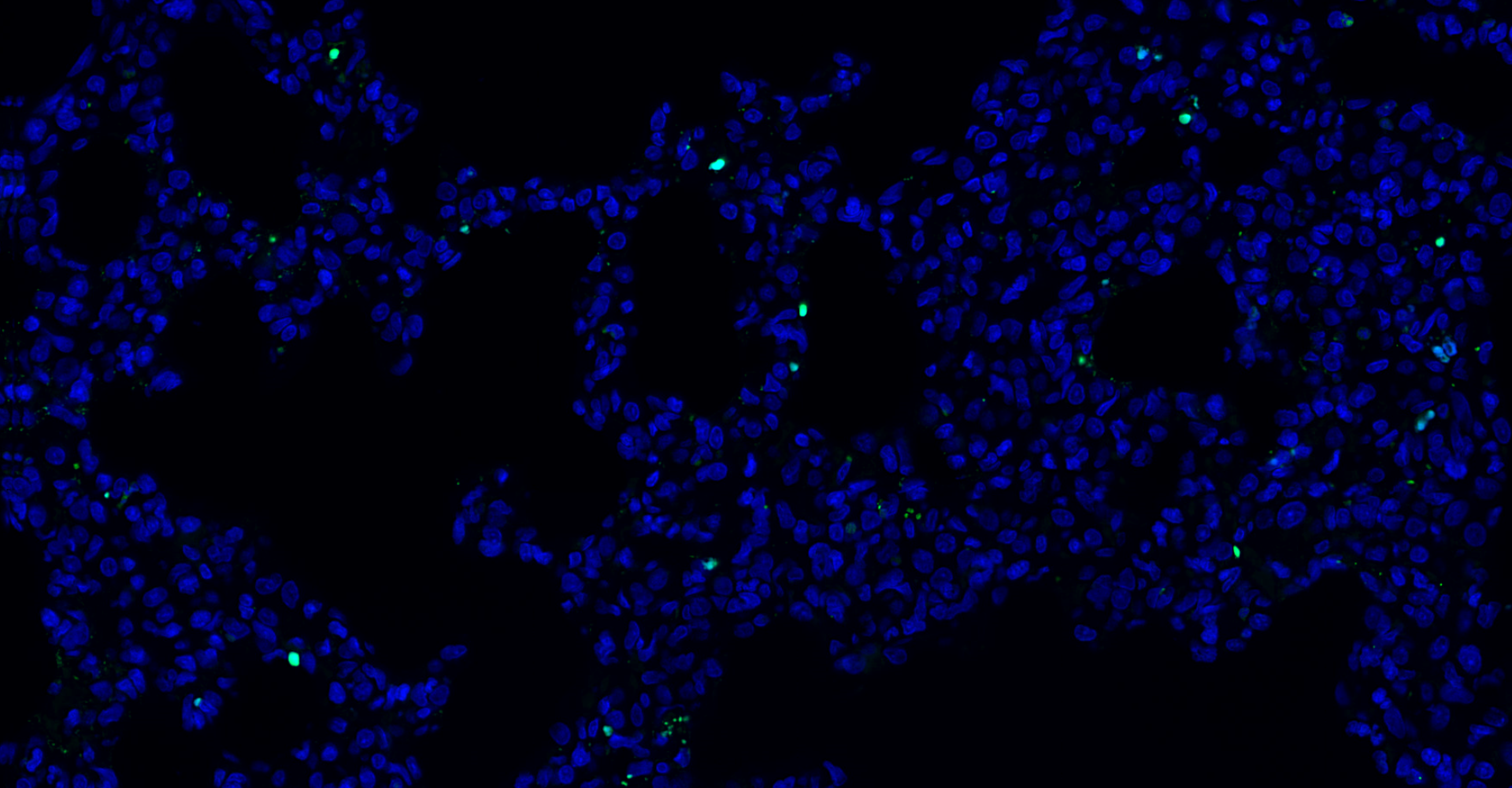

Supplement: Supplementary file 1 [file nutrients-17-02242-s001.zip › Figure S2 Original images/figure2-P-1 tunel IF_40.0x.tif]

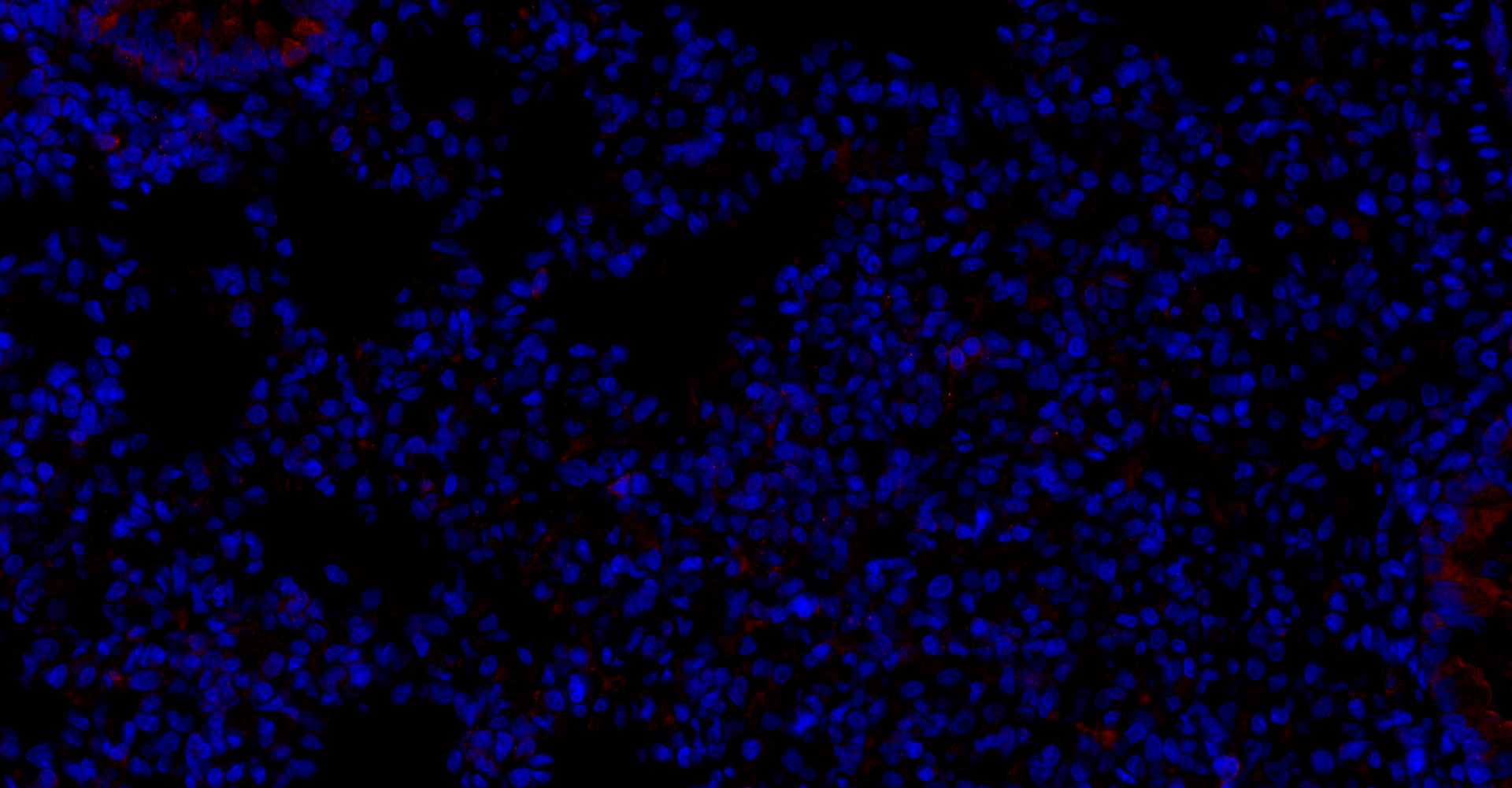

Supplement: Supplementary file 1 [file nutrients-17-02242-s001.zip › Figure S2 Original images/figure2-P-2 citH3 IF_40.0x.tif]

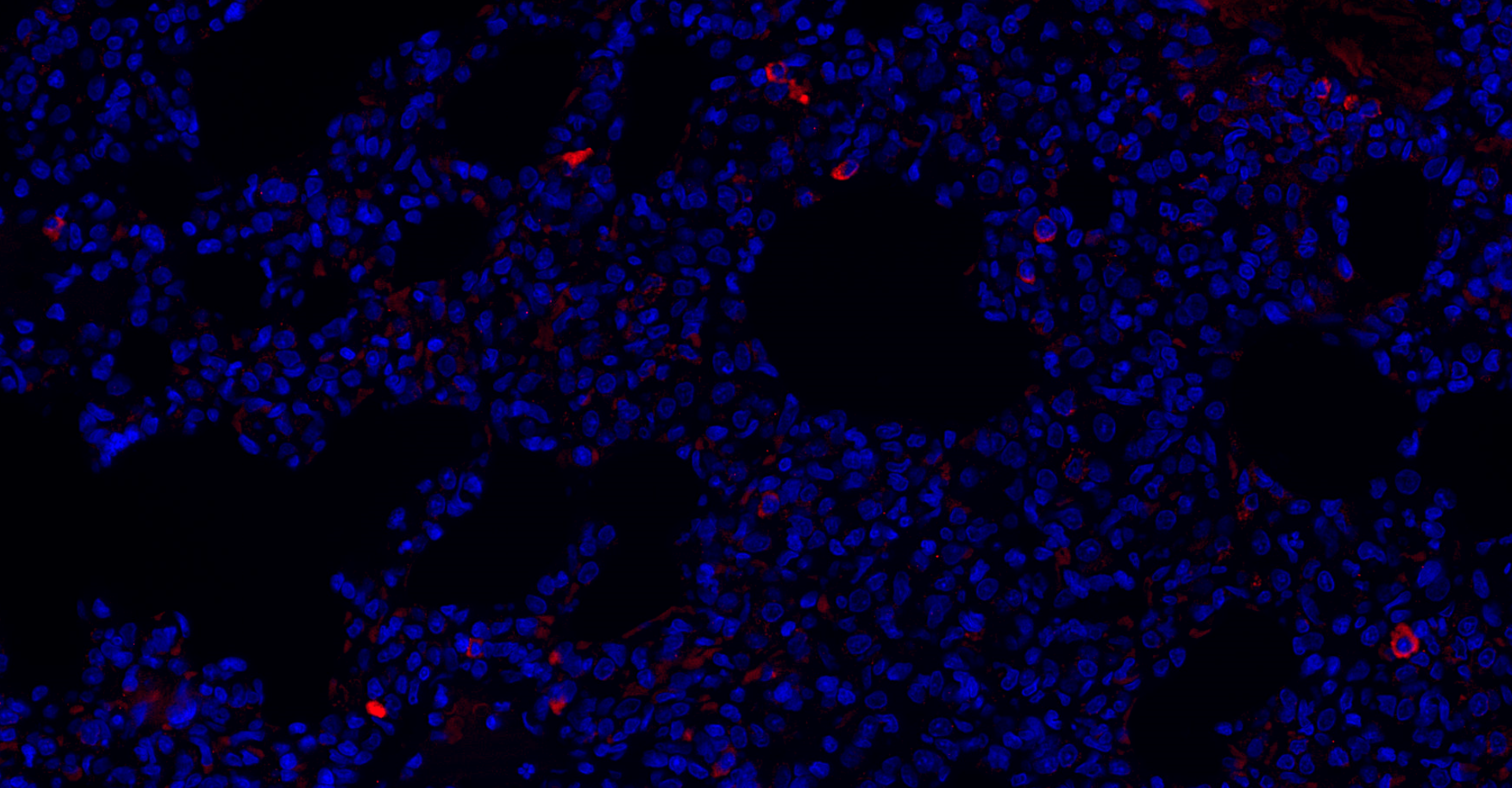

Supplement: Supplementary file 1 [file nutrients-17-02242-s001.zip › Figure S2 Original images/figure2-P-2 ly6g IF_40.0x.tif]

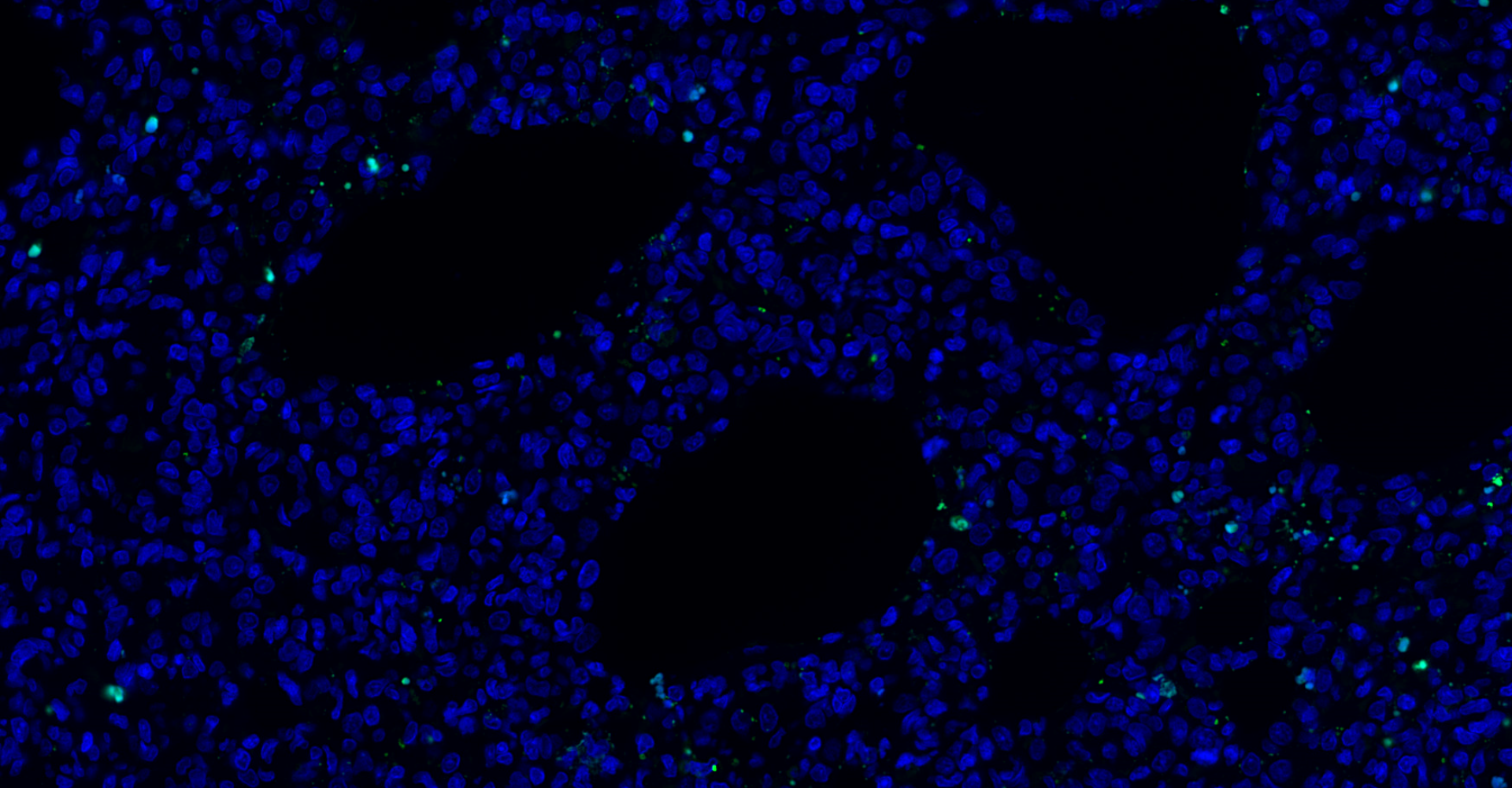

Supplement: Supplementary file 1 [file nutrients-17-02242-s001.zip › Figure S2 Original images/figure2-P-2 tunel IF_40.0x.tif]

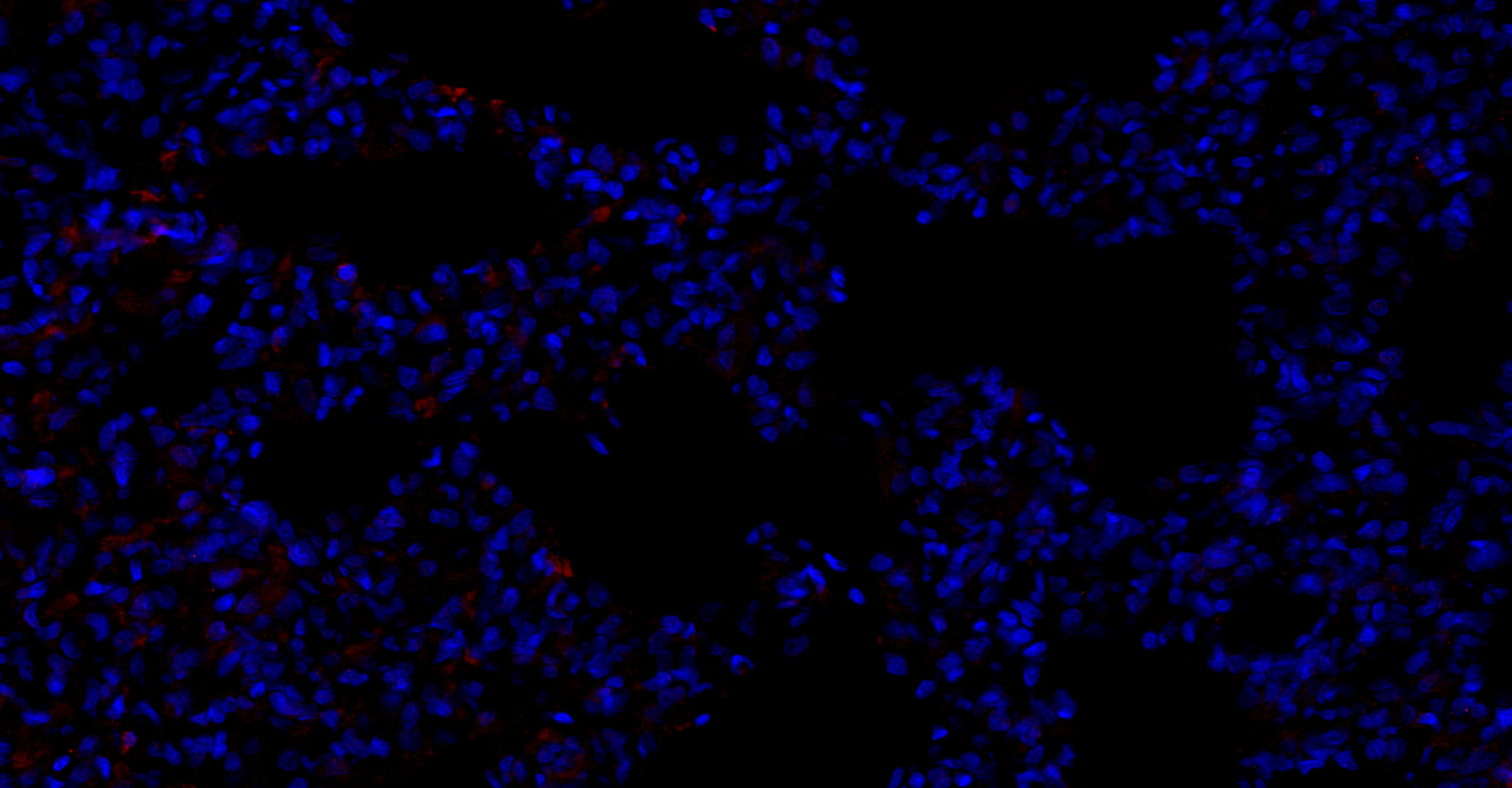

Supplement: Supplementary file 1 [file nutrients-17-02242-s001.zip › Figure S2 Original images/figure2-P-3 citH3 IF_40.0x.tif]

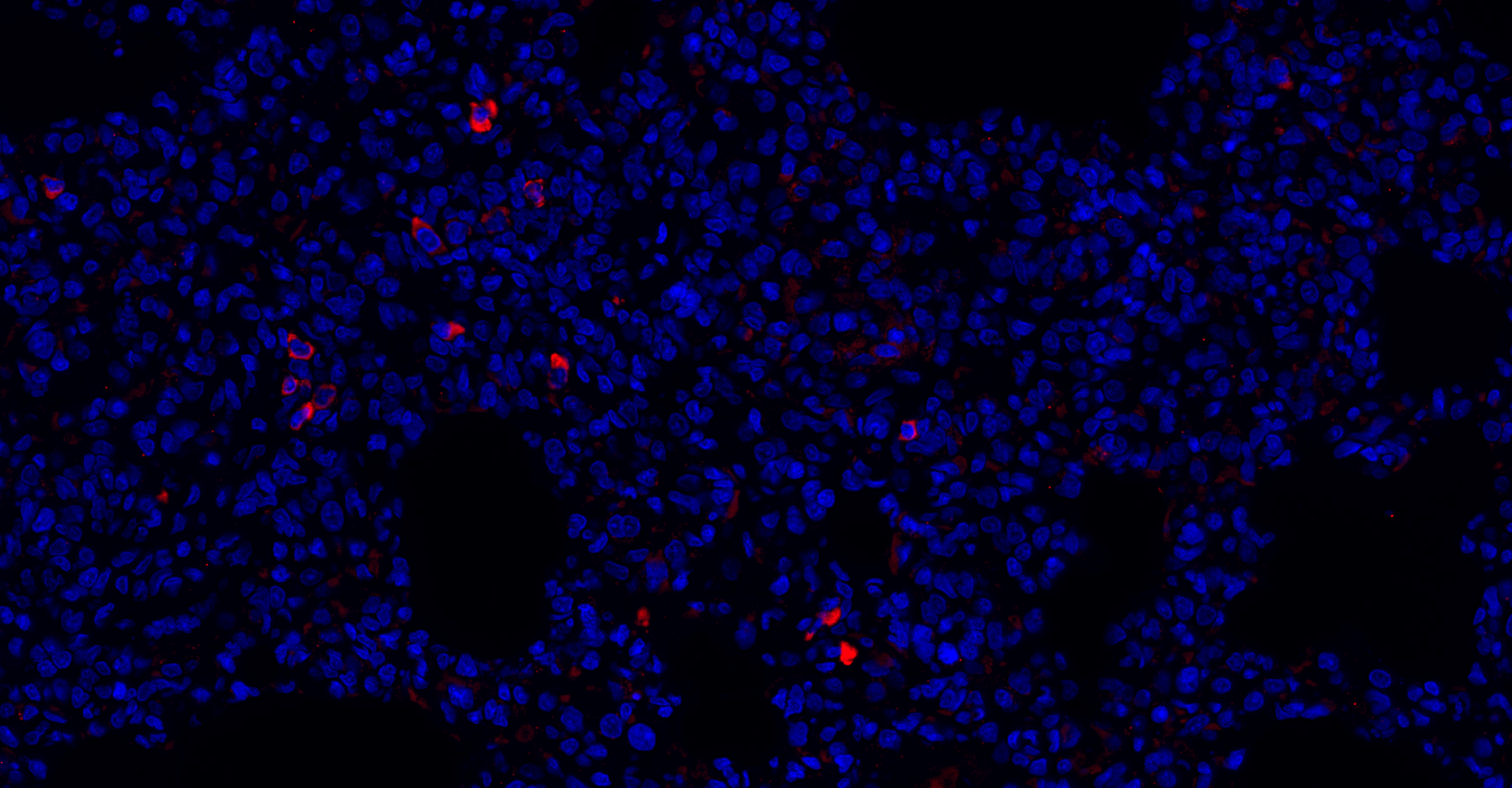

Supplement: Supplementary file 1 [file nutrients-17-02242-s001.zip › Figure S2 Original images/figure2-P-3 ly6g IF_40.0x.tif]

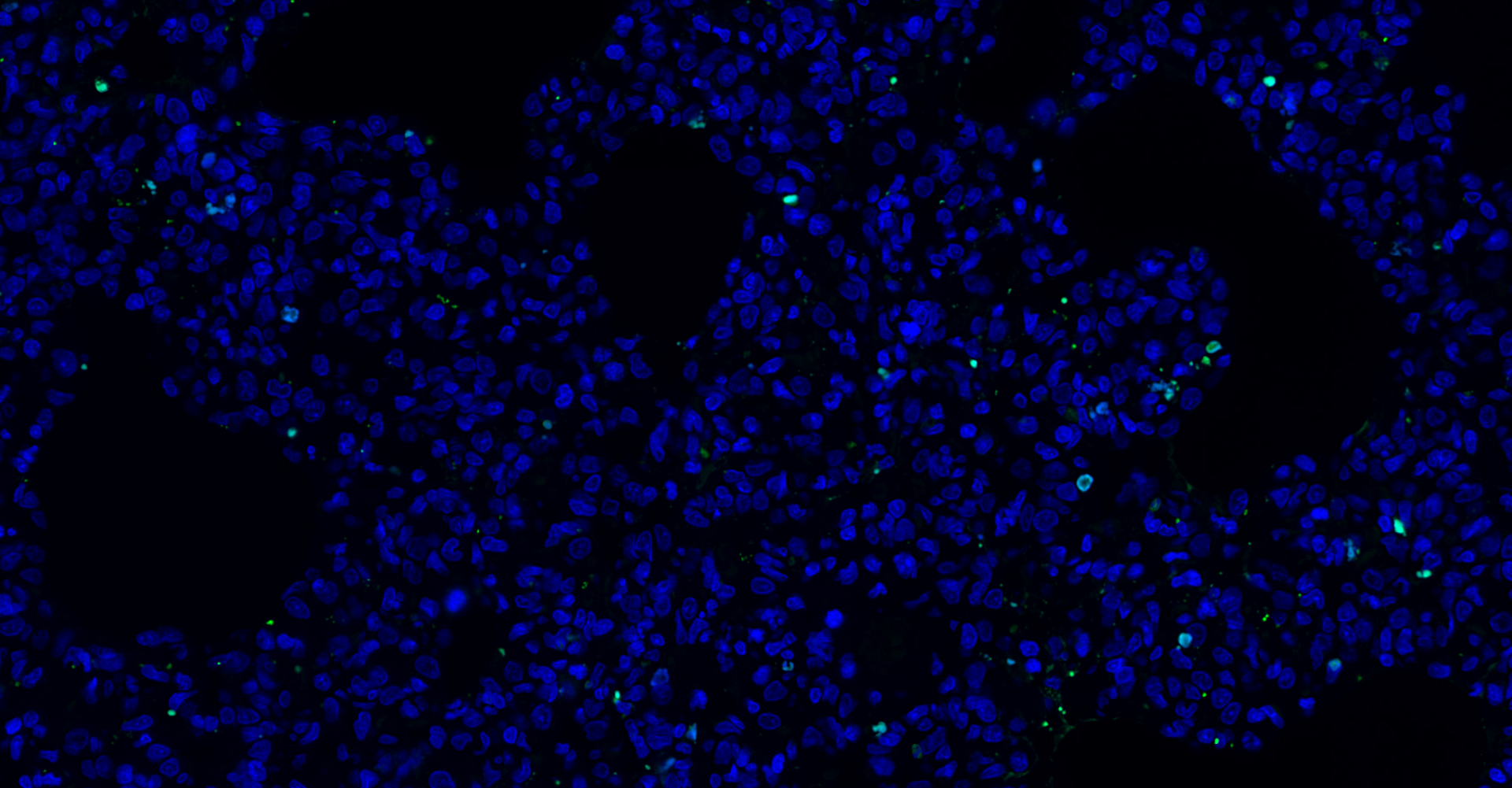

Supplement: Supplementary file 1 [file nutrients-17-02242-s001.zip › Figure S2 Original images/figure2-P-3 tunel IF_40.0x.tif]

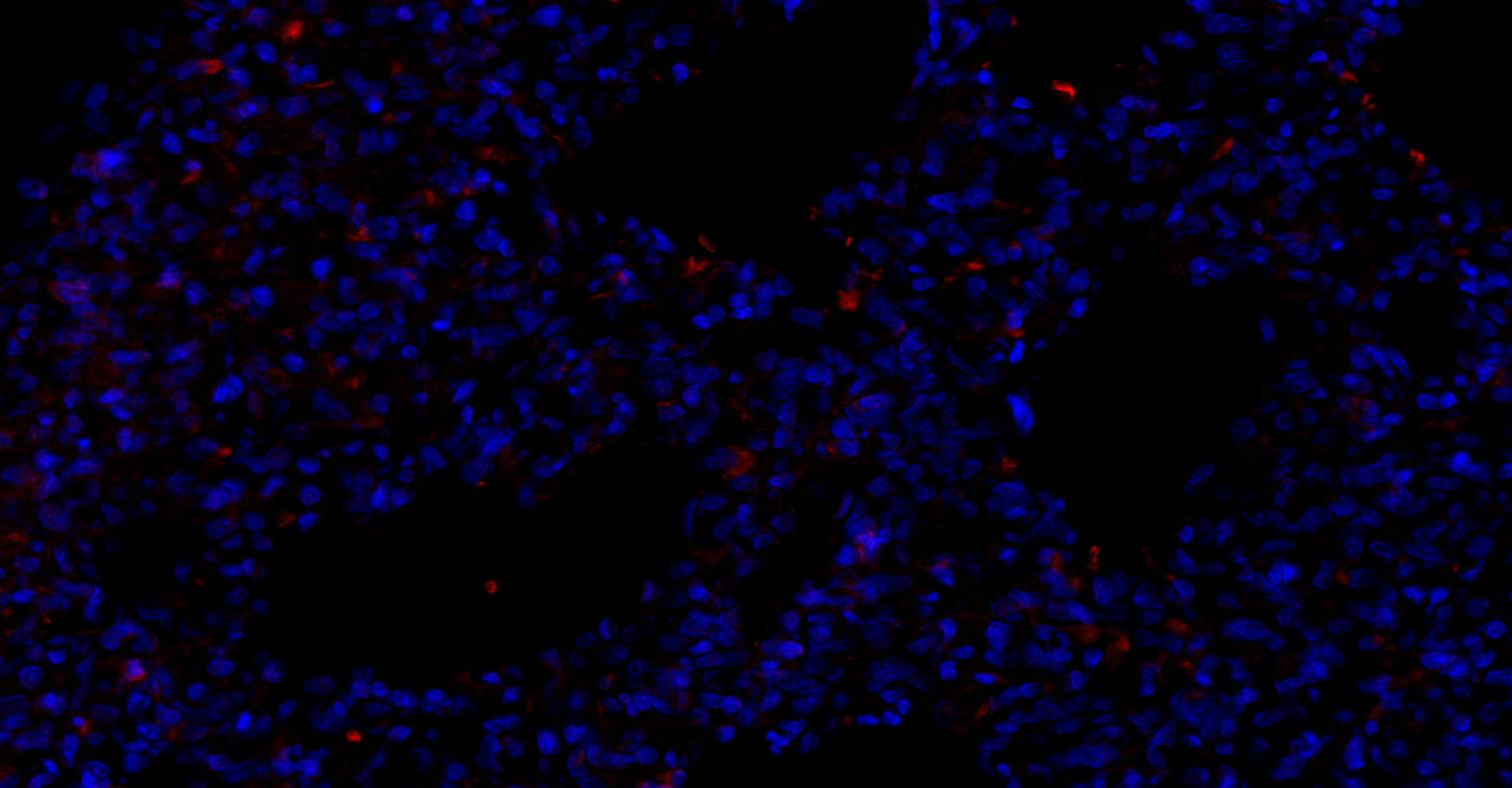

Supplement: Supplementary file 1 [file nutrients-17-02242-s001.zip › Figure S2 Original images/figure2-P-4 citH3 IF_40.0x.tif]

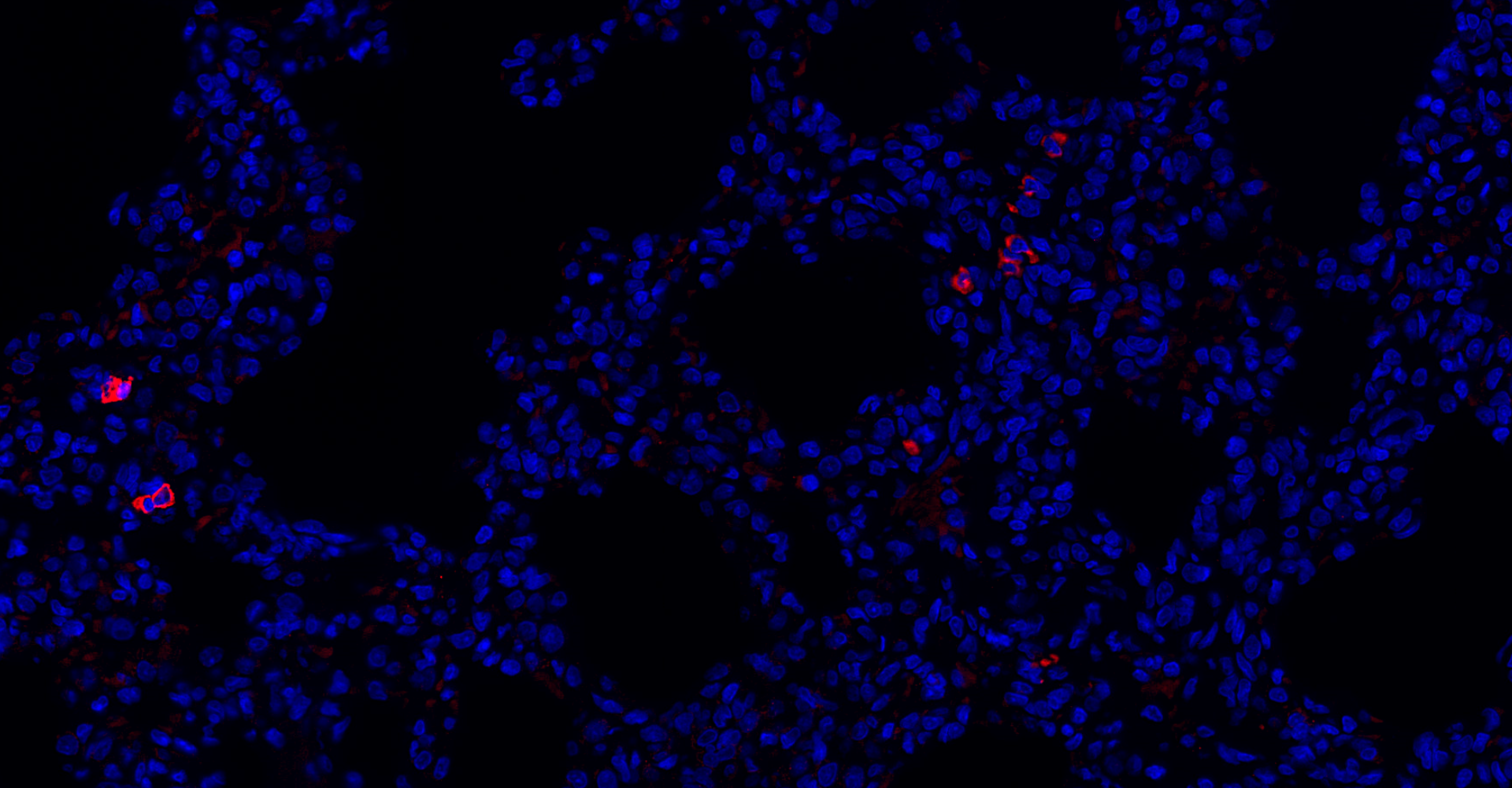

Supplement: Supplementary file 1 [file nutrients-17-02242-s001.zip › Figure S2 Original images/figure2-P-4 ly6g IF_40.0x.tif]

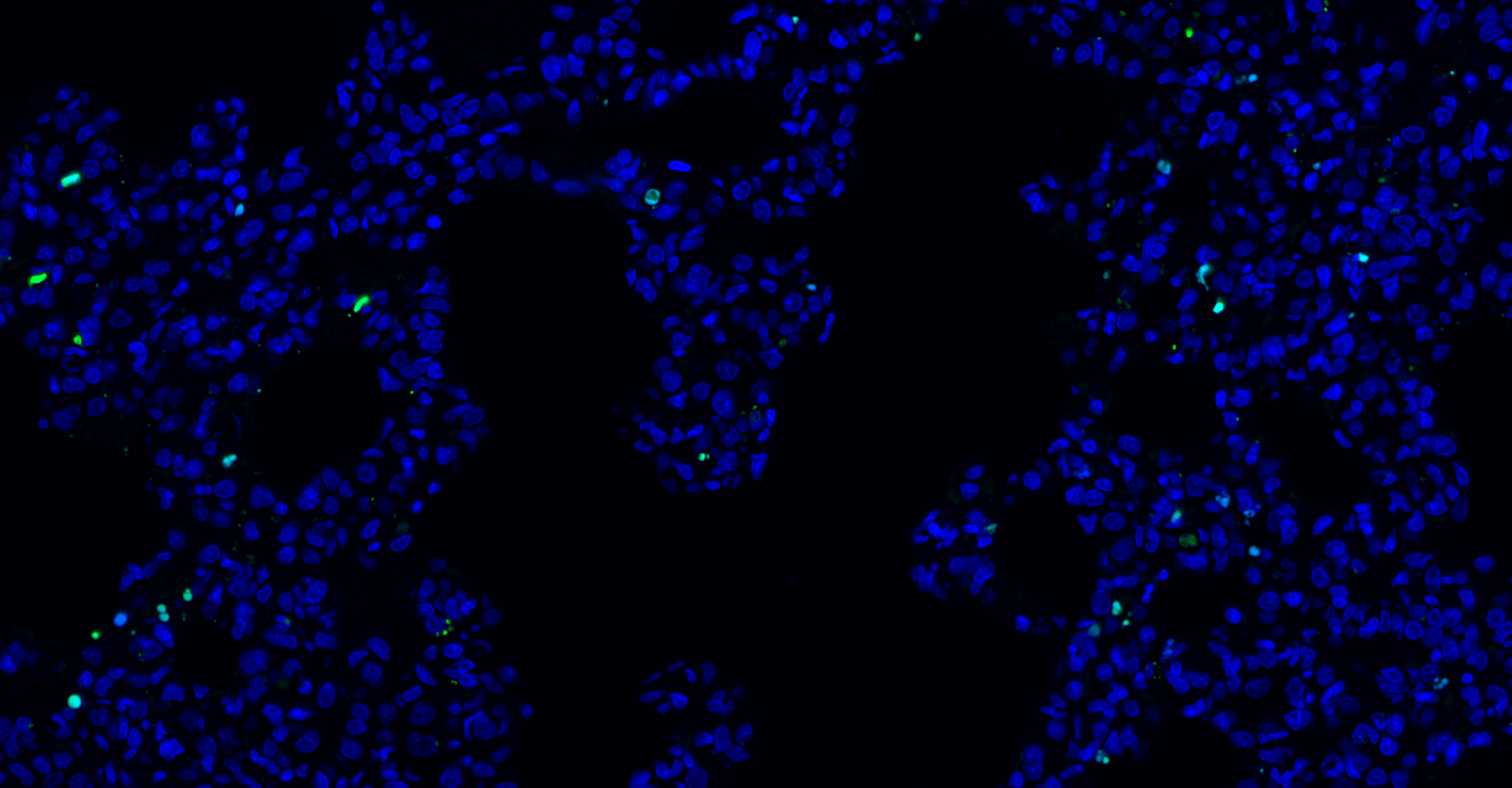

Supplement: Supplementary file 1 [file nutrients-17-02242-s001.zip › Figure S2 Original images/figure2-P-4 tunel IF_40.0x.tif]

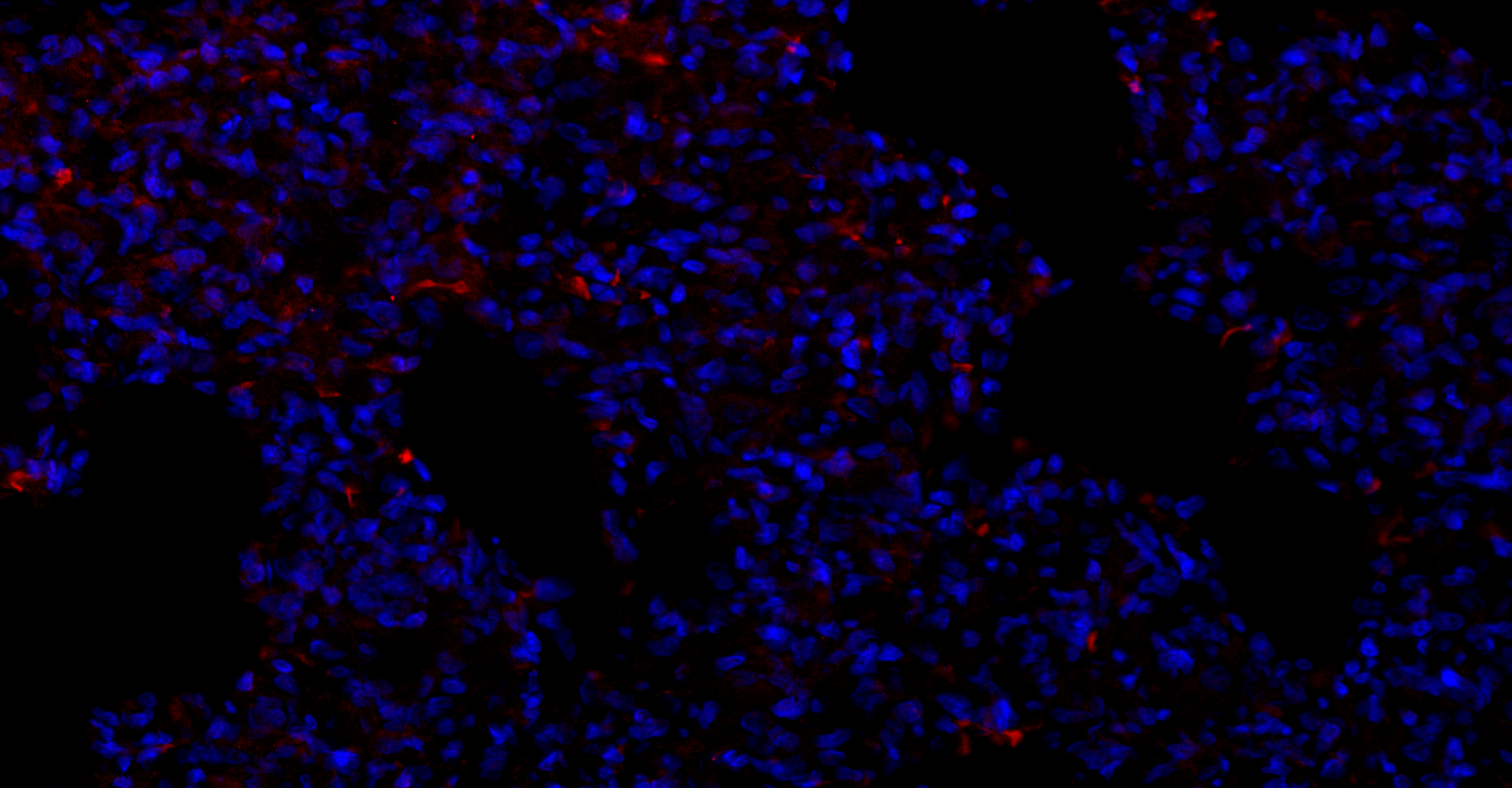

Supplement: Supplementary file 1 [file nutrients-17-02242-s001.zip › Figure S2 Original images/figure2-P-5 citH3 IF_40.0x.tif]

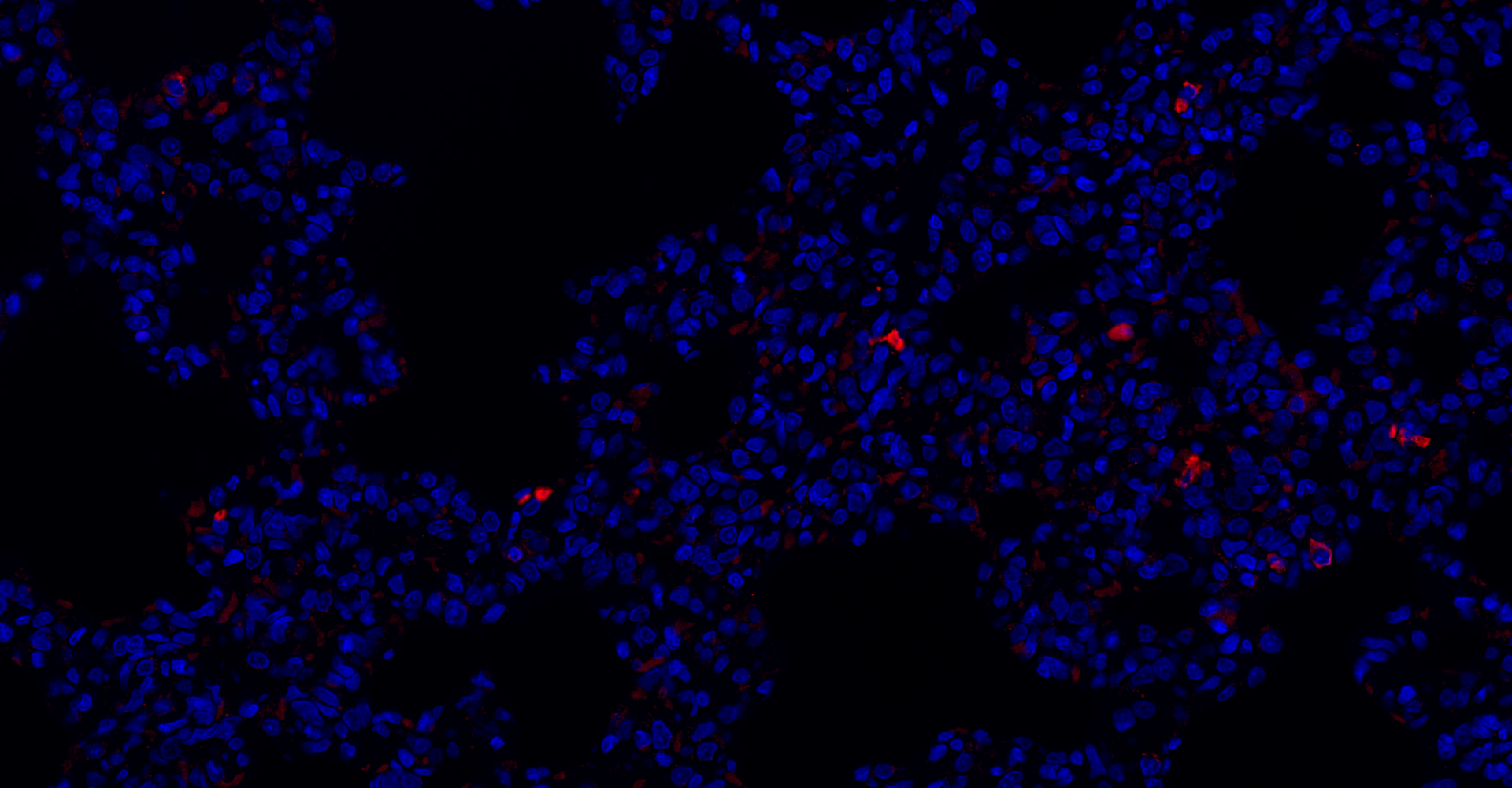

Supplement: Supplementary file 1 [file nutrients-17-02242-s001.zip › Figure S2 Original images/figure2-P-5 ly6g IF_40.0x.tif]

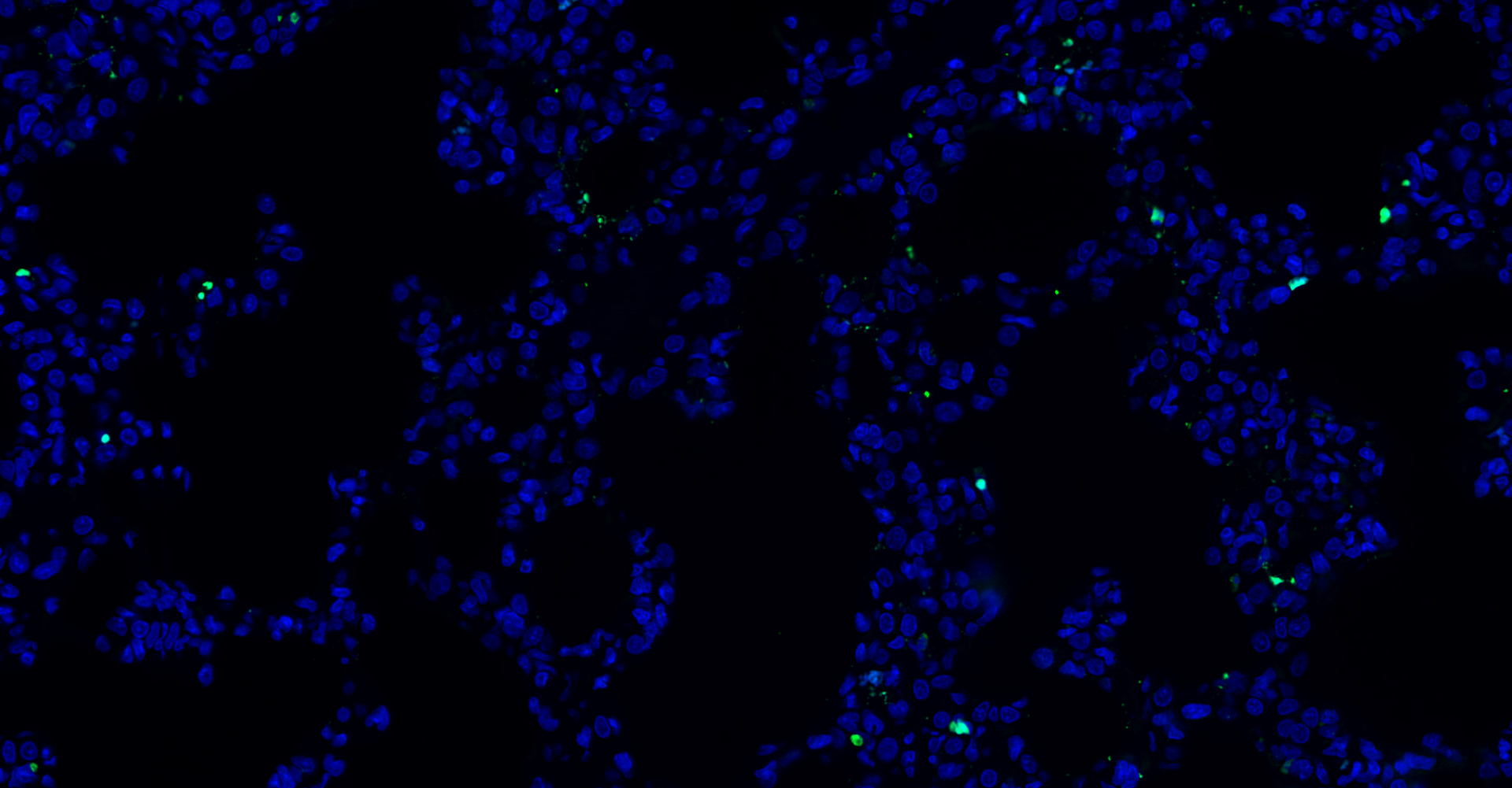

Supplement: Supplementary file 1 [file nutrients-17-02242-s001.zip › Figure S2 Original images/figure2-P-5 tunel IF_40.0x.tif]

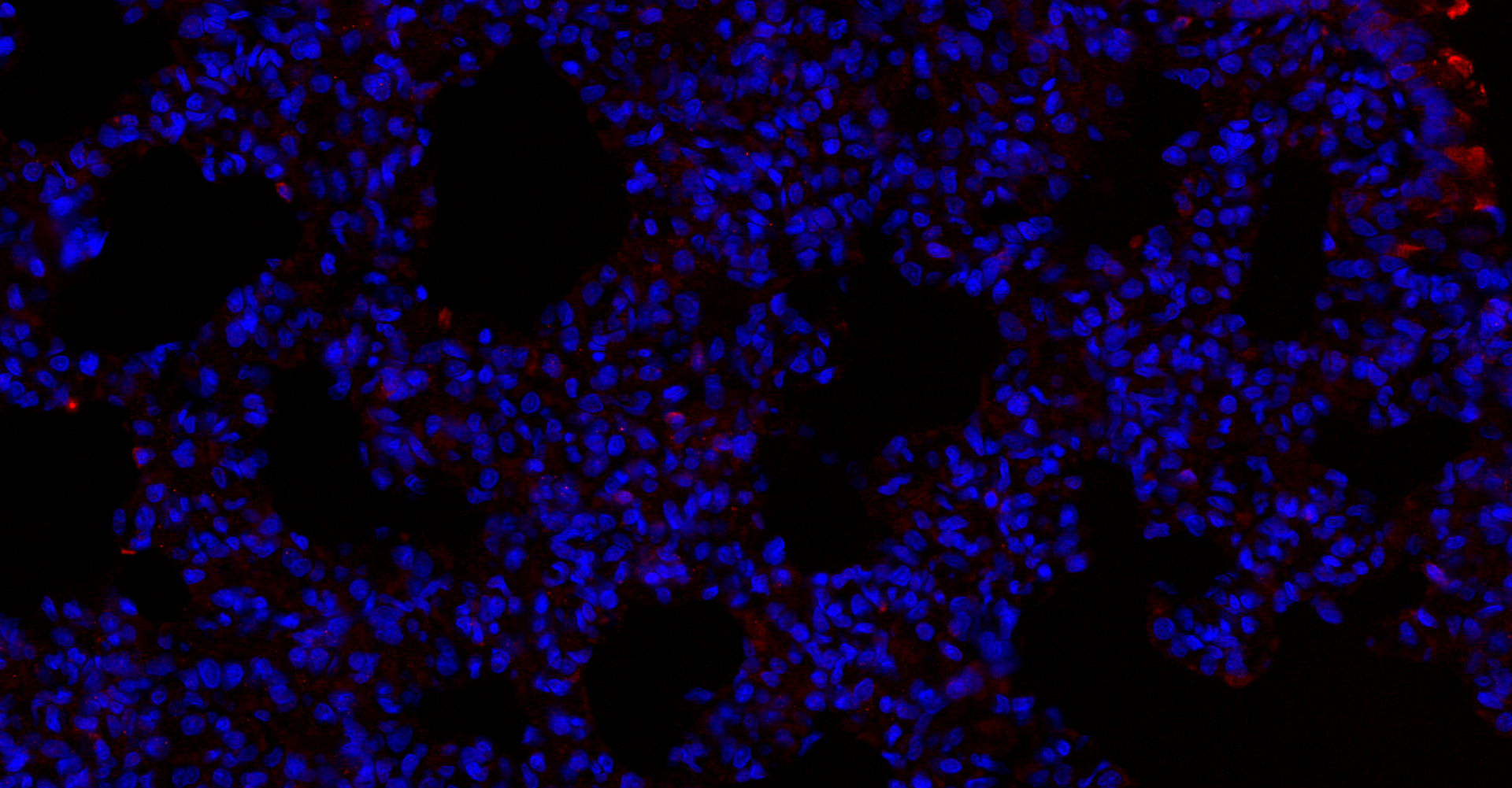

Supplement: Supplementary file 1 [file nutrients-17-02242-s001.zip › Figure S2 Original images/figure2-P-6 citH3 IF_40.0x.tif]

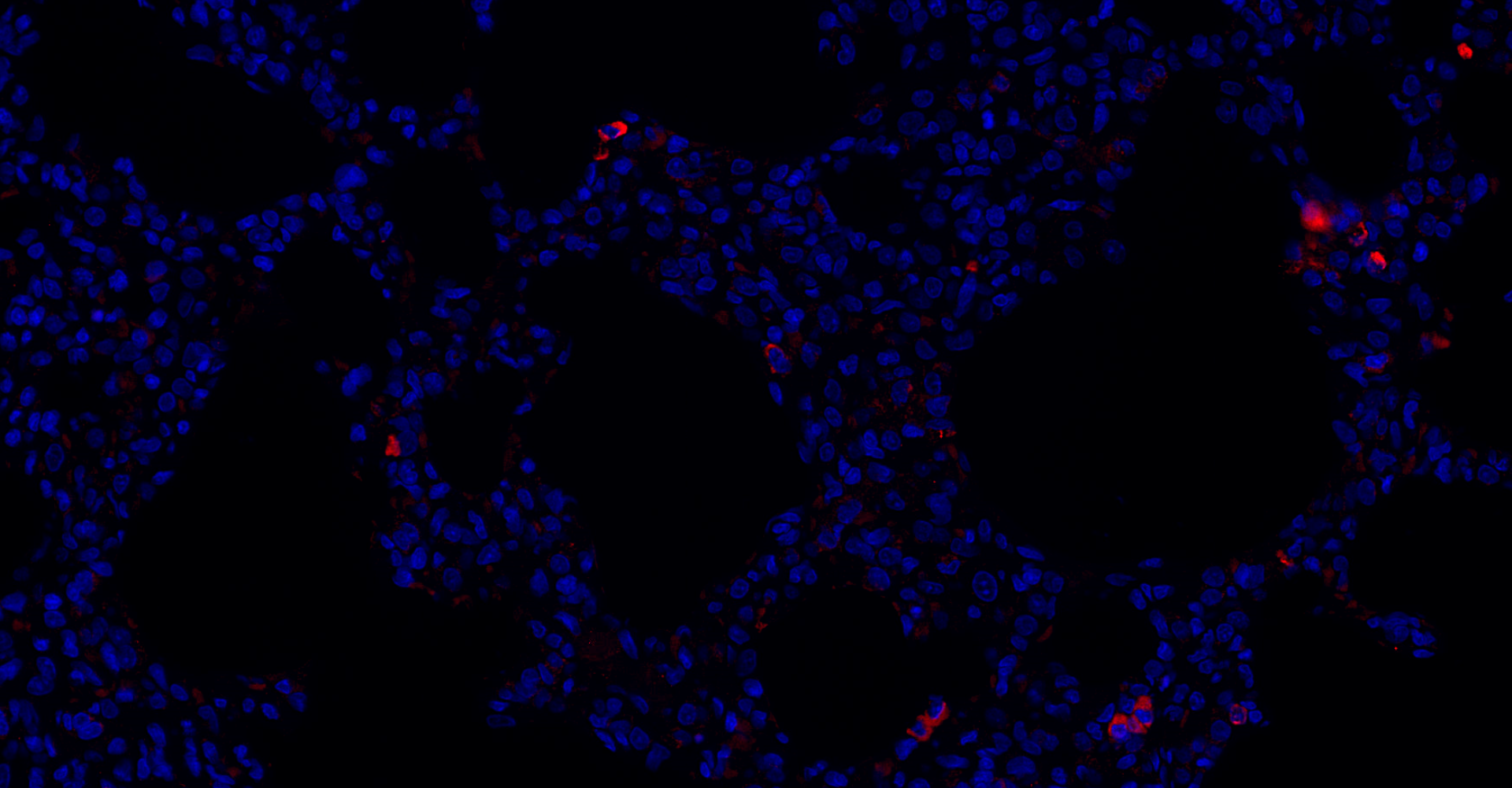

Supplement: Supplementary file 1 [file nutrients-17-02242-s001.zip › Figure S2 Original images/figure2-P-6 ly6g IF_40.0x.tif]

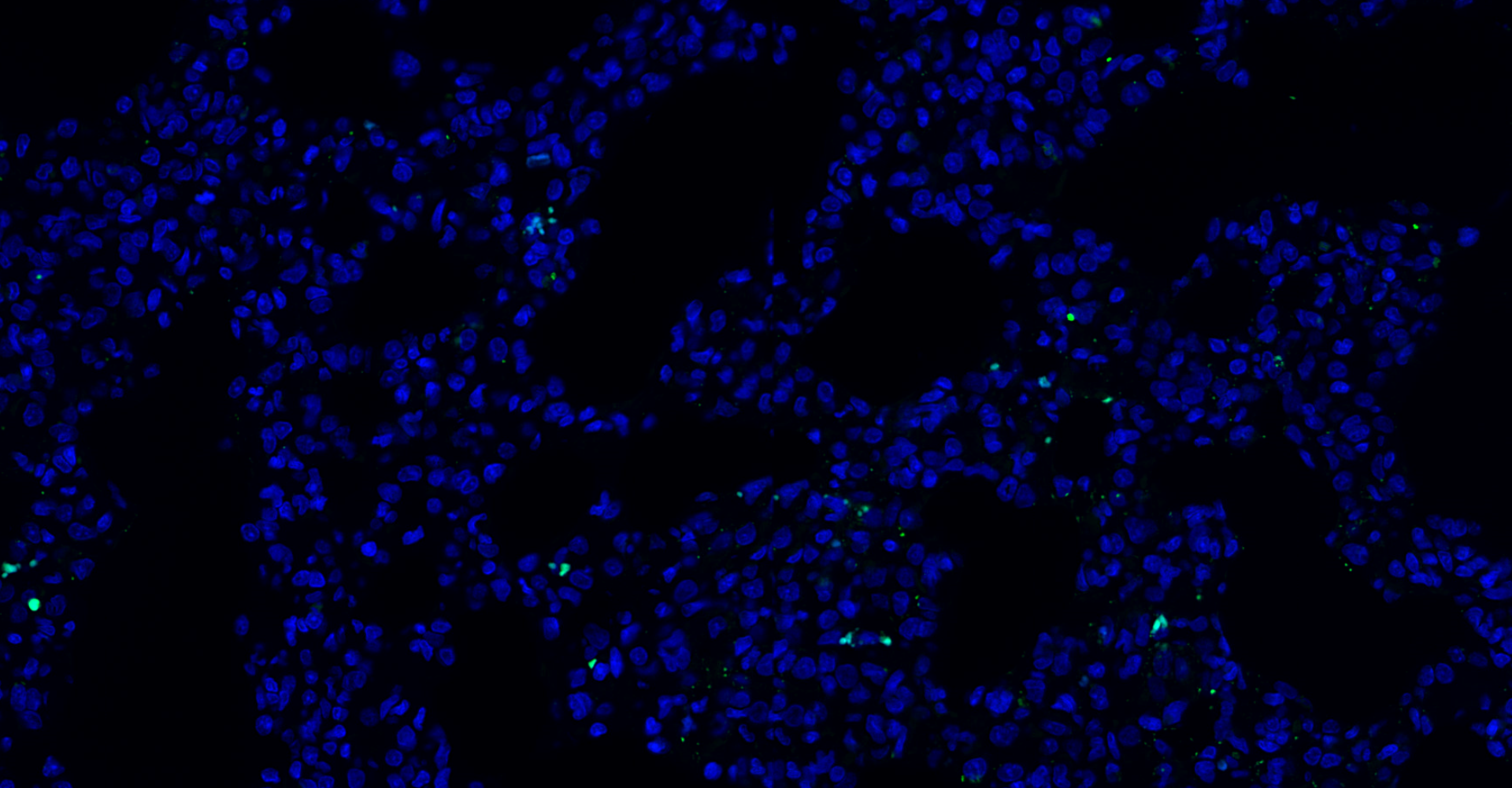

Supplement: Supplementary file 1 [file nutrients-17-02242-s001.zip › Figure S2 Original images/figure2-P-6 tunel IF_40.0x.tif]

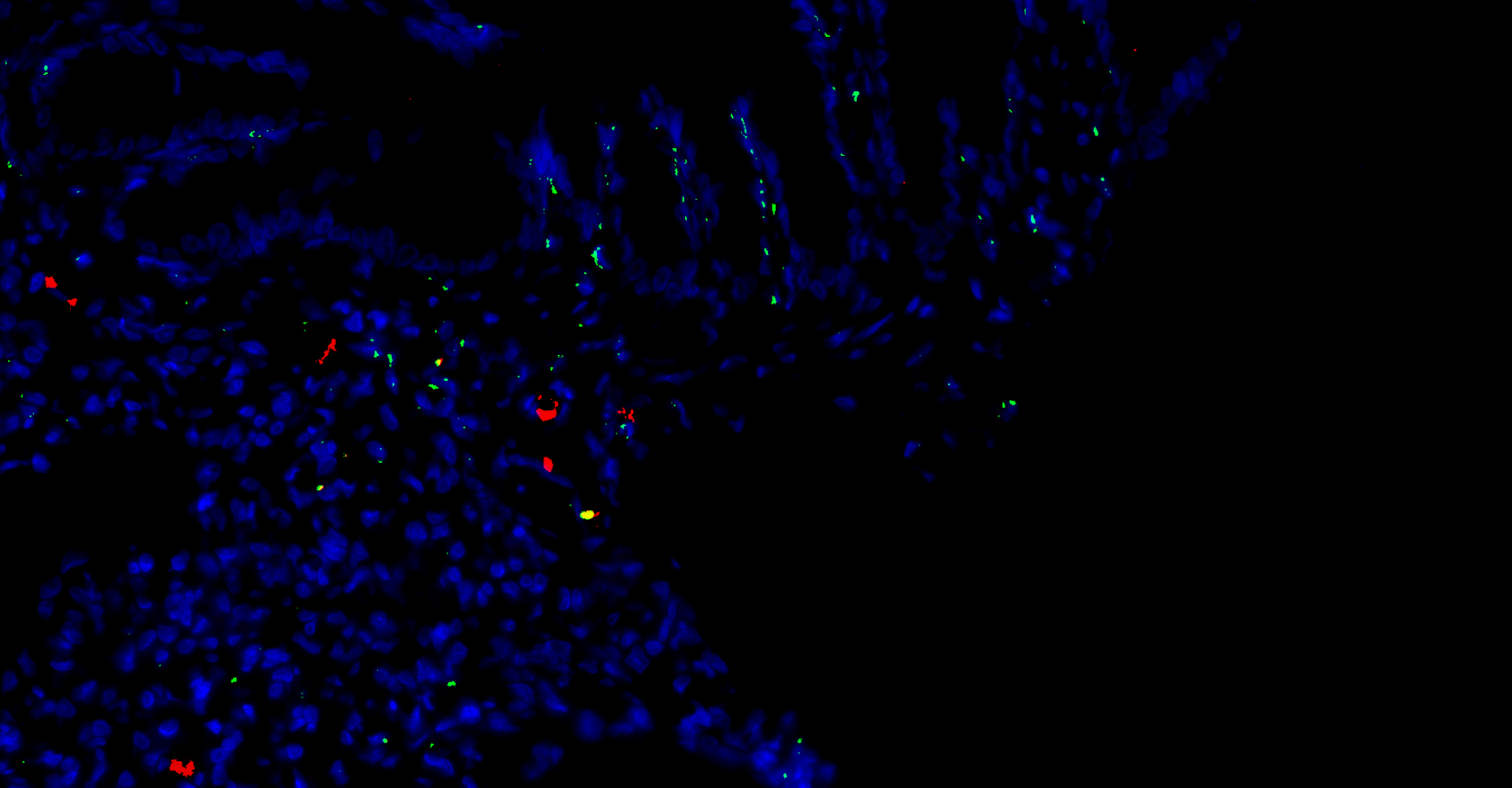

Supplement: Supplementary file 1 [file nutrients-17-02242-s001.zip › Figure S2 Original images/figure6-GP-1 LY6G-ACH4_40.0x.tif]

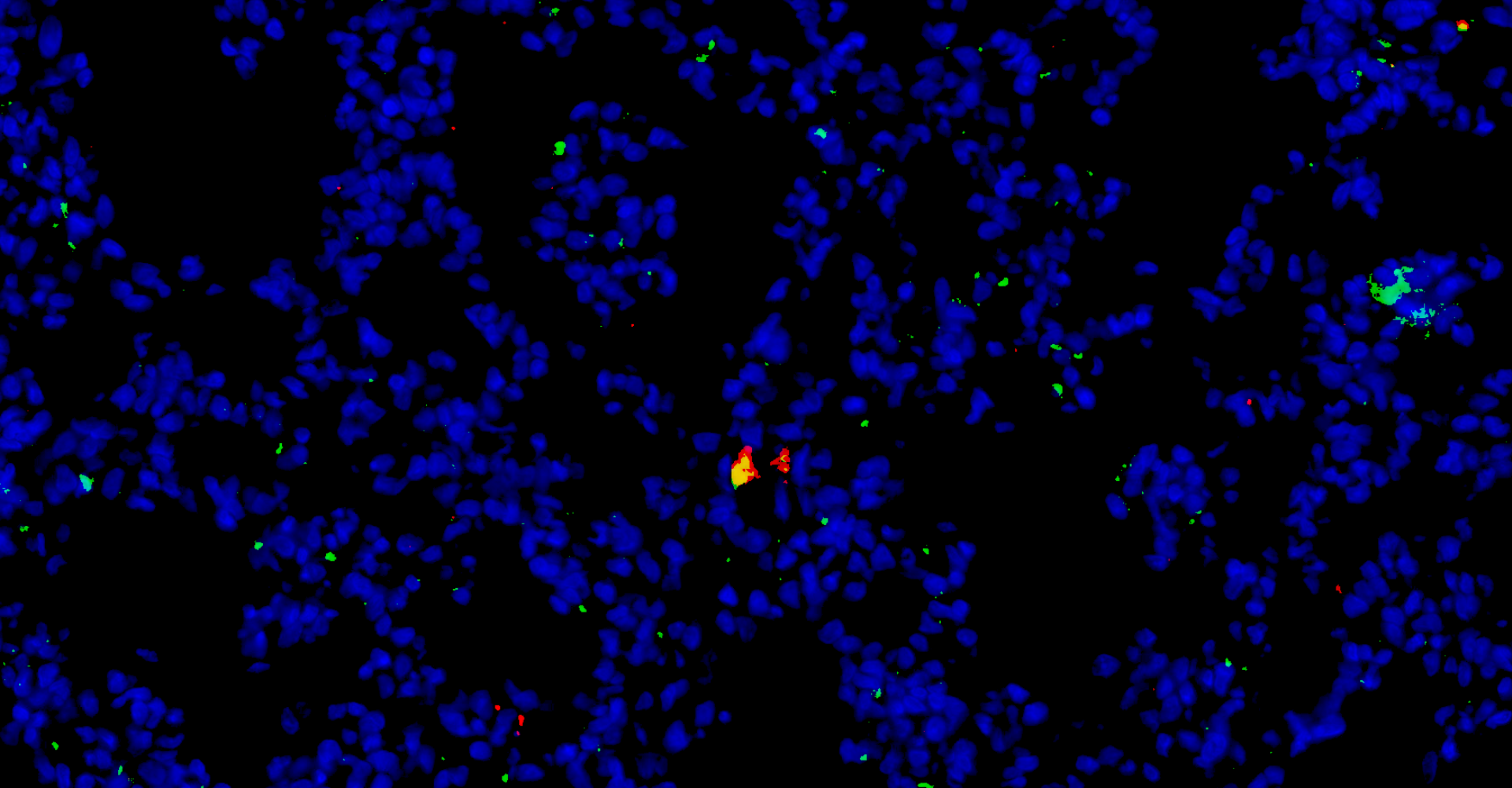

Supplement: Supplementary file 1 [file nutrients-17-02242-s001.zip › Figure S2 Original images/figure6-GP-1 LY6G-CITH3_40.0x.tif]

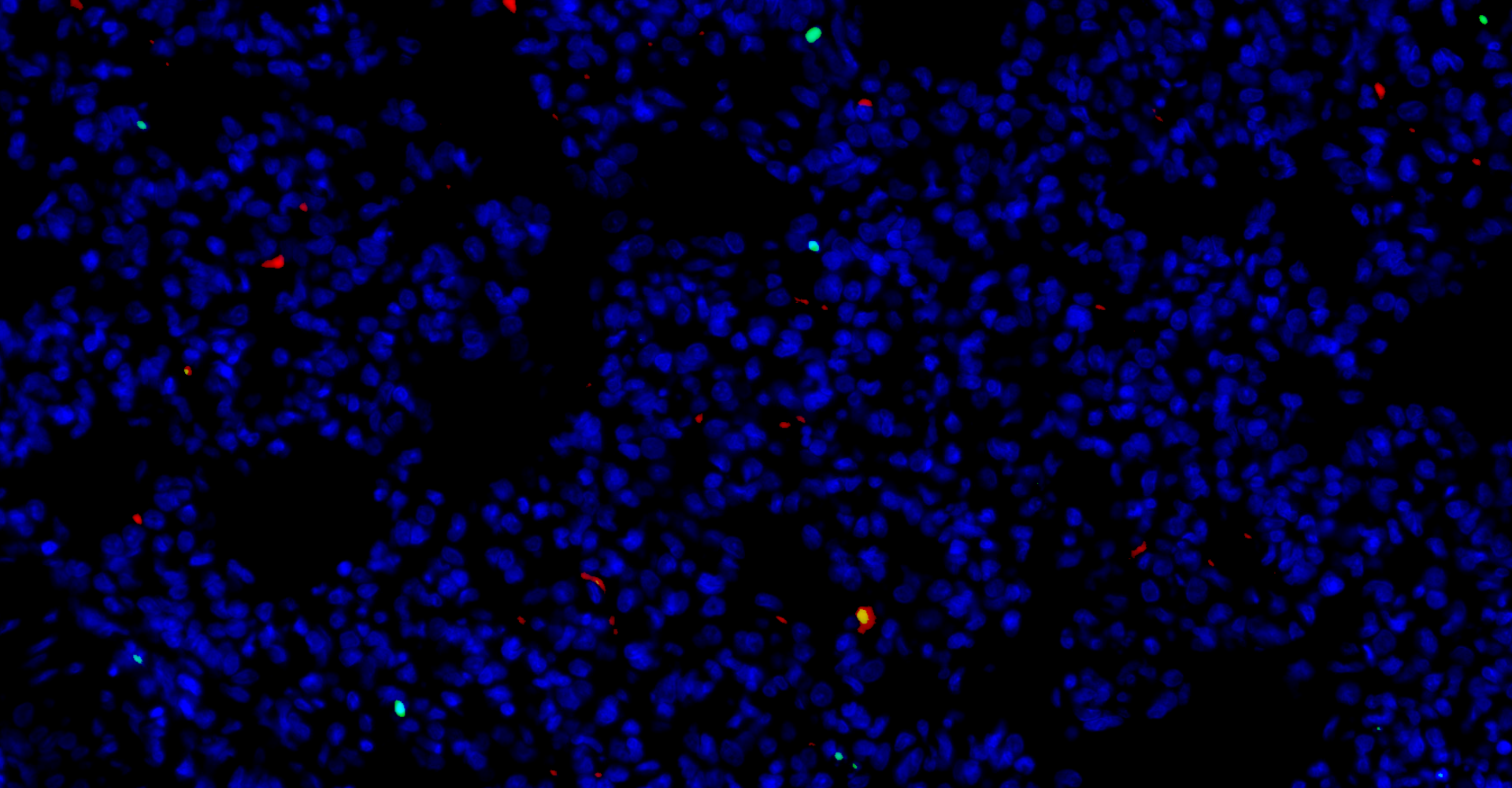

Supplement: Supplementary file 1 [file nutrients-17-02242-s001.zip › Figure S2 Original images/figure6-GP-1 tunel-IY6G_40.0x.tif]

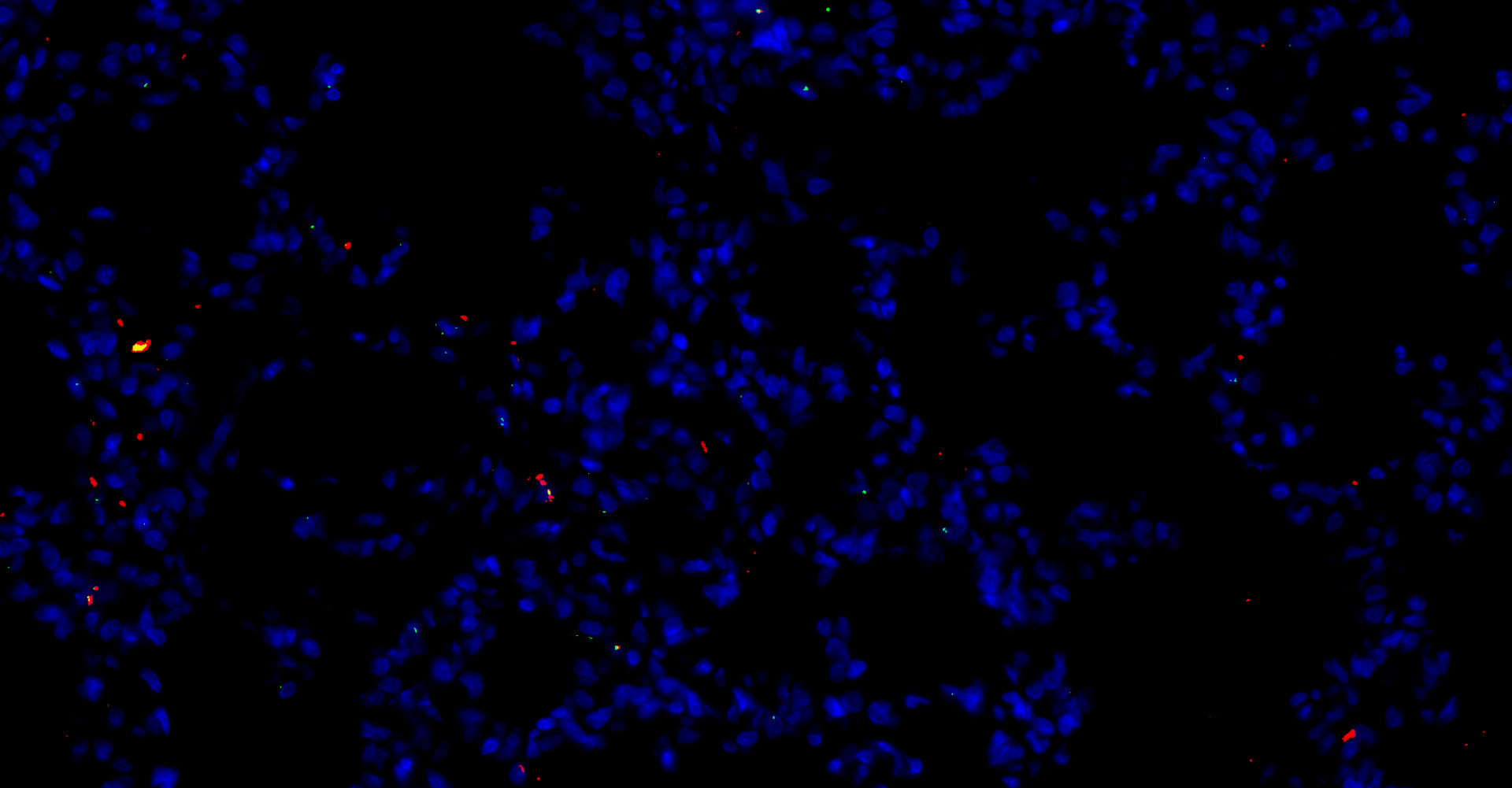

Supplement: Supplementary file 1 [file nutrients-17-02242-s001.zip › Figure S2 Original images/figure6-GP-2 LY6G-ACH4_40.0x.tif]

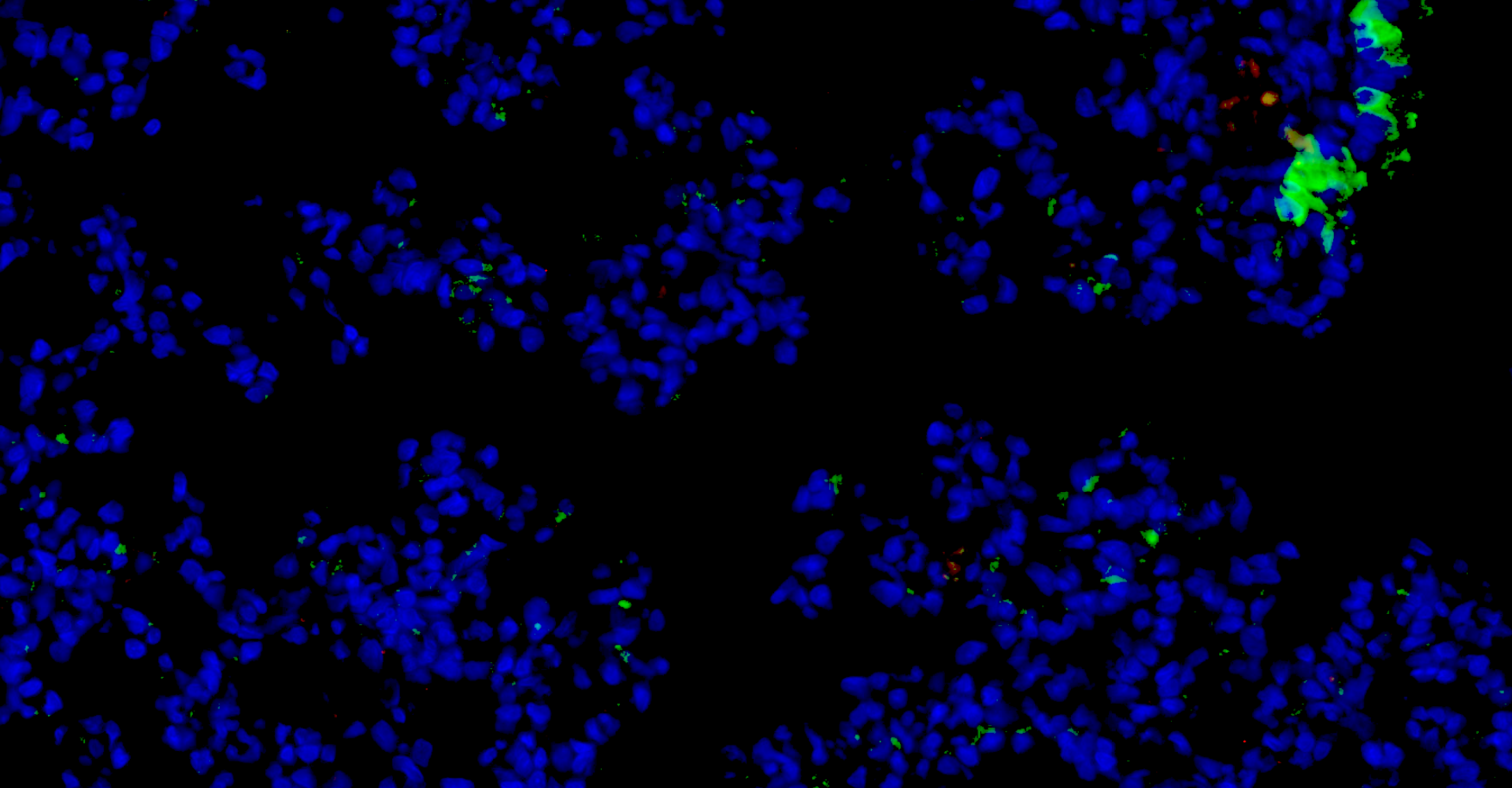

Supplement: Supplementary file 1 [file nutrients-17-02242-s001.zip › Figure S2 Original images/figure6-GP-2 LY6G-CITH3_40.0x.tif]

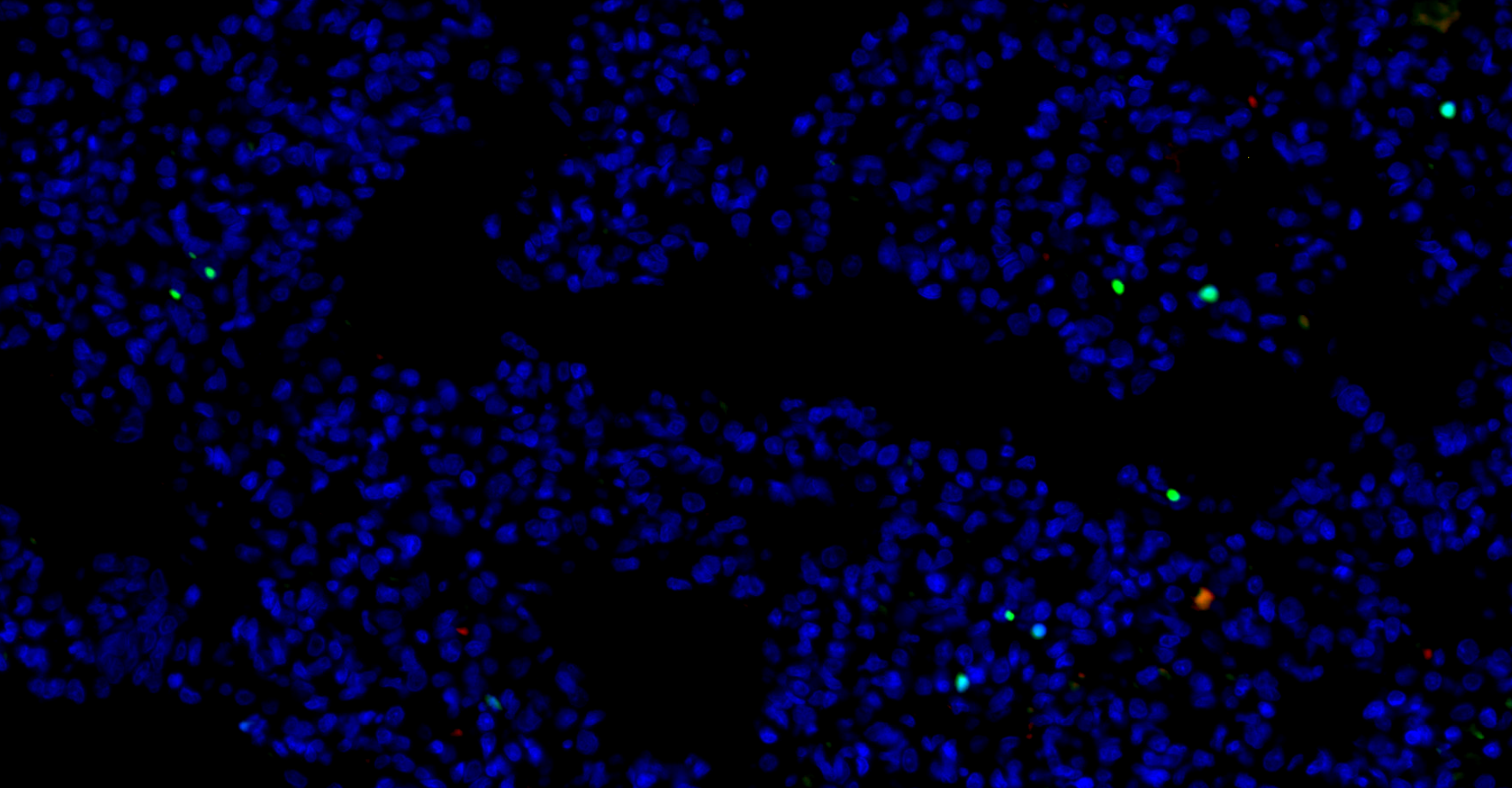

Supplement: Supplementary file 1 [file nutrients-17-02242-s001.zip › Figure S2 Original images/figure6-GP-2 tunel-IY6G_40.0x.tif]

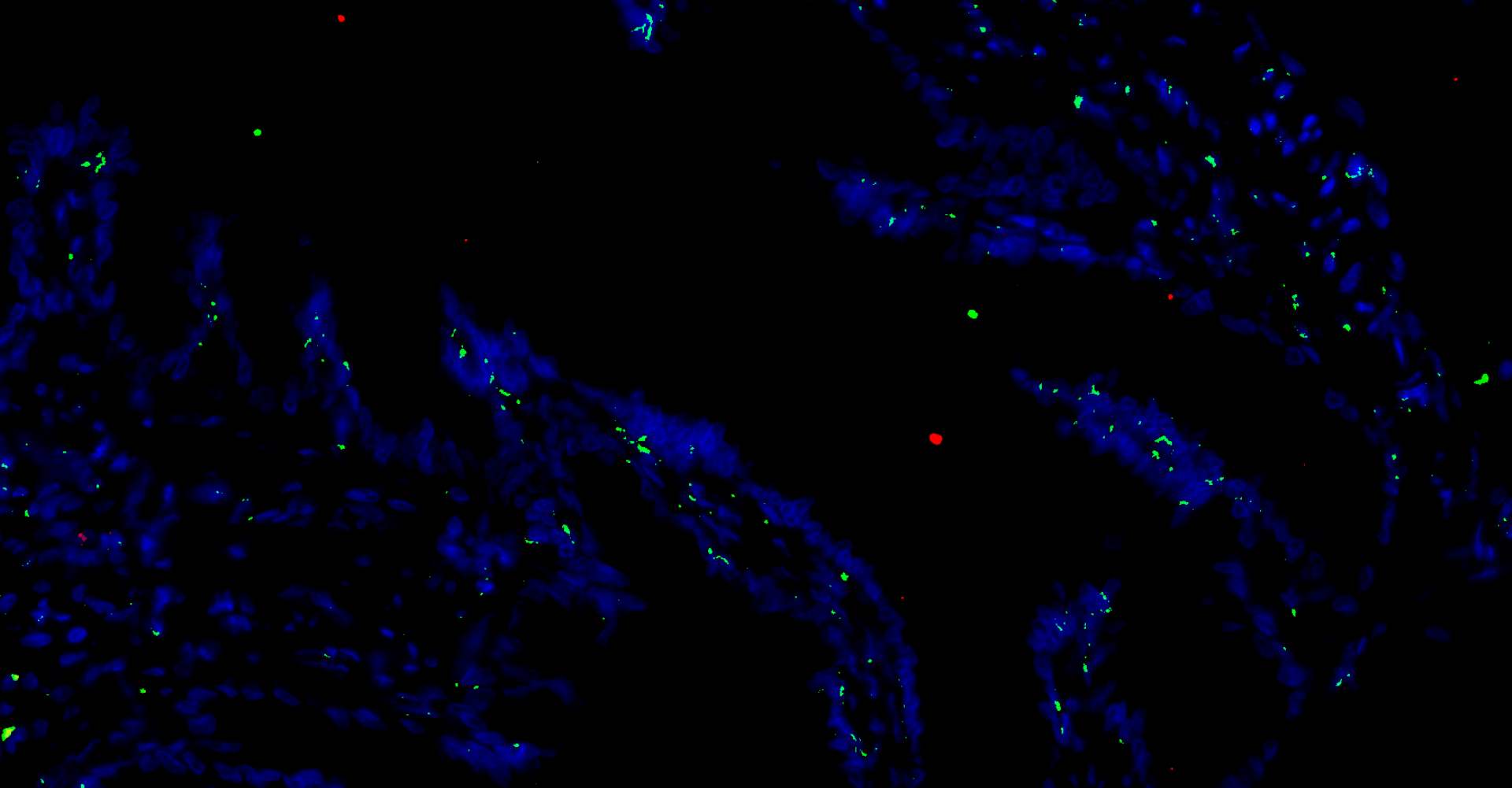

Supplement: Supplementary file 1 [file nutrients-17-02242-s001.zip › Figure S2 Original images/figure6-GP-3 LY6G-ACH4_40.0x.tif]

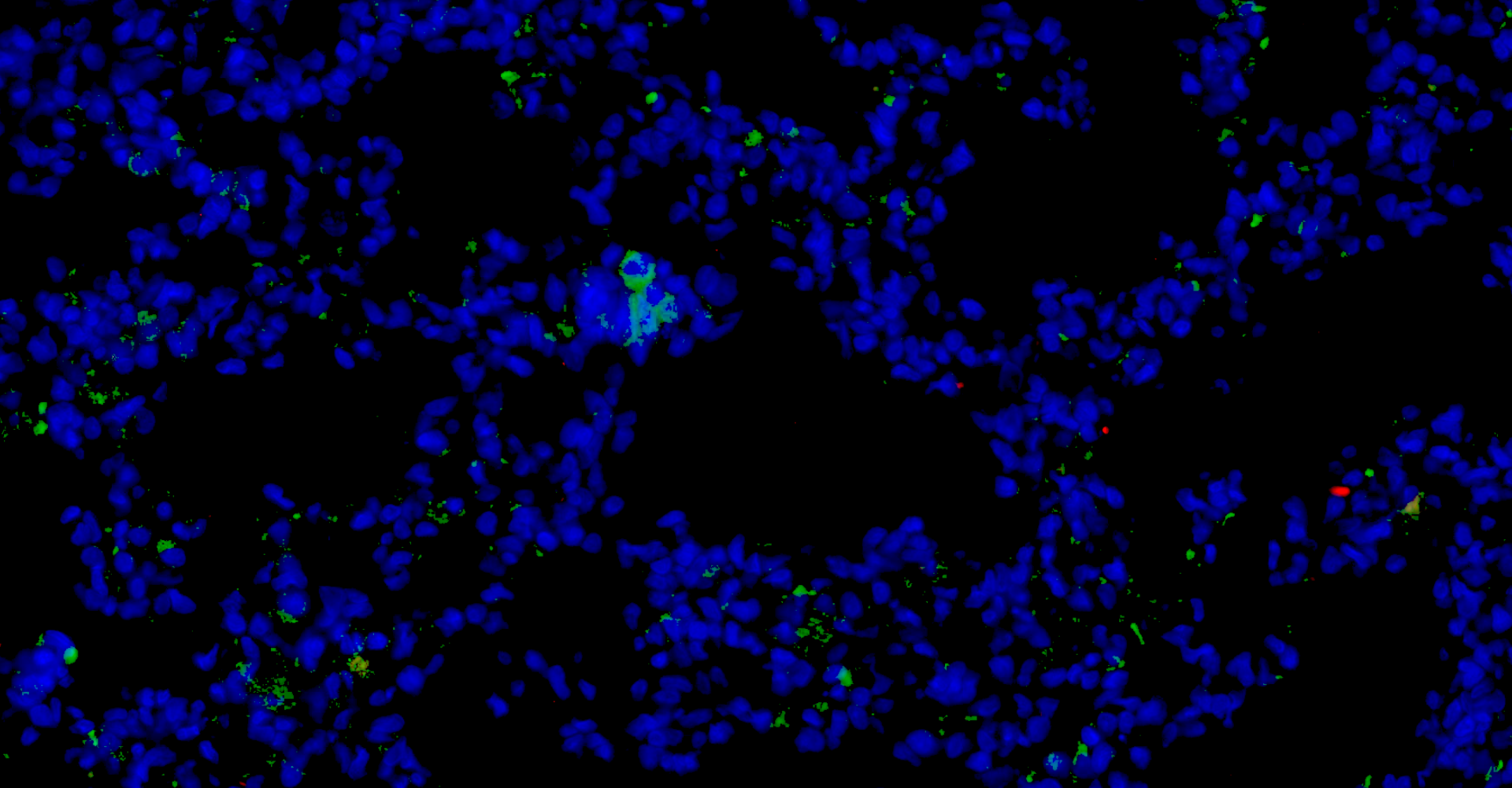

Supplement: Supplementary file 1 [file nutrients-17-02242-s001.zip › Figure S2 Original images/figure6-GP-3 LY6G-CITH3_40.0x.tif]

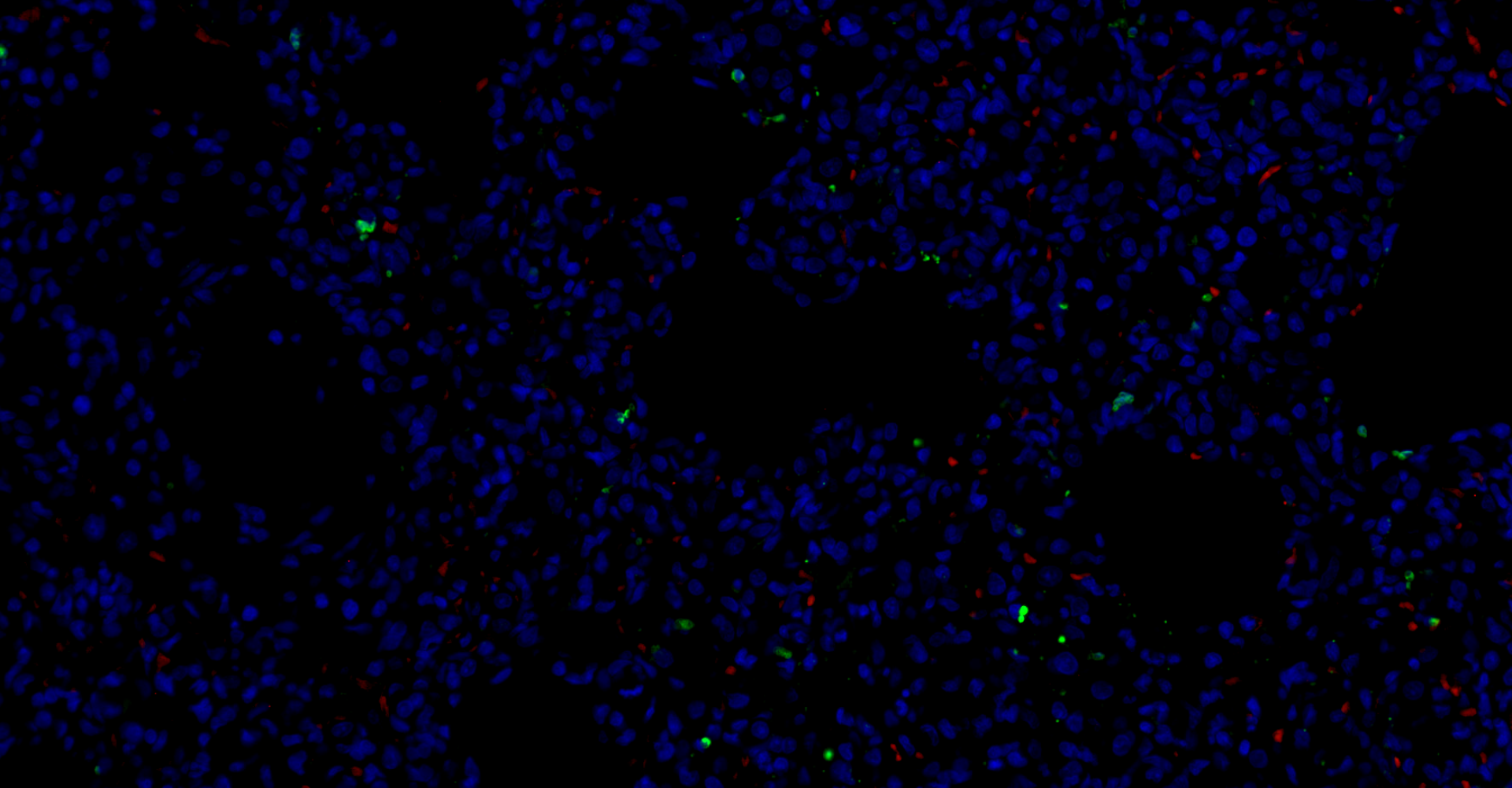

Supplement: Supplementary file 1 [file nutrients-17-02242-s001.zip › Figure S2 Original images/figure6-GP-3 tunel-IY6G_40.0x.tif]

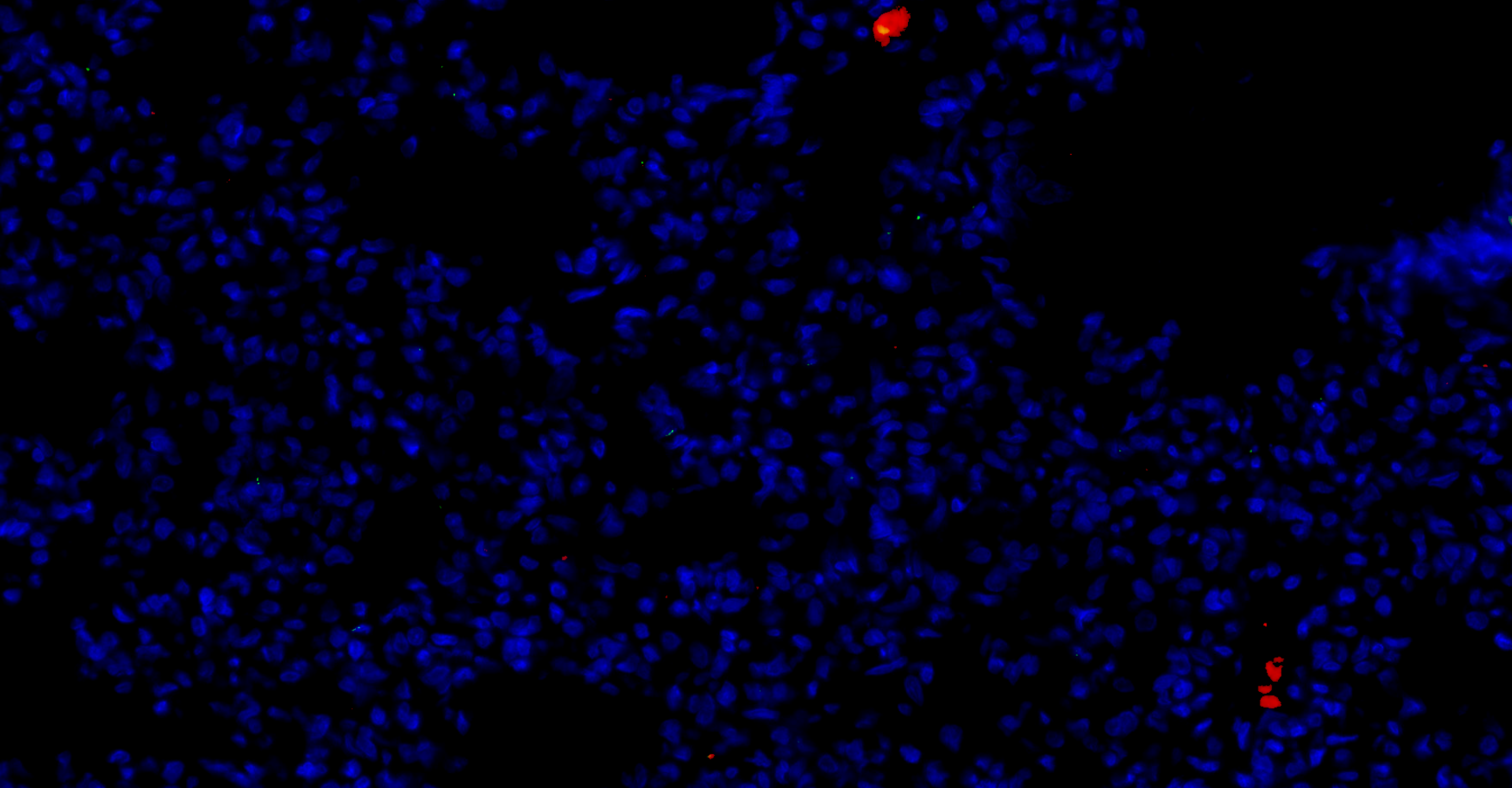

Supplement: Supplementary file 1 [file nutrients-17-02242-s001.zip › Figure S2 Original images/figure6-GP-4 LY6G-ACH4_40.0x.tif]

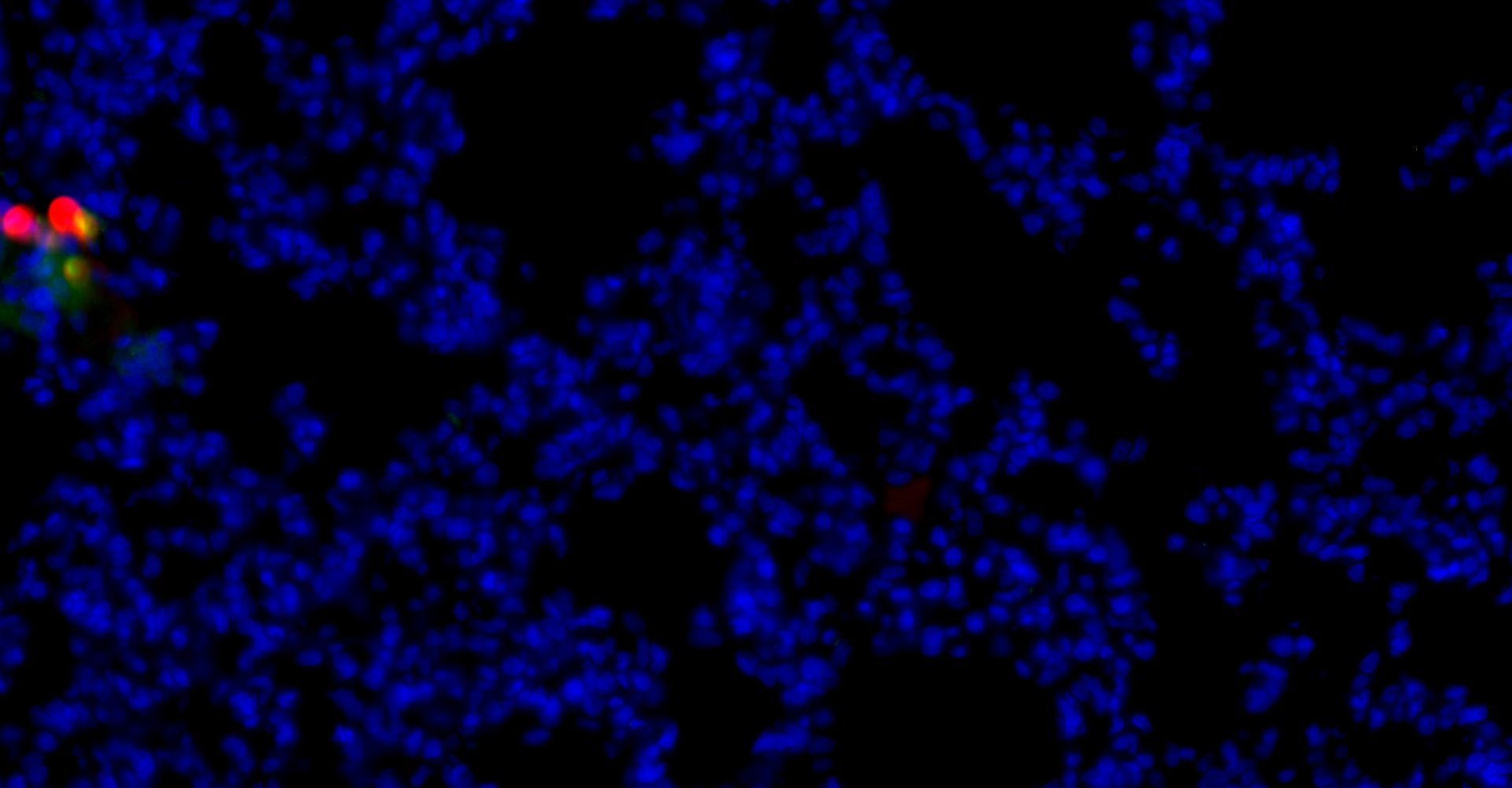

Supplement: Supplementary file 1 [file nutrients-17-02242-s001.zip › Figure S2 Original images/figure6-GP-4 LY6G-CITH3_40.0x.tif]

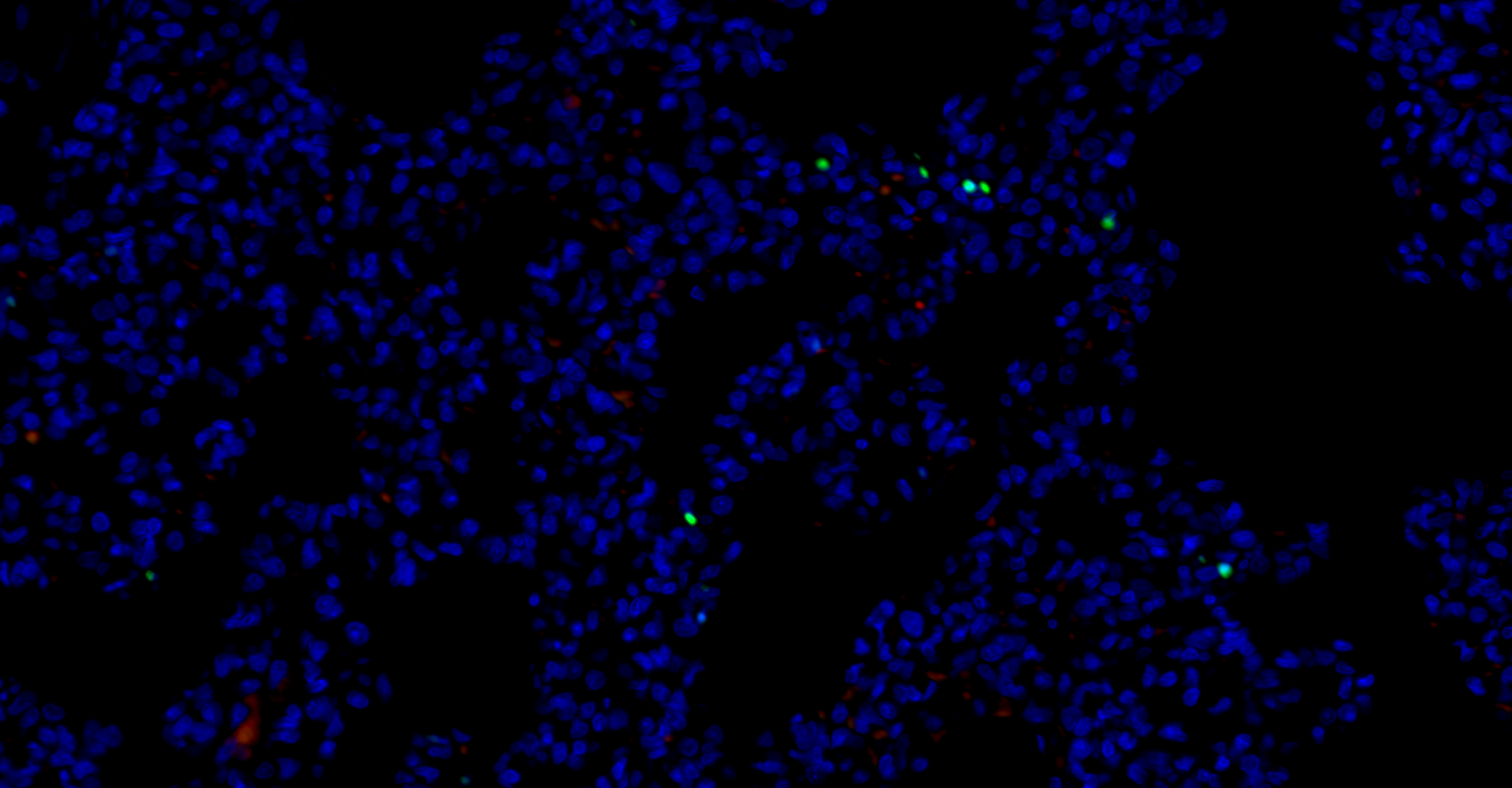

Supplement: Supplementary file 1 [file nutrients-17-02242-s001.zip › Figure S2 Original images/figure6-GP-4 tunel-IY6G_40.0x.tif]

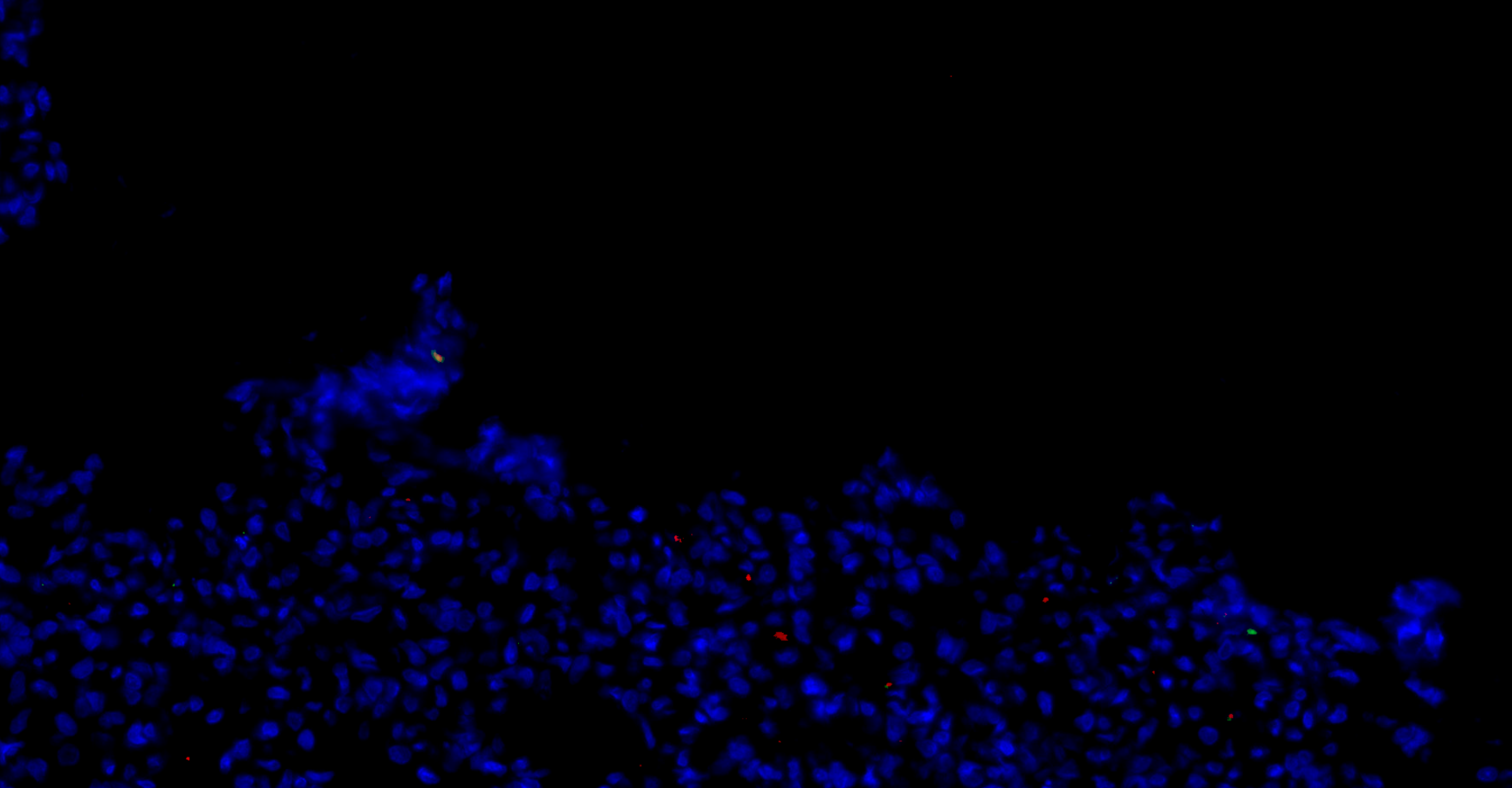

Supplement: Supplementary file 1 [file nutrients-17-02242-s001.zip › Figure S2 Original images/figure6-GP-5 LY6G-ACH4_40.0x.tif]

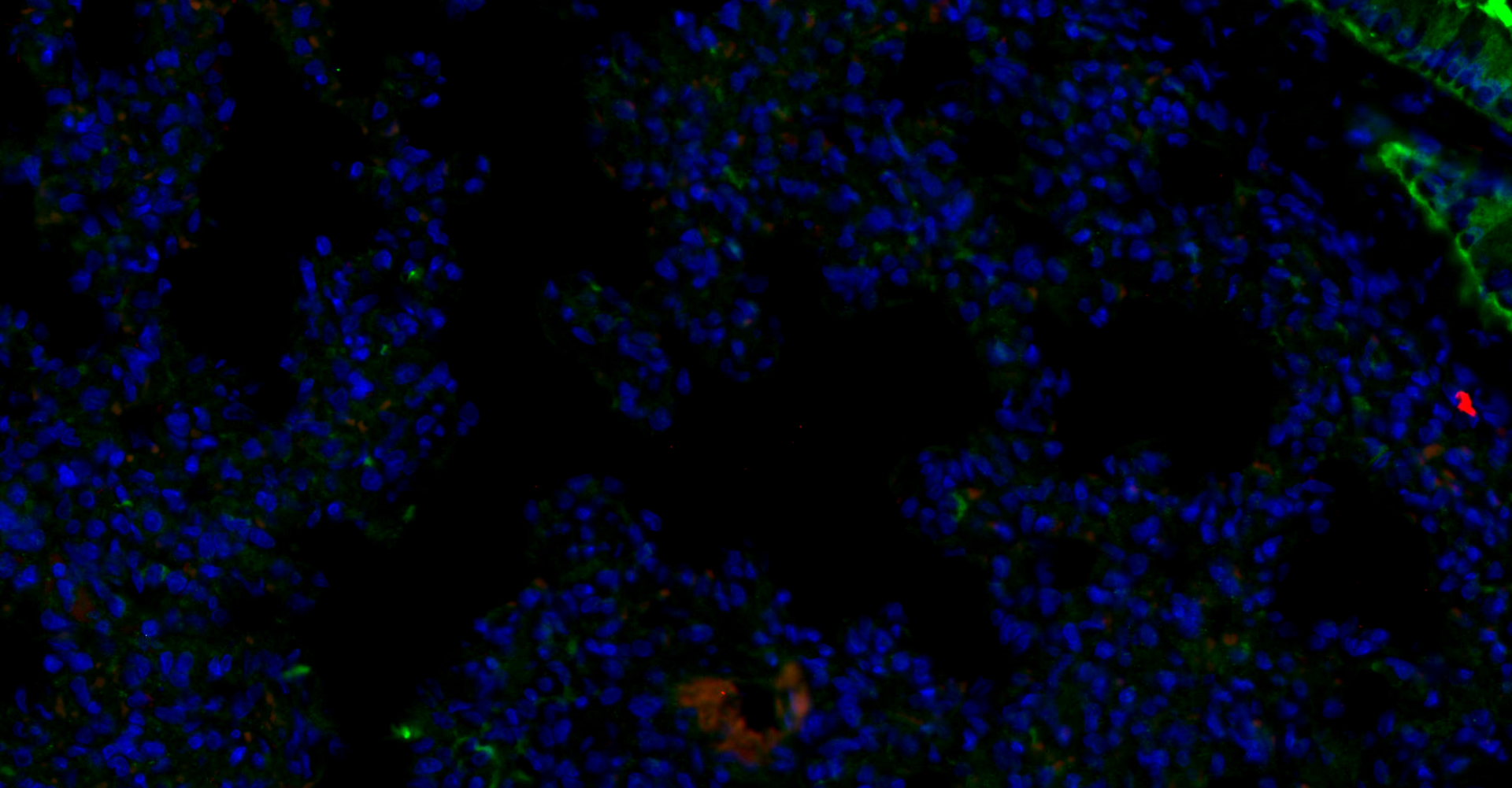

Supplement: Supplementary file 1 [file nutrients-17-02242-s001.zip › Figure S2 Original images/figure6-GP-5 LY6G-CITH3_40.0x.tif]

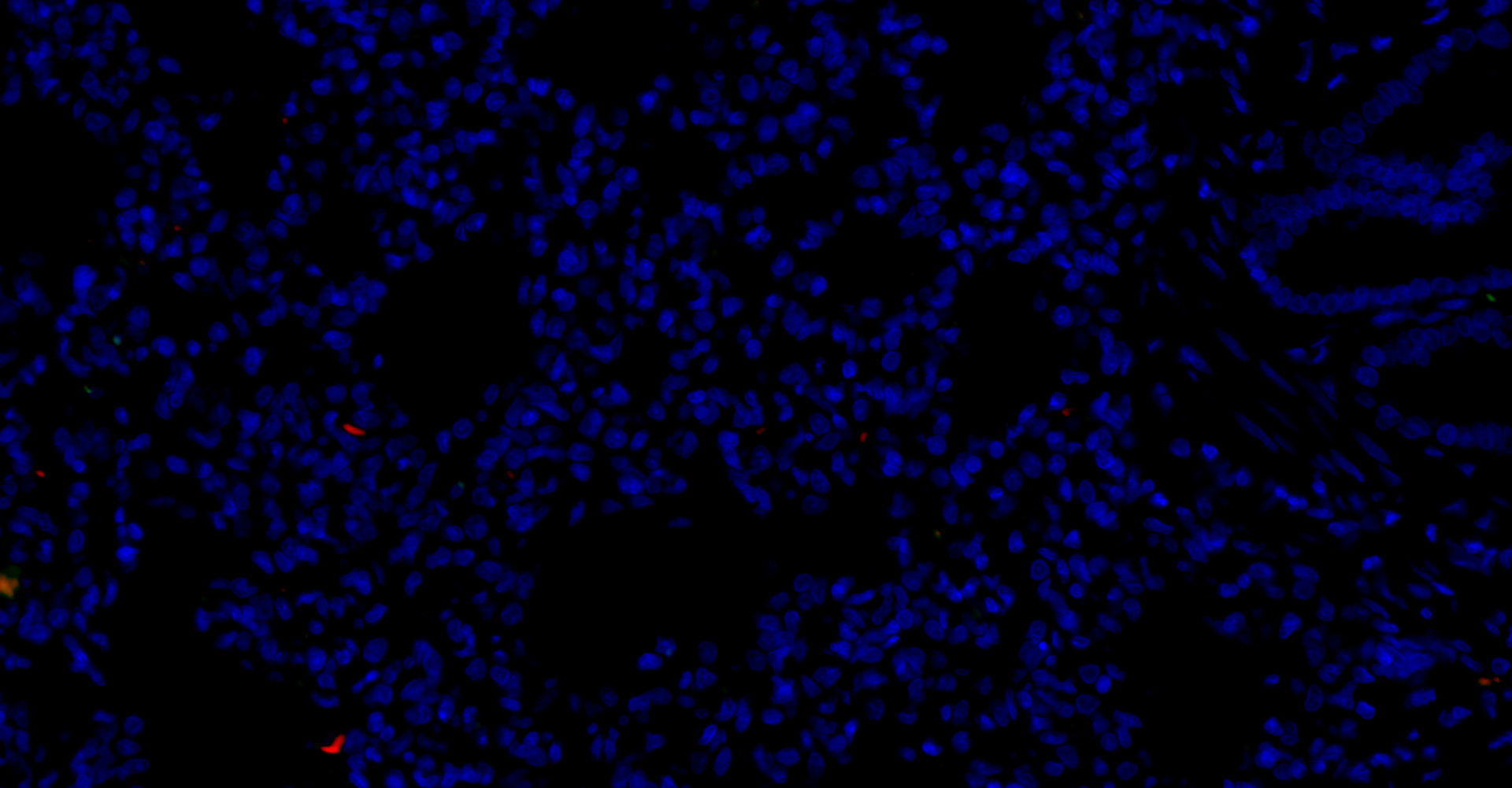

Supplement: Supplementary file 1 [file nutrients-17-02242-s001.zip › Figure S2 Original images/figure6-GP-5 tunel-IY6G_40.0x.tif]

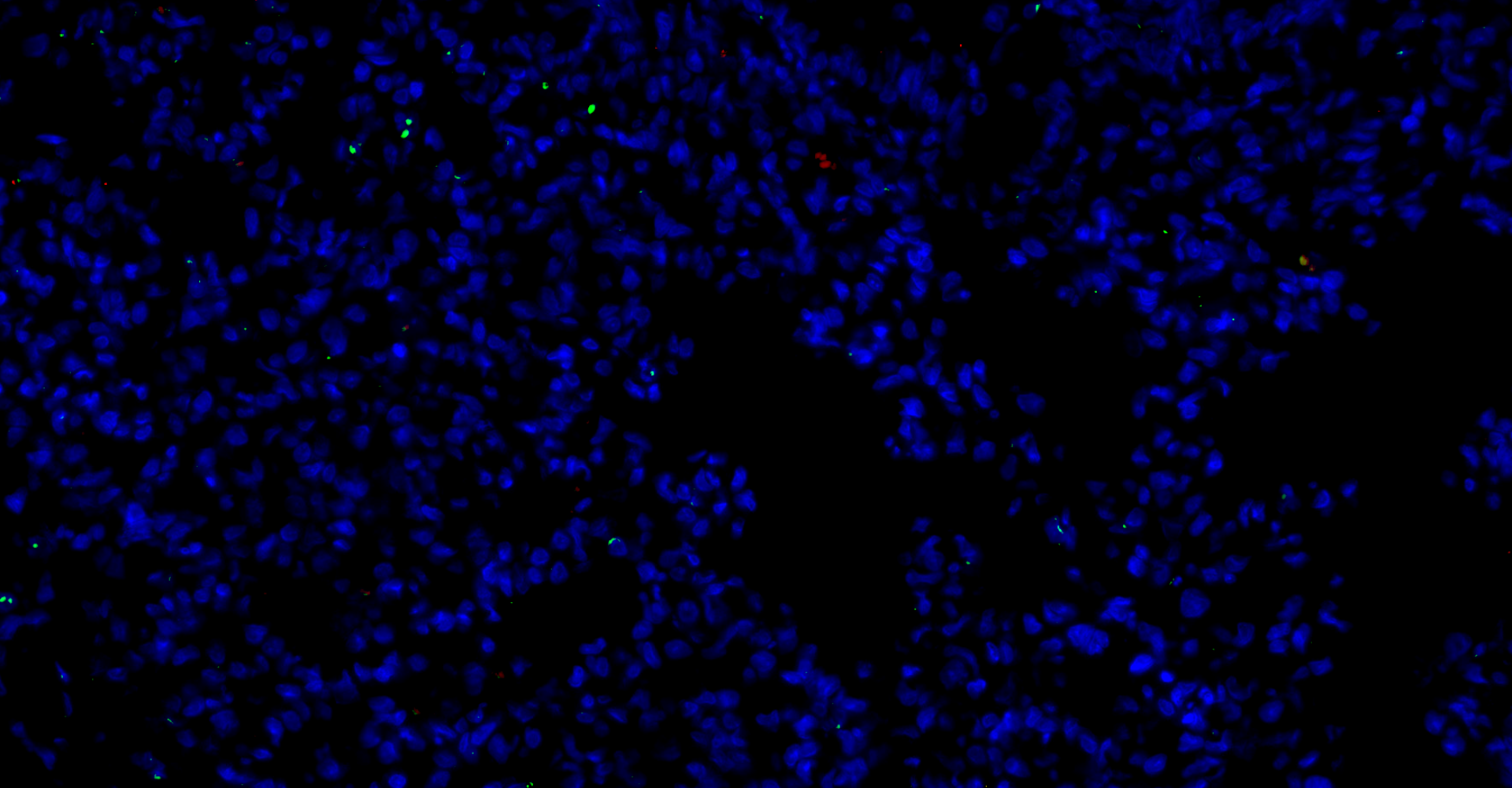

Supplement: Supplementary file 1 [file nutrients-17-02242-s001.zip › Figure S2 Original images/figure6-GP-6 LY6G-ACH4_40.0x.tif]

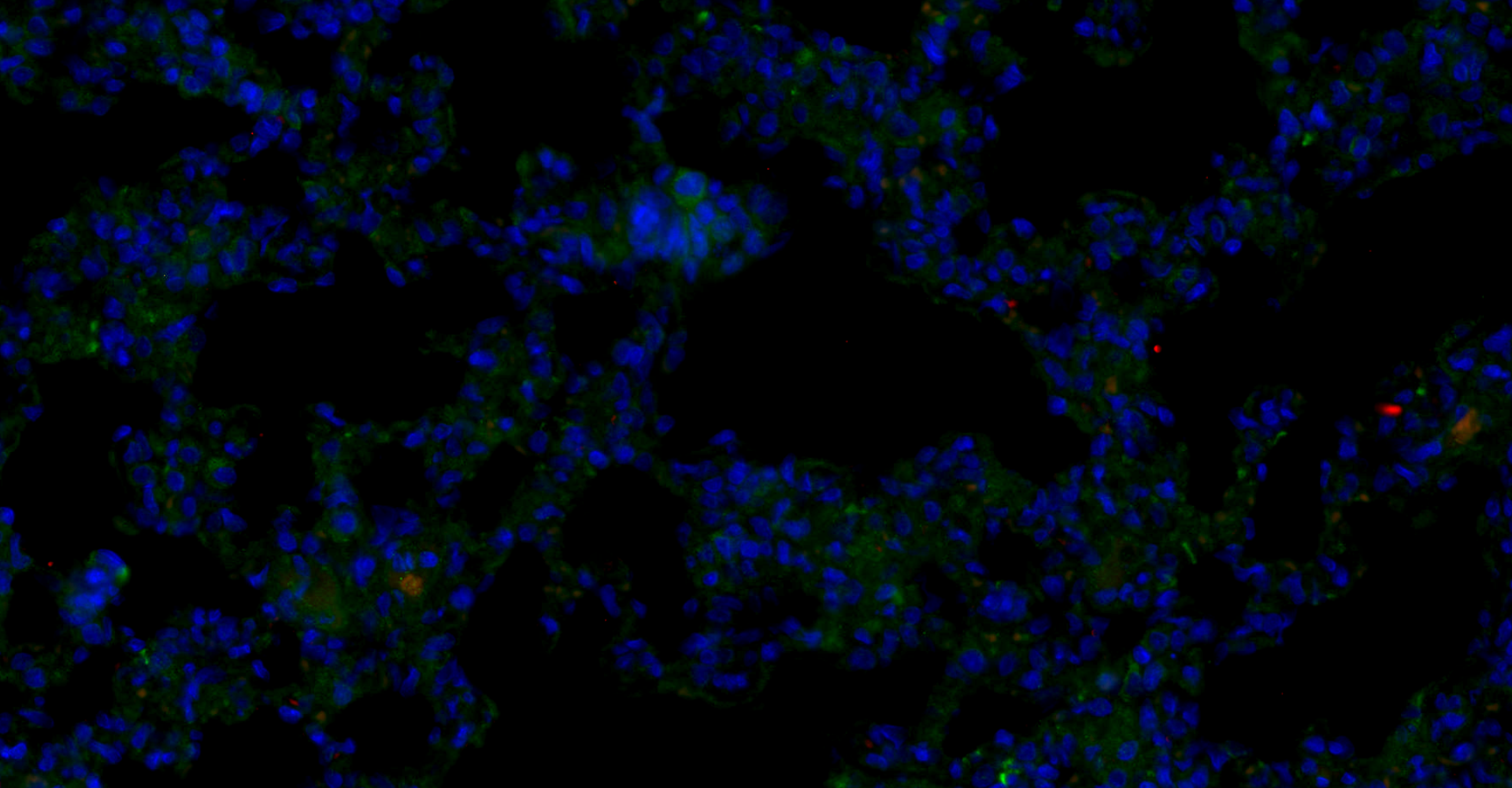

Supplement: Supplementary file 1 [file nutrients-17-02242-s001.zip › Figure S2 Original images/figure6-GP-6 LY6G-CITH3_40.0x.tif]

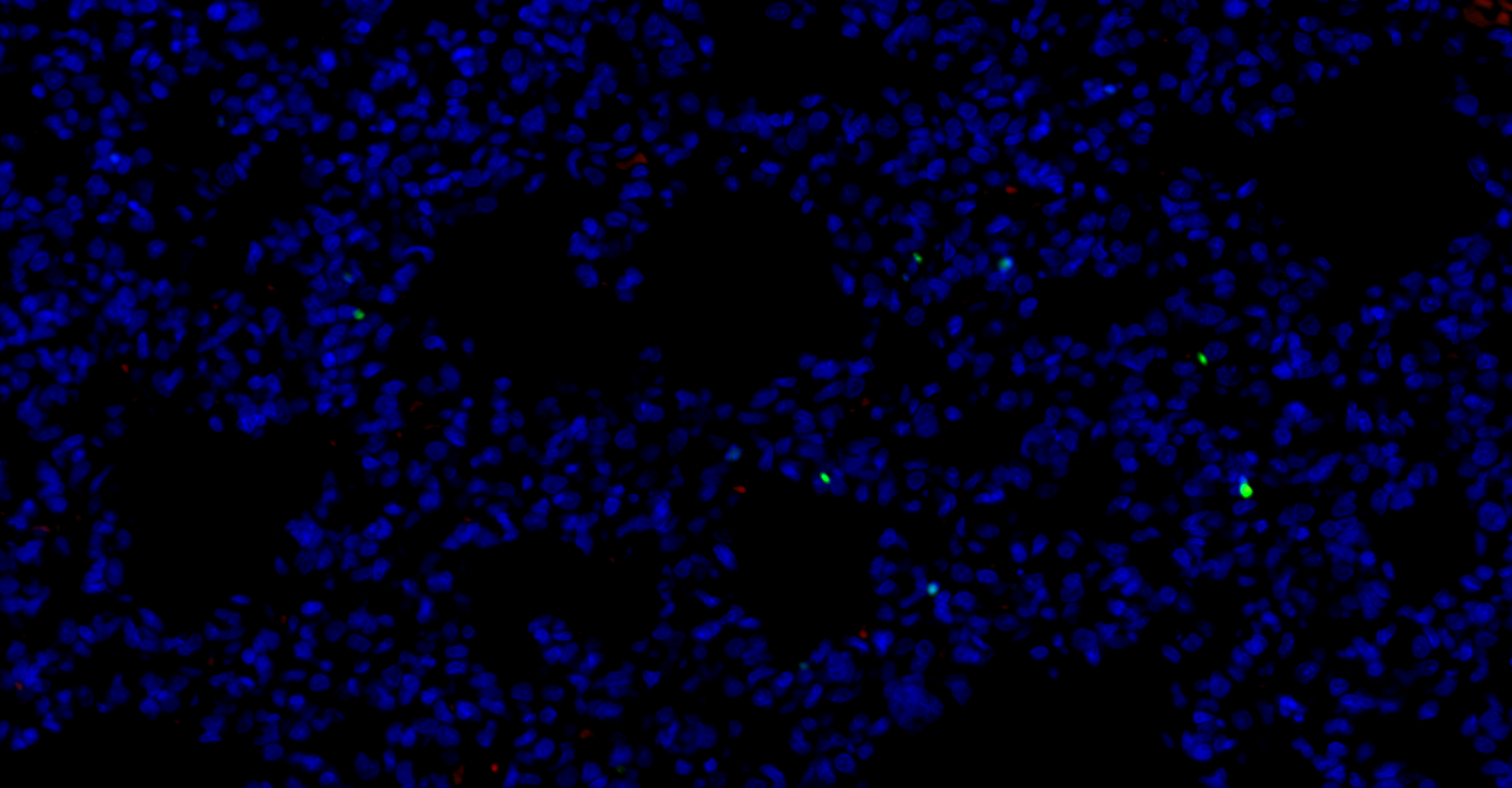

Supplement: Supplementary file 1 [file nutrients-17-02242-s001.zip › Figure S2 Original images/figure6-GP-6 tunel-IY6G_40.0x.tif]

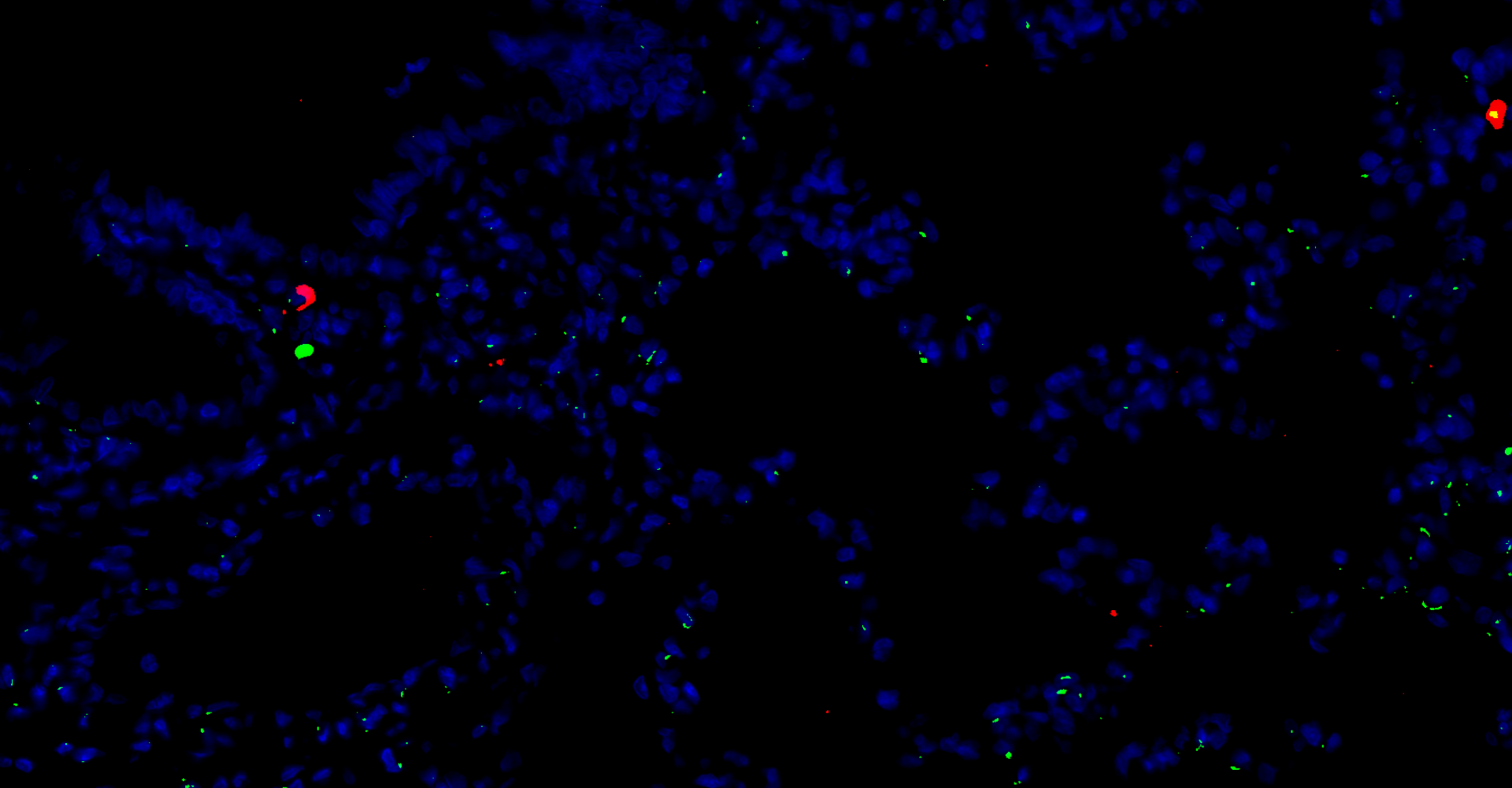

Supplement: Supplementary file 1 [file nutrients-17-02242-s001.zip › Figure S2 Original images/figure6-N-1 LY6G-ACH4_40.0x.tif]

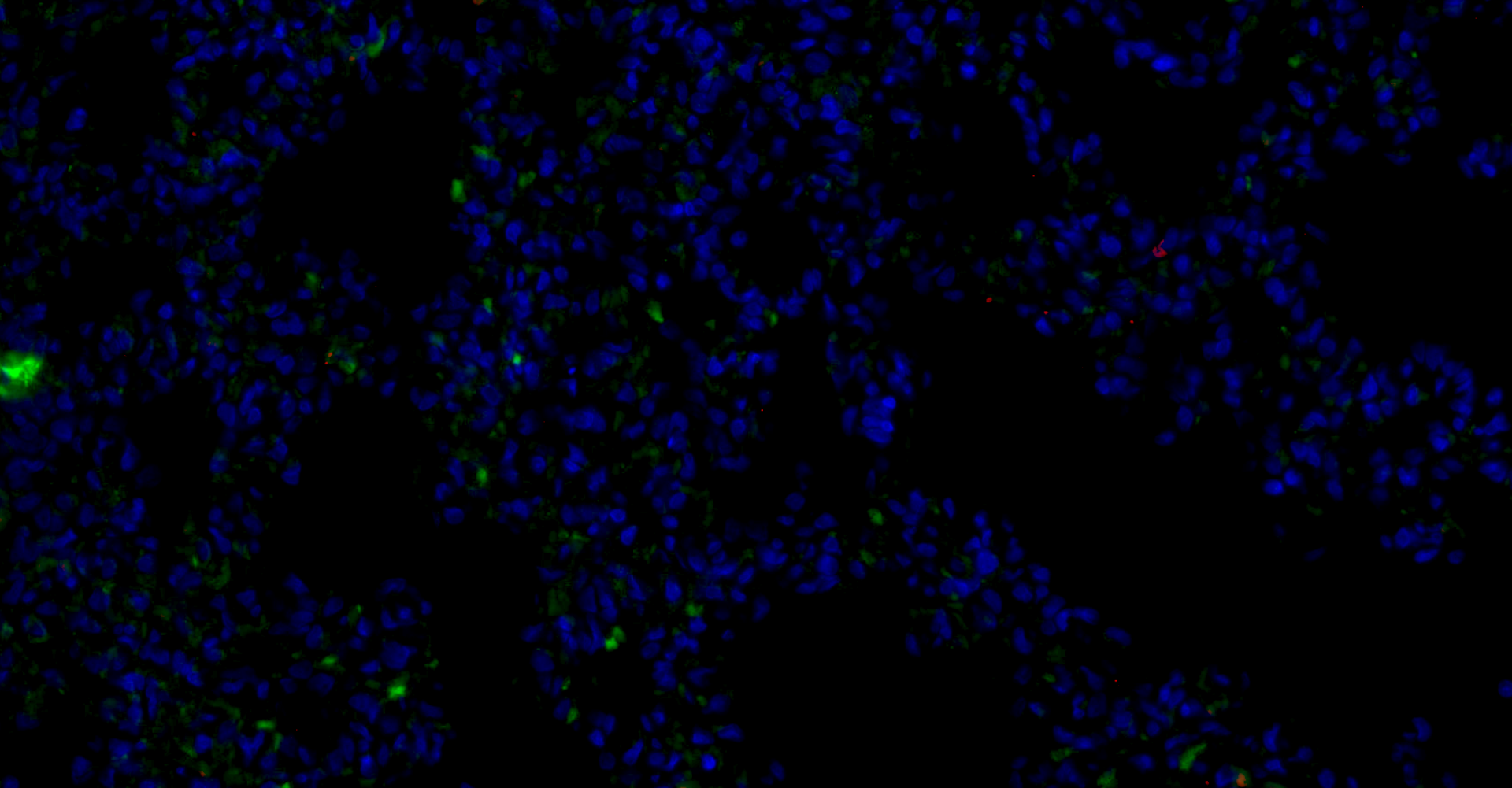

Supplement: Supplementary file 1 [file nutrients-17-02242-s001.zip › Figure S2 Original images/figure6-N-1 LY6G-CITH3_40.0x.tif]

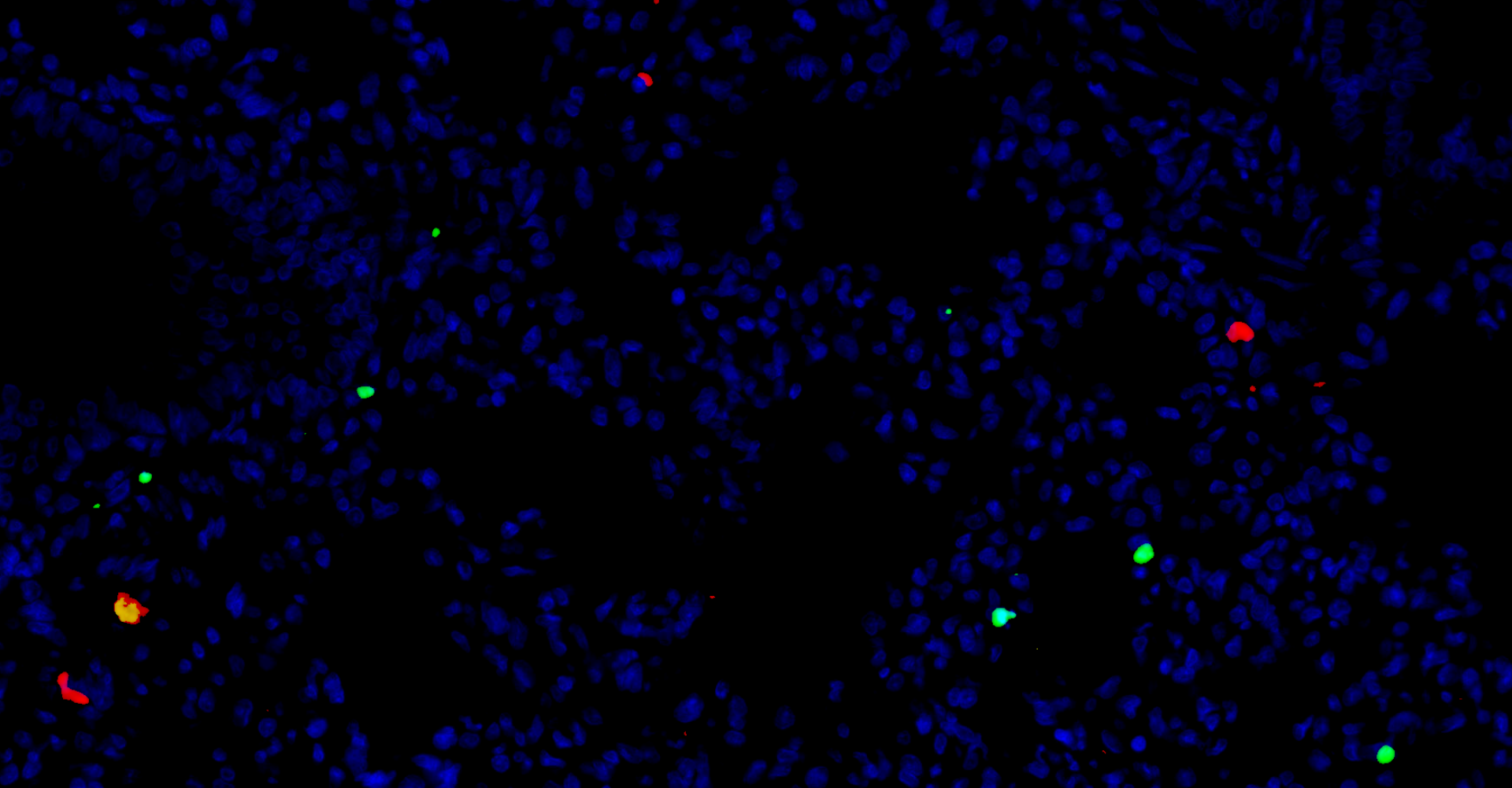

Supplement: Supplementary file 1 [file nutrients-17-02242-s001.zip › Figure S2 Original images/figure6-N-1 TUNEL-LY6G_40.0x.tif]

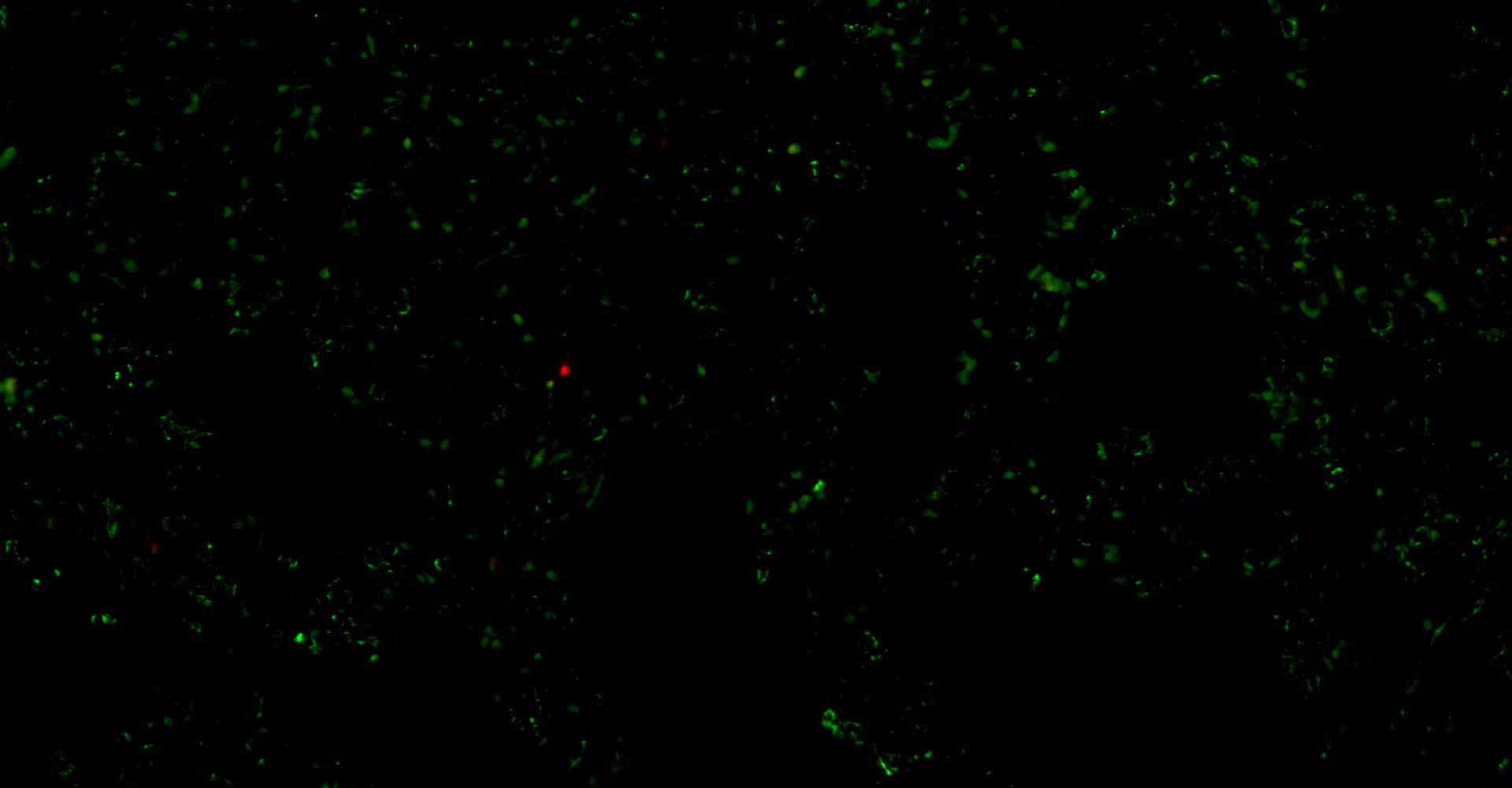

Supplement: Supplementary file 1 [file nutrients-17-02242-s001.zip › Figure S2 Original images/figure6-N-2 LY6G-ACH4_40.0x.tif]

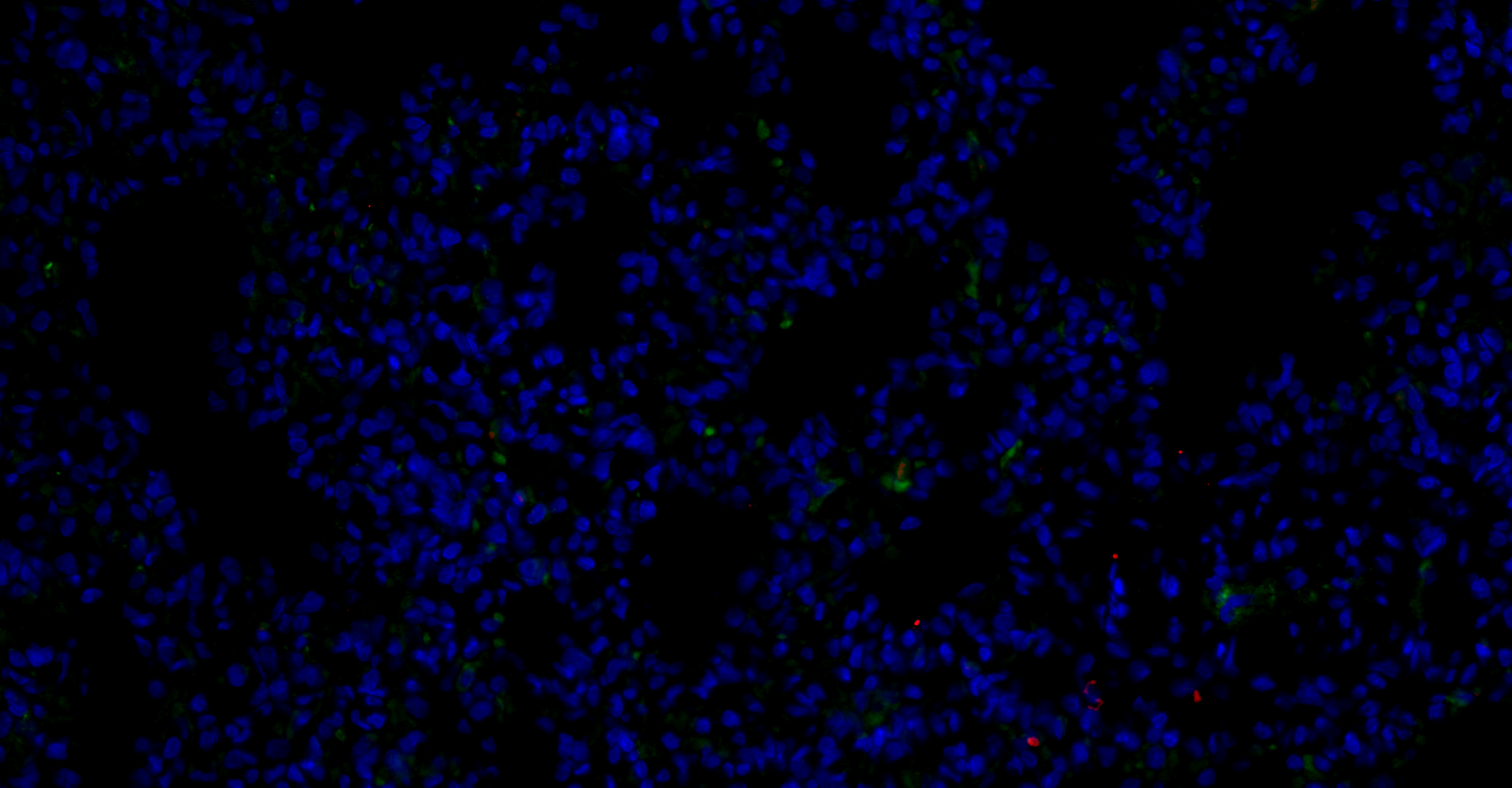

Supplement: Supplementary file 1 [file nutrients-17-02242-s001.zip › Figure S2 Original images/figure6-N-2 LY6G-CITH3_40.0x.tif]

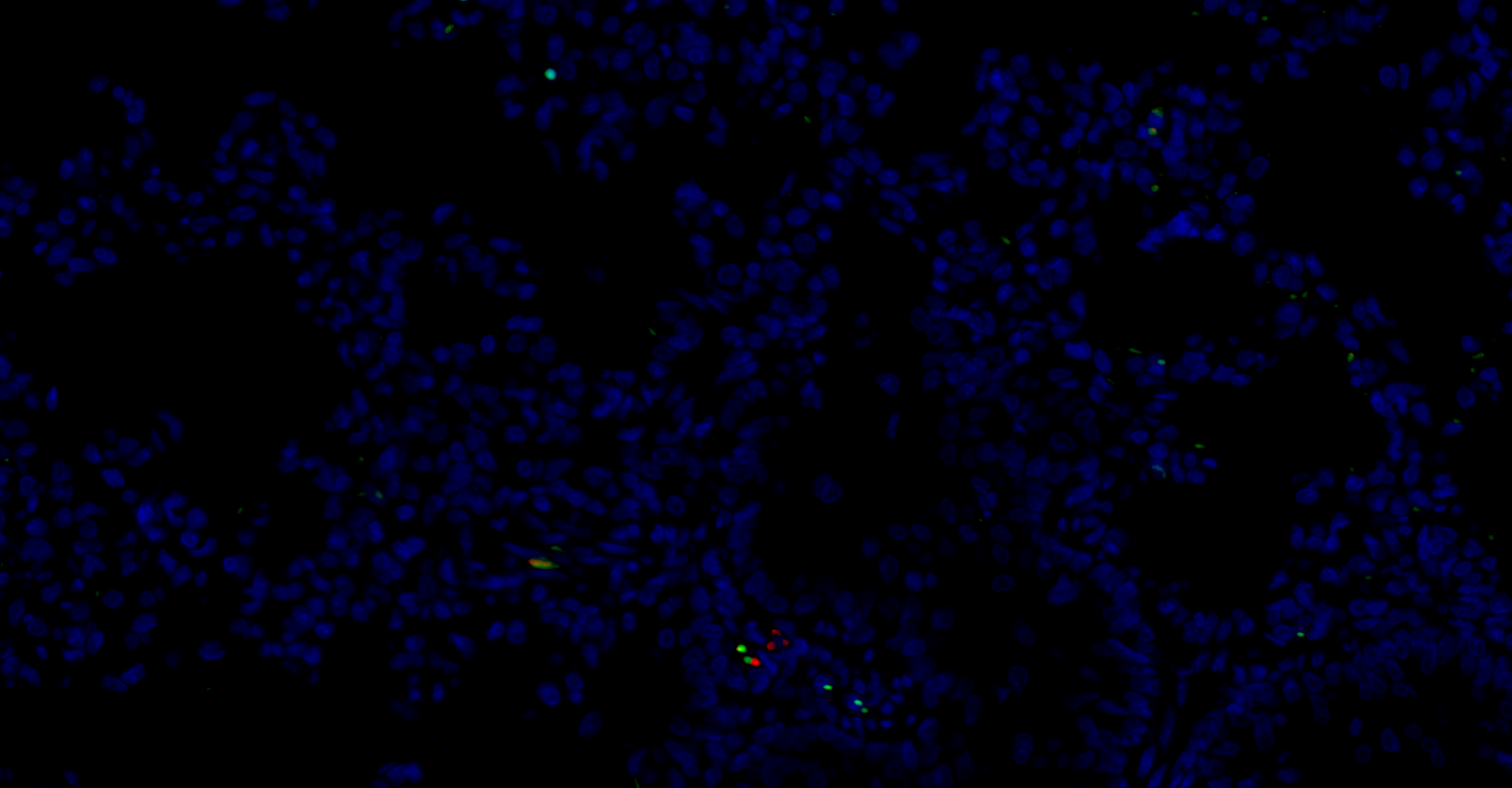

Supplement: Supplementary file 1 [file nutrients-17-02242-s001.zip › Figure S2 Original images/figure6-N-2 TUNEL-LY6G_40.0x.tif]

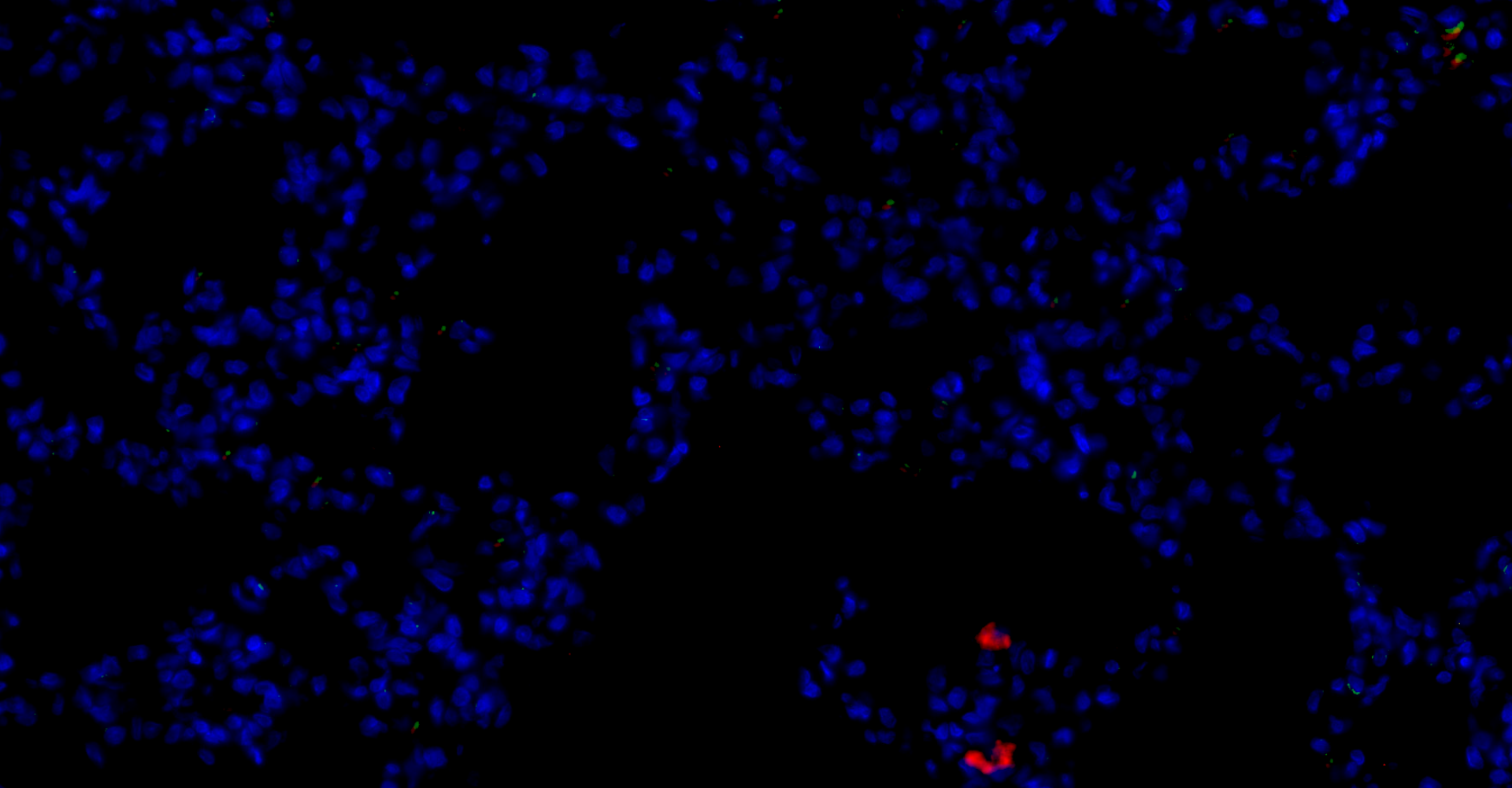

Supplement: Supplementary file 1 [file nutrients-17-02242-s001.zip › Figure S2 Original images/figure6-N-3 LY6G-ACH4_40.0x.tif]

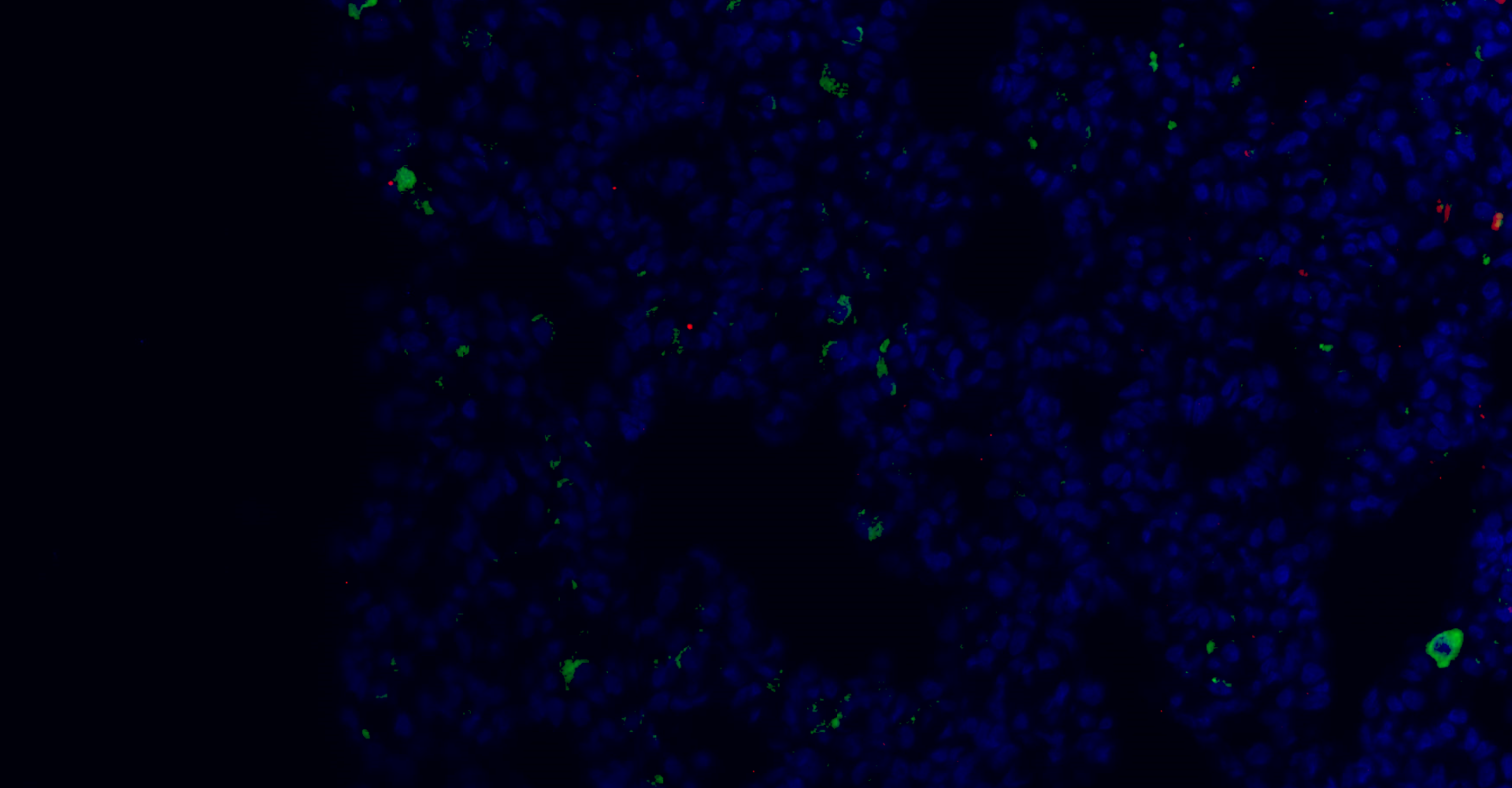

Supplement: Supplementary file 1 [file nutrients-17-02242-s001.zip › Figure S2 Original images/figure6-N-3 LY6G-CITH3_40.0x.tif]

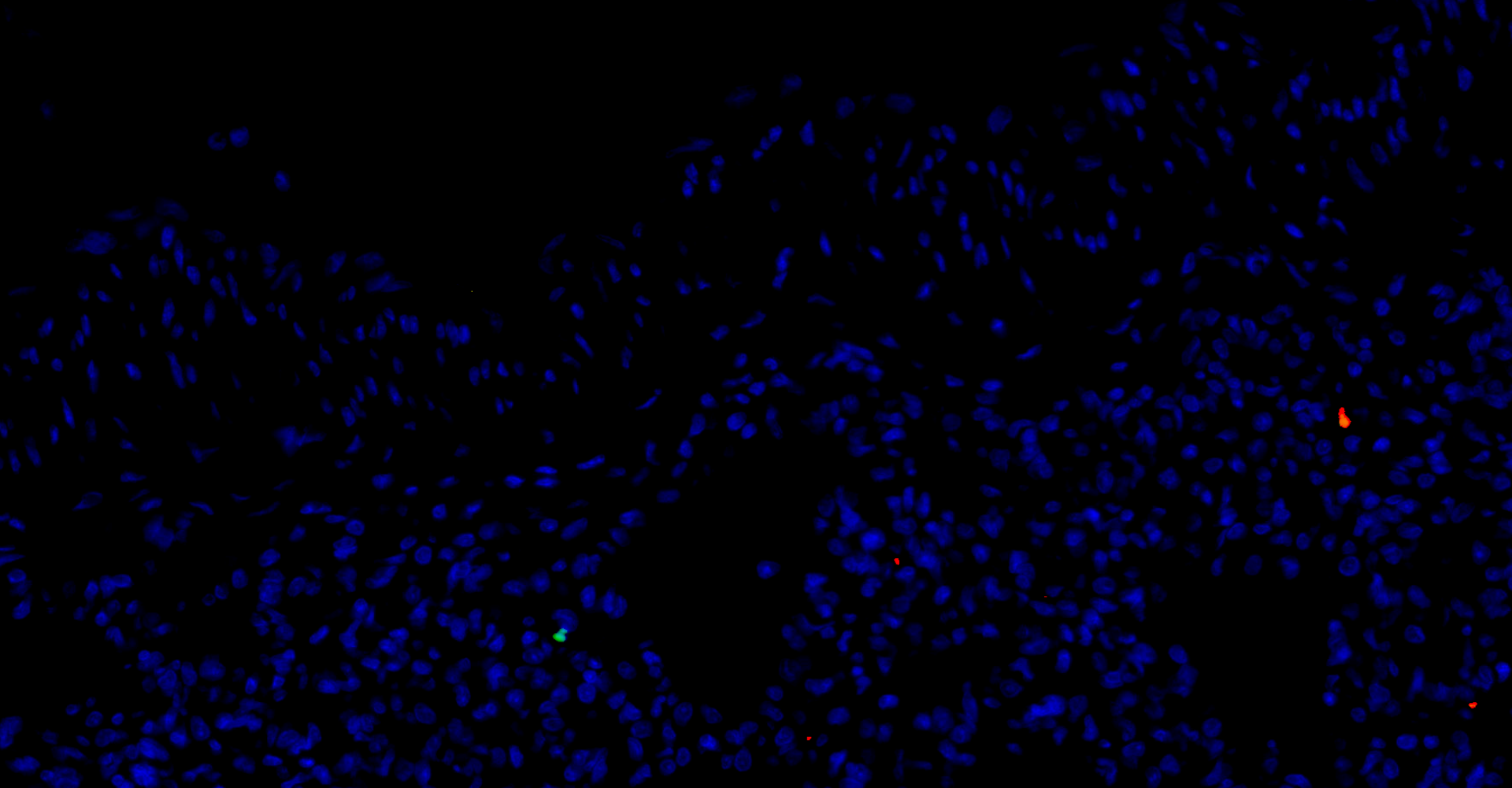

Supplement: Supplementary file 1 [file nutrients-17-02242-s001.zip › Figure S2 Original images/figure6-N-3 TUNEL-LY6G_40.0x.tif]

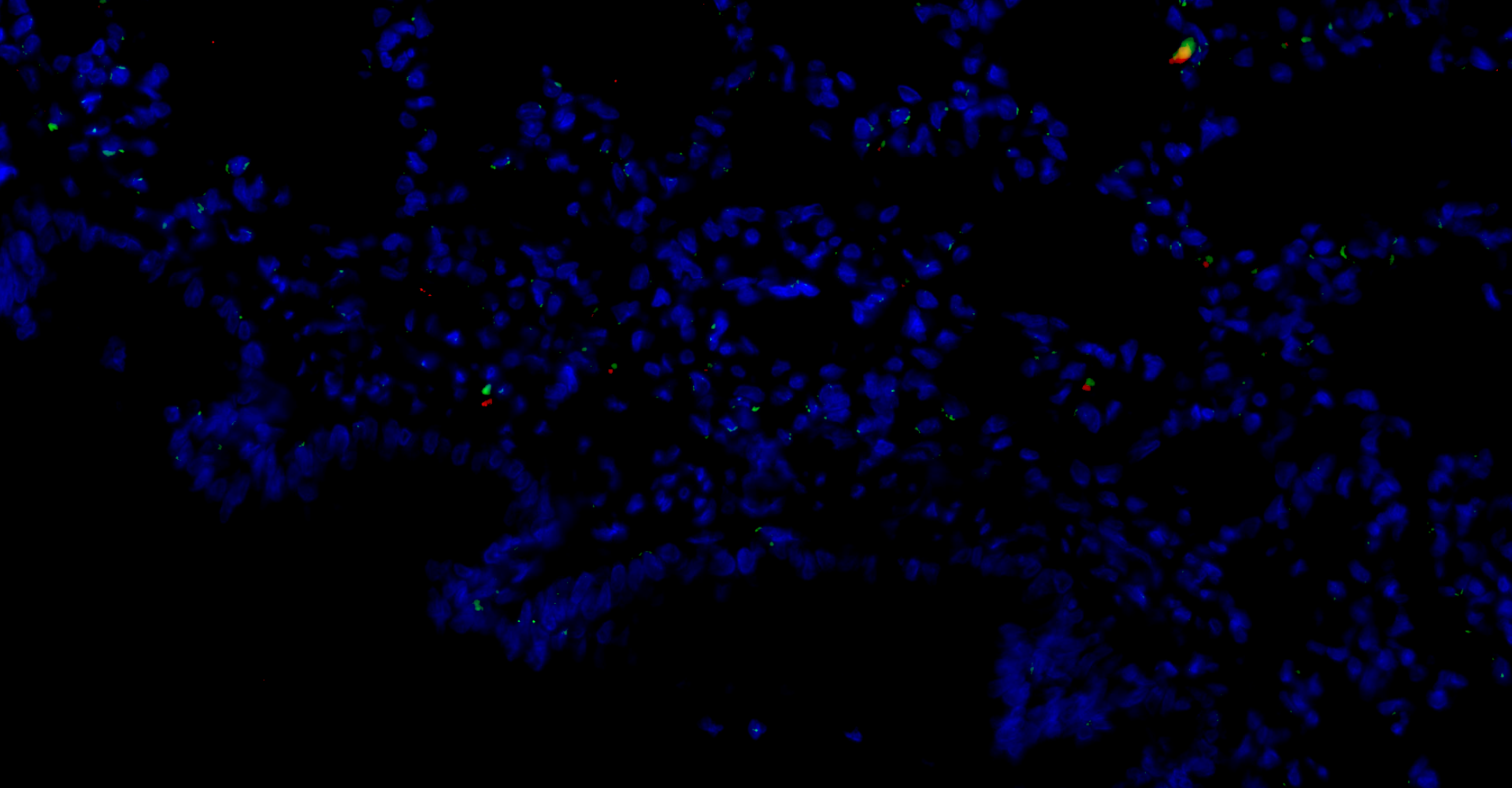

Supplement: Supplementary file 1 [file nutrients-17-02242-s001.zip › Figure S2 Original images/figure6-N-4 LY6G-ACH4_40.0x.tif]
